# Enantioconvergent access to 1,1-diarylmethine silanes and germanes through nickel/photoredox-catalyzed cross-electrophile coupling

**DOI:** 10.1039/d6sc05170g

**Published:** 2026-07-09

**Authors:** Emilio G. A. Acuña-Bolomey, Nektarios Kranidiotis-Hisatomi, Elisabeth Irran, Martin Oestreich

**Affiliations:** a Institut für Chemie, Technische Universität Berlin Strasse des 17. Juni 115 10623 Berlin Germany martin.oestreich@tu-berlin.de

## Abstract

An enantioconvergent nickel/photoredox-catalyzed C(sp^3^)–C(sp^2^) cross-electrophile coupling of α-chlorobenzyl silanes and germanes with aryl bromides is reported. This protocol makes use of a Hantzsch ester as an organic reductant and furnishes chiral 1,1-diarylmethine silanes and germanes with high enantioselectivity under mild reaction conditions. This work expands the development of enantioconvergent cross-electrophile coupling reactions adjacent to silicon and germanium atoms and provides direct access to valuable chiral benzhydryl silane and germane motifs.

## Abstract 2

An enantioconvergent nickel/photoredox cross-electrophile coupling of α-chlorobenzyl silanes and germanes with aryl bromides affords chiral 1,1-diarylmethine silanes and germanes with high enantioselectivity using a Hantzsch ester as the reductant.

## Graphical abstract

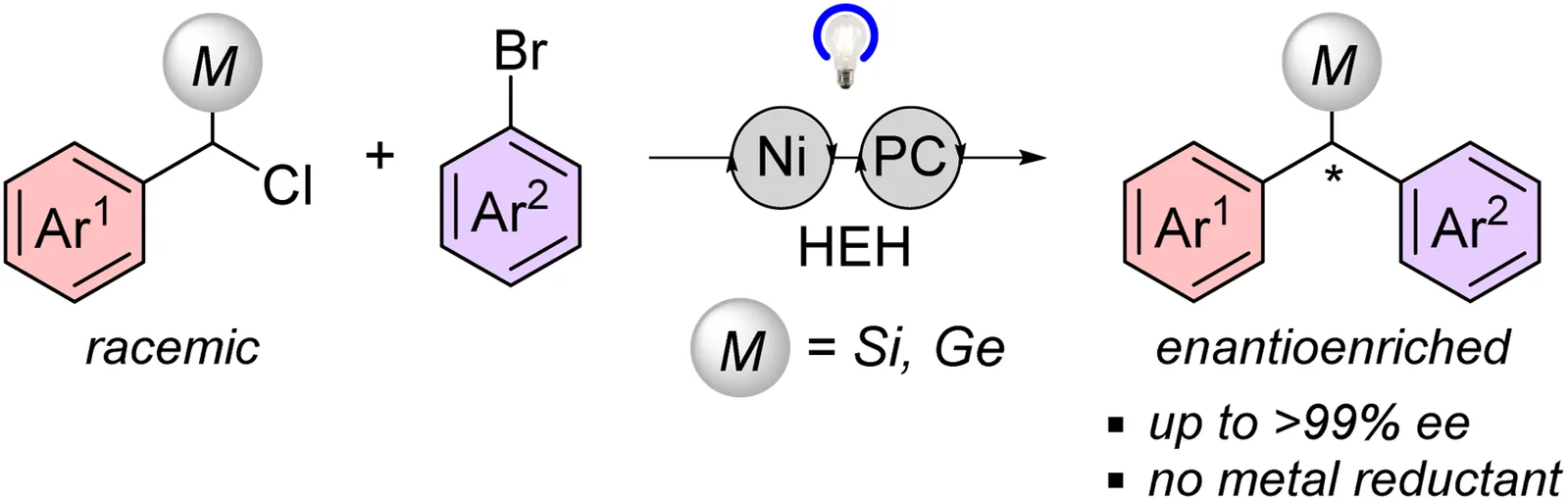

## Introduction

**Scheme 1:**
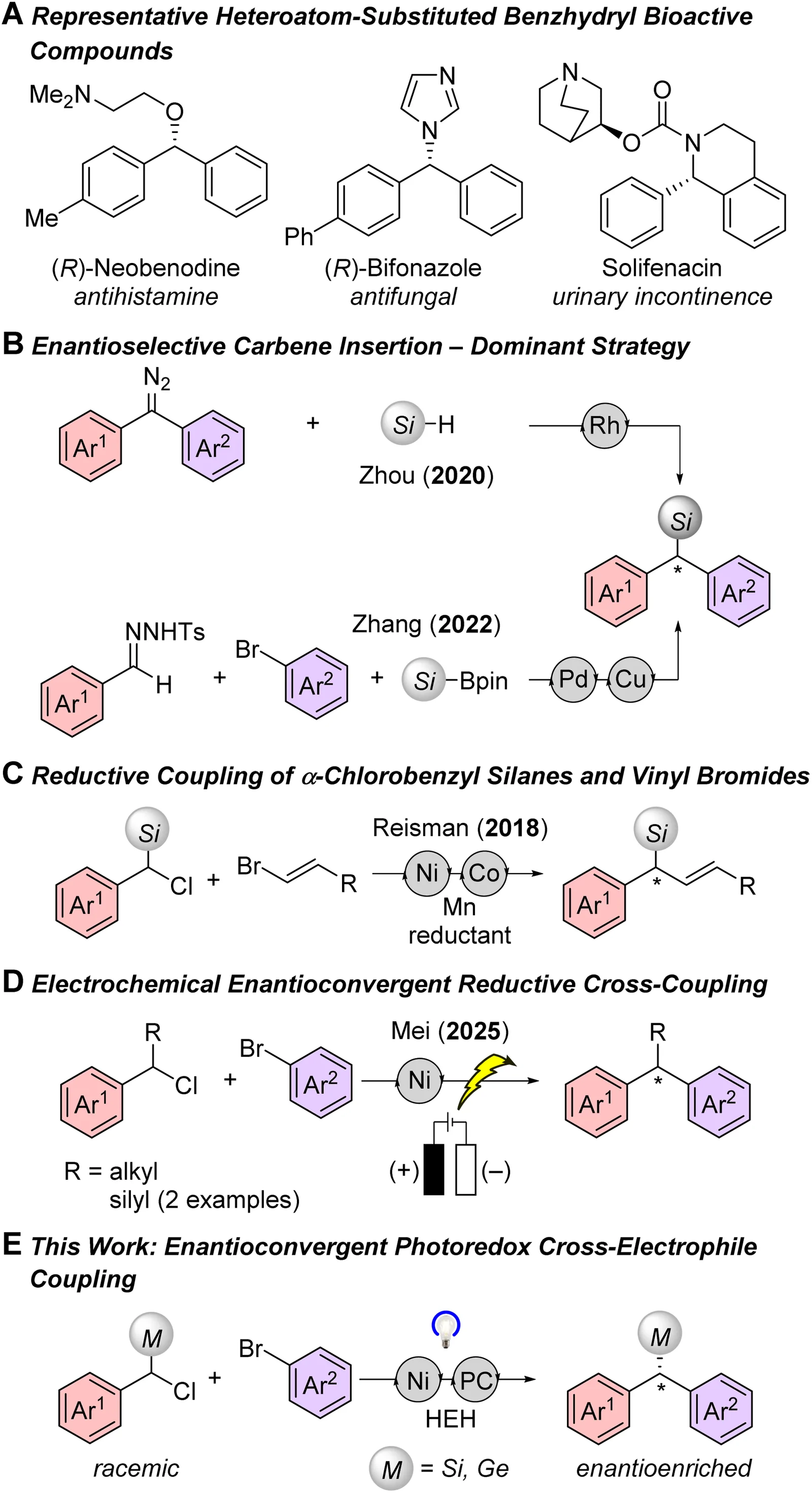
State of the art for the enantioselective synthesis of benzhydryl silanes.

Chiral 1,1-diaryl compounds represent an important scaffold found in numerous biologically active molecules. As such, the development of enantioselective methods to access these motifs in high enantiopurity has attracted considerable attention (Scheme 1A).^1–4^ For example, enantioenriched 1,1-diarylalkanes have been accessed through transition-metal-catalyzed strategies.^5–7^ In this context, significant progress has been achieved toward the enantioselective synthesis of 1,1-diarylmethine derivatives bearing heteroatoms such as oxygen,^8–10^ nitrogen,^3,11,12^ and boron,^9,13,14^ among others,^15–17^ notably through desymmetrization, carbene insertion and transition-metal-catalyzed cross-coupling strategies. In contrast, procedures enabling the enantioselective synthesis of silicon-substituted benzhydryls remain comparatively underexplored. To date, such compounds have primarily been accessed through transition-metal-catalyzed insertion of diarylcarbenes into Si–H bonds (Scheme 1B). These approaches, including rhodium-catalyzed systems, have also been extended to germanium analogues by the Zhou laboratory (for a recent review on chiral organogermanes, see ref. 18).^19–21^ In addition, an alternative strategy based on the *in situ* generation of diarylcarbenes from *N*-sulfonylhydrazones under palladium catalysis has been reported by Zhang and co-workers.^22^ Despite these advances, cross-coupling approaches enabling the direct enantioselective construction of silicon-substituted benzylic stereocenters remain limited. A notable contribution was reported by the Reisman group, demonstrating a nickel-catalyzed cross-electrophile coupling between α-chlorobenzyl silanes and alkenyl bromides.^23^ Their system used a cobalt co-catalyst as well as manganese as the reductant, yielding enantioenriched allylic silanes (Scheme 1C). Of note, the same group had also previously disclosed a related strategy for the synthesis of enantioenriched 1,1-diarylalkanes.^5^ More recently, Mei and co-workers reported a nickel-catalyzed electrochemical cross-electrophile coupling between benzylic chlorides and aryl bromides.^24^ While this method focused on alkyl-substituted substrates, two examples using α-chlorobenzyl silanes were included (Scheme 1D).

Building on our work on nickel-catalyzed enantioconvergent cross-couplings of α-silylated/germylated alkyl and allyl halides,^25–32^ and inspired by the work of Reisman,^23^ we sought to develop an enantioconvergent arylation of α-chlorobenzyl silanes *via* cross-electrophile coupling, thus avoiding the use of preformed organometallic reagents (for selected reviews on cross-electrophile couplings, see ref. 33–42). To achieve this, we envisioned the use of a Hantzsch ester (HEH) as an organic reductant in combination with photoredox catalysis. Hantzsch esters have emerged as efficient electron donors in a variety of photocatalyzed transformations, and we have previously reported their utility in reductive cross-coupling reactions of α-silylated alkyl electrophiles with allylic sulfones.^43^ In this context, a photoredox system enabling the generation of a low-valent nickel species from HEH was considered a promising strategy.^44^ In particular, photoredox enantioconvergent cross-electrophile coupling has enabled the synthesis of enantioenriched 1,1-diarylalkanes, notably through the work of Fang and Lu.^7^

Herein we report a nickel-catalyzed enantioconvergent cross-electrophile coupling of α-chlorobenzyl silanes and aryl bromides under photoredox conditions using the Hantzsch ester as the sole reductant. The 1,1-diarylmethine silanes were obtained with excellent enantioselectivity. Furthermore, the method could be extended to α-chlorobenzyl germanes, albeit with reduced efficiency (Scheme 1E).

## Results and discussion

**Scheme 2:**
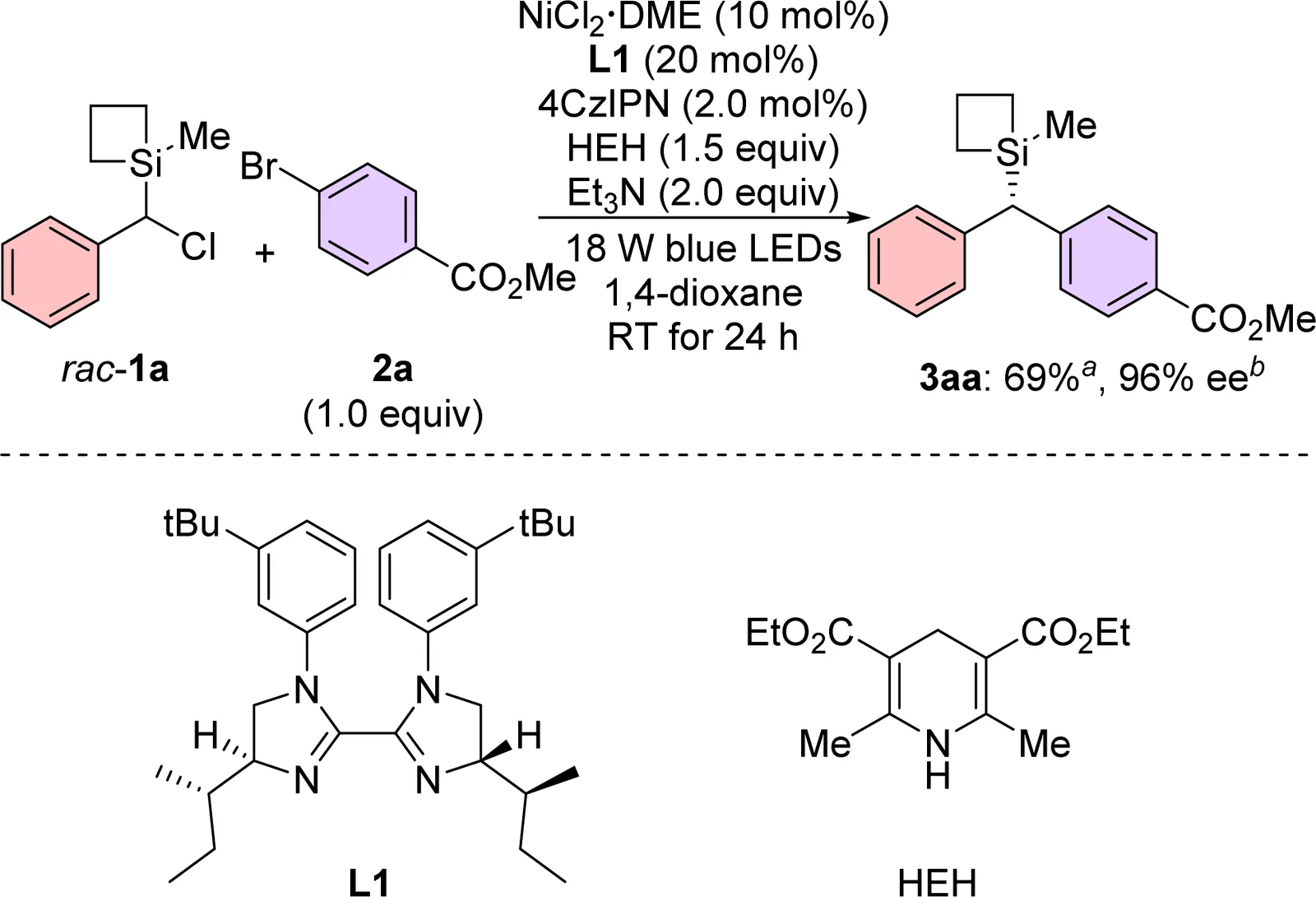
Preliminary cross-electrophile-coupling of α-chlorobenzyl silacyclobutanes. Reaction performed on a 0.10 mmol scale. ^a^ Yield determined by ^1^H NMR analysis with dibromomethane as an internal standard. ^b^ Enantiomeric excess determined by HPLC analysis on a chiral stationary phase.

Preliminary investigations in our group toward the enantioconvergent cross-electrophile coupling of α-chlorobenzyl silanes with aryl bromides under dual nickel/photoredox conditions revealed extensive side reactions, including hydrodehalogenation and homocoupling pathways. α-Chlorobenzylic silanes bearing trimethylsilyl and *tert*-butyldimethylsilyl substituents failed to provide the desired cross-coupling products under the investigated conditions (see Table S1 for details). In contrast, replacement of these bulkier groups by a silacyclobutane motif, as in *rac*-1a, enabled formation of the desired coupling product. Using aryl bromide 2a under conditions employing NiCl_2_·DME, biimidazoline (biIm) ligand L1, 4CzIPN as the photocatalyst and HEH as the reductant, product 3aa could be obtained with promising yield and high levels of enantioinduction (Scheme 2). At this stage, however, reliable isolation and analysis of the desired products remained challenging due to partial protodesilylation during purification, overlapping NMR resonances in the crude mixtures, and co-elution with the corresponding aryl bromide.

Considering these analytical challenges, we turned our attention to the corresponding α-chlorobenzyl trimethylgermanes as model substrates, whose greater C–Ge bond length relative to that to silicon was anticipated to reduce steric congestion at the benzylic center (Table 1). Using α-chlorobenzyl germane 4a and methyl 4-bromobenzoate (2a) under the conditions identified during the preliminary silane studies afforded product 5aa in 19% yield and with 91% ee (entry 1). While facing at first similar purification problems as with the siletanes, yield estimation after column chromatography was feasible by NMR analysis and allowed us to pursue our efforts until proper purification conditions could be found. Screening of alternative photocatalysts revealed 4CzIPN to be uniquely effective, as *fac*-Ir(ppy)_3_ only gave trace amounts of product while Ru(bpy)_3_(PF_6_)_2_, and Ru(bpz)_3_(PF_6_)_2_ all failed to provide detectable product formation (entries 2–4). In the absence of photocatalyst no reaction occurred (entry 5), indicating that the direct photoexcitation of the Hantzsch ester alone does not promote the reaction. The superior performance of 4CzIPN may arise from a favorable combination of excited-state oxidizing power toward HEH and reducing ability of the corresponding radical anion toward nickel intermediates. While Ru(bpz)_3_(PF_6_)_2_ should also be sufficiently oxidizing to accept an electron from HEH, the reduced 4CzIPN radical anion lies closer to the redox potential range reported for related biIm–nickel(ii) complexes,^45^ which may account for its unique efficiency in the present system. Evaluation of DMF, THF and DMA identified THF as superior to 1,4-dioxane as solvent by increasing the yield to 31% while maintaining high enantioselectivity (entries 6–8). Mixtures of DMA with THF and 1,4-dioxane in a 4 : 1 ratio provided the same results as with the ethereal solvents alone (entries 9–10). Ligand L2 bearing a cyclopentyl substituent instead of the (*S*)-*s*-butyl from L1 led to improved product formation with only slight erosion in enantioselectivity (entry 11). Finally, a nickel pre-catalyst screening of various Ni^II^ and Ni^0^ sources revealed NiBr_2_·DME to provide the best balance between yield and enantioinduction, affording product 5aa in 50% yield and 87% ee (entry 12). All other nickel pre-catalysts resulted in lower yields despite higher enantioselectivities (entries 13–15).

**Table 1:** Optimization of the enantioconvergent cross-electrophile-coupling of α-chlorobenzyl germanes^a^

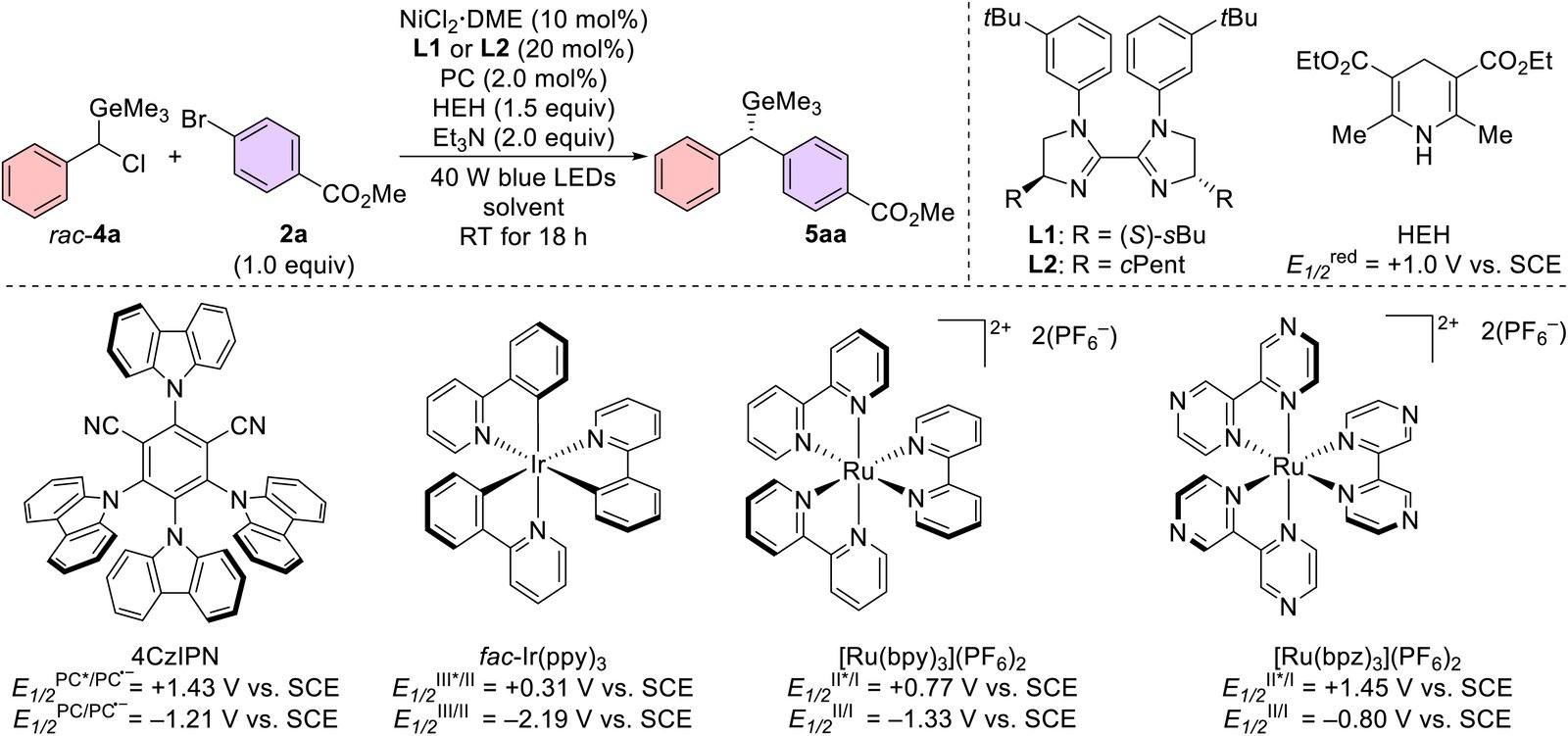						
Entry	PC	Solvent	Ligand	Pre-catalyst	Yield (%)	ee (%)^f^
1	4CzIPN	1,4-Dioxane	L1	NiCl_2_·DME	19^b^	91
2	*fac*-Ir(ppy)_3_	1,4-Dioxane	L1	NiCl_2_·DME	Trace^c^	—
3	[Ru(bpy)_3_](PF_6_)_2_	1,4-Dioxane	L1	NiCl_2_·DME	n.p.^d^	—
4	[Ru(bpz)_3_](PF_6_)_2_	1,4-Dioxane	L1	NiCl_2_·DME	n.p.^d^	—
5	No PC	1,4-Dioxane	L1	NiCl_2_·DME	n.p.^d^	—
6	4CzIPN	DMF	L1	NiCl_2_·DME	17^b^	91
7	4CzIPN	THF	L1	NiCl_2_·DME	31^b^	94
8	4CzIPN	DMA	L1	NiCl_2_·DME	19^b^	96
9	4CzIPN	DMA/THF (4 : 1)	L1	NiCl_2_·DME	31^b^	94
10	4CzIPN	DMA/1,4-dioxane (4 : 1)	L1	NiCl_2_·DME	19^b^	90
11	4CzIPN	THF	L2	NiCl_2_·DME	39^e^	89
12	4CzIPN	THF	L2	NiBr_2_·DME	50^e^	87
13	4CzIPN	THF	L2	NiBr_2_·diglyme	39^e^	93
14	4CzIPN	THF	L2	NiI_2_	32^e^	95
15	4CzIPN	THF	L2	Ni(^4-*t*Bu^stb)_3_	37^e^	96

a All reactions were performed on a 0.10 mmol scale using a Kessil A160WE Tuna Blue lamp (*λ*_max_ = 456 nm).

b Yield determined by ^1^H NMR analysis with dibromomethane as an internal standard.

c Trace amounts of product 5aa detected by GC analysis (<1%), starting materials *rac*-4a and 2a were still observed in the crude reaction mixture.

d No product detected by GC analysis, starting materials *rac*-4a and 2a were still observed in the crude reaction mixture.

e Isolated yield after purification by flash column chromatography on silica gel.

f Enantiomeric excesses were determined by HPLC analysis on a chiral stationary phase. DME = 1,2-dimethoxyethane, diglyme = 2-methoxyethyl ether, n.p. = no product, ^4-*t*Bu^stb = *trans*-4,4′-di-*tert*-butylstilbene.

**Scheme 3:**
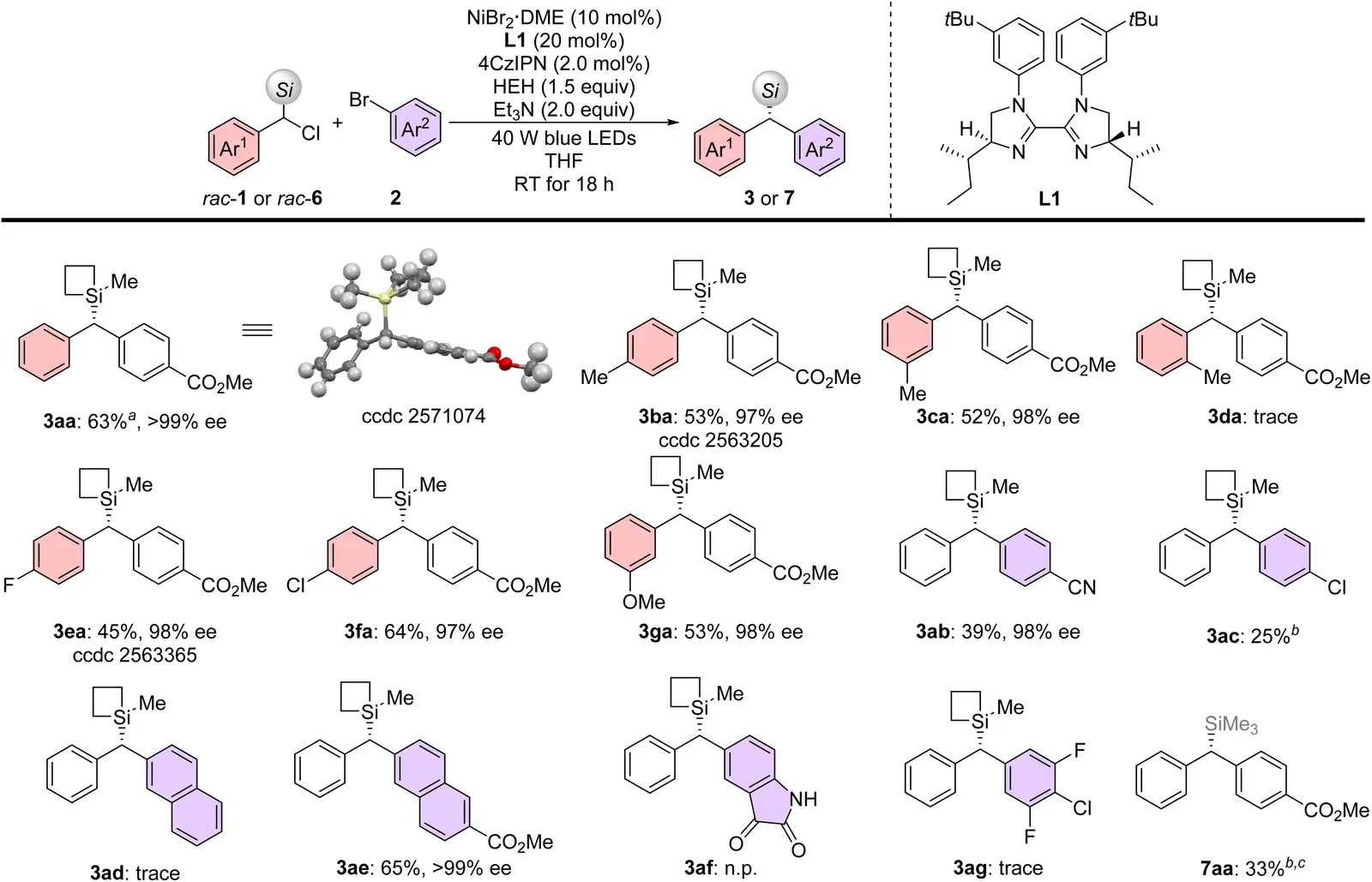
Scope of photoredox cross-electrophile coupling of α-chlorobenzyl silanes. Unless otherwise indicated, all reactions were performed on a 0.20 mmol scale. Isolated yields after purification by flash column chromatography on silica gel. Enantiomeric excesses were determined by HPLC on chiral stationary phases. ^a^ Performed on a 0.10 mmol scale. ^b^ No baseline separation could be obtained by HPLC or GC analysis on a chiral stationary phase. ^c^L2 was used instead of L1. n.p. = no product.

Having established optimal conditions with the germane model substrate, we evaluated their applicability to the silane series. Gratifyingly, only a change of ligand from L2 back to L1 was required to achieve efficient coupling. Under these conditions, silane 3aa was thus isolated in 63% yield, and with an enantiomeric excess improved to >99%. The scope of the transformation was then investigated using a range of α-chlorobenzyl silanes 1 and aryl bromides 2 (Scheme 3). Variation of the substitution pattern on the α-chlorobenzyl silane component showed that both *para-* and *meta-*substituted substrates were well tolerated while maintaining high levels of enantioselectivity (3ba and 3ca). *Ortho* substitution, however, was detrimental, with product 3da being formed in only trace amounts. Electronically varied substrates bearing *para*-fluoro, *para*-chloro, and *meta*-methoxy substituents were all compatible, affording products 3ea–ga in moderate to good yields and with excellent enantioselectivities. Notably, the chloro-substituted substrate furnished 3fa in 64% yield, demonstrating good chemoselectivity for oxidative addition in favor of the aryl bromide partner. The influence of the aryl bromide coupling partner was then examined using silane 1a. In addition to ester-substitution, a nitrile-substituted substrate provided the desired product 3ab in a synthetically useful yield with excellent enantioselectivity. In turn, 1-bromo-4-chlorobenzene afforded 3ac in low yield, consistent with the requirement for a sufficiently activated aryl bromide. This trend was further illustrated with *β*-naphthyl bromides: electron-neutral 2-bromonaphthalene (2d) gave only trace amounts of 3ad, whereas ester-substituted naphthyl bromide 2e delivered 3ae in 65% yield and >99% ee. More electron-deficient aryl bromides such as 2f and 2g were not productive, possibly due to competing SET processes under the photoredox conditions. Finally, replacement of the silacyclobutane substituent by a trimethylsilyl group was also possible by using L2 instead of L1, affording product 7aa in 33% yield. The absolute configuration of compounds 3aa, 3ba and 3ea was unambiguously determined by single-crystal X-ray diffraction analysis, and the stereochemistry of the remaining products was assigned by analogy. Overall, the benzhydryl silane products were obtained with uniformly high enantioselectivity, with all measured ee values being 97% or higher.

**Scheme 4:**
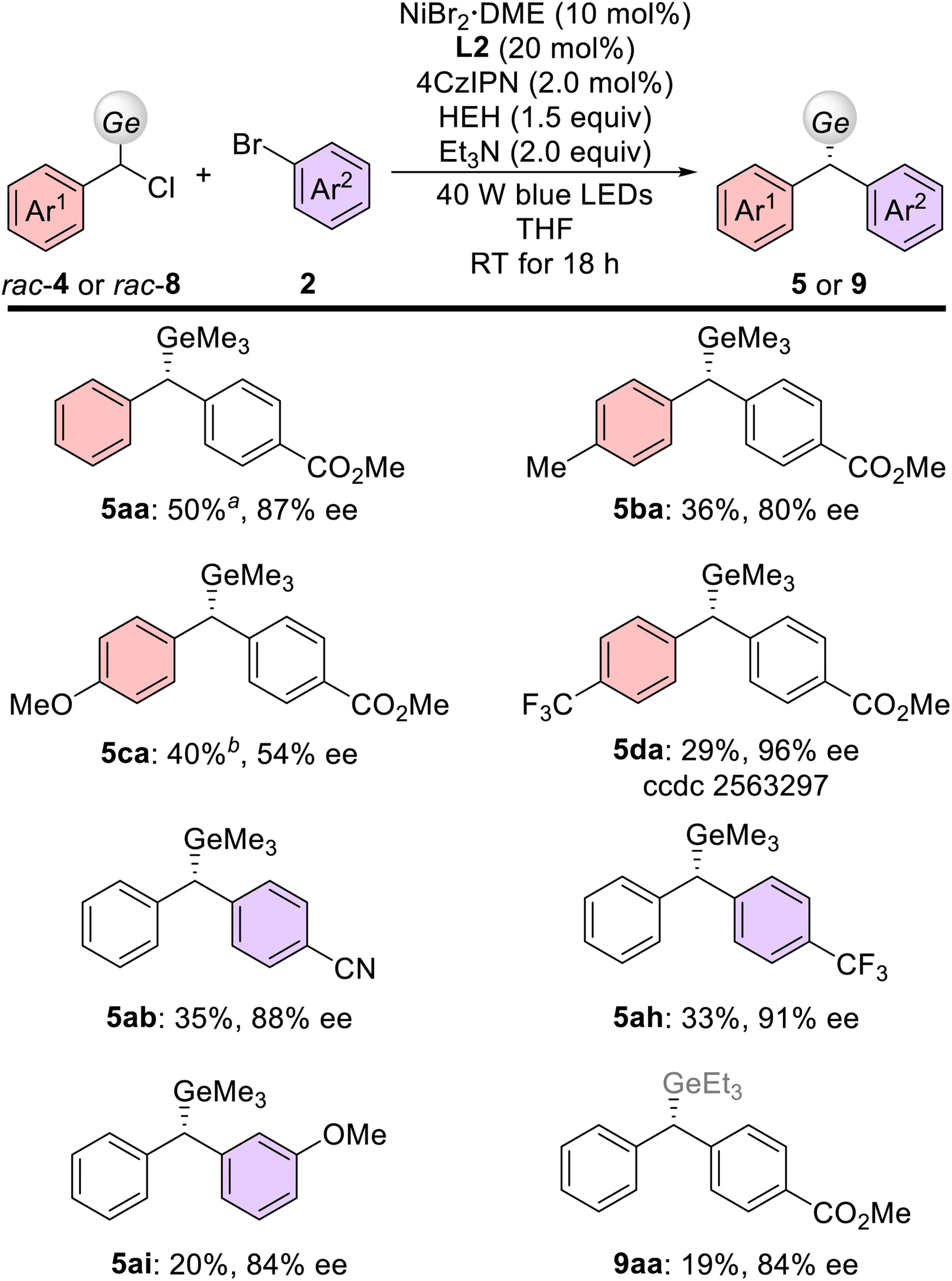
Scope of photoredox cross-electrophile coupling of α-chlorobenzyl germanes. Unless otherwise indicated, all reactions were performed on a 0.20 mmol scale. Isolated yields after purification by flash column chromatography on silica gel. Enantiomeric excesses were determined by HPLC or GC analysis on chiral stationary phases. ^a^ Performed on a 0.10 mmol scale. ^b^ Product 5ca was obtained as a mixture with its protodegermylation byproduct (9%).

The scope of α-chlorobenzyl germanes was next investigated (Scheme 4). Overall, the germane series provided lower yields than those observed for model compound 5aa. In addition, substitution on the α-chlorobenzyl germane component had a more pronounced effect on enantioselectivity compared to the corresponding silane series. In general, *para*-substitution with methyl, methoxy and trifluoromethyl groups was tolerated (5ba–da), but product 5ca was obtained with a significantly lower ee of 54% than the other examples of the series. Exposure of isolated 5ca to the standard reaction conditions for 4 h did not result in any erosion of its enantiomeric excess, indicating that the reduced enantioselectivity observed for this substrate does not arise from product racemization under the reaction conditions. Variation of the aryl bromide coupling partner was also possible using substrates bearing electron-withdrawing groups such as *para*-cyano, *para*-trifluoromethyl and *meta*-methoxy substituents (5ab–ai). Using the sterically more hindered triethylgermyl substrate 8a afforded product 9aa with 19% yield and 84% ee. A more extensive investigation of the germane scope, including unsuccessful or low-yielding examples resulting from decomposition, purification difficulties, or low reactivity, is provided in the SI (Scheme S1). The stereochemistry of the benzhydryl germanes was consistent with that of the corresponding silanes and was confirmed by single-crystal X-ray diffraction analysis of compound 5da.

Interestingly, the enantioselectivity in the germane series showed a clear dependence on the electronic nature of the benzylic aryl substituent, with the electron-withdrawing trifluoromethyl substituent providing the highest enantioinduction of 96% ee, electronically neutral substituents giving intermediate values of 80 to 87% ee, and the electron-donating methoxy substituent resulting in a significantly lower level of enantioselection of 54% ee. Under the hypothesis that the stereodetermining step indeed involves reversible radical capture by a nickel intermediate, this trend could tentatively be rationalized by electronic effects: While the 4-methoxy-substituted benzylic radical is expected to be more stabilized than the trifluoromethyl-substituted congener, it should also be more nucleophilic, potentially leading to faster radical capture by the nickel intermediate with diminished stereochemical discrimination. It may also slow down the reversibility of this step, thus limiting stereochemical equilibration between the diastereomeric benzylnickel intermediates. Finally, the slightly larger GeMe_3_ group may also promote faster reductive elimination than the corresponding silacyclobutane analog. Since even very small energy differences can have a pronounced impact on asymmetric induction, together these subtle steric and electronic effects could account for the stronger dependence of the enantioinduction on the electronic nature of the benzylic aryl substituent in the germane series. At present, however, this interpretation remains speculative.

**Scheme 5:**
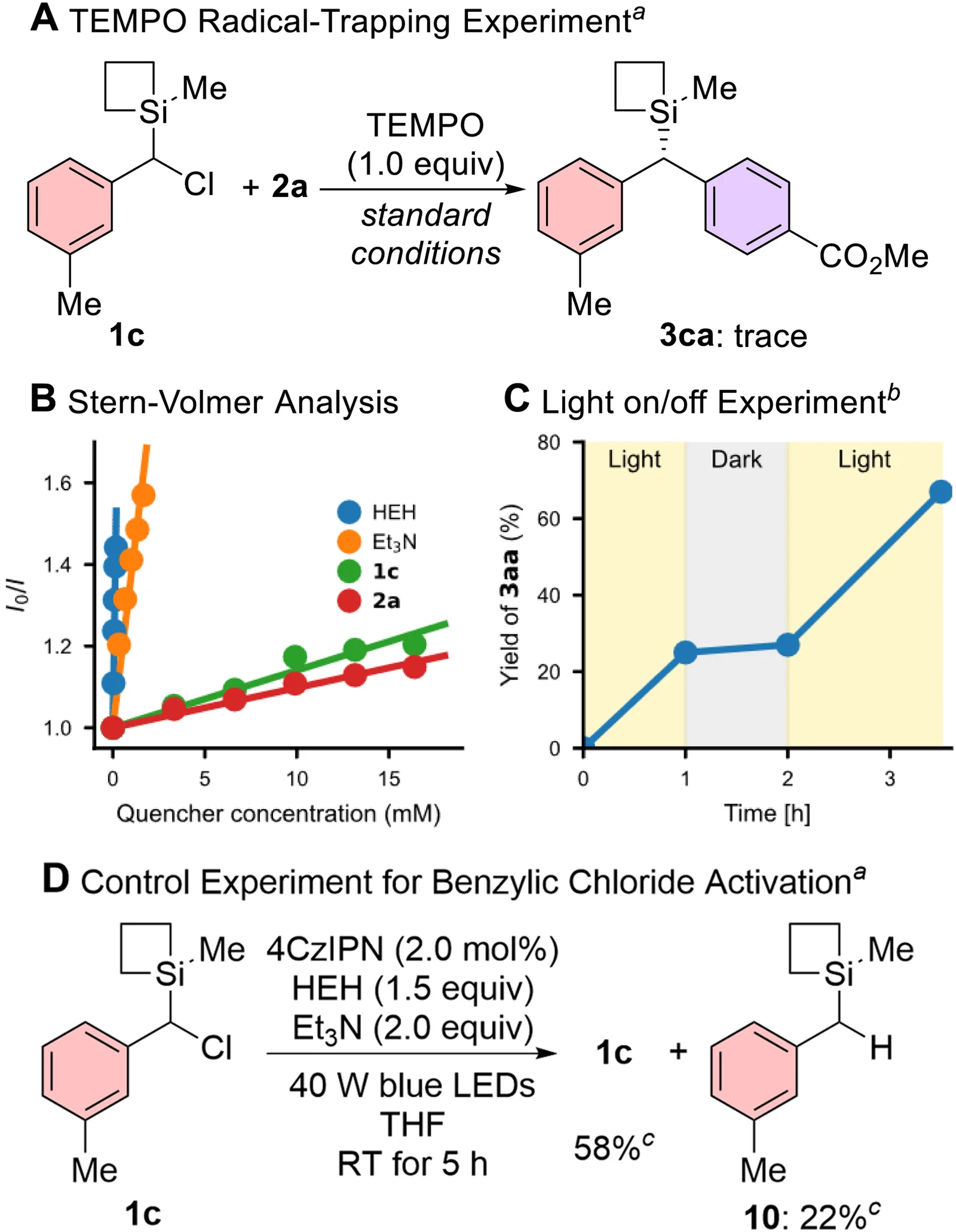
Control and mechanistic experiments. ^a^ Performed on a 0.10 mmol scale. ^b^ Performed on a 0.075 mmol scale with substrates 1a and 2a in THF-d_8_ using mesitylene as an internal standard. ^c^ Yield determined by ^1^H NMR analysis of the crude mixture after work-up using 1,2,3,4-tetrachlorobenzene as an internal standard.

To gain further insight into the reaction mechanism, radical trapping and Stern–Volmer quenching experiments were conducted (Scheme 5). Addition of TEMPO to the reaction mixture using substrate 1c suppressed product formation, although no TEMPO adducts could be detected by HRMS analysis (Scheme 5A). This result is nevertheless consistent with the involvement of radical intermediates under the reaction conditions. Stern–Volmer fluorescence quenching experiments (Scheme 5B) indicated that the Hantzsch ester and triethylamine efficiently quench the excited state of 4CzIPN, with HEH being the strongest quencher, whereas the α-chlorobenzyl substrate 1c and aryl bromide 2a showed little or no quenching under comparable conditions. These observations are consistent with a reductive quenching pathway involving the Hantzsch ester and/or triethylamine. In addition, an on/off experiment was conducted with 1a and 2a, showing that the reaction needs to be irradiated by light in order for it to proceed (Scheme 5C). Finally, irradiation of α-chlorobenzyl silane 1c in the presence of only 4CzIPN, HEH and Et_3_N resulted in partial substrate consumption and formation of the corresponding hydrodechlorination product 10 but without any trace of dimerization (Scheme 5D). Given the reported reduction potential of other benzylic chlorides (*ca.* −2.0 to −2.5 V *vs.* SCE),^46^ direct SET by the reduced photocatalyst (
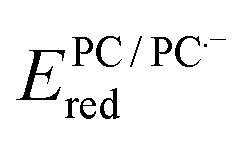
 = −1.21 V *vs.* SCE) appears unlikely.^47^ Instead, activation of the benzylic chloride *via* halogen atom abstraction (XAT) by an α-aminoalkyl radical derived from triethylamine may account for the observed reactivity.^48^ This does not, however, rule out the possibility of activation by a nickel intermediate.

**Scheme 6:**
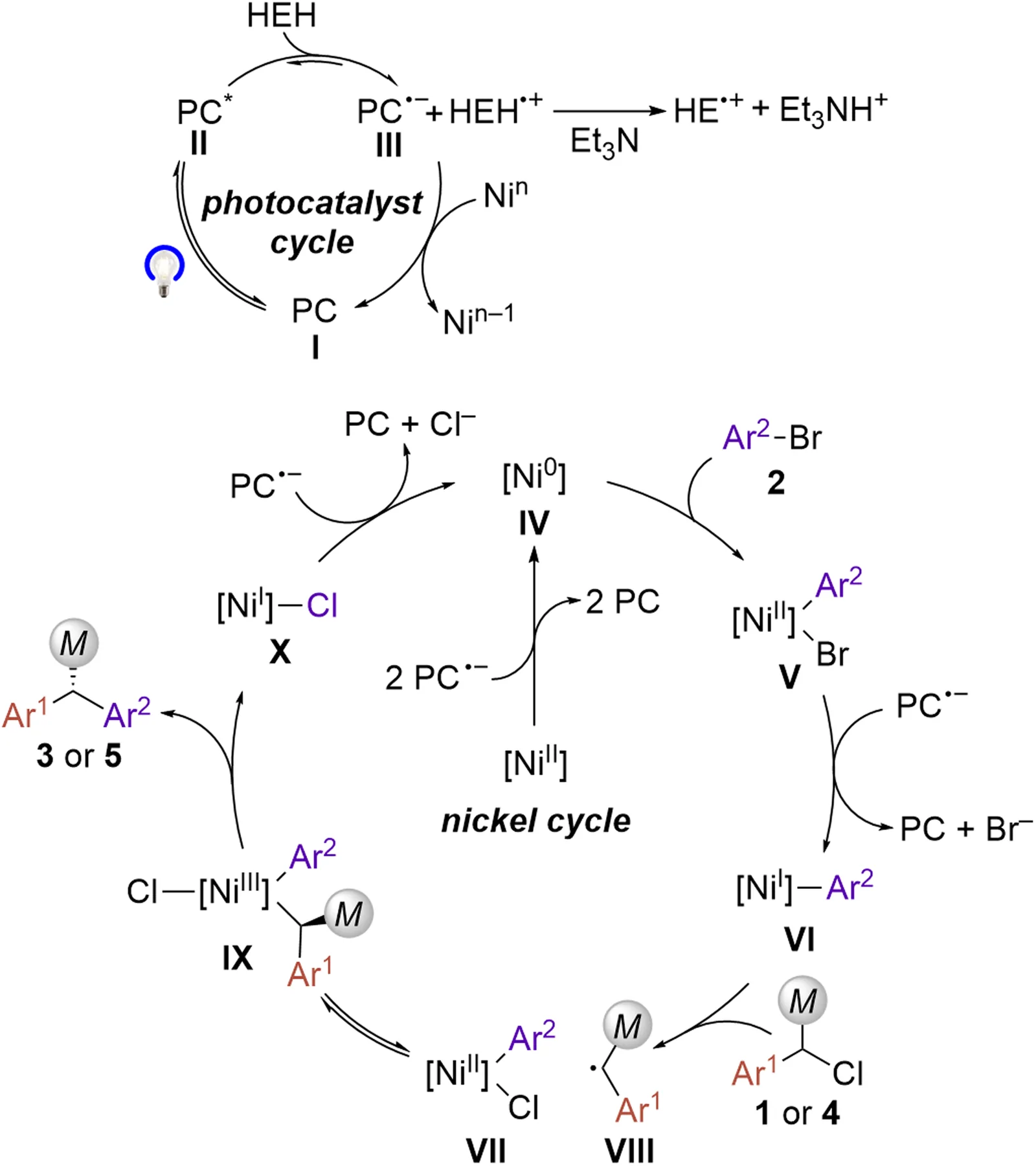
Plausible mechanism of the cross-electrophile coupling.

Based on these observations, the mechanistic experiments and literature precedent,^24,39,49,50^ we propose a plausible catalytic cycle depicted in Scheme 6: Upon blue light irradiation, photocatalyst I is promoted to its excited state II, which undergoes reductive quenching by the Hantzsch ester, forming the radical ion pair III and HEH^˙+^. The resulting radical anion III can then reduce nickel intermediates *via* single-electron transfer (SET), regenerating I, while HEH^˙+^ is deprotonated by the base and will eventually be converted to its corresponding pyridine derivative. In the nickel catalytic cycle, reduction of the nickel(ii) pre-catalyst generates the active Ni(0) species IV, which undergoes oxidative addition with aryl bromide 2, to form arylnickel(ii) intermediate V. Subsequent SET reduction affords arylnickel(i) species VI, which may in turn react with α-chlorobenzyl metalloid 1 or 4 by XAT, to generate Ni(ii) intermediate VII and benzylic radical VIII. Radical recombination then furnishes nickel(iii) intermediate IX, which undergoes reductive elimination to deliver coupling product 3 or 5 and Ni(i) species X. Final SET reduction of X by III regenerates the active nickel catalyst, closing the catalytic cycle. This proposed mechanism should be regarded as a plausible catalytic manifold. Alternative pathways involving direct oxidative addition of the benzylic chloride to Ni(0) or activation by other nickel(i) species *via* a radical chain mechanism cannot be excluded based on the available data.^33,37,51–53^

## Conclusions

In summary, we have developed an enantioconvergent nickel/photoredox-catalyzed C(sp^3^)–C(sp^2^) cross-electrophile coupling of α-chlorobenzyl silanes and germanes with aryl bromides. This methodology provides direct access to highly enantioenriched 1,1-diarylmethine silanes and germanes while avoiding the use of stoichiometric metal reductants through the use of a Hantzsch ester as the electron source. Although the reaction scope was limited to sterically less demanding silicon and germanium substituents, this method enabled the synthesis of a range of chiral benzhydryl silanes with very high enantioselectivities. The corresponding germanes could also be accessed with good enantiocontrol. Preliminary mechanistic studies are consistent with a radical-mediated pathway involving photoredox reduction of nickel intermediates under dual catalytic conditions. We anticipate that these findings will contribute to the further development of enantioconvergent cross-electrophile coupling reactions involving organosilicon and organogermanium compounds.

## Author contributions

E. G. A. A.-B., N. K.-H. and M. O. conceptualized this work. N. K.-H. achieved initial results in the silicon series, and E. G. A. A.-B. turned both the silicon and germanium series into useful methodology. E. G. A. A.-B. and N. K.-H. performed and analyzed the experiments. E. I. conducted the X-ray diffraction analysis. E. G. A. A.-B. and M. O. co-wrote the manuscript.

## Conflicts of interest

There are no conflicts to declare.

## Supplementary Material

SC-017-D6SC05170G-s001

SC-017-D6SC05170G-s002

## Data Availability

The data supporting this article have been included as part of the supplementary information (SI). Supplementary information: [reaction optimization, experimental procedures, full characterization data and copies of NMR spectra]. See DOI: https://doi.org/10.1039/d6sc05170g. CCDC 2571074, 2563205, 2563365 and 2563297 contain the supplementary crystallographic data for this paper.^54*a*–*d*^
