# Supplementary material for: Enantioconvergent access to 1,1-diarylmethine silanes and germanes through nickel/photoredox-catalyzed cross-electrophile coupling
Source: Chem Sci. 2026 Jul 9;17(34):16388–94. doi: 10.1039/d6sc05170g (PMC13370581; doi:10.1039/d6sc05170g)
Supplement: Supplementary file 1 [file SC-017-D6SC05170G-s001.pdf]

Supplementary Information (SI) for Chemical Science.
This journal is © The Royal Society of Chemistry 2026

Enantioconvergent access to 1,1-diarylmethine silanes and germanes through nickel/photoredox-catalyzed cross-electrophile coupling

Emilio G. A. Acuña-Bolomey, Nektarios Kranidiotis-Hisatomi, Elisabeth Irran and Martin Oestreich*

Institut für Chemie, Technische Universität Berlin,

Straße des 17. Juni 115, 10623 Berlin, Germany

martin.oestreich@tu-berlin.de

Supporting Information

Table of Contents

1. General Information S3

1.1 Information about the Photochemical Setup S5

2. Preliminary Studies of Cross-Electrophile Coupling S6

2.1 Table S1 .... S7

3. Additional Examples Not Included in the Main Manuscript S8

4. Experimental Details for the Preparation of Ligands S9

5. General Procedure for the Synthesis of *α*-Chlorobenzyl- Silanes and Germanes (**GP1**) S10

6. Experimental Details for the Nickel-Catalyzed Cross-Electrophile Coupling S18

6.1 General Procedure for the Nickel-Catalyzed Cross-Electrophile Coupling (**GP2**) S18

6.2 Optimization of Reaction Conditions S30

7. Control Experiments S30

7.1 Radical Trapping with TEMPO S30

7.2 Stern-Volmer Luminescence Quenching Experiment S31

7.3 Light on/off Experiment S33

7.4 Control Experiment for Benzylic Chloride Activation S33

8. Determination of the Absolute Configuration S36

8.1 Crystal Data of (*S*)-**3aa** S36

8.2 Crystal Data of (*S*)-**3ba** S44

8.3 Crystal Data of (*R*)-**3ea** S49

8.4 Crystal Data of (*R*)-**5da** S61

9. HPLC Traces S66

10. NMR Spectra S86

11. References S182

1. General Information

Air and/or water sensitive reactions were performed in oven-dried glassware using conventional Schlenk technique under a static pressure of nitrogen. Cross-electrophile coupling reactions were set up in a nitrogen filled glovebox in medium sized vials with tight caps before being taken outside to the photoreactor setup. Syringes and Pasteur pipettes were used to transfer liquids and solutions. Standard solvents and reagents were obtained from commercial suppliers (*ABCR, Acros Organics, Alfa Aesar, Angene, BLDpharm, Carbolution, chemPUR, Fluorochem, Merck, Sigma-Aldrich, Strem, Thermo Fisher Scientific, Tokyo Chemical Industry (TCI), VWR*). Technical grade solvents for extraction or chromatography (acetone, cyclohexane, ethyl acetate, diethyl ether, dichloromethane, *n*-hexane, *n*-pentane) were distilled prior to use. Unless otherwise noted, all commercially available reagents were used as received, including $NiBr_2 \cdot$DME (*BLDpharm*) and *N,N*-dimethylacetamide (DMA, 99.5% extra dry over molecular sieves, AcroSeal®, *Thermo Scientific Chemicals*). 1,4-Dioxane and THF were dried over sodium/benzophenone and freshly distilled prior to use. Solvents in the glovebox were degassed using freeze-thaw-pump technique before being brought inside a nitrogen-filled glovebox and stored over 4 Å molecular sieves. $CDCl_3$ was obtained from commercial suppliers and used as received. Flash column chromatography was performed on Grace silica gel 60 (40–63 µm, 230–400 mesh, ASTM) using the indicated solvents. Analytical thin layer chromatography (TLC) was performed with *Macherey-Nagel* Alugram Xtra SIL G/UV_{254} silica gel 60 pre-coated aluminum-backed plates (200 µm layer thickness). Product spots were visualized under UV light (λ_{max}= 254 nm) and/or by staining with a ceric ammonium molybdate solution (Seebach stain) or a potassium permanganate solution. Analytical gas liquid chromatography (GLC) was performed on a *Varian* 430-GC gas chromatograph equipped with a *Varian* Factor Four Capillary column (30 m × 0.25 mm, 0.25 µm film thickness) using the following program: N_2 carrier gas, injection temperature 250 °C, detector temperature 250 °C, flow rate: 4 mL/min; temperature program: start temperature 40 °C, heating rate 15 °C/min, end temperature 250 °C for 5 min, unless otherwise stated. Analytical high performance liquid chromatography (HPLC) was performed on an *Agilent Technologies* 1290 Infinity instrument equipped with a chiral stationary phase using *Daicel Chiralcel* or *Chiralpak* columns, using *n*-heptane/isopropanol mixtures as the mobile phase. Analytical chiral GC was performed on an *Agilent* gas chromatograph equipped with a *Supelco Astec CHIRALDEX B-DM* column (30 m × 250 µm × 0.12 µm film thickness), injection temperature 240 °C, detector temperature 300 °C, column temperature 60 °C for 30 min, then 0.5 °C/min until 175 °C, hold 20 min, then 3 °C/min until 200 °C, flow rate 1.3 mL/min. Gas-liquid chromatography mass spectrometry (GLC-MS) measurements were conducted on an Agilent Technologies 5975C TAD – GC/MSD-System with electron impact ionization (EI) connected to a fused silica HP-5ms capillary column (length: 30 m, inner diameter: 0.25 mm, thickness of the stationary phase: 0.25 µm). Measurements were performed using the following protocol: Carrier gas: He, injector temperature: 300

°C, detector temperature: 250 °C, flow rate: 0.8 mL/min, temperature program: starting temperature: 40 °C, heating rate: 20 °C/min, final temperature: 250 °C for 10 min. Nuclear magnetic resonance (NMR) spectra were recorded in $CDCl_3$ on a *Bruker* AV 400, or AV 500 instrument with the deuterated solvent acting as an internal deuterium lock. Chemical shifts are reported in parts per million (ppm) and are referenced to the residual solvent resonance as the internal standard ($CHCl_3$: δ = 7.26 ppm for ^{1}H NMR and $CDCl_3$: δ = 77.16 ppm for ^{13}C NMR). NMR assignments were performed using COSY, HSQC, HMQC and HMBC experiments. The ^{29}Si nuclei is referenced in compliance with the unified scale for NMR chemical shifts as recommended by the IUPAC stating the chemical shift relative to Me_4Si. Data are reported as follows: chemical shift, multiplicity (s = singlet, d = doublet, t = triplet, q = quartet, quint = quintet, sext = sextet, m = multiplet, and combinations thereof), coupling constants (Hz), and integration. High resolution mass spectra (HRMS) were recorded on a *Thermo Scientific* LTQ Orbitrap XL by *the Analytical Facility* at the *Institut für Chemie, Technische Universität Berlin* using atmospheric-pressure chemical ionization (APCI) methods. Data for single crystal structure determination was collected with an Agilent SuperNova diffractometer equipped with a CCD area Atlas detector and a mirror monochromator by using the Cu-Kα radiation (λ = 1.5418 Å). Data collection, cell refinement, and data reduction were performed using CrysAlisPro. Structures were solved using SHELXS and refined using SHELXT. Optical rotations were measured on a Schmidt & Haensch Polartronic H532 Polarimeter at the wavelength of the sodium D line (λ = 589 nm) at 20 °C and with concentration *c* given in g/100 mL. IR spectra were recorded on an Agilent Cary 630 FTIR using MicroLab PC and analyzed with Agilent Resolutions. Emission spectra were recorded on a Duetta™ Fluorescence and Absorbance Spectrometer (HORIBA Scientific).

1.1 Information about the Photochemical Setup

Photoreactions were carried out using a Kessil A160WE Tuna Blue lamp (40 W) at 100% intensity in actinic setting. The EvoluChem PhotoRedOx Box™ from HepatoChem (US Patent #10,906,022) was used as photoreactor, its inbuilt fan enabling room temperature reactions as well as running up to 8 reactions simultaneously in medium sized vials.

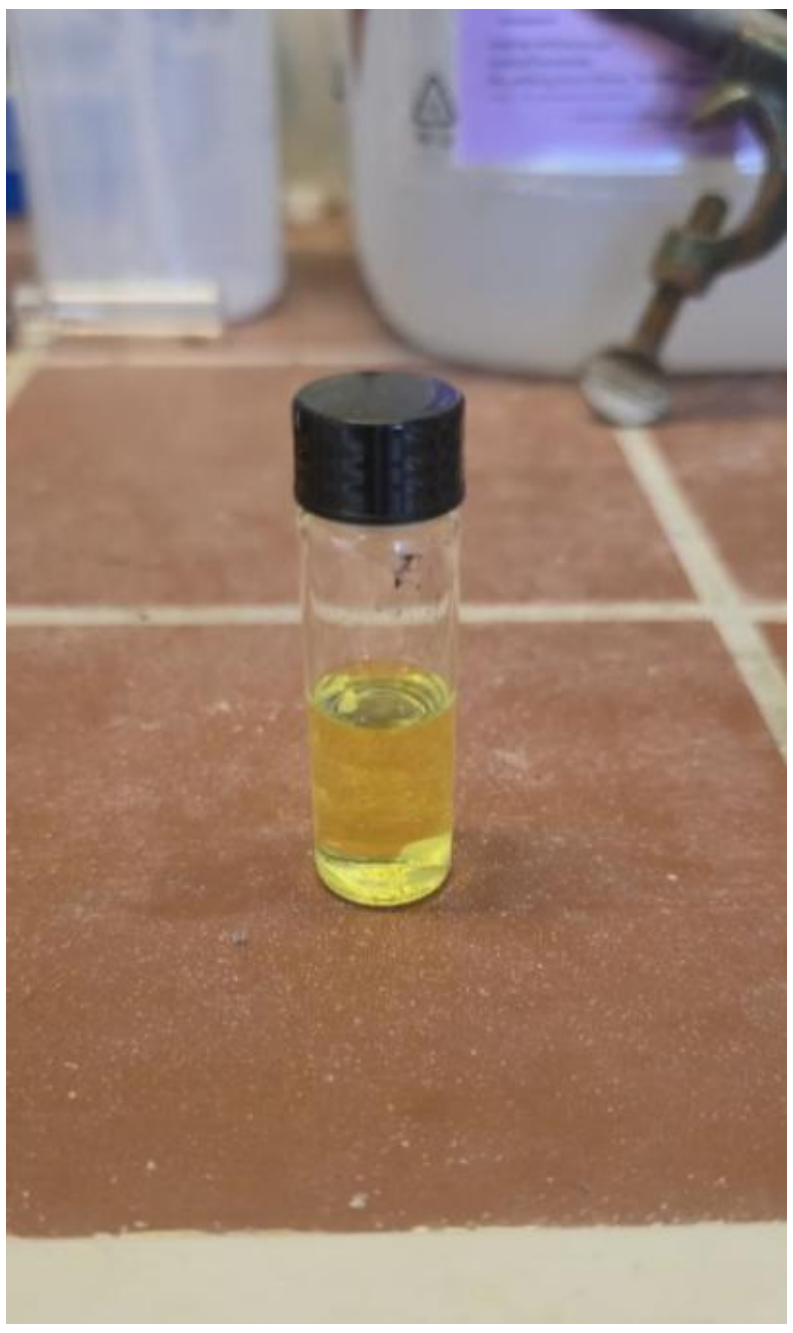

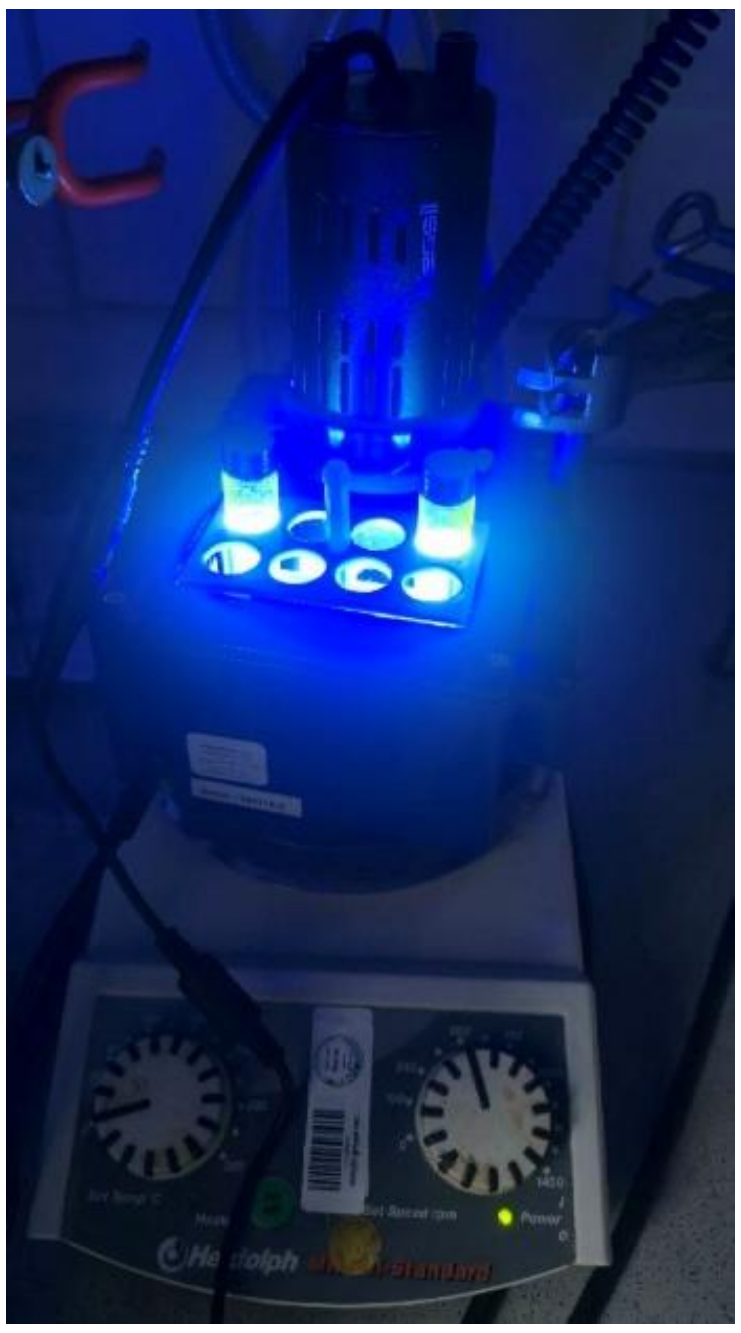

2. Preliminary Studies of Cross-Electrophile Coupling

Preliminary optimization studies were conducted by N. K.-H. as part of unpublished work in our group. Selected results are summarized in Table S1. Initial experiments were performed by using Fang and Lu's reported conditions on the enantioselective cross-coupling of benzylic chlorides and aryl halides.[S1] Under these conditions depicted in entry 1, trimethylsilyl-substituted substrate *rac*-**6a** did not afford the desired cross-coupled product. Instead homocoupling (**S12**) and hydrodechlorination (**S13**) products of the substrate were observed, as well as hydrodebromination (**S13**) and chlorodebromination (**S15**) of the aryl halide. Switching to 4CzIPN as the catalyst and triethylamine as the base (entry 2) also did not lead to detectable product formation. Evaluation of the substituent at silicon revealed a strong influence on reactivity. Installation of a bulkier silyl group as in *rac*-**S11a** (entry 3) did not provide the desired product, whereas using a strained silacyclobutane substituent (*rac*-**1a**, entry 4) allowed detection of the product by HRMS. Subsequent variation of the aryl bromide loading indicated that using 1.0 equiv minimized the formation of side products relative to the desired product (entries 5–7). Finally, a ligand screening was conducted, with BiIm ligands **L1** and **SL3** being able to give product **3aa** in 69% and 45% yields respectively (entries 8 and 9), both with excellent but opposite enantioselectivity due to the opposite configuration of the ligand backbone. At this stage, however, the product could not be isolated in analytically pure form, as its polarity was nearly identical to that of the unreacted aryl bromide and its by-products **S13** and **S14**, preventing purification by column chromatography or preparative TLC.

2.1 Table S1.

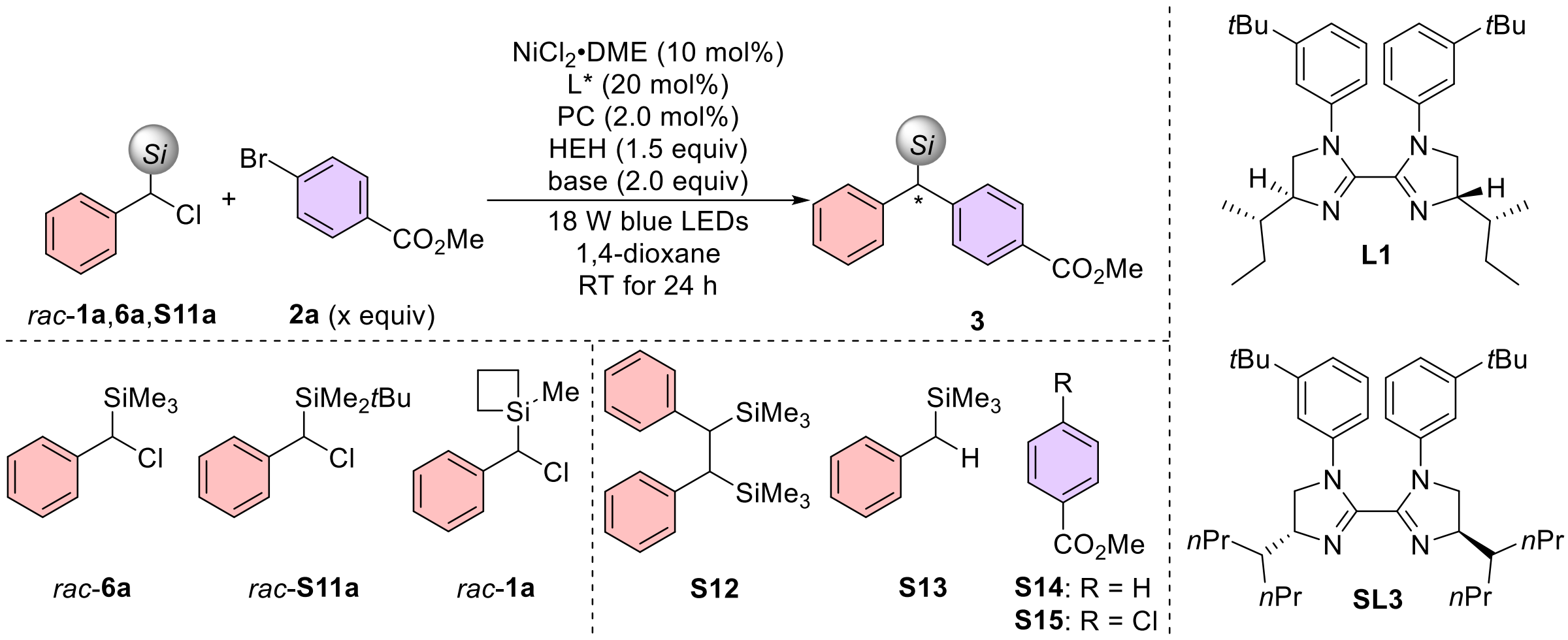

entry	substrate	equiv of **2a**	ligand	photocatalyst	base	product	ratio[c] (2a+S14+S15):2	yield[d] (%)	ee[e] (%)
1[a]	*rac*-**6a**	2.0	**L1**	[Ir(dFCF$_3$ppy)$_2$(dtbpy)]PF$_6$	DIPEA	**3ba**	–	n.p.	–
2[a]	*rac*-**6a**	2.0	**L1**	4CzIPN	Et$_3$N	**3ba**	–	n.p.	–
3[a]	*rac*-**S11a**	2.0	**L1**	4CzIPN	Et$_3$N	**S3ca**	–	n.p.	–
4[a]	*rac*-**1a**	2.0	**L1**	4CzIPN	Et$_3$N	**3aa**	–	HRMS	–
5[b]	*rac*-**1a**	2.0	**L1**	4CzIPN	Et$_3$N	**3aa**	41:59	–	
6[b]	*rac*-**1a**	1.5	**L1**	4CzIPN	Et$_3$N	**3ba**	38:62	–	–
7[b]	*rac*-**1a**	1.0	**L1**	4CzIPN	Et$_3$N	**3ba**	21:79	–	–
8[a]	*rac*-**1a**	1.0	**L1**	4CzIPN	DIPEA	**3ba**	–	69	96
9[a]	*rac*-**1a**	1.0	**SL3**	4CzIPN	DIPEA	**3ba**	–	45	–96

Preliminary conditions explored in the development of the enantioconvergent cross-electrophile coupling. [a] Reactions performed on a 0.10 mmol scale. [b] Reactions performed on a 0.05 mmol scale. [c] Determined by GLC analysis, side products ratio corresponds to the combined integrated areas of **2a**, **S14** and **S15**. [d] Determined by ^{1}H NMR analysis with dibromomethane as an internal standard. [e] Enantiomeric ratios were determined by HPLC on a chiral stationary phase, n.p. = no product.

3. Additional Examples Not Included in the Main Manuscript

The following examples were not included in the main manuscript and were not characterized. For these products only trace amounts of the desired products were detected. In some cases, inseparable co-elution with the corresponding aryl bromides or extensive protodegermylation prevented reliable isolation and full characterization. The synthesis and characterization of their corresponding substrates (*rac*-**4e–i**) is included under **GP1**.

Scheme S1. Additional Unsuccessful Examples of the Photoredox Cross-Electrophile Coupling with Germanes

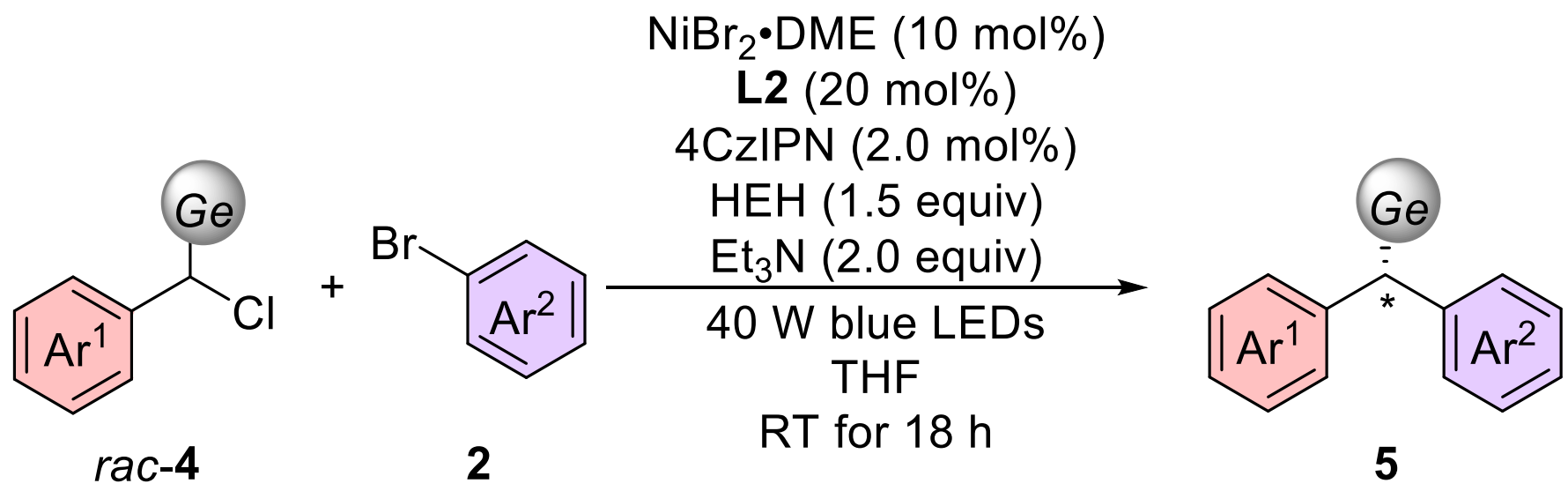

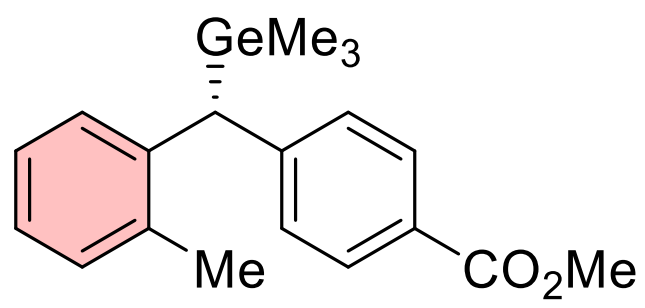

S5ea
<10% + decomp.

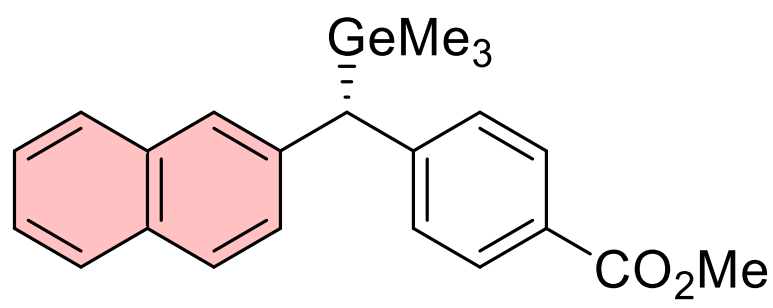

S5fa
<10% + decomp.

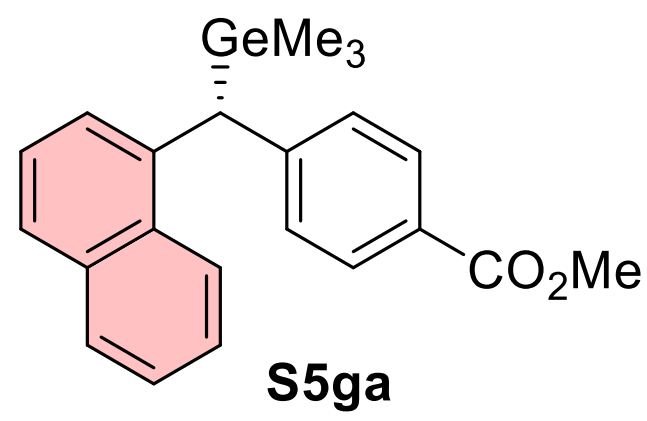

S5ga
decomp.

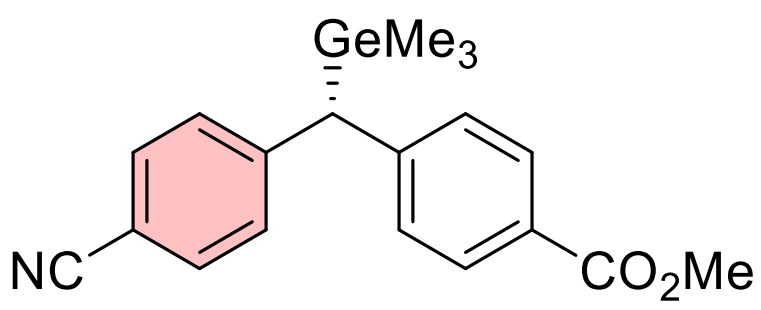

S5ha
trace

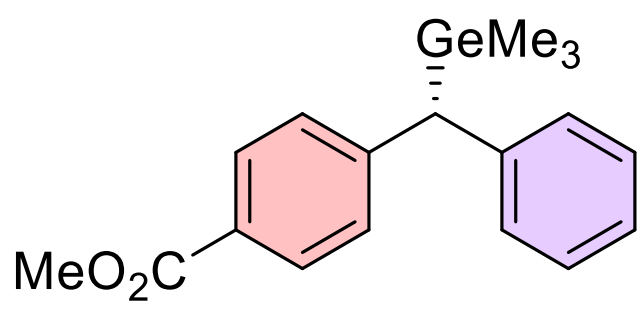

S5ij (*ent*-**5aa**)
trace

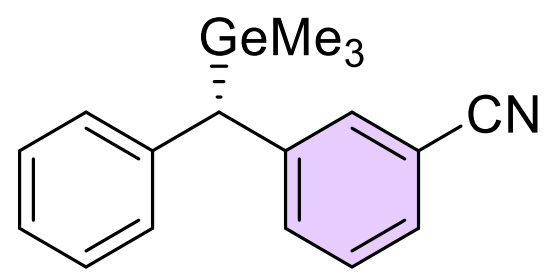

S5ak
<20%
mixture w/ Ar-Br

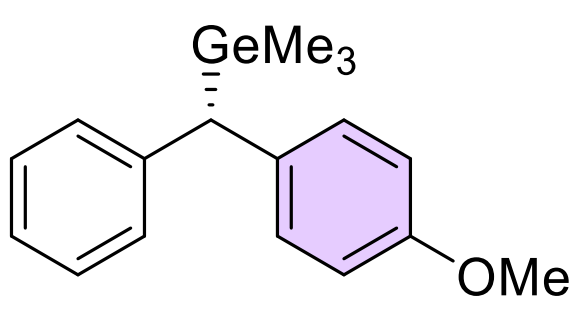

S5al
complex mixture

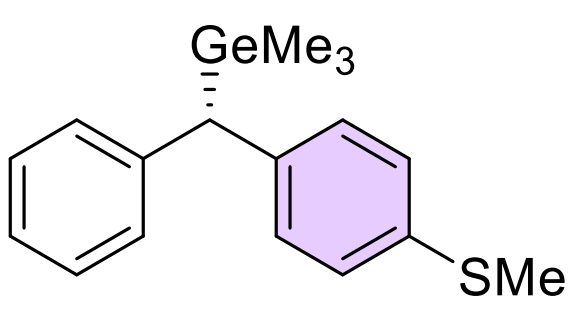

S5am
decomp.

All reactions were performed on a 0.20 mmol scale. decomp. = decomposition of the product during purification.

4. Experimental Details for the Preparation of Ligands

The synthesis of ligand **L1** was performed in previous work from our group from reported procedures.[S2–4] Ligand **L2** was prepared in an analogous way as follows:

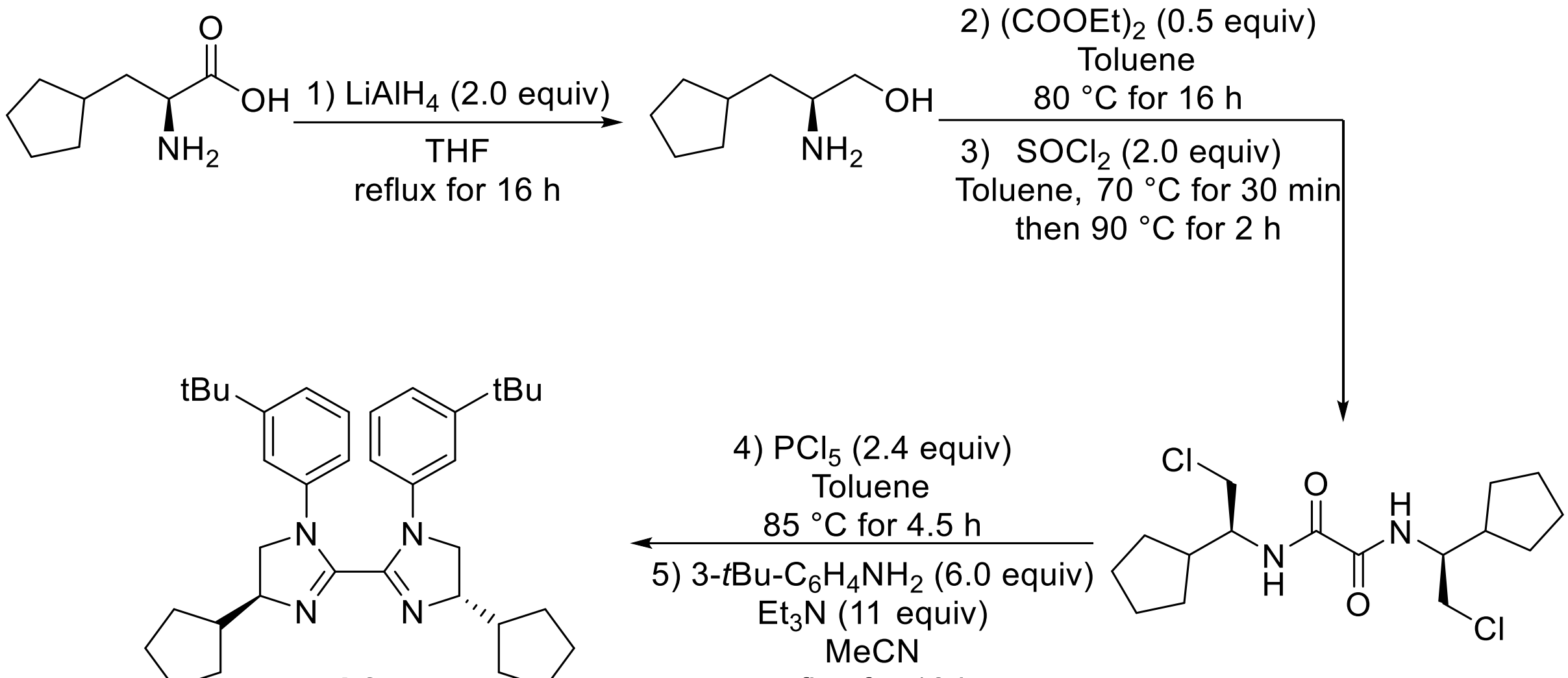

Step one: To a nitrogen flushed round bottom flask charged with $LiAlH_4$ (377 mg, 9.9 mmol, 2.0 equiv), anhydrous THF (50 mL) was added, and the suspension was cooled down to 0 °C with an ice bath. (*S*)-2-amino-3-cyclopentylpropanoic acid (716 mg, 5.0 mmol, 1.0 equiv) was added portion-wise over 5 min. After stirring at 0 °C for 20 min, the mixture was refluxed for 16 h, cooled down to 0 °C and quenched via Fieser work-up. After stirring for 20 min at RT, $MgSO_4$ was added and after a further 15 min, the mixture was filtered over celite. After concentration under reduced pressure, the amino alcohol was obtained as a very clean white solid that was used without further purification (557 mg, 4.3 mmol, 85% yield).

Step two: In a round bottom flask mounted by a reflux condenser, the amino alcohol (539 mg, 4.2 mmol, 2.0 equiv) was suspended in anhydrous toluene (42 mL) and diethyl oxalate (0.28 mL, 2.1 mmol, 1.0 equiv) was added. The reaction mixture was heated to 80 °C in an oil bath and stirred for 16 h, with the diamide precipitating as a white solid. After cooling down to RT, it was concentrated under reduced pressure to give the crude diol as a white powder.

Step three: The crude diol was suspended in toluene (22 mL) and thionyl chloride (0.30 mL, 4.1 mmol, 2.0 equiv) was added (the compound dissolved readily as thionyl chloride was added) The mixture was heated to 70 °C for 30 min, and then 90 °C for 2 h. After cooling down to RT, it was poured into a 20% aq. KOH solution in an ice bath, and the two layers were extracted with chloroform (×3). The combined organic layers were then washed with 20% aq. KOH solution, sat. Aq. $NaHCO_3$, brine, dried over Na_2SO_4, filtered and concentrated under reduced pressure to afford the crude dichloro intermediate as a beige solid (651 mg, 1.9 mmol, 90% yield).

Step four: PCl_5 (951 mg, 4.6 mmol, 2.4 equiv) was added to the flask containing the crude dichloride and it was nitrogen flushed. Toluene (14 mL) was added, and the mixture was stirred at 85 °C in the oil bath for 4.5 h. After cooling down, it was concentrated under reduced pressure to give a yellowish oily crude.

Step five: The crude was nitrogen flushed, and anhydrous MeCN (19 mL) was added, followed by triethylamine (2.9 mL, 20.8 mmol, 11 equiv) and 3-*tert*-butylaniline (1.65 g, 11 mmol, 5.9 equiv). The mixture was refluxed in an oil bath for 18 h. After cooling down, excess water was added and it was extracted with chloroform (×3), and the combined organic layers were washed with brine, dried with Na_2SO_4, filtered and concentrated under reduced pressure to give a dark brown oily crude. The product was isolated by flash column chromatography on silica gel. First the impurities (aniline and others) were quickly washed with *n*-pentane/AcOEt 1:1, until there was no more color to be seen on the column and no more UV active species on TLC, then the eluent was changed to pure AcOEt, affording ligand **L2** as a beige solid (308 mg, 0.57 mmol, 23% yield over 5 steps). In some cases, we observed the ligand coming with a dark color. If desired it can be recrystallized from hot *n*-hexane/AcOEt and obtained as a white solid. Control experiments showed that this dark impurity did not compromise the outcome of the photoredox catalysis.

^{1}H NMR (500 MHz, $CDCl_3$) δ 6.98 (t, *J* = 7.7 Hz, 2H), 6.95 (dt, *J* = 8.0, 1.5 Hz, 3H), 6.73 (t, *J* = 1.9 Hz, 2H), 6.50 – 6.42 (m, 2H), 4.03 (q, *J* = 9.7 Hz, 2H), 3.78 (t, *J* = 9.5 Hz, 2H), 3.49 (t, *J* = 9.3 Hz, 2H), 2.12 (sext, *J* = 8.0 Hz, 2H), 2.05 – 1.93 (m, 2H), 1.81 – 1.70 (m, 2H), 1.70 – 1.61 (m, 4H), 1.61 – 1.50 (m, 6H), 1.22 (s, 18H).

The spectroscopic data are in agreement with those reported in the literature.[S5]

5. General Procedure for the Synthesis of *α*-Chlorobenzyl- Silanes and Germanes (GP1)[S6]

In an oven-dried flask that was evacuated and nitrogen backfilled (×3), diisopropylamine (1.0 equiv) and anhydrous THF (1.0 M) were added and cooled down to –78 °C with an acetone/dry ice bath. *n*-Butyllithium (2.5 M in hexanes, 1.0 equiv) was added, and the mixture was allowed to warm up to RT after stirring for 5 min. In a separate oven-dried flask under nitrogen, to a 3:1 mixture of anhydrous THF and *n*-hexane (1.0 M), the benzylic chloride (1.0 equiv) and the chlorosilane or chlorogermane (1.0 equiv) were added and cooled down to –100 °C with a diethyl ether/liquid nitrogen bath. The LDA solution was added dropwise over 30 min, and the mixture was allowed to stir further 1 h before being warmed up to 0 °C with an ice bath. Water was added, and this was extracted with Et_2O (×3). The combined organic layers were washed with 1 M HCl, water, brine, dried over anhydrous Na_2SO_4, filtered and concentrated under reduced pressure to afford the crude product as a colorless oil. The product was isolated as indicated.

Note: the reaction of benzylic chlorides bearing electron-neutral or electron-donating substituents on the aryl ring is very slow and necessitates prolongated reaction times to ensure sufficient conversion of the starting material. We recommend stirring up to three hours for these compounds to ensure sufficient conversion.

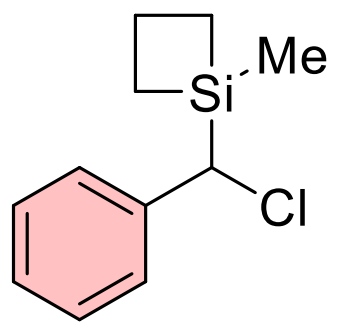

rac-**1a**
$C_{11}H_{15}ClSi$
M_W = 210.78

1-(Chloro(phenyl)methyl)-1-methylsiletane (*rac*-**1a**) was prepared according to **GP1** from 1-chloro-1-methylsiletane (1.22 mL, 10.0 mmol, 1.0 equiv) and benzyl chloride (1.15 mL, 10.0 mmol, 1.0 equiv). The product was purified by flash column chromatography on silica gel using *n*-pentane as eluent (R_f = 0.63, *n*-pentane, $KMnO_4$ stain) and obtained as a colorless oil (522 mg, 2.5 mmol, 25% yield).

^{1}H NMR (500 MHz, $CDCl_3$) δ 7.36 – 7.29 (m, 4H), 7.25 – 7.19 (m, 1H), 4.55 (s, 1H), 2.03 (dddd, *J* = 16.3, 12.3, 8.3, 5.1 Hz, 1H), 1.90 (dtt, *J* = 13.2, 10.3, 6.9 Hz, 1H), 1.22 (ddd, *J* = 17.1, 12.8, 6.8 Hz, 2H), 1.14 – 0.98 (m, 2H), 0.32 (s, 3H).

^{13}C NMR (126 MHz, $CDCl_3$) δ 139.5, 128.6, 126.94, 126.89, 51.7, 17.6, 13.8, 13.5, –3.4.

^{29}Si NMR (99 MHz, $CDCl_3$) δ 18.9.

HRMS (APCI) m/z: $[M]^+$ Calcd for $C_{11}H_{15}ClSi^+$ 210.0626; Found 210.0623.

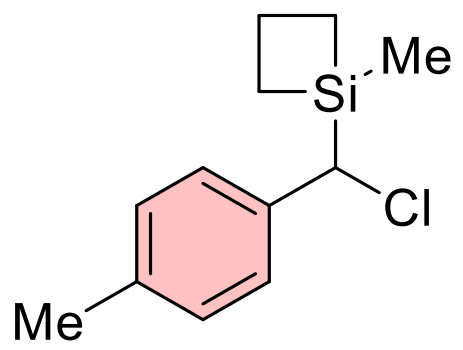

rac-**1b**
$C_{12}H_{17}ClSi$
M_W = 224.80

1-(Chloro(*p*-tolyl)methyl)-1-methylsiletane (*rac*-**1b**) was prepared according to **GP1** from 1-chloro-1-methylsiletane (0.61 mL, 5.0 mmol, 1.0 equiv) and 4-methylbenzyl chloride (0.66 mL, 5.0 mmol, 1.0 equiv). The product was purified by flash column chromatography on silica gel using *n*-pentane as eluent (R_f = 0.63, *n*-pentane, UV-active, $KMnO_4$ stain) and obtained as a colorless oil (522 mg, 2.3 mmol, 47% yield).

^{1}H NMR (500 MHz, $CDCl_3$) δ 7.20 (d, *J* = 8.0 Hz, 2H), 7.13 (d, *J* = 7.9 Hz, 2H), 4.51 (s, 1H), 2.33 (s, 3H), 2.10 – 1.97 (m, 1H), 1.97 – 1.84 (m, 1H), 1.29 – 1.15 (m, 2H), 1.13 – 0.99 (m, 2H), 0.31 (s, 3H).

^{13}C NMR (101 MHz, $CDCl_3$) δ 136.7, 136.5, 129.3, 126.9, 51.7, 21.2, 17.6, 13.7, 13.5, –3.4.

^{29}Si NMR (99 MHz, $CDCl_3$) δ 18.9.

HRMS (APCI) m/z: $[M]^+$ Calcd for $C_{12}H_{17}ClSi^+$ 224.0783; Found 224.0777.

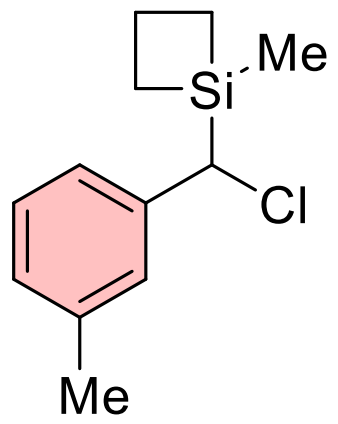

rac-**1c**
$C_{12}H_{17}ClSi$
M_W = 224.80

1-(Chloro(*m*-tolyl)methyl)-1-methylsiletane (*rac*-**1c**) was prepared according to **GP1** from 1-chloro-1-methylsiletane (0.61 mL, 5.0 mmol, 1.0 equiv) and 3-methylbenzyl chloride (0.66 mL, 5.0 mmol, 1.0 equiv). The product was purified by gravity column chromatography on silica gel using *n*-pentane as eluent (R_f = 0.54, *n*-pentane, UV-active, Seebach stain) and obtained as a colorless oil (278 mg, 1.2 mmol, 25% yield).

^{1}H NMR (500 MHz, $CDCl_3$) δ 7.21 (t, *J* = 7.5 Hz, 1H), 7.14 – 7.08 (m, 2H), 7.04 (d, *J* = 7.4 Hz, 1H), 4.51 (s, 1H), 2.35 (s, 3H), 2.10 – 1.97 (m, 1H), 1.97 – 1.85 (m, 1H), 1.29 – 1.15 (m, 2H), 1.13 – 0.99 (m, 2H), 0.31 (s, 3H).

^{13}C NMR (101 MHz, $CDCl_3$) δ 139.4, 138.2, 128.4, 127.8, 127.6, 124.0, 51.8, 21.6, 17.6, 13.8, 13.6, –3.4.

^{29}Si NMR (99 MHz, $CDCl_3$) δ 18.9.

HRMS (APCI) m/z: $[M]^+$ Calcd for $C_{12}H_{17}ClSi^+$ 224.0783; Found 224.0779.

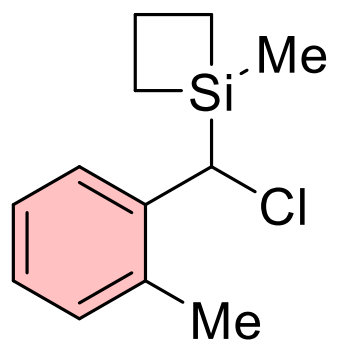

rac-**1d**
$C_{12}H_{17}ClSi$
M_W = 224.80

1-(Chloro(*o*-tolyl)methyl)-1-methylsiletane (*rac*-**1d**) was prepared according to **GP1** from 1-chloro-1-methylsiletane (0.61 mL, 5.0 mmol, 1.0 equiv) and 2-methylbenzyl chloride (0.66 mL, 5.0 mmol, 1.0 equiv). The product was purified by gravity column chromatography on silica gel using *n*-pentane as eluent (R_f = 0.54, *n*-pentane, UV-active, Seebach stain) and obtained as a colorless oil (547 mg, 2.4 mmol, 49% yield).

^{1}H NMR (500 MHz, $CDCl_3$) δ 7.46 (d, *J* = 7.7 Hz, 1H), 7.24 – 7.19 (m, 1H), 7.17 – 7.09 (m, 2H), 4.80 (s, 1H), 2.32 (s, 3H), 2.07 – 1.96 (m, 1H), 1.96 – 1.84 (m, 1H), 1.31 – 1.20 (m, 1H), 1.20 – 1.11 (m, 1H), 1.11 – 0.99 (m, 2H), 0.37 (s, 3H).

^{13}C NMR (126 MHz, $CDCl_3$) δ 138.0, 134.5, 130.4, 128.0, 126.9, 126.6, 48.1, 20.1, 17.8, 14.1, 13.6, –3.0.

^{29}Si NMR (99 MHz, $CDCl_3$) δ 18.9.

HRMS (APCI) m/z: $[M]^+$ Calcd for $C_{12}H_{17}ClSi^+$ 224.0783; Found 224.0778.

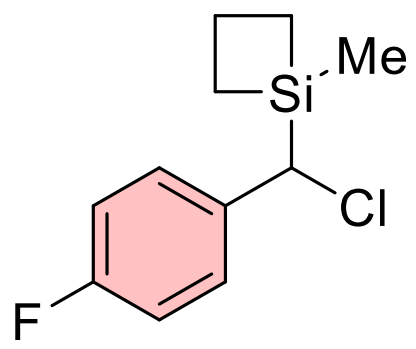

rac-**1e**
$C_{11}H_{14}ClFSi$
M_W = 228.77

1-(Chloro(4-fluorophenyl)methyl)-1-methylsiletane (*rac*-**1e**) was prepared according to **GP1** from 1-chloro-1-methylsiletane (0.25 mL, 2.0 mmol, 1.0 equiv) and 4-fluorobenzyl chloride (0.24 mL, 2.0 mmol, 1.0 equiv). The product was purified by flash column chromatography on silica gel using *n*-pentane as eluent (R_f = 0.57, *n*-pentane, UV-active, $KMnO_4$ stain) and obtained as a colorless oil (113 mg, 0.49 mmol, 25% yield).

^{1}H NMR (400 MHz, $CDCl_3$) δ 7.32 – 7.26 (m, 2H), 7.07 – 6.97 (m, 2H), 4.52 (s, 1H), 2.10 – 1.97 (m, 1H), 1.96 – 1.82 (m, 1H), 1.29 – 1.14 (m, 2H), 1.14 – 1.00 (m, 2H), 0.32 (s, 3H).

^{13}C NMR (101 MHz, $CDCl_3$) δ 161.83 (d, *J* = 245.6 Hz), 135.3 (d, *J* = 3.1 Hz), 128.5 (d, *J* = 8.0 Hz), 115.5 (d, *J* = 21.6 Hz), 50.9, 17.6, 13.6, 13.4, –3.4.

^{29}Si NMR (99 MHz, $CDCl_3$) δ 18.9.

^{19}F NMR (471 MHz, $CDCl_3$) δ –115.75 (tt, *J* = 8.6, 5.3 Hz).

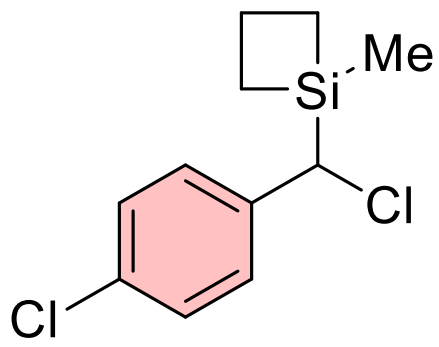

rac-**1f**
$C_{11}H_{14}Cl_2Si$
M_W = 245.22

1-(Chloro(4-chlorophenyl)methyl)-1-methylsiletane (*rac*-**1f**) was prepared according to **GP1** from 1-chloro-1-methylsiletane (0.61 mL, 5.0 mmol, 1.0 equiv) and 4-chlorobenzyl chloride (805 mg, 5.0 mmol, 1.0 equiv). The product was purified by gravity column chromatography on silica gel using *n*-pentane as eluent (R_f = 0.54, *n*-pentane, UV-active, Seebach stain) and obtained as a colorless oil (325 mg, 1.3 mmol, 27% yield).

^{1}H NMR (500 MHz, $CDCl_3$) δ 7.33 – 7.27 (m, 2H), 7.25 (d, *J* = 8.6 Hz, 2H), 4.51 (s, 1H), 2.10 – 1.96 (m, 1H), 1.94 – 1.80 (m, 1H), 1.28 – 1.13 (m, 2H), 1.13 – 1.00 (m, 2H), 0.31 (s, 3H).

^{13}C NMR (101 MHz, $CDCl_3$) δ 138.2, 132.6, 128.7, 128.1, 50.9, 17.6, 13.7, 13.4, –3.5.

^{29}Si NMR (99 MHz, $CDCl_3$) δ 18.9.

HRMS (APCI) m/z: $[M+H]^+$ Calcd for $C_{11}H_{15}Cl_2Si^+$ 245.0315; Found 245.0315.

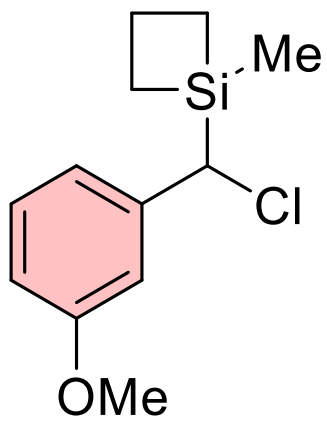

rac-**1g**
$C_{12}H_{17}ClOSi$
M_W = 240.80

1-(Chloro(3-methoxyphenyl)methyl)-1-methylsiletane (*rac*-**1g**) was prepared according to **GP1** from 1-chloro-1-methylsiletane (0.61 mL, 5.0 mmol, 1.0 equiv) and 3-methoxybenzyl chloride (0.73 mL, 5.0 mmol, 1.0 equiv). The product was purified by flash column chromatography on silica gel using cyclohexane/Et_2O 100:1 as eluent (R_f = 0.41, *n*-pentane/Et_2O 40:1) and obtained as a colorless oil (640 mg, 2.7 mmol, 53% yield).

^{1}H NMR (500 MHz, $CDCl_3$) δ 7.25 – 7.19 (m, 1H), 6.93 – 6.84 (m, 2H), 6.77 (ddd, *J* = 8.3, 2.4, 1.0 Hz, 1H), 4.52 (s, 1H), 3.81 (s, 3H), 2.10 – 1.98 (m, 1H), 1.98 – 1.87 (m, 1H), 1.28 – 1.16 (m, 2H), 1.13 – 1.01 (m, 2H), 0.32 (s, 3H).

^{13}C NMR (126 MHz, $CDCl_3$) δ 159.9, 141.2, 129.5, 119.3, 112.7, 112.4, 55.4, 51.7, 17.6, 13.9, 13.7, –3.4.

^{29}Si NMR (99 MHz, $CDCl_3$) δ 18.9.

HRMS (APCI) m/z: $[M+H]^+$ Calcd for $C_{12}H_{18}ClOSi^+$ 241.0810; Found 241.0809.

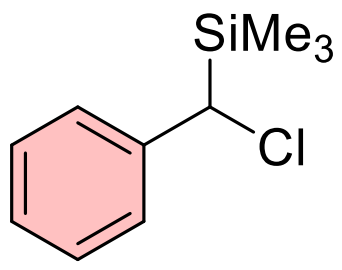

rac-**6a**
$C_{10}H_{15}ClSi$
M_W = 198.77

(Chloro(phenyl)methyl)trimethylsilane (*rac*-**6a**) was prepared according to **GP1** from chlorotrimethylsilane (1.27 mL, 10.0 mmol, 1.0 equiv) and benzyl chloride (1.15 mL, 10.0 mmol, 1.0 equiv). The product was purified by flash column chromatography on silica gel using *n*-pentane as eluent (R_f = 0.63, *n*-pentane, UV-active, $KMnO_4$ stain) and obtained as a colorless oil (1.265 g, 6.4 mmol, 64% yield).

^{1}H NMR (500 MHz, $CDCl_3$) δ 7.31 (t, *J* = 7.6 Hz, 2H), 7.28 – 7.24 (m, 2H), 7.21 (t, *J* = 7.2 Hz, 1H), 4.34 (s, 1H), 0.10 (s, 9H).

^{13}C NMR (101 MHz, $CDCl_3$) δ 140.3, 128.3, 127.0, 126.7, 53.1, –3.4.

^{29}Si NMR (99 MHz, $CDCl_3$) δ 6.0.

HRMS (APCI) m/z: $[M–Cl]^+$ Calcd for $C_{10}H_{15}Si^+$ 163.0938; Found 163.0935.

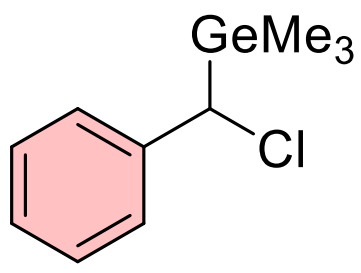

rac-**4a**
$C_{10}H_{15}ClGe$
M_W = 243.31

(Chloro(phenyl)methyl)trimethylgermane (*rac*-**4a**) was prepared according to **GP1** from trimethylgermanium chloride (0.65 mL, 5.0 mmol, 1.0 equiv) and benzyl chloride (0.58 mL, 5.0 mmol, 1.0 equiv). The product was purified by flash column chromatography on silica gel using *n*-pentane as eluent (R_f = 0.42, *n*-pentane) and obtained as a colorless oil (1.067 g, 4.4 mmol, 88% yield).

^{1}H NMR (500 MHz, $CDCl_3$) δ 7.30 (t, *J* = 7.6 Hz, 2H), 7.23 (d, *J* = 7.3 Hz, 2H), 7.19 (t, *J* = 7.3 Hz, 1H), 4.48 (s, 1H), 0.21 (s, 9H).

^{13}C NMR (126 MHz, $CDCl_3$) δ 141.39, 128.36, 126.51, 126.31, 53.00, –3.54.

HRMS (APCI) m/z: $[M–Cl]^+$ Calcd for $C_{10}H_{15}Ge^+$ 209.0380; Found 209.0378.

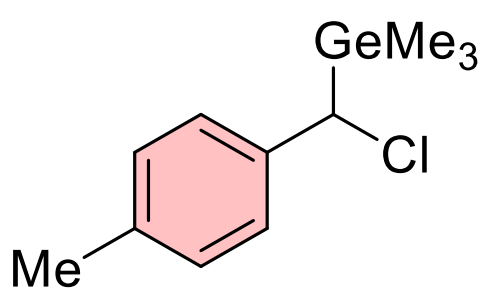

rac-**4b**
$C_{11}H_{17}ClGe$
M_W = 257.34

(Chloro(*p*-tolyl)methyl)trimethylgermane (*rac*-**4b**) was prepared according to **GP1** from trimethylgermanium chloride (0.37 mL, 3.0 mmol, 1.0 equiv) and 4-methylybenzyl chloride (0.40 mL, 3.0 mmol, 1.0 equiv). The product was purified by flash column chromatography on silica gel using *n*-pentane as eluent (R_f = 0.58, *n*-pentane) and obtained as a colorless oil (520 mg, 2.0 mmol, 67% yield).

^{1}H NMR (500 MHz, $CDCl_3$) δ 7.16 – 7.06 (m, 4H), 4.45 (s, 1H), 2.32 (s, 3H), 0.20 (s, 9H).

^{13}C NMR (101 MHz, $CDCl_3$) δ 138.3, 136.2, 129.1, 126.3, 53.0, 21.2, –3.5.

HRMS (APCI) m/z: $[M–Cl]^+$ Calcd for $C_{11}H_{17}Ge^+$ 223.0537; Found 223.0534.

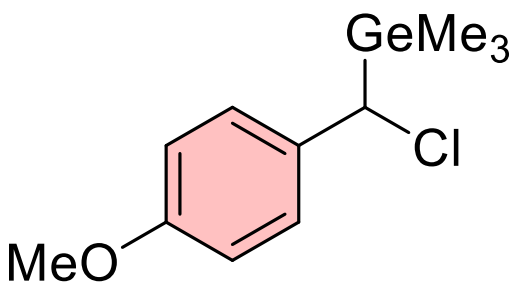

rac-**4c**
$C_{11}H_{17}ClGeO$
M_W = 273.34

(Chloro(4-methoxyphenyl)methyl)trimethylgermane (*rac*-**4c**) was prepared according to **GP1** from trimethylgermanium chloride (0.37 mL, 3.0 mmol, 1.0 equiv) and 4-methoxybenzyl chloride (0.68 mL, 5.0 mmol, 1.0 equiv). The crude product was first distilled by Kugelohr distillation (150 °C/10 mbar to remove part of the remaining starting material, then 200 °C) giving an enriched mixture of product and starting material. From this mixture the product started crystallizing. The liquid was removed with a pipette and the solid was triturated with cold *n*-pentane in the freezer before being filtered. The product was obtained as colorless crystals (110 mg, 0.4 mmol, 8% yield). *Note: purification of this compound via column chromatography on silica gel is not feasible due to immediate decomposition.*

^{1}H NMR (500 MHz, $CDCl_3$) δ 7.16 (d, *J* = 7.9 Hz, 2H), 6.84 (d, *J* = 8.0 Hz, 2H), 4.45 (s, 1H), 3.80 (s, 3H), 0.20 (s, 9H).

^{13}C NMR (126 MHz, $CDCl_3$) δ 158.4, 133.6, 127.7, 113.8, 55.4, 52.9, –3.5.

HRMS (APCI) m/z: $[M–Cl]^+$ Calcd for $C_{11}H_{17}GeO^+$ 239.0486; Found 239.0484.

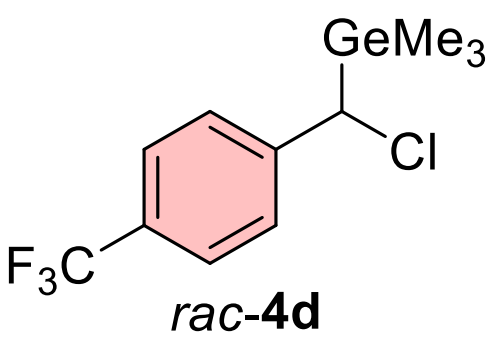

rac-**4d**
$C_{11}H_{14}ClF_3Ge$
M_W = 311.31

(Chloro(4-(trifluoromethyl)phenyl)methyl)trimethylgermane (*rac*-**4d**) was prepared according to **GP1** from trimethylgermanium chloride (0.37 mL, 3.0 mmol, 1.0 equiv) and 4-trifluoromethyl benzyl chloride (0.44 mL, 3.0 mmol, 1.0 equiv). The product was purified by flash column chromatography on silica gel using *n*-pentane as eluent (R_f = 0.42, *n*-pentane, UV-active, Seebach stain) and obtained as a colorless oil (763 mg, 2.5 mmol, 82% yield).

^{1}H NMR (400 MHz, $CDCl_3$) δ 7.56 (d, *J* = 8.2 Hz, 2H), 7.33 (d, *J* = 8.1 Hz, 2H), 4.52 (s, 1H), 0.22 (s, 9H).

^{13}C NMR (101 MHz, $CDCl_3$) Due to strong C–F coupling not all peaks of the quartets of the CF_3 ipso carbons were observed. δ 145.6, 128.6 (q, *J* = 33 Hz), 126.3, 125.35 (q, *J* = 4.0 Hz), 124.6 (q, *J* = 335 Hz), 52.1, –3.6.

^{19}F NMR (471 MHz, $CDCl_3$) δ –62.34.

HRMS (APCI) m/z: $[M–Cl]^+$ Calcd for $C_{11}H_{14}F_3Ge^+$ 277.0254; Found 277.0252.

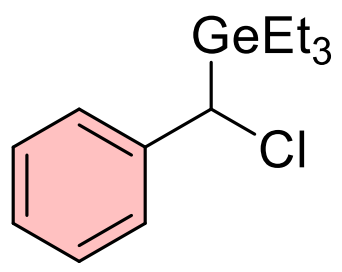

rac-**8a**
$C_{13}H_{21}ClGe$
M_W = 285.39

(Chloro(phenyl)methyl)triethylgermane (*rac*-**8a**) was prepared according to **GP1** from triethylgermanium chloride (0.83 mL, 5.0 mmol, 1.0 equiv) and benzyl chloride (0.58 mL, 5.0 mmol, 1.0 equiv). The crude product was obtained spectroscopically pure and used without further purification (1.37 g, 4.8 mmol, 96% yield).

^{1}H NMR (500 MHz, $CDCl_3$) δ 7.32 – 7.27 (m, 2H), 7.26 – 7.23 (m, 1H, overlap with residual $CHCl_3$), 7.17 (t, *J* = 7.1 Hz, 1H), 4.60 (s, 1H), 1.00 (t, *J* = 7.8 Hz, 9H), 0.94 – 0.76 (m, 6H).

^{13}C NMR (126 MHz, $CDCl_3$) δ 142.0, 128.4, 126.4 (overlap of two peaks), 51.1, 8.9, 3.5.

HRMS (APCI) m/z: [M–Cl]$^+$ Calcd for $C_{13}H_{21}Ge^+$ 251.0850; Found 251.0851.

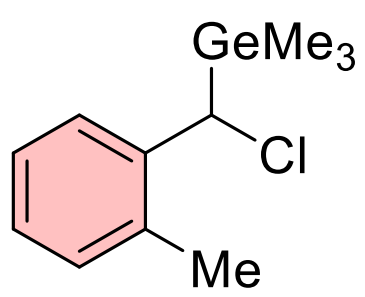

rac-**S4e**
$C_{11}H_{17}ClGe$
M_W = 257.34

(Chloro(*o*-tolyl)methyl)trimethylgermane (*rac*-**S4e**) was prepared according to **GP1** from trimethylgermanium chloride (0.35 mL, 2.8 mmol, 1.0 equiv) and 2-methylbenzyl chloride (0.37 mL, 2.8 mmol, 1.0 equiv). The product was purified by flash column chromatography on silica gel using *n*-pentane as eluent (R_f = 0.47) and obtained as a colorless oil (135 mg, 93.6 wt%, 0.52 mmol, 17% yield) as a mixture with the starting material benzylic chloride (7.4 wt%).

^{1}H NMR (500 MHz, $CDCl_3$) δ 7.40 (d, *J* = 7.9 Hz, 1H), 7.24 – 7.17 (m, 1H), 7.14 – 7.04 (m, 2H), 4.79 (s, 1H), 2.28 (s, 3H), 0.23 (s, 9H).

^{13}C NMR (101 MHz, $CDCl_3$) δ 139.8, 133.6, 130.2, 127.6, 126.5, 126.4, 49.1, 20.0, –3.1.

HRMS (APCI) m/z: [M–Cl]$^+$ Calcd for $C_{11}H_{17}Ge^+$ 223.0537; Found 223.0534.

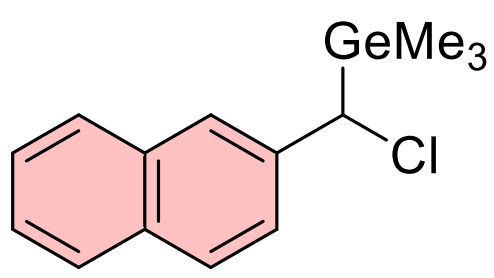

rac-**S4f**
$C_{14}H_{17}ClGe$
M_W = 293.37

(Chloro(naphthalen-2-yl)methyl)trimethylgermane (*rac*-**S4f**) was prepared according to **GP1** from trimethylgermanium chloride (0.37 mL, 3.0 mmol, 1.0 equiv) and 2-(chloromethyl)naphthalene (531 mg, 3.0 mmol, 1.0 equiv). The product was purified by flash column chromatography on silica gel using *n*-pentane as eluent (R_f = 0.34, *n*-pentane) and obtained as a colorless oil that quickly turned to a white solid (404 mg, 1.4 mmol, 46% yield).

^{1}H NMR (500 MHz, $CDCl_3$) δ 7.88 – 7.73 (m, 3H), 7.68 (s, 1H), 7.54 – 7.40 (m, 2H), 7.36 (d, *J* = 8.4 Hz, 1H), 4.66 (s, 1H), 0.24 (s, 9H).

^{13}C NMR (101 MHz, $CDCl_3$) δ 138.9, 133.4, 132.3, 128.1, 127.8, 127.8, 126.4, 125.7, 125.1, 124.4, 53.3, –3.4.

HRMS (APCI) m/z: [M–Cl]$^+$ Calcd for $C_{14}H_{17}Ge^+$ 259.0537; Found 259.0534.

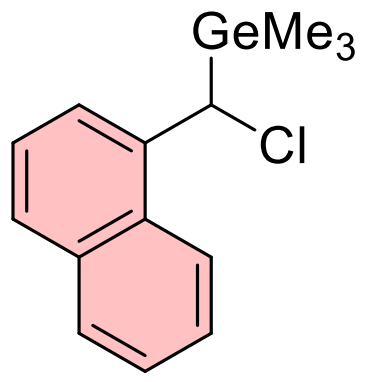

rac-**S4g**
$C_{14}H_{17}ClGe$
M_W = 293.37

(Chloro(naphthalen-1-yl)methyl)trimethylgermane (*rac*-**S4g**) was prepared according to **GP1** from trimethylgermanium chloride (0.37 mL, 3.0 mmol, 1.0 equiv) and 1-(chloromethyl)naphthalene (540 mg, 3.1 mmol, 1.0 equiv). The product was first filtered through silica gel with *n*-pentane/CH_2Cl_2 1:1 to get rid of insoluble impurities, and purified by flash column chromatography on silica gel using *n*-pentane as eluent (R_f = 0.38, UV-active, Seebach stain) and obtained as a viscous colorless oil (89 mg, 0.30 mmol, 10% yield).

^{1}H NMR (400 MHz, $CDCl_3$) δ 7.94 (d, *J* = 8.2 Hz, 1H), 7.86 (dd, *J* = 7.1, 2.4 Hz, 1H), 7.72 (d, *J* = 8.2 Hz, 1H), 7.64 (d, *J* = 7.2 Hz, 1H), 7.56 – 7.44 (m, 3H), 5.44 (s, 1H), 0.21 (s, 9H).

^{13}C NMR (101 MHz, $CDCl_3$) δ 137.3, 133.7, 130.2, 129.1, 127.2, 125.9, 125.7, 125.6, 125.1, 123.2, 49.3, –2.7.

HRMS (APCI) m/z: $[M–Cl]^+$ Calcd for $C_{14}H_{17}Ge^+$ 259.0537; Found 259.0533.

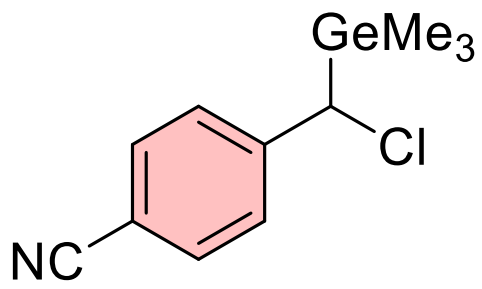

rac-**S4h**
$C_{11}H_{14}ClGeN$
M_W = 268.32

4-(Chloro(trimethylgermyl)methyl)benzonitrile (*rac*-**S4h**) was prepared according to **GP1** from trimethylgermanium chloride (0.25 mL, 2.0 mmol, 1.0 equiv) and 4-cyanobenzyl chloride (303 mg, 2.0 mmol, 1.0 equiv). The product was purified by flash column chromatography on silica gel using cyclohexane/Et_2O 50:1 as eluent (R_f = 0.27, *n*-pentane/Et_2O 20:1) and obtained as a colorless oil (434 mg, 1.6 mmol, 80% yield).

^{1}H NMR (500 MHz, $CDCl_3$) δ 7.60 (d, *J* = 8.4 Hz, 2H), 7.32 (d, *J* = 8.2 Hz, 2H), 4.50 (s, 1H), 0.22 (s, 9H).

^{13}C NMR (101 MHz, $CDCl_3$) δ 147.2, 132.2, 126.6, 119.0, 110.0, 52.1, –3.6.

HRMS (APCI) m/z: $[M+H]^+$ Calcd for $C_{11}H_{15}ClGeN^+$ 270.009; Found 270.0101.

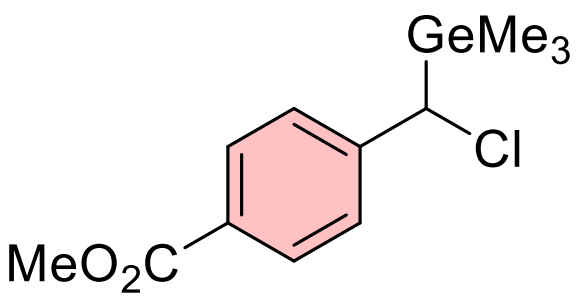

rac-**S4i**
$C_{12}H_{17}ClGeO_2$
M_W = 301.35

Methyl 4-(chloro(trimethylgermyl)methyl)benzoate (*rac*-**S4i**) was prepared according to **GP1** from trimethylgermanium chloride (0.25 mL, 2.0 mmol, 1.0 equiv) and methyl 4-(chloromethyl)benzoate (371 mg, 2.0 mmol, 1.0 equiv). The product was purified by flash column chromatography on silica gel using *n*-pentane/Et_2O 50:1 as eluent (R_f = 0.35, *n*-pentane/Et_2O 20:1) and obtained as a colorless oil that turned to a white solid after some hours in the freezer (449 mg, 1.5 mmol, 74% yield).

^{1}H NMR (500 MHz, $CDCl_3$) δ 7.98 (d, *J* = 8.3 Hz, 2H), 7.29 (d, *J* = 8.3 Hz, 2H), 4.52 (s, 1H), 3.91 (s, 3H), 0.21 (s, 9H).

^{13}C NMR (101 MHz, $CDCl_3$) δ 167.0, 146.8, 129.7, 128.2, 126.0, 52.4, 52.2, –3.6.

HRMS (APCI) m/z: $[M+H]^+$ Calcd for$C_{12}H_{18}ClGeO_2^+$ 303.0202; Found 303.0202.

6. Experimental Details for the Nickel-Catalyzed Cross-Electrophile Coupling

6.1 General Procedure for the Nickel-Catalyzed Cross-Electrophile Coupling (GP2)

In a nitrogen-filled glovebox, a medium-sized vial was charged with $NiBr_2 \cdot DME$ (6.2 mg, 20 μmol, 10 mol%) and the BiIm ligand (40 μmol, 20 mol%). THF (2 mL) was added, and the mixture was stirred for 20 to 30 min. In a GC vial, 4CzIPN (3.2 mg, 4.1 μmol, 2.0 mol%), the aryl bromide (0.20 mmol, 2.0 equiv, when solid) and the Hantzsch ester (67.6 mg, 0.30 mmol, 1.5 equiv) were combined and added to the catalyst mixture. In another GC vial containing the benzylic chloride (0.20 mmol, 1.0 equiv), THF (1 mL) was used to transfer the contents to the reaction vial, and this vial as well as the previous vial were rinsed with THF (2×0.5 mL) that was also added to the reaction mixture. Triethylamine (56 μL, 0.40 mmol, 2.0 equiv) was added, as well as the aryl bromide (0.20 mmol, 2.0 equiv, when liquid). The reaction vial was capped, removed from the glovebox, placed in the photoreactor and stirred for 18 h at RT. During this time, the reaction mixture turned from yellow to brown and a white precipitate formed. The reaction mixture was transferred to a separatory funnel containing 1M aq. HCl and diluted with Et_2O. The two layers were separated, and the aqueous layer was extracted with Et_2O (×2). Then the combined organic layers were washed with water, brine, dried over anhydrous Na_sSO_4, filtered and concentrated under reduced pressure at maximum 40 °C to limit protodesilylation or protodegermylation. The product was purified by flash column chromatography on silica gel using the indicated solvent mixture.

Racemic compounds were prepared according to the same procedure with the dtbbpy (4,4'-di-*tert*-butyl-2,2'-bipyridine) ligand.

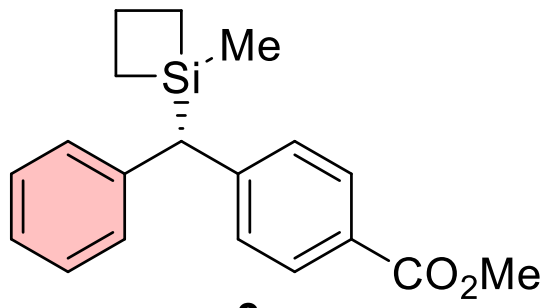

3aa
$C_{19}H_{22}O_2Si$
M_W = 310.47

Methyl (*S*)-4-((1-methylsiletan-1-yl)(phenyl)methyl)benzoate (**3aa**) was prepared according to **GP2** from 1-(chloro(phenyl)methyl)-1-methylsiletane (*rac*-**1a**) (42.6 mg, 0.20 mmol, 1.0 equiv) and methyl 4-bromobenzoate **2a** (21.1 mg, 0.10 mmol, 0.5 equiv). The product was purified by flash column chromatography on silica gel using *n*-pentane/Et_2O 100:1 as eluent (R_f = 0.34, *n*-pentane/Et_2O 20:1) and obtained as a white solid (39.4 mg, 0.13 mmol, 63%

yield). Crystals suitable for single crystal X-ray diffraction analysis were grown by slow evaporation of *n*-pentane from a nearly saturated solution.

^{1}H NMR (500 MHz, $CDCl_3$) δ 7.95 (d, *J* = 8.2 Hz, 2H), 7.33 – 7.26 (m, 4H), 7.24 – 7.17 (m, 3H), 3.89 (s, 3H), 3.85 (s, 1H), 1.97 (dtt, *J* = 12.8, 9.8, 7.1 Hz, 1H), 1.76 (dtt, *J* = 12.6, 10.0, 6.3 Hz, 1H), 1.18 – 0.98 (m, 4H), 0.22 (s, 3H).

^{13}C NMR (101 MHz, $CDCl_3$) δ 167.3, 148.1, 141.1, 129.9, 129.1, 128.8, 128.3, 127.3, 125.9, 52.1, 45.8, 18.0, 14.3, 14.2, –2.1.

^{29}Si NMR (99 MHz, $CDCl_3$) δ 20.0.

HRMS (APCI) m/z: $[M+H]^+$ Calcd for $C_{19}H_{23}O_2Si^+$ 311.1462; Found 311.1461.

Optical rotation: $[\alpha]_D^{20}$ = –28.1 (c 1.13, $CHCl_3$, >99% ee). The enantiomeric ratio of **3aa** was determined by HPLC analysis on a chiral stationary phase (*Daicel Chiralcel* OD-H column, column temperature 20 °C, solvent *n*-heptane:isopropanol = 99.9:0.1, flow rate 1.2 mL/min): t_R = 25.9 min (major), t_R = 33.2 min (minor). The minor enantiomer was not found in the HPLC trace of the enantioenriched sample for this compound.

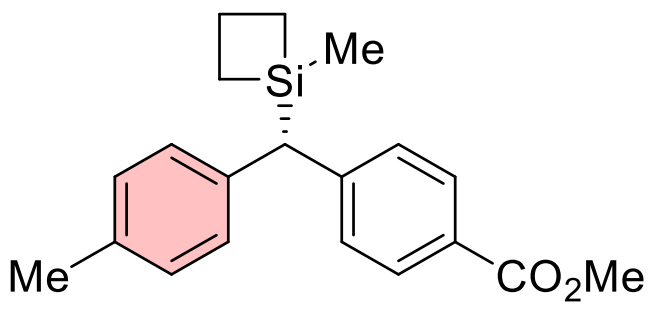

3ba
$C_{20}H_{24}O_2Si$
M_W = 324.50

Methyl (*S*)-4-((1-methylsiletan-1-yl)(*p*-tolyl)methyl)benzoate (**3ba**) was prepared according to **GP2** from 1-(chloro(*p*-tolyl)methyl)-1-methylsiletane (*rac*-**1b**) (45.0 mg, 0.20 mmol, 1.0 equiv) and methyl 4-bromobenzoate **2a** (43.1 mg, 0.20 mmol, 1.0 equiv). The product was purified by flash column chromatography on silica gel using *n*-pentane/Et_2O 100:1 as eluent (R_f = 0.33, *n*-pentane/Et_2O 20:1, UV-active, Seebach stain) and obtained as a white solid (34.3 mg, 0.11 mmol, 53% yield). Crystals suitable for single crystal X-ray diffraction analysis were grown by slow evaporation of *n*-pentane from a nearly saturated solution.

^{1}H NMR (500 MHz, $CDCl_3$) δ 7.93 (d, *J* = 8.2 Hz, 2H), 7.27 (s, 1H), 7.26 (s, 1H), 7.11 (s, 4H), 3.89 (s, 3H), 3.80 (s, 1H), 2.32 (s, 3H), 2.01 – 1.89 (m, 1H), 1.81 – 1.69 (m, 1H), 1.18 – 0.98 (m, 4H), 0.22 (s, 3H).

^{13}C NMR (126 MHz, $CDCl_3$) δ 167.3, 148.5, 137.9, 135.5, 129.9, 129.5, 129.1, 128.2, 127.2, 52.1, 45.3, 21.1, 18.0, 14.3, 14.2, –2.2.

^{29}Si NMR (99 MHz, $CDCl_3$) δ 20.0. 17.02.2026.

HRMS (APCI) m/z: $[M+H]^+$ Calcd for $C_{20}H_{25}O_2Si^+$ 325.1618; Found 325.1624.

Optical rotation: $[\alpha]_D^{20}$ = –43.3 (c 0.96, $CHCl_3$, 97% ee). The enantiomeric ratio of **3ba** was determined by HPLC analysis on a chiral stationary phase (*Daicel Chiralcel* OD-H column, column temperature 20 °C, solvent *n*-heptane:isopropanol = 100:0, flow rate 0.6 mL/min): t_R = 45.8 min (major), t_R = 51.2 min (minor).

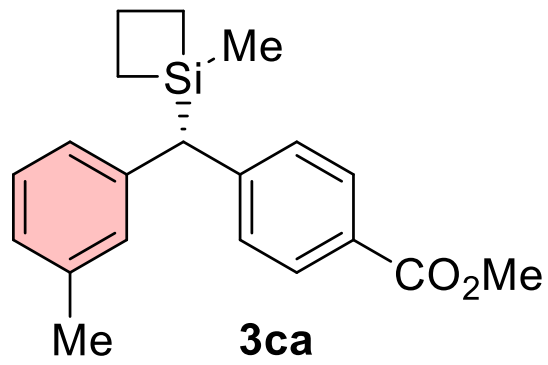

3ca

$C_{20}H_{24}O_2Si$
M_W = 324.50

Methyl (*R*)-4-((1-methylsiletan-1-yl)(*m*-tolyl)methyl)benzoate (**3ca**) was prepared according to **GP2** from 1-(chloro(*p*-tolyl)methyl)-1-methylsiletane (*rac*-**1c**) (44.4 mg, 0.20 mmol, 1.0 equiv) and methyl 4-bromobenzoate **2a** (43.0 mg, 0.20 mmol, 1.0 equiv). The product was purified by flash column chromatography on silica gel using *n*-pentane/Et_2O 100:1 as eluent (R_f = 0.32, *n*-pentane/Et_2O 20:1, UV-active, Seebach stain) and obtained as a colorless oil (33.4 mg, 0.10 mmol, 52% yield).

^{1}H NMR (500 MHz, $CDCl_3$) δ 7.94 (d, *J* = 8.3 Hz, 2H), 7.28 (d, *J* = 8.4 Hz, 2H), 7.20 (t, *J* = 7.9 Hz, 1H), 7.07 – 6.98 (m, 3H), 3.89 (s, 3H), 3.80 (s, 1H), 2.32 (s, 3H), 2.02 – 1.89 (m, 1H), 1.81 – 1.68 (m, 1H), 1.17 – 0.98 (m, 4H), 0.22 (s, 3H).

^{13}C NMR (126 MHz, $CDCl_3$) δ 167.3, 148.3, 141.0, 138.4, 130.0, 129.9, 128.6, 128.3, 127.2, 126.7, 126.1, 52.1, 45.7, 21.7, 18.0, 14.3, 14.3, –2.1.

^{29}Si NMR (99 MHz, $CDCl_3$) δ 20.0. 17.02.2026.

HRMS (APCI) m/z: $[M+H]^+$ Calcd for $C_{20}H_{25}O_2Si^+$ 325.1618; Found 325.1625.

Optical rotation: $[\alpha]_D^{20}$ = –30.2 (c 0.98, $CHCl_3$, 98% ee). The enantiomeric ratio of **3ca** was determined by HPLC analysis on a chiral stationary phase (*Daicel Chiralpak* AD-H column, column temperature 20 °C, solvent *n*-heptane:isopropanol = 99.9:0.1, flow rate 1.1 mL/min): t_R = 14.9 min (minor), t_R = 17.6 min (major).

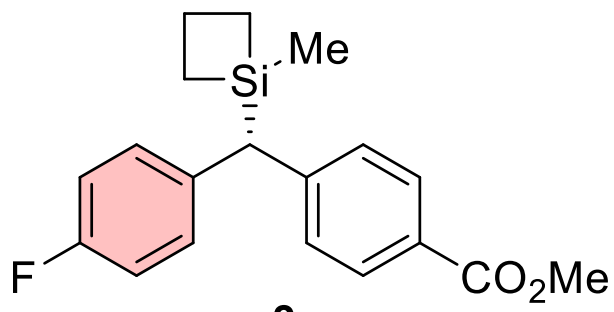

3ea

$C_{19}H_{21}FO_2Si$
M_W = 328.46

Methyl (*R*)-4-((4-fluorophenyl)(1-methylsiletan-1-yl)methyl)-benzoate (**3ea**) was prepared according to **GP2** from 1-(chloro(4-fluorophenyl)methyl)-1-methylsiletane (*rac*-**1e**) (45.0 mg, 0.20 mmol, 1.0 equiv) and methyl 4-bromobenzoate **2a** (43.0 mg, 0.20 mmol, 1.0 equiv). The product was purified by flash column chromatography on silica gel using *n*-pentane/Et_2O 100:1 as eluent (R_f = 0.34, *n*-pentane/Et_2O 20:1, UV-active, Seebach stain) and obtained as a white solid (29.0 mg, 0.09 mmol, 45% yield). Crystals suitable for single crystal X-ray diffraction analysis were grown by slow evaporation of *n*-pentane from a nearly saturated solution.

^{1}H NMR (500 MHz, $CDCl_3$) δ 7.95 (d, *J* = 8.3 Hz, 2H), 7.25 (d, *J* = 8.3 Hz, 2H), 7.22 – 7.15 (m, 2H), 7.05 – 6.95 (m, 2H), 3.90 (s, 3H), 3.82 (s, 1H), 2.03 – 1.90 (m, 1H), 1.80 – 1.67 (m, 1H), 1.16 – 0.99 (m, 4H), 0.22 (s, 3H).

^{13}C NMR (126 MHz, $CDCl_3$) δ 167.2, 161.32 (d, *J* = 244.1 Hz), 147.9, 136.81 (d, *J* = 2.9 Hz), 130.43 (d, *J* = 7.7 Hz), 130.0, 128.2, 127.4, 115.64 (d, *J* = 21.2 Hz), 52.1, 44.8, 18.0, 14.2, 14.1, –2.2.

^{19}F NMR (471 MHz, $CDCl_3$) δ –117.38 (tt, *J* = 8.7, 5.3 Hz).

^{29}Si NMR (99 MHz, $CDCl_3$) δ 20.0.

HRMS (APCI) m/z: $[M+H]^+$ Calcd for $C_{19}H_2FO_2Si^+$ 329.1378; Found 329.1373.

Optical rotation: $[\alpha]_D^{20}$ = –36.4 (c 0.96, $CHCl_3$, 98% ee). The enantiomeric ratio of **3ea** was determined by HPLC analysis on a chiral stationary phase (*Daicel Chiralpak* AD-H column, column temperature 20 °C, solvent *n*-heptane:isopropanol = 99.9:0.1, flow rate 1.1 mL/min): t_R = 26.6 min (minor), t_R = 33.3 min (major).

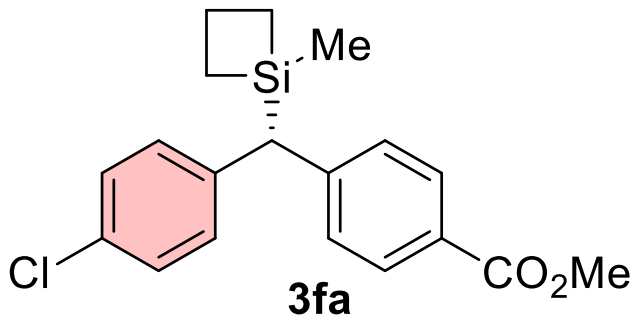

3fa
$C_{19}H_{21}ClO_2Si$
M_W = 344.91

Methyl (*R*)-4-((4-chlorophenyl)(1-methylsiletan-1-yl)methyl)-benzoate (**3fa**) was prepared according to **GP2** from 1-(chloro(4-chlorophenyl)methyl)-1-methylsiletane (*rac*-**1f**) (45.0 mg, 0.18 mmol, 1.0 equiv) and methyl 4-bromobenzoate **2a** (42.9 mg, 0.20 mmol, 1.1 equiv). The product was purified by flash column chromatography on silica gel using *n*-pentane/Et_2O 100:1 as eluent (R_f = 0.32, *n*-pentane/Et_2O 20:1, UV-active, Seebach stain) and obtained as a colorless oil (40.6 mg, 0.12 mmol, 64% yield).

^{1}H NMR (500 MHz, $CDCl_3$) δ 7.95 (d, *J* = 8.2 Hz, 2H), 7.27 (d, *J* = 8.4 Hz, 2H), 7.25 (d, *J* = 8.2 Hz, 2H), 7.15 (d, *J* = 8.5 Hz, 2H), 3.90 (s, 3H), 3.81 (s, 1H), 2.03 – 1.91 (m, 1H), 1.81 – 1.68 (m, 1H), 1.16 – 0.99 (m, 4H), 0.22 (s, 3H).

^{13}C NMR (126 MHz, $CDCl_3$) δ 167.2, 147.5, 139.7, 131.7, 130.3, 130.1, 128.9, 128.3, 127.6, 52.1, 45.1, 18.0, 14.2, 14.2, –2.2.

^{29}Si NMR (99 MHz, $CDCl_3$) δ 20.0.

HRMS (APCI) m/z: $[M+H]^+$ Calcd for $C_{20}H_{22}ClO_2Si^+$ 345.1072; Found 345.1076.

Optical rotation: $[\alpha]_D^{20}$ = –25.9 (c 0.85, $CHCl_3$, 97% ee). The enantiomeric ratio of **3fa** was determined by HPLC analysis on a chiral stationary phase (*Daicel Chiralpak* AD-H column, column temperature 20 °C, solvent *n*-heptane:isopropanol = 99.9:0.1, flow rate 1.1 mL/min): t_R = 30.4 min (minor), t_R = 49.4 min (major).

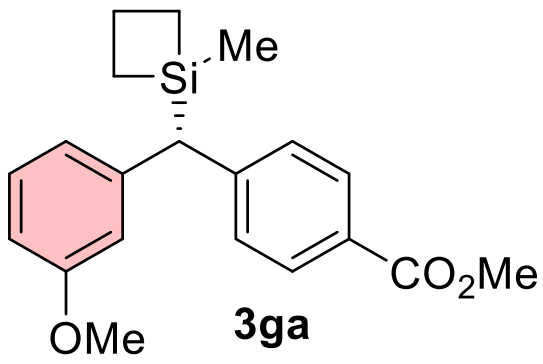

3ga
$C_{20}H_{24}O_3Si$
M_W = 340.49

Methyl (*R*)-4-((3-methoxyphenyl)(1-methylsiletan-1-yl)methyl)-benzoate (**3ga**) was prepared according to **GP2** from 1-(chloro(3-methoxyphenyl)methyl)-1-methylsiletane (*rac*-**1g**) (44.4 mg, 0.18 mmol, 1.0 equiv) and methyl 4-bromobenzoate **2a** (43.2 mg, 0.20 mmol, 1.1 equiv). The product was purified by flash column chromatography on silica gel using *n*-pentane/Et_2O 50:1 as eluent (R_f = 0.49, *n*-pentane/Et_2O 5:1, UV-active, Seebach stain) and obtained as a colorless oil (33.4 mg, 0.10 mmol, 53% yield).

^{1}H NMR (500 MHz, $CDCl_3$) δ 7.95 (d, *J* = 8.4 Hz, 2H), 7.29 (d, *J* = 8.2 Hz, 2H), 7.22 (t, *J* = 7.9 Hz, 1H), 6.83 (d, *J* = 7.6 Hz, 1H), 6.80 – 6.72 (m, 2H), 3.89 (s, 3H), 3.81 (s, 1H), 3.78 (s, 3H), 2.03 – 1.90 (m, 1H), 1.82 – 1.69 (m, 1H), 1.19 – 0.98 (m, 4H), 0.23 (s, 3H).

^{13}C NMR (126 MHz, $CDCl_3$) δ 167.3, 159.9, 147.9, 142.7, 129.9, 129.7, 128.4, 127.3, 121.6, 115.2, 110.9, 55.3, 52.1, 45.8, 18.0, 14.4, 14.3, –2.1.

^{29}Si NMR (99 MHz, $CDCl_3$) δ 19.6. 17.02.2026.

HRMS (APCI) m/z: $[M+H]^+$ Calcd for$C_{20}H_{25}O_3Si^+$ 341.1567; Found 341.1574.

Optical rotation: $[\alpha]_D^{20}$ = –22.9 (c 0.94, $CHCl_3$, 98% ee). The enantiomeric ratio of **3ga** was determined by HPLC analysis on a chiral stationary phase (*Daicel Chiralpak* AD-H column, column temperature 20 °C, solvent *n*-heptane:isopropanol = 98:2, flow rate 1.1 mL/min): t_R = 8.4 min (minor), t_R = 9.2 min (major).

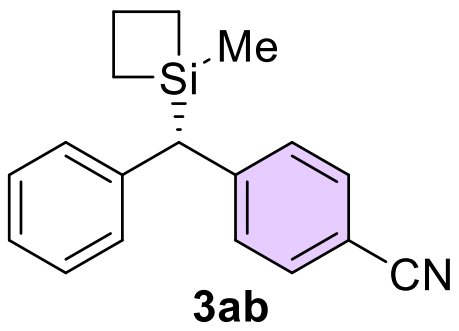

3ab
$C_{18}H_{19}NSi$
M_W = 277.44

(*S*)-4-((1-Methylsiletan-1-yl)(phenyl)methyl)benzonitrile (**3ab**) was prepared according to **GP2** from 1-(chloro(phenyl)methyl)-1-methylsiletane (*rac*-**1a**) (42.8 mg, 0.20 mmol, 1.0 equiv) and 4-bromobenzonitrile **2b** (31.1 mg, 0.17 mmol, 0.8 equiv). The product was purified by flash column chromatography on silica gel using *n*-pentane/Et_2O 100:1 as eluent (R_f = 0.33, *n*-pentane/Et_2O 20:1, UV-active, Seebach stain) and obtained as a colorless oil (18.4 mg, 0.07 mmol, 39% yield).

^{1}H NMR (500 MHz, $CDCl_3$) δ 7.56 (d, *J* = 8.3 Hz, 2H), 7.37 – 7.28 (m, 4H), 7.25 – 7.18 (m, 3H), 3.84 (s, 1H), 2.06 – 1.91 (m, 1H), 1.82 – 1.68 (m, 1H), 1.19 – 1.00 (m, 4H), 0.23 (s, 3H).

^{13}C NMR (126 MHz, $CDCl_3$) δ 148.5, 140.4, 132.4, 129.2, 129.0, 128.9, 126.3, 119.3, 109.0, 46.0, 18.0, 14.3, 14.2, –2.1.

^{29}Si NMR (99 MHz, $CDCl_3$) δ 20.0.

HRMS (APCI) m/z: $[M+H]^+$ Calcd for $C_{18}H_{20}NSi^+$ 278.1360; Found 278.1366.

Optical rotation: $[\alpha]_D^{20}$ = –50.6 (c 1.18, $CHCl_3$, 98% ee). The enantiomeric ratio of **3ab** was determined by HPLC analysis on a chiral stationary phase (*Daicel Chiralpak* AD-H

column, column temperature 20 °C, solvent *n*-heptane:isopropanol = 99.9:0.1, flow rate 1.1 mL/min): t_R = 31.3 min (minor), t_R = 33.1 min (major).

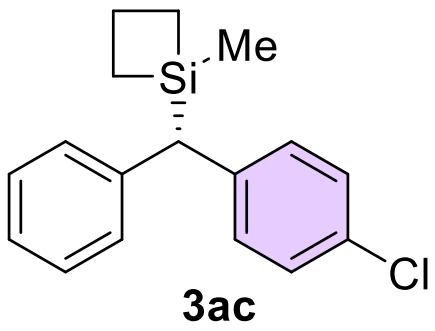

3ac
$C_{17}H_{19}ClSi$
M_W = 286.87

(*S*)-1-((4-Chlorophenyl)(phenyl)methyl)-1-methylsiletane (**3ac**) was prepared according to **GP2** from 1-(chloro(phenyl)methyl)-1-methylsiletane (*rac*-**1a**) (42.0 mg, 0.20 mmol, 1.0 equiv) and 1-bromo-4-chlorobenzene **2c** (38.3 mg, 0.20 mmol, 1.0 equiv). The product was purified by flash column chromatography on silica gel using *n*-pentane as eluent (R_f = 0.39, *n*-pentane, UV-active, Seebach stain) and obtained as a colorless oil (14.5 mg, 0.05 mmol, 25% yield).

^{1}H NMR (500 MHz, $CDCl_3$) δ 7.29 (t, *J* = 7.6 Hz, 2H), 7.26 – 7.22 (m, 2H), 7.21 – 7.17 (m, 2H), 7.15 (d, *J* = 8.5 Hz, 2H), 3.73 (s, 1H), 1.97 (dtt, *J* = 12.4, 9.7, 7.1 Hz, 1H), 1.77 (dtt, *J* = 12.5, 9.9, 6.2 Hz, 1H), 1.16 – 0.98 (m, 4H), 0.22 (s, 3H).

^{13}C NMR (126 MHz, $CDCl_3$) δ 148.5, 140.4, 132.4, 129.2, 129.0, 128.9, 126.3, 119.3, 109.0, 46.0, 18.0, 14.3, 14.2, –2.1.

^{29}Si NMR (99 MHz, $CDCl_3$) δ 20.0.

HRMS (APCI) m/z: $[M+H]^+$ Calcd for $C_{17}H_{20}ClSi^+$ 287.1017; Found 287.1013.

Optical rotation: $[\alpha]_D^{20}$ = –0.2 (c 0.64, $CHCl_3$). No baseline separation could be obtained by HPLC or GC analysis on a chiral stationary phase.

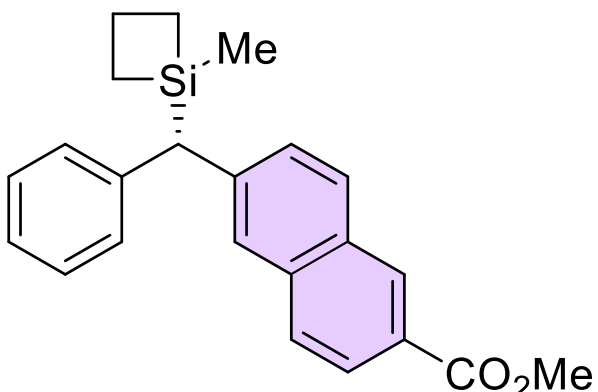

3ae
$C_{23}H_{24}O_2Si$
M_W = 360.53

Methyl (*S*)-6-((1-methylsiletan-1-yl)(phenyl)methyl)-2-naphthoate (**3ae**) was prepared according to **GP2** from 1-(chloro(phenyl)methyl)-1-methylsiletane (*rac*-**1a**) (42.0 mg, 0.20 mmol, 1.0 equiv) and methyl 6-bromo-2-naphthoate **2e** (53.0 mg, 0.20 mmol, 1.0 equiv). The product was purified by flash column chromatography on silica gel using *n*-*n*-pentane/Et_2O 100:1 as eluent (R_f = 0.21, *n*-pentane/Et_2O 20:1, UV-active) and obtained as a white solid (47.0 mg, 0.13 mmol, 65% yield).

^{1}H NMR (500 MHz, $CDCl_3$) δ 8.56 (s, 1H), 8.02 (dd, *J* = 8.6, 1.7 Hz, 1H), 7.87 (d, *J* = 8.5 Hz, 1H), 7.78 (d, *J* = 8.6 Hz, 1H), 7.68 (s, 1H), 7.44 (dd, *J* = 8.5, 1.8 Hz, 1H), 7.36 – 7.27 (m, 4H), 7.24 – 7.18 (m, 1H), 4.02 – 3.94 (m, 4H), 2.05 – 1.91 (m, 1H), 1.84 – 1.71 (m, 1H), 1.22 – 1.02 (m, 4H), 0.26 (s, 3H).

^{13}C NMR (126 MHz, $CDCl_3$) δ 167.5, 143.0, 141.5, 136.0, 130.9, 130.8, 129.5, 129.1, 128.8, 128.7, 127.8, 126.7, 126.1, 125.9, 125.5, 52.3, 45.8, 18.1, 14.43, 14.40, –2.1.

^{29}Si NMR (99 MHz, $CDCl_3$) δ 20.0.

HRMS (APCI) m/z: [M+H]$^+$ Calcd for $C_{23}H_{25}O_2Si^+$ 361.1618; Found 361.1628.

Optical rotation: $[\alpha]_D^{20}$ = –7.5 (c 0.95, $CHCl_3$, >99% ee). The enantiomeric ratio of **3ae** was determined by HPLC analysis on a chiral stationary phase (*Daicel Chiralcel* OD-H column, column temperature 20 °C, solvent *n*-heptane:isopropanol = 100:0, flow rate 0.6 mL/min): t_R = 110.2 min (minor), t_R = 120.9 min (major). The minor enantiomer was not found in the HPLC trace of the enantioenriched sample for this compound.

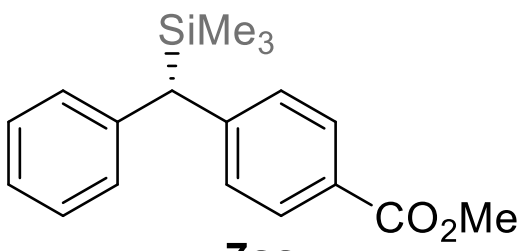

7aa
$C_{18}H_{22}O_2Si$
M_W = 298.46

Methyl (*S*)-4-(phenyl(trimethylsilyl)methyl)benzoate (**7aa**) was prepared according to **GP2** from (chloro(phenyl)methyl)trimethylsilane (*rac*-**6a**) (40.6 mg, 0.20 mmol, 1.0 equiv) and methyl 4-bromobenzoate **2a** (42.9 mg, 0.20 mmol, 1.1 equiv). The product was purified by flash column chromatography on silica gel using *n*-pentane/Et_2O 100:1 as eluent (R_f = 0.28, *n*-pentane/Et_2O 20:1, UV-active, Seebach stain) and obtained as a white solid (19.5 mg, 0.07 mmol, 33% yield).

^{1}H NMR (500 MHz, $CDCl_3$) δ 7.93 (d, *J* = 8.4 Hz, 2H), 7.28 (dd, *J* = 7.6, 4.7 Hz, 4H), 7.24 (d, *J* = 7.0 Hz, 2H), 7.17 (t, *J* = 7.2 Hz, 1H), 3.89 (s, 3H), 3.60 (s, 1H), 0.04 (s, 9H).

^{13}C NMR (101 MHz, $CDCl_3$) δ 167.3, 148.9, 142.0, 129.8, 129.1, 128.6, 128.5, 127.1, 125.6, 52.1, 46.7, –1.6.

^{29}Si NMR (99 MHz, $CDCl_3$) δ 3.8.

HRMS (APCI) m/z: [M+H]$^+$ Calcd for $C_{18}H_{23}O_2Si^+$ 299.1462; Found 299.1465.

Optical rotation: $[\alpha]_D^{20}$ = = –13.8 (c 0.62, $CHCl_3$). No baseline separation could be obtained by HPLC or GC analysis on a chiral stationary phase.

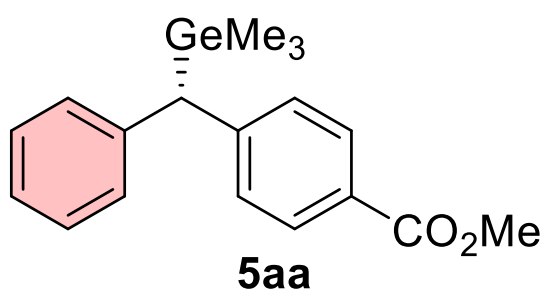

5aa
$C_{18}H_{22}GeO_2$
M_W = 343.00

Methyl (*S*)-4-(phenyl(trimethylgermyl)methyl)benzoate (**5aa**) was prepared according to **GP2** from (chloro(phenyl)methyl)trimethylgermane (*rac*-**4a**) (24.2 mg, 0.10 mmol, 1.0 equiv) and methyl 4-bromobenzoate **2a** (22.0 mg, 0.10 mmol, 1.0 equiv). The product was purified by flash column chromatography on silica gel using *n*-pentane/Et_2O 100:1 as eluent (R_f = 0.34, *n*-pentane/Et_2O 20:1) and obtained as a white solid (17.1 mg, 0.05 mmol, 50% yield).

^{1}H NMR (500 MHz, $CDCl_3$) δ 7.93 (d, *J* = 8.3 Hz, 2H), 7.29 (t, *J* = 7.6 Hz, 2H), 7.24 (d, *J* = 8.3 Hz, 2H), 7.21 (d, *J* = 7.2 Hz, 2H), 7.17 (t, *J* = 7.2 Hz, 1H), 3.89 (s, 3H), 3.79 (s, 1H), 0.15 (s, 9H).

^{13}C NMR (101 MHz, $CDCl_3$) δ 167.3, 149.3, 142.2, 129.8, 128.7, 128.6, 127.9, 126.9, 125.6, 52.1, 46.4, -1.9.

HRMS (APCI) m/z: $[M+H]^+$ Calcd for $C_{18}H_{23}GeO_2^+$ 345.0904; Found 345.0908.

Optical rotation: $[\alpha]_D^{20}$ = +0.5 (c 1.06, $CHCl_3$, 87% ee). The enantiomeric ratio of **5aa** was determined by HPLC analysis on a chiral stationary phase (*Daicel Chiralcel* OD-H column, column temperature 20 °C, solvent *n*-heptane:isopropanol = 99.9:0.1, flow rate 1.2 mL/min): t_R = 20.0 min (major), t_R = 26.2 min (minor).

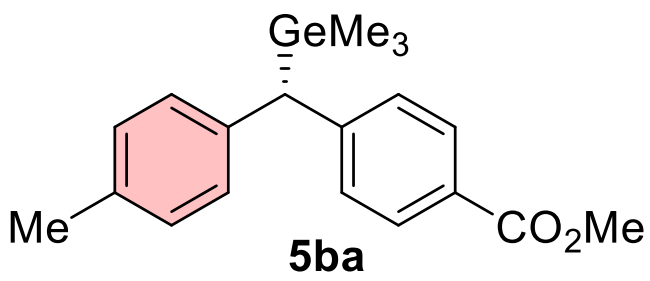

5ba
$C_{19}H_{24}GeO_2$
M_W = 357.03

Methyl (*S*)-4-(*p*-tolyl(trimethylgermyl)methyl)benzoate (**5ba**) was prepared according to **GP2** from (chloro(*p*-tolyl)methyl)triethylgermane (*rac*-**4b**) (51.2 mg, 0.20 mmol, 1.0 equiv) and methyl 4-bromobenzoate **2a** (43.0 mg, 0.20 mmol, 1.0 equiv). The product was purified by flash column chromatography on silica gel using *n*-pentane/Et_2O 50:1 as eluent (R_f = 0.23, *n*-pentane/Et_2O 20:1, UV-active, Seebach stain) and obtained as a white solid (25.7 mg, 0.07 mmol, 36% yield).

^1H NMR (500 MHz, $CDCl_3$) δ 7.92 (d, *J* = 8.4 Hz, 2H), 7.22 (d, *J* = 8.2 Hz, 2H), 7.10 (s, 4H), 3.88 (s, 3H), 3.75 (s, 1H), 2.32 (s, 3H), 0.14 (s, 9H).

^{13}C NMR (101 MHz, $CDCl_3$) δ 167.4, 149.7, 139.1, 135.1, 129.8, 129.3, 128.8, 127.8, 126.8, 77.5, 77.2, 76.8, 52.1, 45.9, 21.1, –1.9. 17.02.2026.

HRMS (APCI) m/z: $[M+H]^+$ Calcd for $C_{19}H_{25}GeO_2^+$ 359.1061; Found 359.1054.

Optical rotation: $[\alpha]_D^{20}$ = –7.0 (c 1.0, $CHCl_3$, 80% ee). The enantiomeric ratio of **5ba** was determined by HPLC analysis on a chiral stationary phase (*Daicel Chiralpak* AD-H column, column temperature 40 °C, solvent *n*-heptane:isopropanol = 99.9:0.1, flow rate 1.2 mL/min): t_R = 17.1 min (minor), t_R = 25.3 min (major).

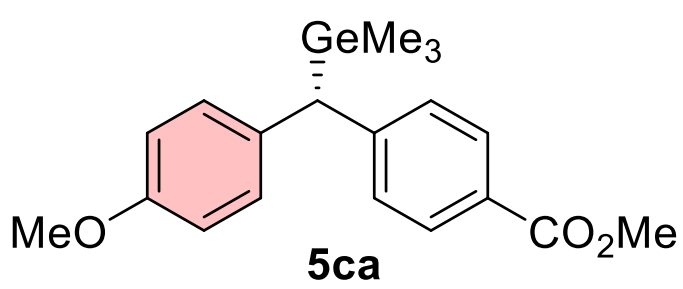

5ca
$C_{19}H_{24}GeO_3$
M_W = 373.03

Methyl (*R*)-4-((4-methoxyphenyl)(trimethylgermyl)methyl)-benzoate (**5ca**) was prepared according to **GP2** from (chloro(4-methoxyphenyl)methyl)trimethylgermane (*rac*-**4c**) (55.4 mg, 0.20 mmol, 1.0 equiv) and methyl 4-bromobenzoate **2a** (43.0 mg, 0.20 mmol, 1.0 equiv). The product was purified by flash column chromatography on silica gel using *n*-pentane/Et_2O 20:1 as eluent (R_f = 0.18, *n*-pentane/Et_2O 20:1, UV-active, Seebach stain) and obtained as a colorless oil (35.0 mg, 87.4 wt%, 0.09 mmol, 40% yield). This compound was obtained along with 9% yield (12.6 wt%) of the protodegermylation product.

^{1}H NMR (500 MHz, $CDCl_3$) δ 7.92 (d, *J* = 8.3 Hz, 2H), 7.20 (d, *J* = 8.3 Hz, 2H), 7.13 (d, *J* = 8.6 Hz, 2H), 6.84 (d, *J* = 8.7 Hz, 2H), 3.88 (s, 3H), 3.79 (s, 3H), 3.74 (s, 1H), 0.14 (s, 9H).

^{13}C NMR (101 MHz, $CDCl_3$) δ 167.4, 157.6, 149.9, 134.2, 129.9, 129.9, 129.8, 127.6, 114.0, 55.4, 52.1, 45.3, –1.9. 19.02.2026.

HRMS (APCI) m/z: $[M+H]^+$ Calcd for $C_{18}H_{25}GeO_2^+$ 375.1010; Found 375.1005.

Optical rotation: $[\alpha]_D^{20}$ = –5.6 (c 22.9, $CHCl_3$, 54% ee). The enantiomeric ratio of **5ca** was determined by HPLC analysis on a chiral stationary phase (*Daicel Chiralpak* AD-H column, column temperature 20 °C, solvent *n*-heptane:isopropanol = 98:2, flow rate 0.6 mL/min): t_R = 17.3 min (minor), t_R = 29.2 min (major).

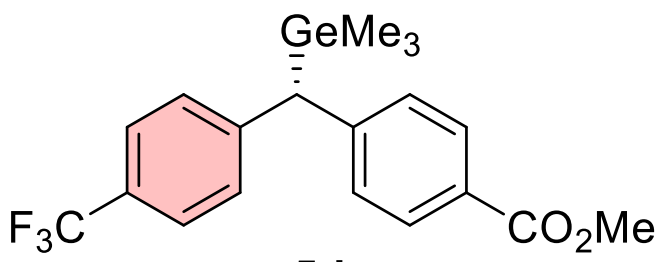

5da
$C_{19}H_{21}F_3GeO_2$
M_W = 411.00

Methyl (*R*)-4-((4-(trifluoromethyl)phenyl)(trimethylgermyl)-methyl)benzoate (**5da**) was prepared according to **GP2** from (chloro(4-(trifluoromethyl)phenyl)methyl)-triethylgermane (*rac*-**4d**) (62.6 mg, 0.20 mmol, 1.0 equiv) and methyl 4-bromobenzoate **2a** (43.0 mg, 0.20 mmol, 1.0 equiv). The product was purified by flash column chromatography on silica gel using *n*-pentane/Et_2O 150:1 as eluent (R_f = 0.23, *n*-pentane/Et_2O 20:1, UV-active, Seebach stain) and obtained as a white solid (23.8 mg, 0.06 mmol, 29% yield). Crystals suitable for single crystal X-ray diffraction analysis were grown by slow evaporation of *n*-pentane from a nearly saturated solution.

^{1}H NMR (500 MHz, $CDCl_3$) δ 7.96 (d, *J* = 8.1 Hz, 2H), 7.54 (d, *J* = 8.0 Hz, 2H), 7.30 (d, *J* = 7.9 Hz, 2H), 7.25 (d, *J* = 8.3 Hz, 2H), 3.90 (s, 3H), 3.85 (s, 1H), 0.16 (s, 9H).

^{13}C NMR (126 MHz, $CDCl_3$) Due to strong C–F coupling not all peaks of the quartets of the CF_3 ipso carbons were observed. δ 167.1, 148.0, 146.7, 130.0, 128.5, 128.2, 127.5, 125.57 (q, *J* = 3.5 Hz), 52.1, 46.4, –1.8.

^{19}F NMR (471 MHz, $CDCl_3$) δ –62.3.

HRMS (APCI) m/z: $[M+H]^+$ Calcd for $C_{19}H_{22}F_3GeO_2^+$ 413.0778; Found 413.0769.

Optical rotation: $[\alpha]_D^{20}$ = +2.2 (c 0.97, $CHCl_3$, 96% ee). The enantiomeric ratio of **5da** was determined by HPLC analysis on a chiral stationary phase (*Daicel Chiralpak* AD-H column, column temperature 40 °C, solvent *n*-heptane:isopropanol = 99.9:0.1, flow rate 1.2 mL/min): t_R = 21.9 min (minor), t_R = 28.2 min (major).

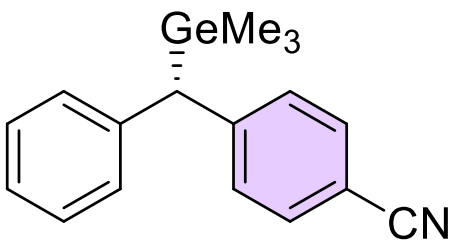

5ab
$C_{17}H_{19}GeN$
M_W = 309.98

(*S*)-4-(Phenyl(trimethylgermyl)methyl)benzonitrile (**5ab**) was prepared according to **GP2** from (chloro(phenyl)methyl)-trimethylgermane (*rac*-**4a**) (49.2 mg, 0.20 mmol, 1.0 equiv) and 4-bromobenzonitrile **2b** (36.4 mg, 0.20 mmol, 1.0 equiv). The product was purified by flash column chromatography on silica gel using *n*-pentane/Et_2O 100:1 as eluent (R_f = 27, *n*-pentane/Et_2O 20:1, UV-active, Seebach stain) and obtained as a colorless oil (21.9 mg, 0.07 mmol, 35% yield).

^{1}H NMR (500 MHz, $CDCl_3$) δ 7.54 (d, *J* = 8.4 Hz, 2H), 7.35 – 7.26 (m, 4H), 7.19 (t, *J* = 7.6 Hz, 3H), 3.78 (s, 1H), 0.16 (s, 9H).

^{13}C NMR (126 MHz, $CDCl_3$) δ 149.7, 141.5, 132.3, 128.9, 128.8, 128.4, 125.9, 119.4, 108.6, 46.6, –1.8.

HRMS (APCI) m/z: $[M+H]^+$ Calcd for $C_{17}H_{20}GeN^+$ 312.0802; Found 312.0808.

Optical rotation: $[\alpha]_D^{20}$ = –1.0 (c 0.66, $CHCl_3$, 88% ee). The enantiomeric ratio of **5ab** was determined by HPLC analysis on a chiral stationary phase (*Daicel Chiralpak* AD-H column, column temperature 20 °C, solvent *n*-heptane:isopropanol = 99.9:0.1, flow rate 1.1 mL/min): t_R = 24.2 min (major), t_R = 33.7 min (minor).

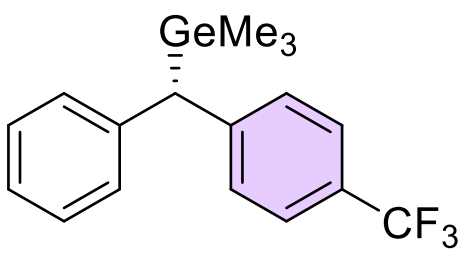

5ah
$C_{17}H_{19}F_3Ge$
M_W = 352.96

(*S*)-Trimethyl(phenyl(4-(trifluoromethyl)phenyl)methyl)-germane (**5ah**) was prepared according to **GP2** from (chloro(phenyl)methyl)trimethylgermane (*rac*-**4a**) (48.4 mg, 0.20 mmol, 1.0 equiv) and 1-bromo-4-(trifluoromethyl)benzene **2h** (36.4 mg, 0.16 mmol, 0.8 equiv). The product was purified by flash column chromatography on silica gel using *n*-pentane/Et_2O 100:1 as eluent (R_f = 0.52, *n*-pentane/Et_2O 20:1, UV-active, Seebach stain) and obtained as a colorless oil (23.5 mg, 0.07 mmol, 33% yield).

^{1}H NMR (500 MHz, $CDCl_3$) δ 7.51 (d, *J* = 8.1 Hz, 2H), 7.33 – 7.27 (m, 4H), 7.18 (m, 3H), 3.78 (s, 1H), 0.16 (s, 9H).

^{13}C NMR (101 MHz, $CDCl_3$) δ 147.8, 142.2, 128.70, 128.67, 128.2, 127.25 (q, *J* = 32.4 Hz), 125.6, 125.4 (q, *J* = 3.8 Hz), 124.6 (q, *J* = 272 Hz), 46.0, –1.9.

^{19}F NMR (471 MHz, $CDCl_3$) δ –62.18.

HRMS (APCI) m/z: $[M–H]^+$ Calcd for $C_{17}H_{18}F_3Ge^+$ 353.0567; Found 353.0562.

Optical rotation: $[\alpha]_D^{20}$ = –8.4 (c 0.25, $CHCl_3$, 91% ee). The enantiomeric ratio of **5ah** was determined by chiral GLC analysis (*Supelco Astec CHIRALDEX B-DM* column (30 m × 250 μm × 0.12 μm film thickness), column temperature 60 °C for 30 min, then 0.5 °C/min until 175 °C, flow rate 1.3 mL/min): t_R = 172.2 min (minor), t_R = 173.0 min (major).

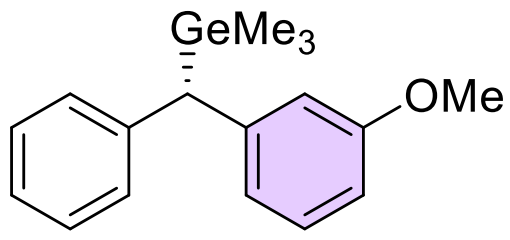

5ai
$C_{17}H_{22}GeO$
M_W = 314.99

(*S*)-((3-Methoxyphenyl)(phenyl)methyl)trimethylgermane (**5ai**) was prepared according to **GP2** from (chloro(phenyl)-methyl)trimethylgermane (*rac*-**4a**) (49.4 mg, 0.20 mmol, 1.0 equiv) and 1-bromo-3-methoxybenzene **2i** (25 µL, 0.20 mmol, 1.0 equiv). The product was purified by flash column chromatography on silica gel using *n*-pentane/Et_2O 150:1 as eluent (R_f = 0.56, *n*-pentane/Et_2O 20:1, UV-active, Seebach stain) and obtained as a colorless oil (12.9 mg, 0.04 mmol, 20% yield).

^{1}H NMR (400 MHz, $CDCl_3$) δ 7.29 – 7.23 (m, 1H, overlap with residual $CHCl_3$), 7.22 – 7.17 (m, 3H), 7.13 (tt, *J* = 6.5, 1.3 Hz, 1H), 6.79 (d, *J* = 7.7 Hz, 1H), 6.75 (t, *J* = 2.1 Hz, 1H), 6.68 (dd, *J* = 8.0, 2.2 Hz, 1H), 3.78 (s, 3H), 3.68 (s, 1H), 0.15 (s, 9H).

^{13}C NMR (101 MHz, $CDCl_3$) δ 159.7, 145.0, 143.1, 129.3, 128.5, 128.4, 125.2, 121.0, 114.5, 110.1, 55.2, 46.0, –1.8.

HRMS (APCI) m/z: $[M+H]^+$ Calcd for $C_{17}H_{23}GeO^+$ 317.0955; Found 317.0952.

Optical rotation: $[\alpha]_D^{20}$ = –0.7 (c 1.0, $CHCl_3$, 84% ee). The enantiomeric ratio of **5ai** was determined by HPLC analysis on a chiral stationary phase (*Daicel Chiralcel* AD-H column, column temperature 20 °C, solvent *n*-heptane:isopropanol = 100:0, flow rate 0.15 mL/min): t_R = 54.7 min (minor), t_R = 57.2 min (major).

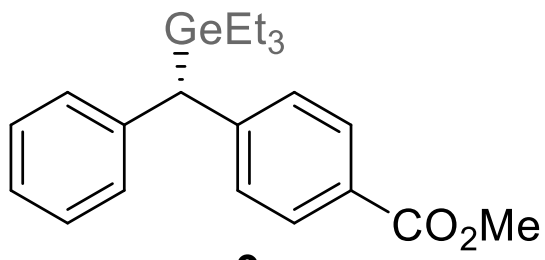

9aa
$C_{21}H_{28}GeO_2$
M_W = 385.08

Methyl (*S*)-4-(phenyl(triethylgermyl)methyl)benzoate (**9aa**) was prepared according to **GP2** from (chloro(phenyl)methyl)-triethylgermane (*rac*-**8a**) (56.9 mg, 0.20 mmol, 1.0 equiv) and methyl 4-bromobenzoate **2a** (43.0 mg, 0.20 mmol, 1.0 equiv). The product was purified by flash column chromatography on silica gel using *n*-pentane/Et_2O 150:1 as eluent (R_f = 42, *n*-pentane/Et_2O 20:1, UV-active + Seebach stain, UV-active, Seebach stain) and obtained as a colorless oil (14.6 mg, 0.04 mmol, 19% yield).

^{1}H NMR (500 MHz, $CDCl_3$) δ 7.92 (d, *J* = 8.2 Hz, 2H), 7.29 (d, *J* = 9.2 Hz, 3H), 7.23 (d, *J* = 7.2 Hz, 2H), 7.16 (t, *J* = 7.2 Hz, 1H), 3.92 (s, 1H), 3.89 (s, 3H), 0.91 (t, *J* = 7.7 Hz, 9H), 0.78 (q, *J* = 7.4 Hz, 6H).

^{13}C NMR (126 MHz, $CDCl_3$) δ 167.3, 149.6, 142.4, 129.8, 128.8, 128.6, 128.0, 126.8, 125.5, 52.0, 43.8, 9.0, 4.8.

HRMS (APCI) m/z: $[M+H]^+$ Calcd for $C_{21}H_{29}GeO_2^+$ 387.1374; Found 387.1383.

Optical rotation: $[\alpha]_D^{20}$ = –3.4 (c 1.3, $CHCl_3$, 84% ee). The enantiomeric ratio of **9aa** was determined by HPLC analysis on a chiral stationary phase (*Daicel Chiralpak* AD-H

column, column temperature 10 °C, solvent *n*-heptane:isopropanol = 99.9:0.1, flow rate 0.7 mL/min): t_R = 23.9 min (minor), t_R = 27.7 min (major).

6.2 Optimization of Reaction Conditions

Optimization reactions were carried out on a 0.10 mmol scale following the procedure of **GP2** with α-chlorobenzyl germane *rac*-**4a** and methyl 4-bromobenzoate.

General procedure for solvent screening:

Solvent screening was done according to **GP2** by replacing THF by the indicated solvent or solvent mixture. At this point of the research a proper purification method had not yet been found. Purification of the crude material by flash column chromatography on silica gel using *n*-pentane/AcOEt 50:1 or 100:1 afforded compound **5aa** as mixture with the aryl bromide and/or its byproducts. The reaction yields were determined by ^{1}H NMR analysis using dibromomethane as an internal standard.

General procedure for nickel precatalyst screening:

Nickel precatalyst screening was done according to **GP2** by replacing $NiCl_2$·DME by the indicated precatalyst. Product **5aa** was isolated by flash column chromatography on silica gel using *n*-pentane/Et_2O 100:1. The rotavapor bath temperature should not exceed 40 °C to minimize protodegermylation and the product should not be left there for exceedingly long times.

7. Control Experiments

7.1 Radical Trapping with TEMPO

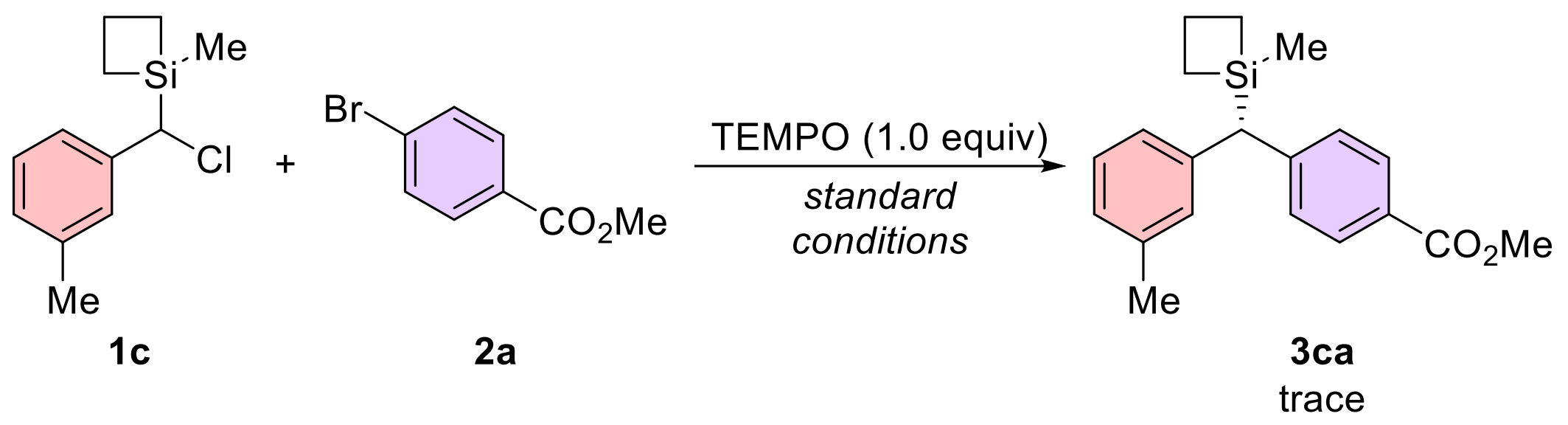

The radical trapping experiment was conducted according to **GP2** on a 0.10 mmol scale with substrates **1c** and **2a**, and by adding TEMPO (15.6 mg, 0.10 mmol, 1.0 equiv) to the reaction mixture. While substrate conversion was observed, the coupling product was only formed in trace amounts. HRMS analysis of the reaction mixture did not allow to detect any TEMPO-adduct.

7.2 Stern-Volmer Luminescence Quenching Experiment

The measurements were performed using a Duetta™ Fluorescence and Absorbance Spectrometer (HORIBA Scientific) with the following parameters: excitation wavelength at 450 nm, emission intensity was collected from 460–800 nm, with the maximum at 525 nm being used for Ster-Volmer analysis. Slit widths for excitation 5 nm, for emission 3 nm, integration time 0.5 s. All solutions were prepared using freshly distilled dry and degassed by nitrogen bubbling THF. The measurements were carried out using a 10 µM solution of 4CzIPN, 0.1 M of HEH, 1 M of Et_3N, 1 M of substrate **1c** and 1 M of aryl bromide **2a**. As the measurement setup was not equipped for measurements under inert conditions, the samples were eventually exposed to air. Measurements were recorded as fast as possible to limit quenching by oxygen. While this was not too problematic for the experiments with HEH and Et_3N, we cannot say for sure that the small quenching observed with **1c** and **2a** may not be due to oxygen entering into the system.

Figure S1. Luminescence quenching of 4CzIPN with HEH.

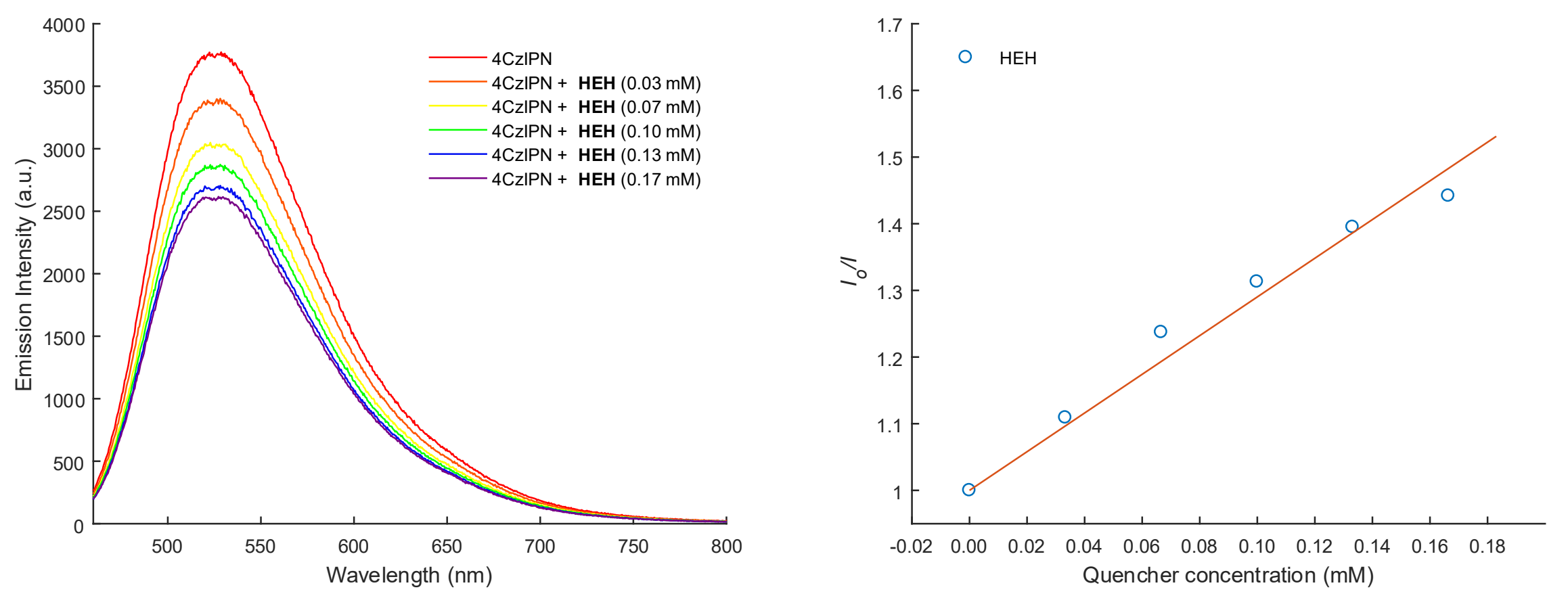

Figure S2. Luminescence quenching of 4CzIPN with Et_3N.

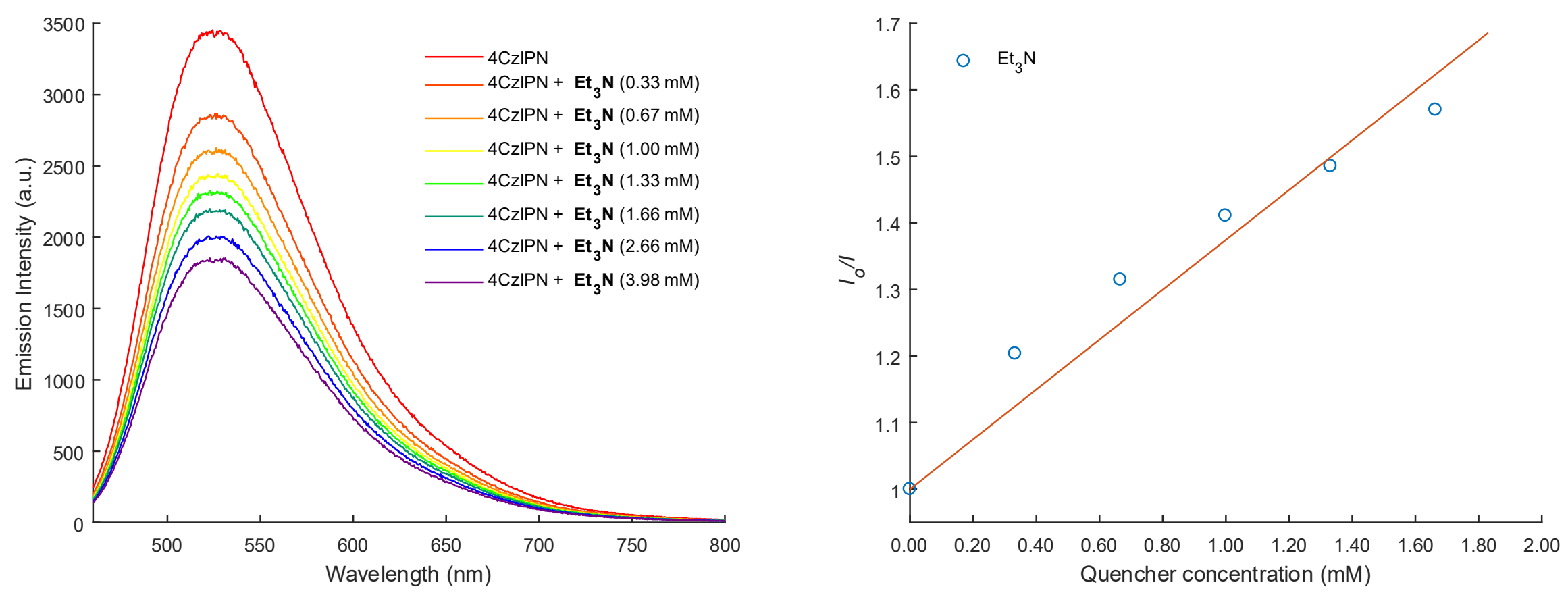

Figure S3. Luminescence quenching of 4CzIPN with **1c**.

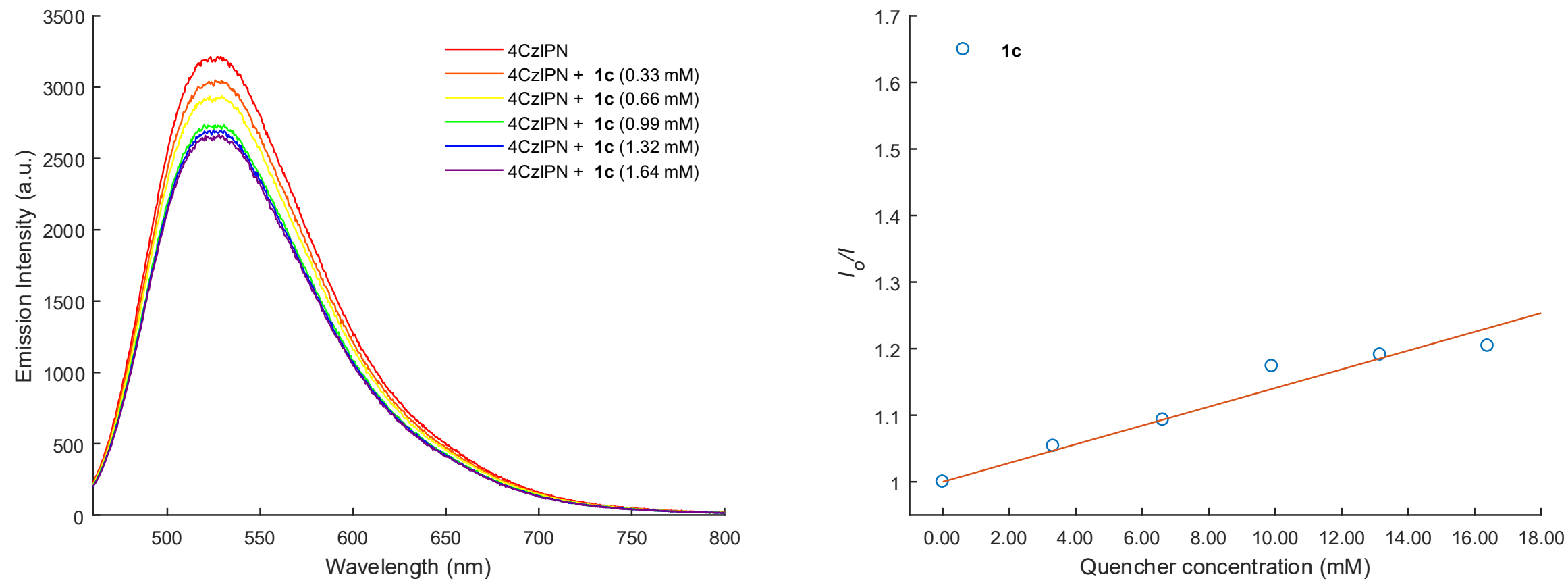

Figure S4. Luminescence quenching of 4CzIPN with **2a**.

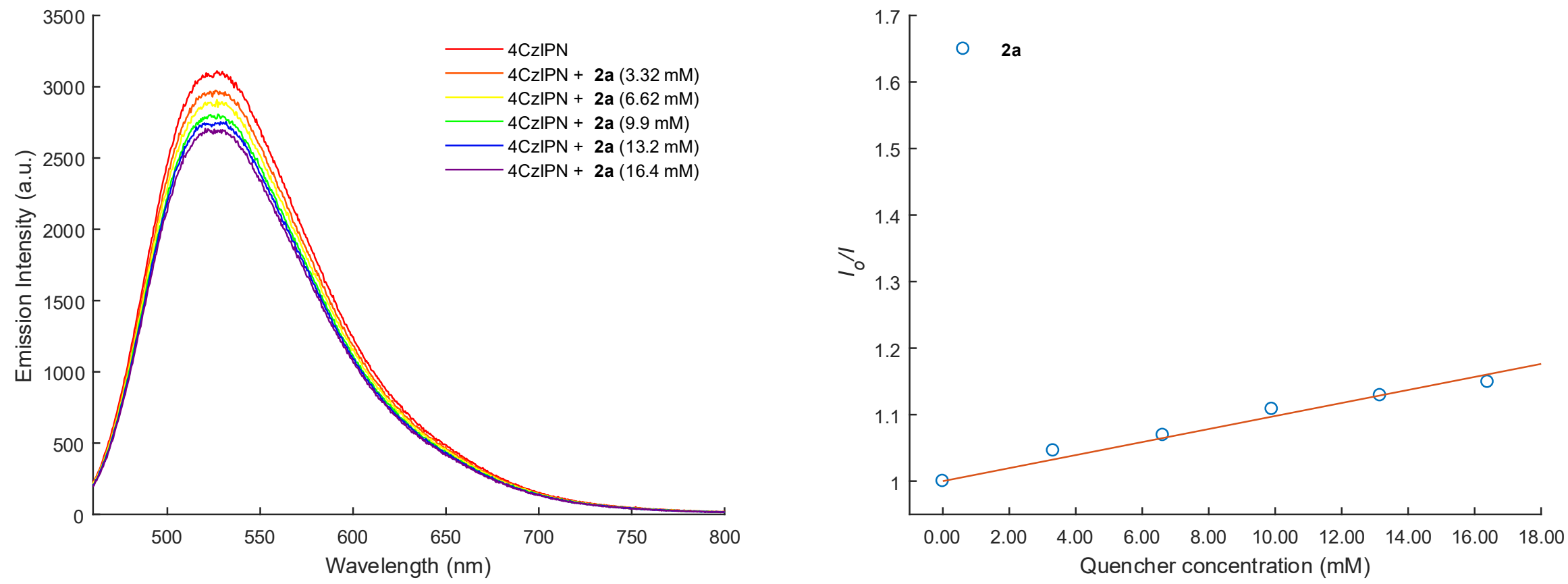

Figure S5. Luminescence quenching of 4CzIPN with Et_3N (constant amount) and HEH.

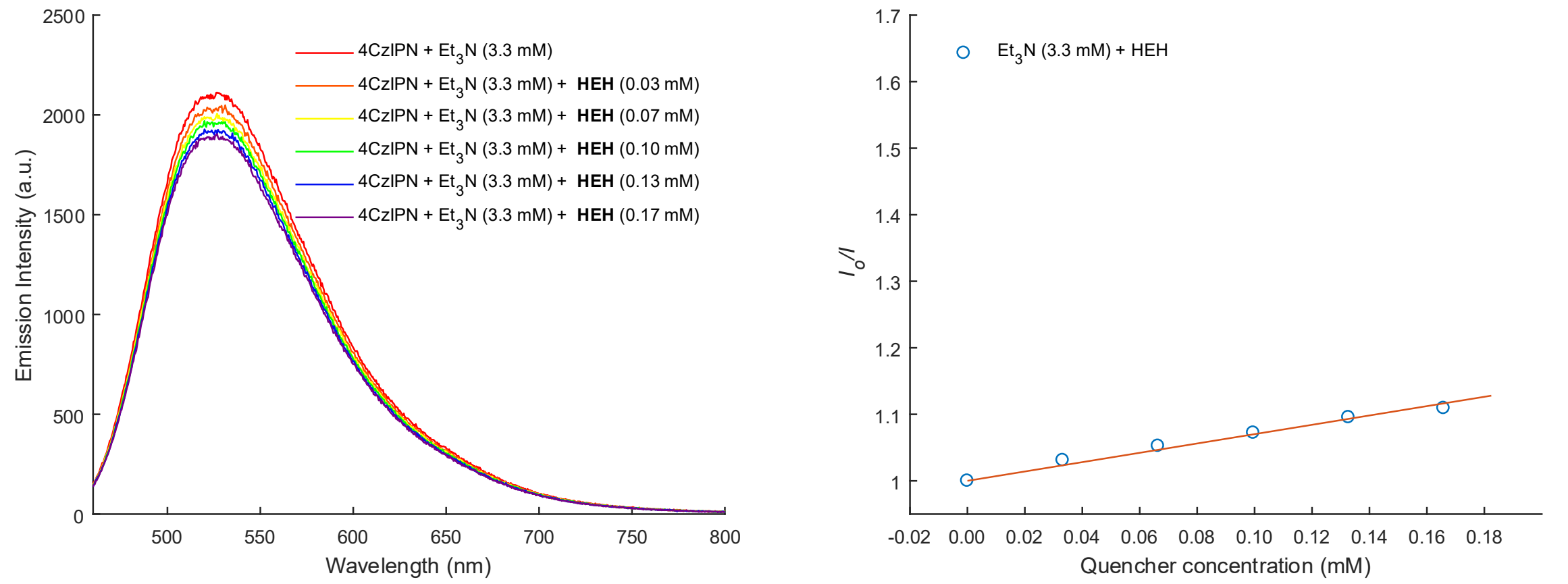

7.3 Light on/off Experiment

The light on/off experiment was carried out according to **GP2** using substrates **1a** and **2a** on a 0.075 mmol scale in a J. Young NMR tube using THF-d_8 as the solvent (1.5 mL). Mesitylene (3.1 µL) was used as an internal standard and ^{1}H analysis was used to determine the yield. The mixture was irradiated at room temperature in the EvoluChem PhotoRedOx Box™ with alternating periods of light and darkness. The methyl peak of mesitylene was used as reference and normalized to 100. The yield of product **3ca** was determined by comparison of its integral compared to that of substrate **1a** at t = 0 before light irradiation.

Figure S6. The light on/off experiment

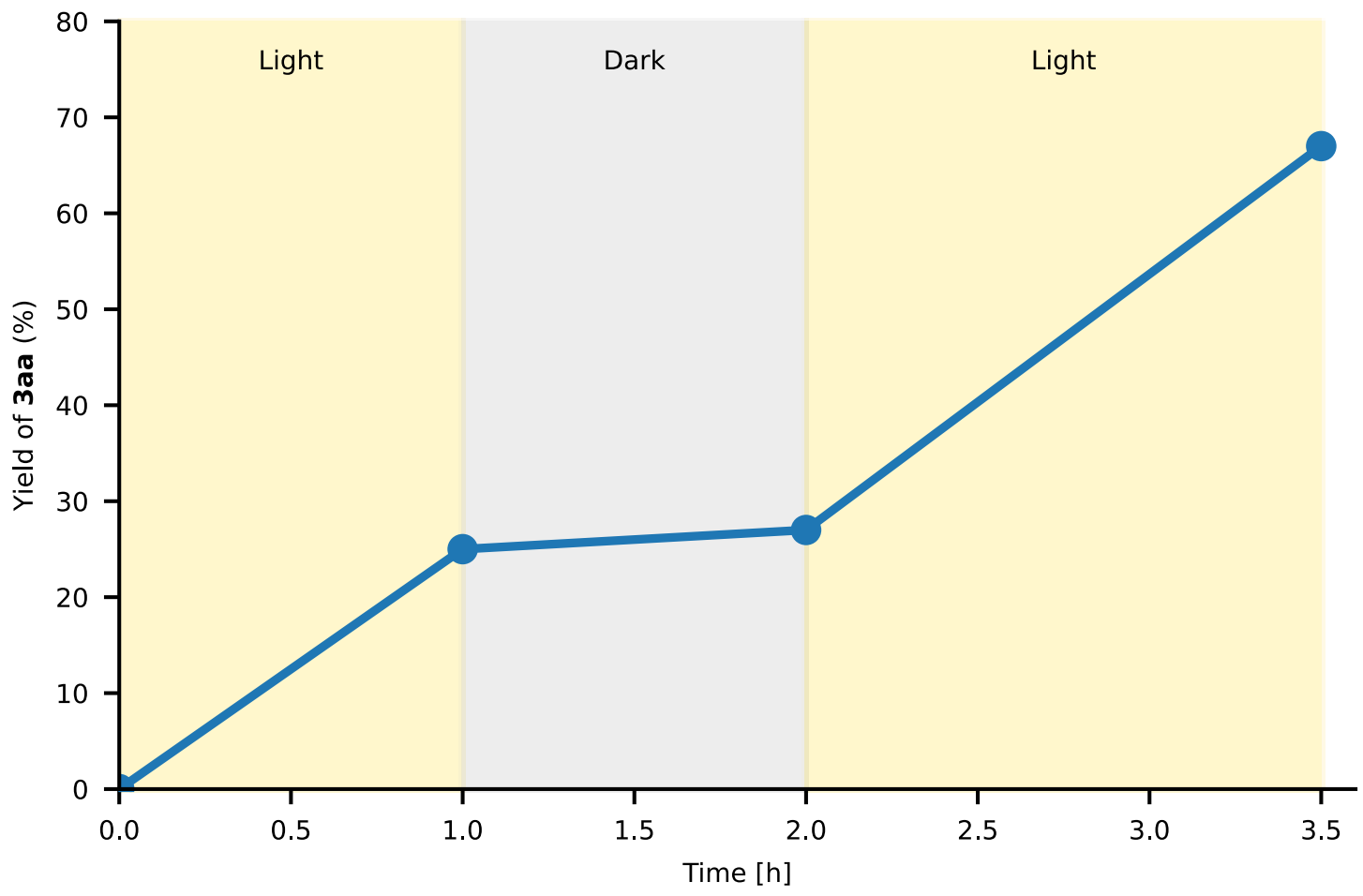

7.4 Control Experiment for Benzylic Chloride Activation

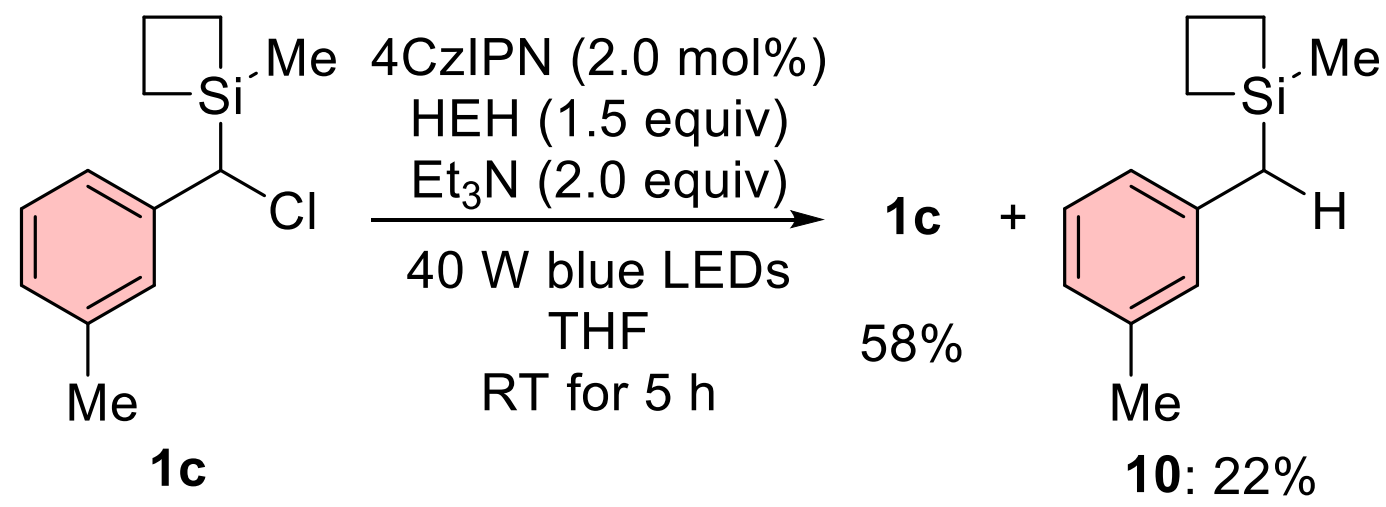

In a nitrogen-filled glovebox, a medium-sized vial was charged with benzylic chloride **1c** (22.5 mg, 0.10 mmol, 1.0 equiv) and THF (1 mL). In a GC vial, 4CzIPN (1.6 mg, 2.0 µmol, 2.0 mol%), the Hantzsch ester (67.7 mg, 0.30 mmol, 1.5 equiv) were combined and added into the medium-sized vial. The GC vial was rinsed with THF (2×0.5 mL) that was also added to the reaction mixture. Triethylamine (28 µL, 0.20 mmol, 2.0 equiv) was added. The reaction vial was capped, removed from the glovebox, placed in the photoreactor and stirred for 5 h at RT. The reaction mixture was transferred to a separatory funnel containing 1M aq. HCl and diluted with Et_2O. The two layers were separated, and the aqueous layer was extracted with Et_2O (×2). Then the combined organic layers were washed with water, brine, dried over anhydrous Na_sSO_4, filtered and concentrated under reduced pressure at

40 °C. The crude mixture was obtained as a yellow oily solid (48.7 mg). The yields of **1c** and **10** in the mixture were determined by ^{1}H NMR analysis of the crude (10.3 mg) using 1,2,3,4-tetrachlorobenzene (3.1 mg) as internal standard.

Figure S7. Stacked ^{1}H NMR spectra of **1c** (top) and the crude mixture (bottom). The following peaks were chosen for quantitative analysis: 7.33 ppm (internal standard, 2H), 4.51 ppm (**1c**, benzylic peak, 1H) and 0.23 ppm (**10**, Si*Me* peak, 3H). The biggest peaks correspond to residual HEH (not integrated here, calculated to 62.8 wt% in the sample).

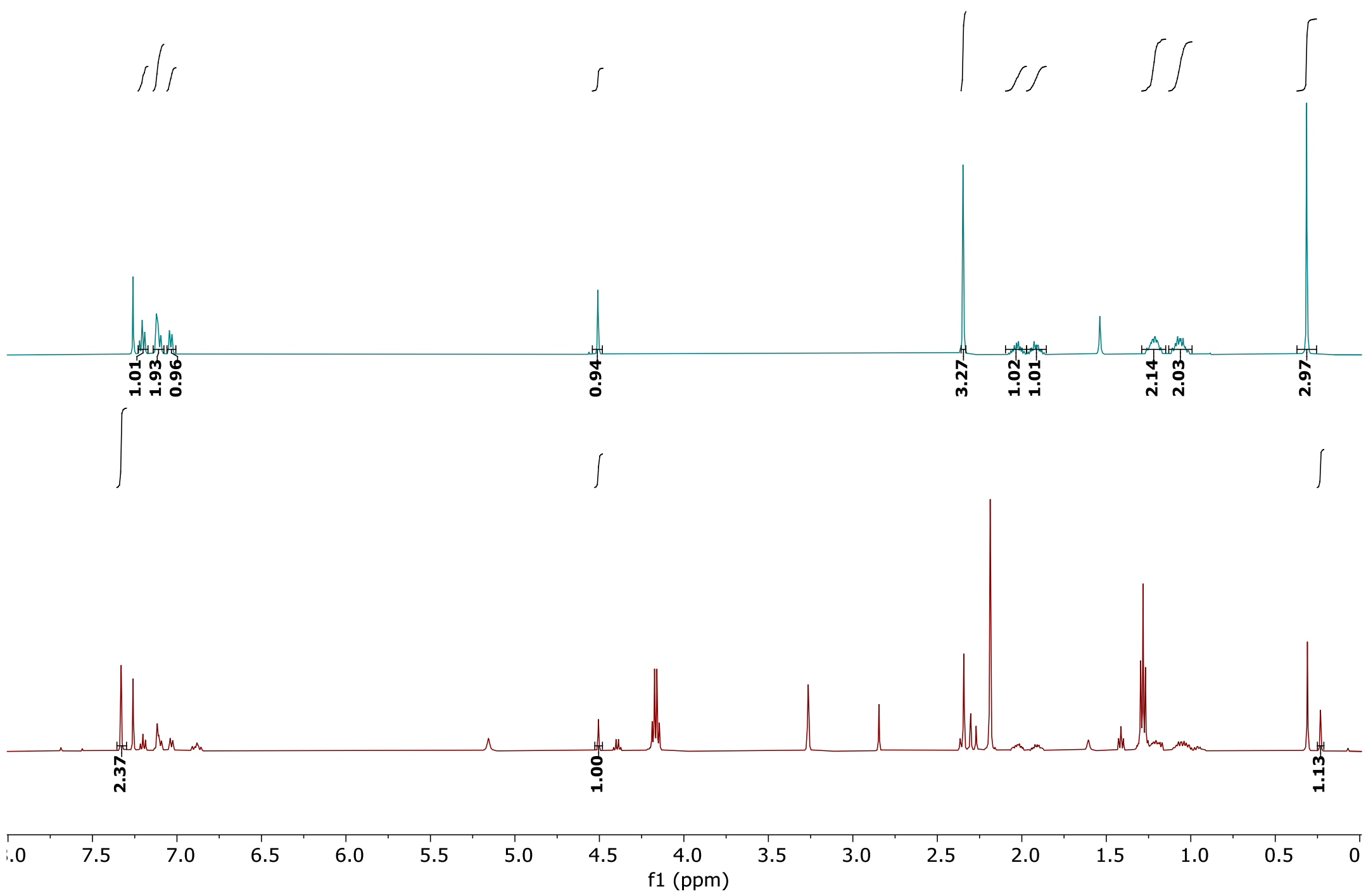

More evidence of the structure of **10** was obtained by 2D ^{1}H–^{29}Si HMQC experiment, as well as 2D ^{1}H HSQC and finally GC-MS analysis:

Figure S8. Comparison of the 2D ^{1}H–^{29}Si HMQC of **1c** (left) and the crude mixture (right). Although the silicon peaks are overlapping, there is a new correlated signal at 2.3/18.5 ppm which corresponds to the benzylic protons of **10**.

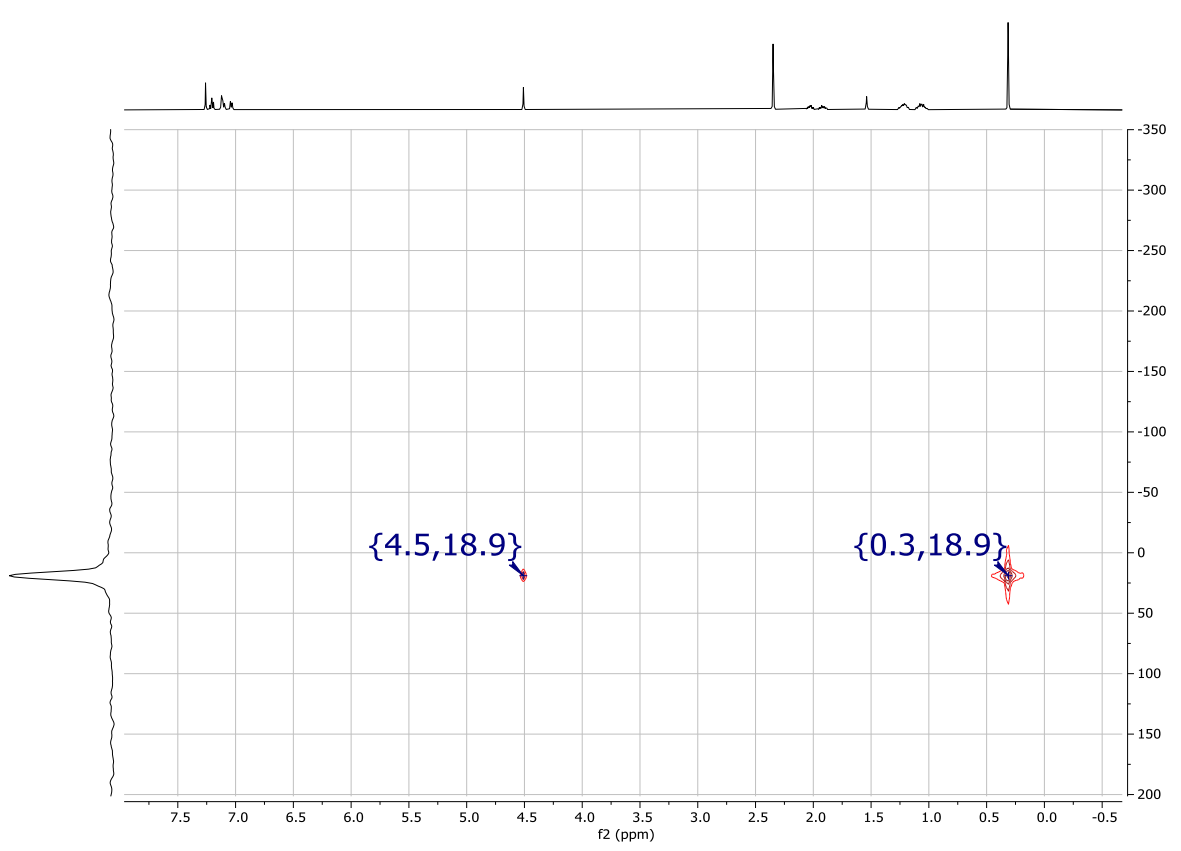

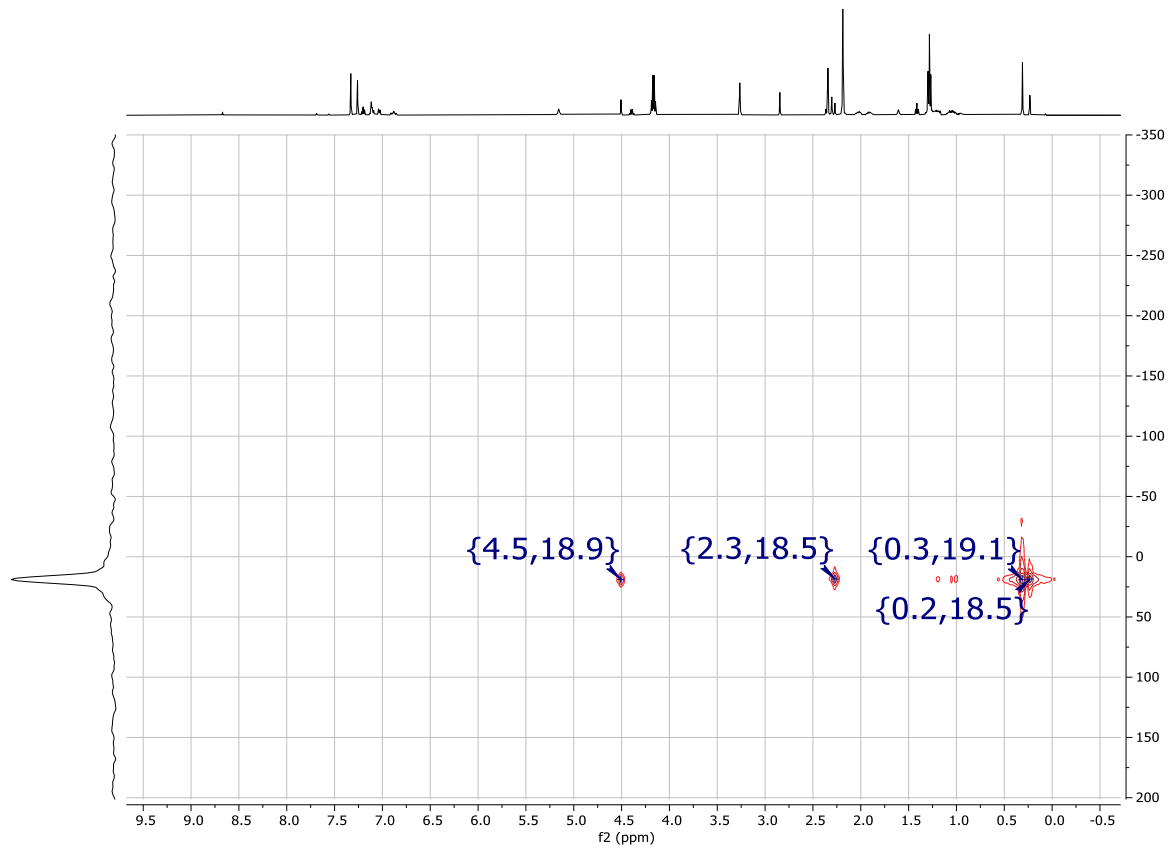

Figure S9. Multiplicity edited HSQC spectra of the crude mixture. CH_3 and CH appear in red while CH_2 appear in blue. The benzylic peak found in Figure S8 (^{1}H 2.27 ppm) appears here in blue, which supports it being a CH_2. The peak next to it was attributed to the methyl group bound to the aryl unit (^{1}H 2.30 ppm). Their respective integrations in the ^{1}H NMR match.

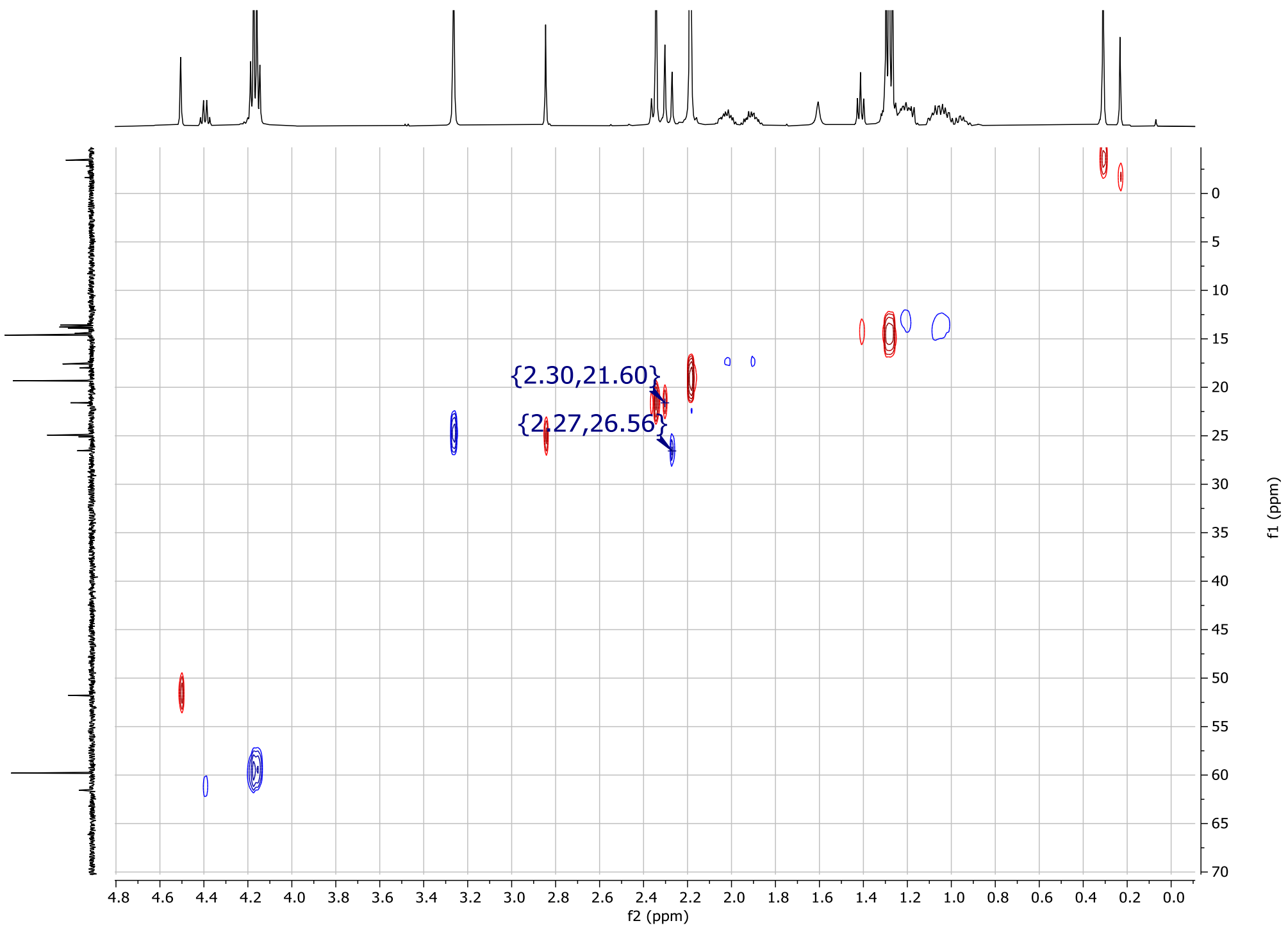

Figure S10. GC-MS analysis of the crude mixture. The first peak (t_R = 7.30 min) corresponds to product **10**, the second peak (t_R = 8.37 min) is **1c** and the third peak (t_R = 9.48 min) is the residual HEH (MS spectra not shown, mass observed as the pyridine).

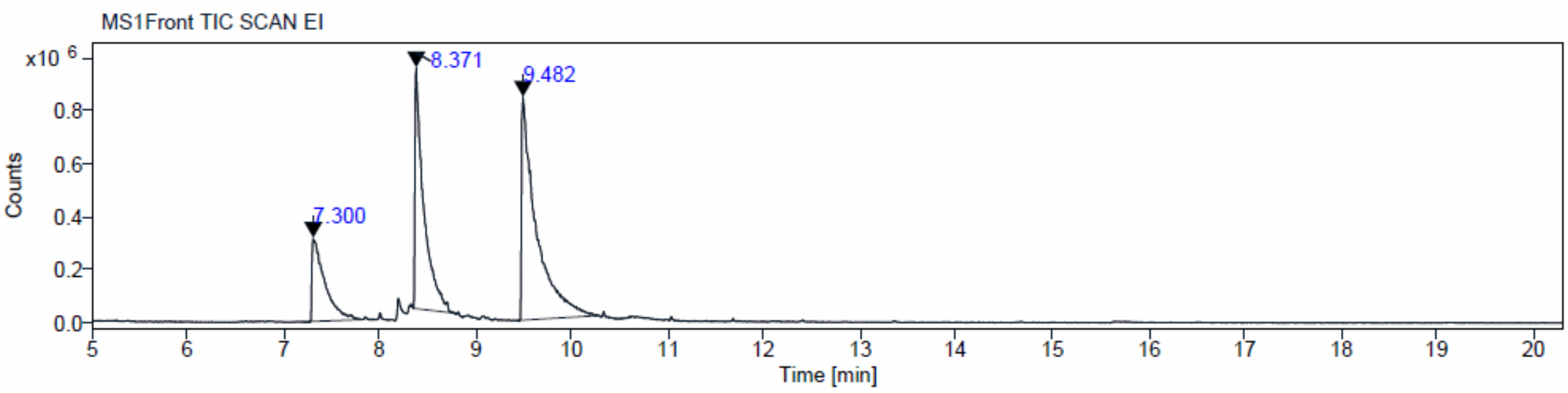

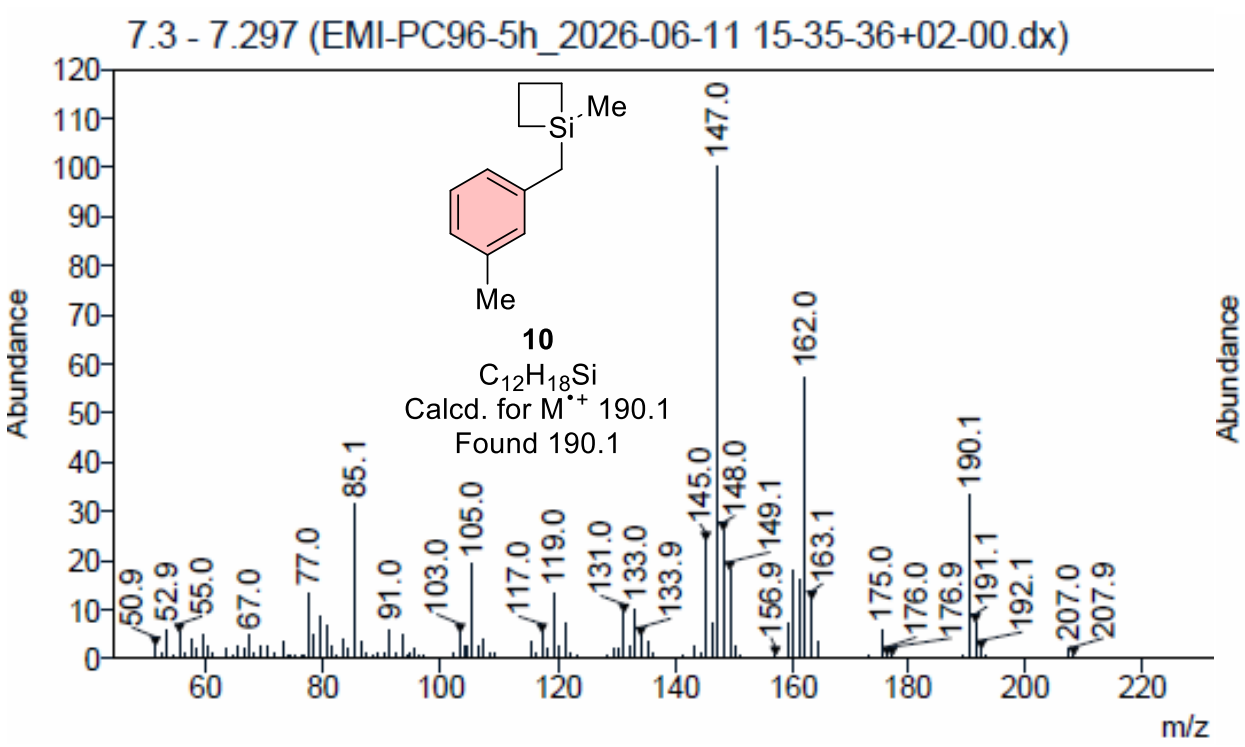

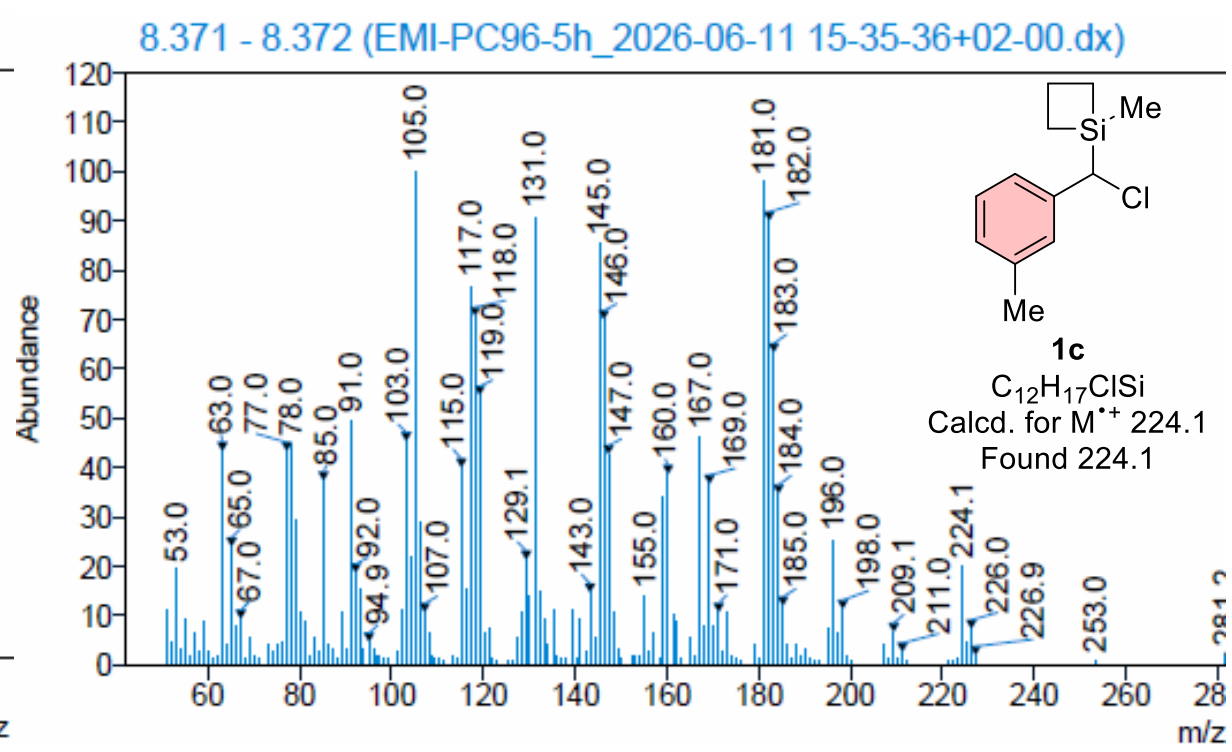

8. Determination of the Absolute Configuration

8.1 Crystal Data of (*S*)-3aa

Methyl (*S*)-4-((1-methylsiletan-1-yl)(phenyl)methyl)benzoate (**3aa**)

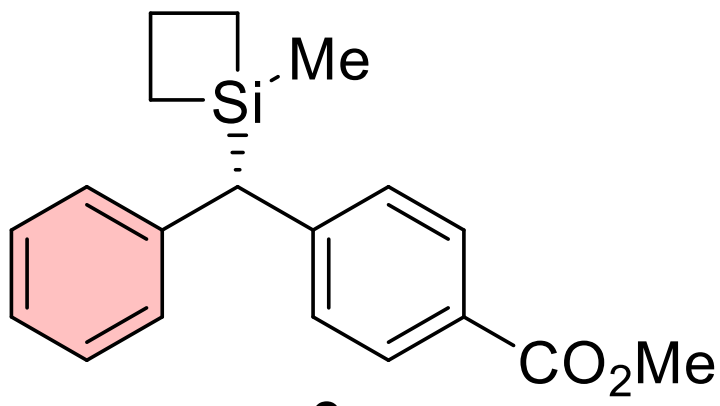

3aa

$C_{19}H_{22}O_2Si$

M_W = 310.47

Suitable crystals for X-ray analysis were grown by slow evaporation of a solution of the sample in *n*-pentane. The absolute configuration of (*S*)-**3aa** was confirmed unambiguously by X-ray diffraction analysis, and other compounds were assigned by analogy. CCDC 2571074 contains the supplementary crystallographic data for this compound. Comparison of the HPLC traces of the measured crystal can be found below under Figure S17.

Figure S11. Molecular structure of (*S*)-**3aa**.

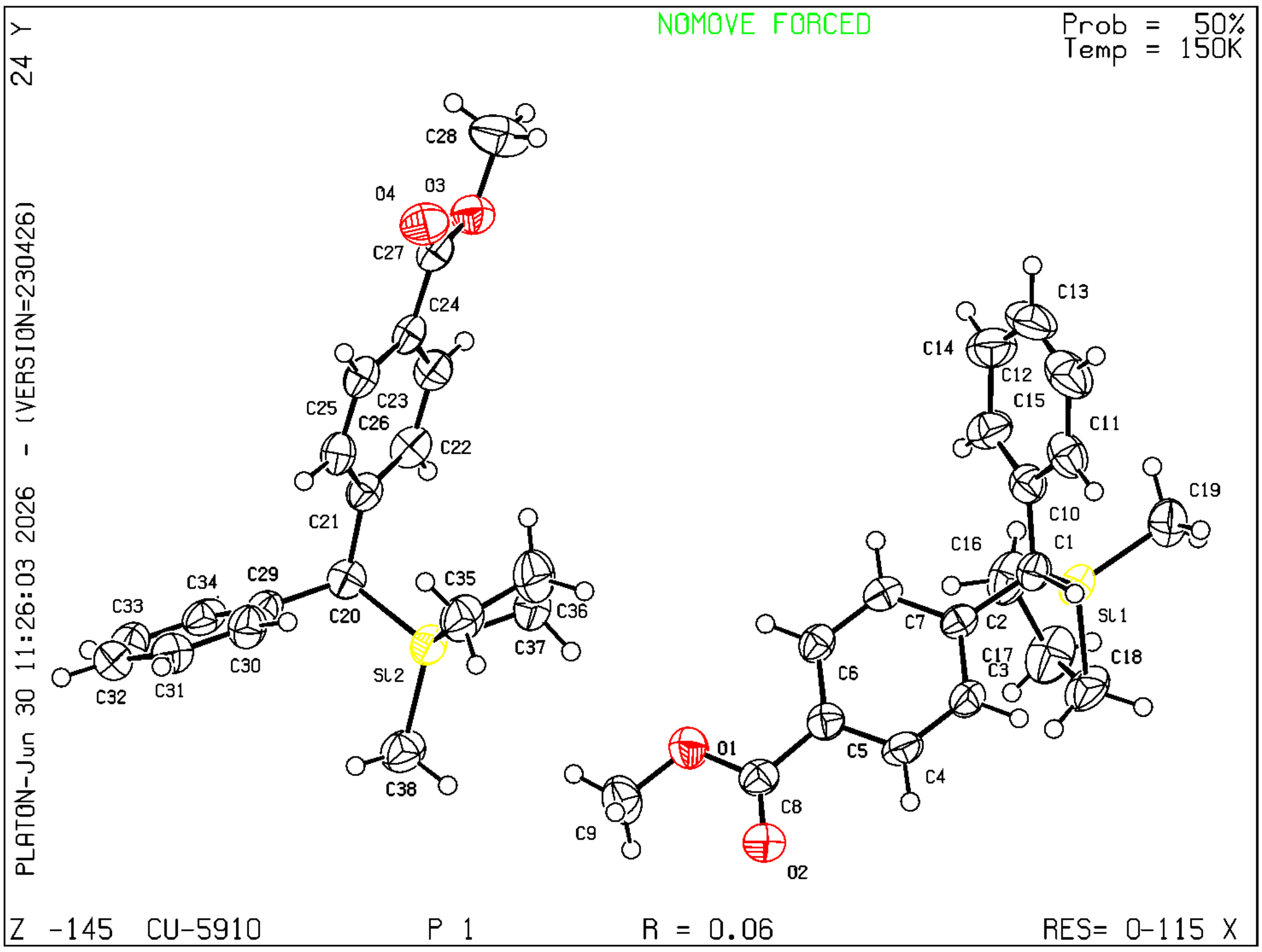

Table S2. Crystal data and structure refinement for cu-5910.

Identification code	CU-5910	
Empirical formula	$C_{19}H_{22}O_2Si$	
Formula weight	310.45	
Temperature	150.01(10) K	
Wavelength	1.54184 Å	
Crystal system	Triclinic	
Space group	P1 (No. 1)	
Unit cell dimensions	a = 8.2624(7) Å	a= 103.389(8)°.
	b = 9.6531(11) Å	b= 96.573(7)°.
	c = 12.5423(10) Å	g = 114.228(10)°.
Volume	862.34(16) $Å^3$	
Z	2	
Density (calculated)	1.196 Mg/m^3	
Absorption coefficient	1.229 mm^{-1}	
F(000)	332	
Crystal size	0.238 x 0.208 x 0.092 mm^3	
Theta range for data collection	3.728 to 72.587°.	
Index ranges	-10<=h<=10, -11<=k<=10, -10<=l<=15	
Reflections collected	6567	
Independent reflections	3850 [R(int) = 0.0333]	
Completeness to theta = 67.684°	99.8 %	
Absorption correction	Semi-empirical from equivalents	
Max. and min. transmission	1.00000 and 0.76839	
Refinement method	Full-matrix least-squares on F^2	
Data / restraints / parameters	3850 / 3 / 401	
Goodness-of-fit on F^2	1.040	
Final R indices [I>2sigma(I)]	R1 = 0.0634, wR2 = 0.1709	
R indices (all data)	R1 = 0.0730, wR2 = 0.1858	
Absolute structure parameter	0.01(5)	
Extinction coefficient	n/a	
Largest diff. peak and hole	0.549 and -0.308 e.$Å^{-3}$	

Table S3. Atomic coordinates (x 10^4) and equivalent isotropic displacement parameters ($Å^2$x 10^3) for cu-5910. U(eq) is defined as one third of the trace of the orthogonalized U^{ij} tensor.

	x	y	z	U(eq)
Si(1)	-52(2)	9074(2)	7197(1)	42(1)
Si(2)	4959(2)	1028(2)	2778(1)	44(1)
O(1)	2076(6)	4456(5)	2546(4)	52(1)
O(2)	651(6)	5413(6)	1515(4)	57(1)
O(3)	11340(6)	4640(5)	8270(3)	51(1)
O(4)	13271(6)	5616(6)	7225(4)	58(1)
C(1)	1950(7)	10039(6)	6556(4)	38(1)
C(2)	1910(7)	8858(6)	5496(4)	35(1)
C(3)	936(8)	8717(7)	4469(5)	42(1)
C(4)	769(8)	7622(8)	3485(5)	44(1)
C(5)	1606(7)	6621(7)	3499(5)	38(1)
C(6)	2599(7)	6767(6)	4530(5)	39(1)
C(7)	2753(7)	7872(7)	5519(4)	39(1)
C(8)	1360(7)	5459(7)	2425(5)	41(1)
C(9)	1913(11)	3341(9)	1514(6)	61(2)
C(10)	3700(8)	10904(7)	7462(5)	42(1)
C(11)	4766(9)	12540(8)	7643(5)	51(1)
C(12)	6315(10)	13414(10)	8517(7)	70(2)
C(13)	6840(10)	12704(12)	9229(6)	75(3)
C(14)	5800(11)	11085(12)	9065(6)	68(2)
C(15)	4229(9)	10178(9)	8179(5)	53(1)
C(16)	-569(9)	7079(8)	7396(7)	61(2)
C(17)	-2605(10)	6589(10)	6881(9)	74(2)
C(18)	-2409(8)	7829(10)	6250(7)	62(2)
C(19)	116(10)	10604(9)	8467(6)	56(2)
C(20)	6150(7)	-51(7)	3352(5)	42(1)
C(21)	7689(7)	1107(6)	4386(5)	39(1)
C(22)	7325(8)	1339(7)	5456(5)	45(1)
C(23)	8619(8)	2437(8)	6408(5)	46(1)
C(24)	10392(7)	3384(7)	6307(5)	40(1)
C(25)	10783(7)	3133(7)	5262(5)	41(1)
C(26)	9489(8)	2013(7)	4317(5)	42(1)

C(27)	11818(8)	4645(7)	7305(5)	46(1)
C(28)	12661(13)	5838(11)	9251(6)	77(2)
C(29)	6709(7)	-967(6)	2435(5)	40(1)
C(30)	7596(9)	-296(8)	1687(5)	49(1)
C(31)	8085(10)	-1155(9)	848(5)	56(2)
C(32)	7691(10)	-2709(9)	757(6)	59(2)
C(33)	6775(9)	-3415(8)	1476(6)	57(2)
C(34)	6283(8)	-2571(7)	2313(5)	48(1)
C(35)	6539(11)	3058(8)	2702(6)	58(2)
C(36)	6123(13)	3796(10)	3792(8)	74(2)
C(37)	4416(10)	2340(9)	3877(6)	59(2)
C(38)	3143(9)	-408(8)	1489(6)	57(2)

Table S4. Bond lengths [Å] and angles [°] for cu-5910.

Si(1)-C(19)	1.852(7)	C(21)-C(26)	1.408(8)
Si(1)-C(16)	1.877(7)	C(22)-C(23)	1.373(9)
Si(1)-C(18)	1.881(7)	C(23)-C(24)	1.413(8)
Si(1)-C(1)	1.907(6)	C(24)-C(25)	1.376(8)
Si(1)-C(17)	2.361(8)	C(24)-C(27)	1.489(8)
Si(2)-C(38)	1.867(7)	C(25)-C(26)	1.373(8)
Si(2)-C(37)	1.873(7)	C(29)-C(30)	1.373(9)
Si(2)-C(35)	1.890(7)	C(29)-C(34)	1.405(8)
Si(2)-C(20)	1.897(6)	C(30)-C(31)	1.389(9)
Si(2)-C(36)	2.392(8)	C(31)-C(32)	1.371(11)
O(1)-C(8)	1.352(7)	C(32)-C(33)	1.367(11)
O(1)-C(9)	1.430(8)	C(33)-C(34)	1.380(10)
O(2)-C(8)	1.204(7)	C(35)-C(36)	1.534(11)
O(3)-C(27)	1.316(7)	C(36)-C(37)	1.566(12)
O(3)-C(28)	1.429(9)		
O(4)-C(27)	1.215(8)	C(19)-Si(1)-C(16)	114.4(3)
C(1)-C(10)	1.507(8)	C(19)-Si(1)-C(18)	114.9(3)
C(1)-C(2)	1.524(7)	C(16)-Si(1)-C(18)	79.6(3)
C(2)-C(3)	1.385(7)	C(19)-Si(1)-C(1)	107.9(3)
C(2)-C(7)	1.395(7)	C(16)-Si(1)-C(1)	118.9(3)
C(3)-C(4)	1.377(8)	C(18)-Si(1)-C(1)	119.1(3)
C(4)-C(5)	1.402(8)	C(19)-Si(1)-C(17)	112.0(3)
C(5)-C(6)	1.395(7)	C(16)-Si(1)-C(17)	41.2(3)
C(5)-C(8)	1.470(8)	C(18)-Si(1)-C(17)	40.8(3)
C(6)-C(7)	1.389(8)	C(1)-Si(1)-C(17)	140.0(3)
C(10)-C(15)	1.394(9)	C(38)-Si(2)-C(37)	119.4(3)
C(10)-C(11)	1.403(9)	C(38)-Si(2)-C(35)	117.8(3)
C(11)-C(12)	1.384(10)	C(37)-Si(2)-C(35)	79.6(3)
C(12)-C(13)	1.376(14)	C(38)-Si(2)-C(20)	108.6(3)
C(13)-C(14)	1.390(14)	C(37)-Si(2)-C(20)	114.4(3)
C(14)-C(15)	1.405(10)	C(35)-Si(2)-C(20)	114.8(3)
C(16)-C(17)	1.558(10)	C(38)-Si(2)-C(36)	135.9(3)
C(17)-C(18)	1.547(12)	C(37)-Si(2)-C(36)	40.9(3)
C(20)-C(29)	1.517(8)	C(35)-Si(2)-C(36)	39.8(3)
C(20)-C(21)	1.527(8)	C(20)-Si(2)-C(36)	115.5(3)
C(21)-C(22)	1.397(8)	C(8)-O(1)-C(9)	114.9(5)

C(27)-O(3)-C(28)	116.0(6)	C(29)-C(20)-C(21)	115.0(4)
C(10)-C(1)-C(2)	116.9(4)	C(29)-C(20)-Si(2)	111.7(4)
C(10)-C(1)-Si(1)	109.6(4)	C(21)-C(20)-Si(2)	109.9(4)
C(2)-C(1)-Si(1)	111.4(4)	C(22)-C(21)-C(26)	117.1(5)
C(3)-C(2)-C(7)	118.4(5)	C(22)-C(21)-C(20)	119.9(5)
C(3)-C(2)-C(1)	118.5(5)	C(26)-C(21)-C(20)	123.0(5)
C(7)-C(2)-C(1)	123.0(4)	C(23)-C(22)-C(21)	122.7(5)
C(4)-C(3)-C(2)	121.6(5)	C(22)-C(23)-C(24)	119.0(5)
C(3)-C(4)-C(5)	120.3(5)	C(25)-C(24)-C(23)	118.8(5)
C(6)-C(5)-C(4)	118.2(5)	C(25)-C(24)-C(27)	120.1(5)
C(6)-C(5)-C(8)	123.8(5)	C(23)-C(24)-C(27)	121.0(5)
C(4)-C(5)-C(8)	118.0(5)	C(26)-C(25)-C(24)	121.8(5)
C(7)-C(6)-C(5)	121.0(5)	C(25)-C(26)-C(21)	120.5(5)
C(6)-C(7)-C(2)	120.4(5)	O(4)-C(27)-O(3)	123.4(6)
O(2)-C(8)-O(1)	122.1(5)	O(4)-C(27)-C(24)	122.6(5)
O(2)-C(8)-C(5)	124.6(5)	O(3)-C(27)-C(24)	114.0(5)
O(1)-C(8)-C(5)	113.3(5)	C(30)-C(29)-C(34)	117.7(6)
C(15)-C(10)-C(11)	118.8(6)	C(30)-C(29)-C(20)	122.3(5)
C(15)-C(10)-C(1)	122.8(5)	C(34)-C(29)-C(20)	120.0(5)
C(11)-C(10)-C(1)	118.2(5)	C(29)-C(30)-C(31)	121.6(6)
C(12)-C(11)-C(10)	120.5(7)	C(32)-C(31)-C(30)	119.9(7)
C(13)-C(12)-C(11)	121.0(8)	C(33)-C(32)-C(31)	119.7(7)
C(12)-C(13)-C(14)	119.4(7)	C(32)-C(33)-C(34)	120.8(6)
C(13)-C(14)-C(15)	120.4(8)	C(33)-C(34)-C(29)	120.4(6)
C(10)-C(15)-C(14)	120.0(7)	C(36)-C(35)-Si(2)	88.0(5)
C(17)-C(16)-Si(1)	86.3(5)	C(35)-C(36)-C(37)	101.9(6)
C(18)-C(17)-C(16)	101.5(5)	C(35)-C(36)-Si(2)	52.1(3)
C(18)-C(17)-Si(1)	52.7(3)	C(37)-C(36)-Si(2)	51.5(3)
C(16)-C(17)-Si(1)	52.5(3)	C(36)-C(37)-Si(2)	87.7(4)
C(17)-C(18)-Si(1)	86.5(5)		

Table S5. Anisotropic displacement parameters ($Å^2 x\ 10^3$) for cu-5910. The anisotropic displacement factor exponent takes the form: $-2\pi^2[h^2 a^{*2}U^{11} + ... + 2 h k a^* b^* U^{12}]$

	U^{11}	U^{22}	U^{33}	U^{23}	U^{13}	U^{12}
Si(1)	36(1)	43(1)	56(1)	20(1)	13(1)	22(1)
Si(2)	40(1)	44(1)	53(1)	15(1)	11(1)	22(1)
O(1)	60(3)	46(2)	50(2)	12(2)	9(2)	28(2)
O(2)	55(2)	64(3)	47(2)	8(2)	3(2)	31(2)
O(3)	52(2)	52(2)	47(2)	16(2)	6(2)	22(2)
O(4)	42(2)	56(3)	63(3)	17(2)	7(2)	13(2)
C(1)	42(3)	38(3)	43(3)	17(2)	12(2)	22(2)
C(2)	32(2)	35(3)	39(3)	17(2)	7(2)	14(2)
C(3)	45(3)	47(3)	43(3)	17(2)	8(2)	28(3)
C(4)	40(3)	57(3)	40(3)	18(2)	3(2)	25(3)
C(5)	30(2)	38(3)	44(3)	13(2)	11(2)	12(2)
C(6)	36(2)	36(3)	49(3)	17(2)	10(2)	17(2)
C(7)	34(2)	41(3)	42(3)	17(2)	5(2)	16(2)
C(8)	32(2)	39(3)	47(3)	13(2)	7(2)	11(2)
C(9)	74(5)	49(4)	63(4)	11(3)	17(3)	33(3)
C(10)	37(3)	44(3)	44(3)	11(2)	14(2)	17(2)
C(11)	49(3)	43(3)	54(3)	8(3)	21(3)	15(3)
C(12)	51(4)	59(4)	68(4)	-7(3)	15(3)	9(3)
C(13)	42(4)	92(6)	52(4)	-17(4)	-1(3)	17(4)
C(14)	58(4)	100(6)	46(3)	18(4)	8(3)	40(4)
C(15)	41(3)	65(4)	52(3)	19(3)	7(2)	23(3)
C(16)	50(4)	54(4)	92(5)	33(4)	29(3)	27(3)
C(17)	41(3)	59(4)	122(7)	36(4)	28(4)	15(3)
C(18)	30(3)	69(4)	84(5)	22(4)	11(3)	23(3)
C(19)	66(4)	62(4)	57(4)	24(3)	25(3)	40(3)
C(20)	36(3)	37(3)	49(3)	16(2)	12(2)	13(2)
C(21)	36(2)	35(3)	48(3)	16(2)	10(2)	16(2)
C(22)	39(3)	48(3)	54(3)	26(3)	16(2)	19(2)
C(23)	48(3)	56(3)	44(3)	22(3)	17(2)	29(3)
C(24)	39(3)	39(3)	50(3)	20(2)	12(2)	24(2)
C(25)	36(3)	42(3)	54(3)	23(2)	17(2)	21(2)
C(26)	41(3)	42(3)	50(3)	19(2)	20(2)	22(2)
C(27)	48(3)	49(3)	49(3)	18(3)	6(2)	30(3)

C(28)	86(6)	69(5)	50(4)	16(3)	-3(4)	18(4)
C(29)	38(3)	35(3)	46(3)	12(2)	0(2)	18(2)
C(30)	55(3)	47(3)	54(3)	20(3)	15(3)	28(3)
C(31)	55(4)	66(4)	49(3)	12(3)	8(3)	32(3)
C(32)	57(4)	58(4)	55(3)	-2(3)	-2(3)	33(3)
C(33)	45(3)	41(3)	78(4)	10(3)	-2(3)	21(3)
C(34)	37(3)	37(3)	62(4)	16(3)	-4(2)	12(2)
C(35)	60(4)	44(3)	74(4)	24(3)	11(3)	25(3)
C(36)	84(5)	49(4)	89(5)	10(4)	12(4)	38(4)
C(37)	58(4)	70(4)	58(4)	12(3)	13(3)	42(4)
C(38)	46(3)	50(4)	69(4)	17(3)	4(3)	19(3)

8.2 Crystal Data of (*S*)-3ba

Methyl (*S*)-4-((1-methylsiletan-1-yl)(*p*-tolyl)methyl)-benzoate (3ba)

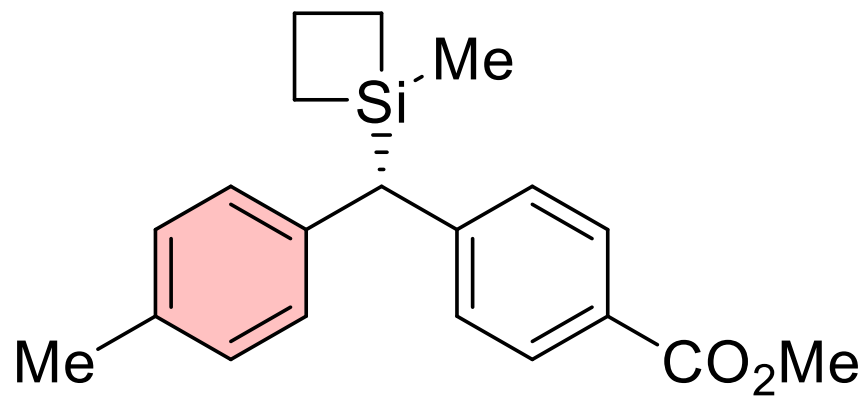

3ba

$C_{20}H_{24}O_2Si$

M_W = 324.50

Suitable crystals for X-ray analysis were grown by slow evaporation of a solution of the sample in *n*-pentane. The absolute configuration of (*S*)-**3ba** was confirmed unambiguously by X-ray diffraction analysis, and other compounds were assigned by analogy. CCDC 2563205 contains the supplementary crystallographic data for this compound. Comparison of the HPLC traces of the measured crystal can be found below under Figure S21.

Figure S12. Molecular structure of (*S*)-**3ba**.

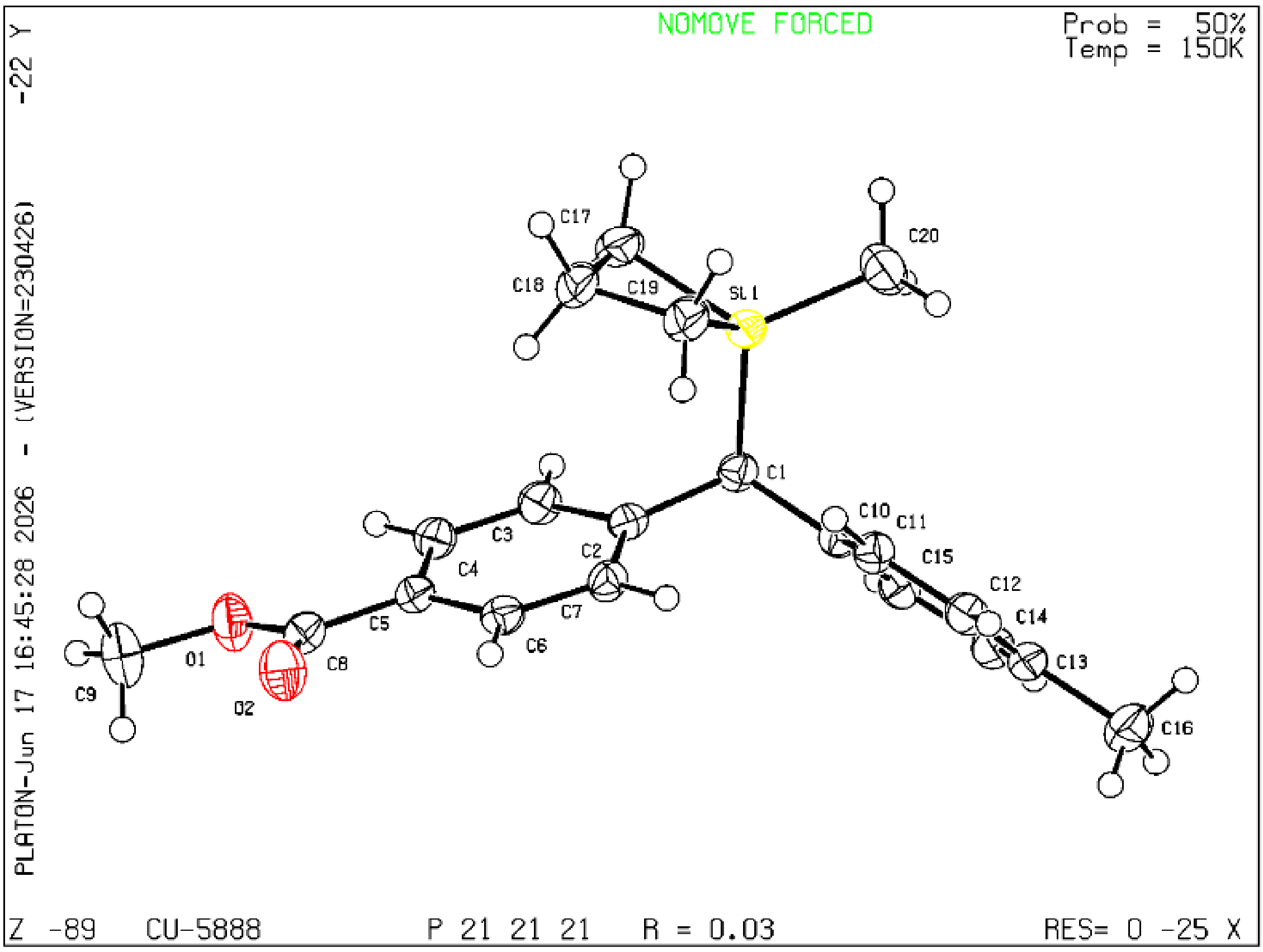

Table S6. Crystal data and structure refinement for cu-5888.

Identification code	CU-5888	
Empirical formula	$C_{20}H_{24}O_2Si$	
Formula weight	324.48	
Temperature	150.01(10) K	
Wavelength	1.54184 Å	
Crystal system	Orthorhombic	
Space group	$P2_12_12_1$ (No. 19)	
Unit cell dimensions	a = 8.1732(2) Å	a= 90°.
	b = 11.7503(4) Å	b= 90°.
	c = 18.8095(6) Å	g = 90°.
Volume	1806.42(10) $Å^3$	
Z	4	
Density (calculated)	1.193 Mg/m^3	
Absorption coefficient	1.194 mm^{-1}	
F(000)	696	
Crystal size	0.476 x 0.150 x 0.135 mm^3	
Theta range for data collection	4.437 to 72.599°.	
Index ranges	-10<=h<=8, -13<=k<=14, -19<=l<=23	
Reflections collected	7250	
Independent reflections	3361 [R(int) = 0.0229]	
Completeness to theta = 67.684°	100.0 %	
Absorption correction	Semi-empirical from equivalents	
Max. and min. transmission	1.00000 and 0.56622	
Refinement method	Full-matrix least-squares on F^2	
Data / restraints / parameters	3361 / 0 / 211	
Goodness-of-fit on F^2	1.068	
Final R indices [I>2sigma(I)]	R1 = 0.0334, wR2 = 0.0868	
R indices (all data)	R1 = 0.0362, wR2 = 0.0904	
Absolute structure parameter	-0.028(19)	
Extinction coefficient	n/a	
Largest diff. peak and hole	0.239 and -0.255 e.$Å^{-3}$	

Table S7. Atomic coordinates (x 10^4) and equivalent isotropic displacement parameters ($Å^2$x 10^3) for cu-5888. U(eq) is defined as one third of the trace of the orthogonalized U^{ij} tensor.

	x	y	z	U(eq)
Si(1)	5650(1)	2477(1)	6565(1)	27(1)
O(1)	1678(3)	6651(2)	4306(1)	42(1)
O(2)	58(3)	6778(2)	5265(1)	42(1)
C(1)	4017(3)	2045(2)	5886(1)	24(1)
C(2)	3161(3)	3094(2)	5612(1)	24(1)
C(3)	3805(3)	3670(2)	5021(1)	29(1)
C(4)	3155(3)	4698(2)	4794(1)	29(1)
C(5)	1831(3)	5178(2)	5144(1)	26(1)
C(6)	1149(3)	4603(2)	5723(1)	27(1)
C(7)	1796(3)	3568(2)	5946(1)	26(1)
C(8)	1089(3)	6282(2)	4928(1)	30(1)
C(9)	1059(4)	7733(2)	4069(2)	50(1)
C(10)	2937(3)	1107(2)	6175(1)	24(1)
C(11)	2150(3)	1174(2)	6832(1)	26(1)
C(12)	1182(3)	287(2)	7082(1)	29(1)
C(13)	1001(3)	-708(2)	6696(1)	30(1)
C(14)	1802(3)	-794(2)	6045(1)	31(1)
C(15)	2745(3)	101(2)	5789(1)	27(1)
C(16)	-45(4)	-1675(2)	6968(2)	44(1)
C(17)	6930(3)	3702(3)	6245(2)	41(1)
C(18)	5745(4)	4495(2)	6663(2)	41(1)
C(19)	4975(4)	3632(2)	7191(1)	35(1)
C(20)	6654(4)	1182(3)	6933(2)	42(1)

Table S8. Bond lengths [Å] and angles [°] for cu-5888.

		C(17)-Si(1)-C(18)	40.79(12)
Si(1)-C(20)	1.862(3)	C(19)-Si(1)-C(18)	40.63(11)
Si(1)-C(17)	1.878(3)	C(1)-Si(1)-C(18)	109.79(10)
Si(1)-C(19)	1.880(3)	C(8)-O(1)-C(9)	115.5(2)
Si(1)-C(1)	1.915(2)	C(2)-C(1)-C(10)	116.61(19)
Si(1)-C(18)	2.380(3)	C(2)-C(1)-Si(1)	109.57(15)
O(1)-C(8)	1.339(3)	C(10)-C(1)-Si(1)	111.17(15)
O(1)-C(9)	1.439(3)	C(7)-C(2)-C(3)	117.6(2)
O(2)-C(8)	1.204(3)	C(7)-C(2)-C(1)	122.82(19)
C(1)-C(2)	1.509(3)	C(3)-C(2)-C(1)	119.4(2)
C(1)-C(10)	1.513(3)	C(4)-C(3)-C(2)	121.4(2)
C(2)-C(7)	1.397(3)	C(3)-C(4)-C(5)	120.5(2)
C(2)-C(3)	1.403(3)	C(4)-C(5)-C(6)	119.0(2)
C(3)-C(4)	1.386(4)	C(4)-C(5)-C(8)	122.9(2)
C(4)-C(5)	1.387(3)	C(6)-C(5)-C(8)	118.1(2)
C(5)-C(6)	1.397(3)	C(7)-C(6)-C(5)	120.4(2)
C(5)-C(8)	1.489(3)	C(6)-C(7)-C(2)	121.1(2)
C(6)-C(7)	1.390(3)	O(2)-C(8)-O(1)	123.7(2)
C(10)-C(15)	1.395(3)	O(2)-C(8)-C(5)	124.3(2)
C(10)-C(11)	1.397(3)	O(1)-C(8)-C(5)	112.0(2)
C(11)-C(12)	1.390(3)	C(15)-C(10)-C(11)	117.1(2)
C(12)-C(13)	1.385(4)	C(15)-C(10)-C(1)	119.8(2)
C(13)-C(14)	1.392(4)	C(11)-C(10)-C(1)	123.0(2)
C(13)-C(16)	1.511(3)	C(12)-C(11)-C(10)	121.2(2)
C(14)-C(15)	1.389(3)	C(13)-C(12)-C(11)	121.2(2)
C(17)-C(18)	1.557(4)	C(12)-C(13)-C(14)	118.1(2)
C(18)-C(19)	1.552(4)	C(12)-C(13)-C(16)	121.2(2)
		C(14)-C(13)-C(16)	120.6(2)
C(20)-Si(1)-C(17)	120.02(14)	C(15)-C(14)-C(13)	120.7(2)
C(20)-Si(1)-C(19)	119.09(13)	C(14)-C(15)-C(10)	121.6(2)
C(17)-Si(1)-C(19)	79.04(12)	C(18)-C(17)-Si(1)	87.20(16)
C(20)-Si(1)-C(1)	109.78(12)	C(19)-C(18)-C(17)	100.6(2)
C(17)-Si(1)-C(1)	112.22(11)	C(19)-C(18)-Si(1)	52.11(12)
C(19)-Si(1)-C(1)	113.83(11)	C(17)-C(18)-Si(1)	52.01(12)
C(20)-Si(1)-C(18)	140.42(12)	C(18)-C(19)-Si(1)	87.26(17)

Table S9. Anisotropic displacement parameters (Å^{2}x 10^3) for cu-5888. The anisotropic displacement factor exponent takes the form: $-2\pi^2[h^2 a^{*2}U^{11} + ... + 2 h k a^* b^* U^{12}]$

	U^{11}	U^{22}	U^{33}	U^{23}	U^{13}	U^{12}
Si(1)	22(1)	29(1)	30(1)	6(1)	0(1)	-2(1)
O(1)	48(1)	34(1)	43(1)	14(1)	3(1)	5(1)
O(2)	53(1)	31(1)	44(1)	-1(1)	4(1)	8(1)
C(1)	21(1)	25(1)	27(1)	0(1)	4(1)	0(1)
C(2)	22(1)	25(1)	25(1)	-1(1)	-1(1)	-3(1)
C(3)	27(1)	33(1)	26(1)	3(1)	4(1)	-1(1)
C(4)	27(1)	33(1)	28(1)	5(1)	0(1)	-3(1)
C(5)	26(1)	25(1)	28(1)	2(1)	-4(1)	-4(1)
C(6)	23(1)	28(1)	30(1)	-2(1)	1(1)	-1(1)
C(7)	25(1)	26(1)	26(1)	3(1)	4(1)	-2(1)
C(8)	32(1)	25(1)	34(1)	0(1)	-4(1)	-4(1)
C(9)	65(2)	36(2)	50(2)	16(1)	0(1)	8(1)
C(10)	20(1)	24(1)	28(1)	4(1)	0(1)	2(1)
C(11)	26(1)	27(1)	27(1)	1(1)	3(1)	0(1)
C(12)	25(1)	36(1)	27(1)	5(1)	2(1)	1(1)
C(13)	24(1)	31(1)	36(1)	9(1)	-4(1)	-5(1)
C(14)	34(1)	26(1)	34(1)	-1(1)	-7(1)	-2(1)
C(15)	27(1)	28(1)	26(1)	1(1)	1(1)	3(1)
C(16)	41(2)	44(2)	48(2)	11(1)	-2(1)	-15(1)
C(17)	30(1)	49(2)	43(1)	14(1)	-6(1)	-14(1)
C(18)	47(2)	30(1)	45(1)	6(1)	-16(1)	-14(1)
C(19)	37(1)	32(1)	37(1)	0(1)	-5(1)	-6(1)
C(20)	39(2)	42(2)	45(1)	11(1)	-4(1)	7(1)

8.3 Crystal Data of (*R*)-3ea

Methyl (*R*)-4-((4-fluorophenyl)(1-methylsiletan-1-yl)methyl)benzoate (**3ea**)

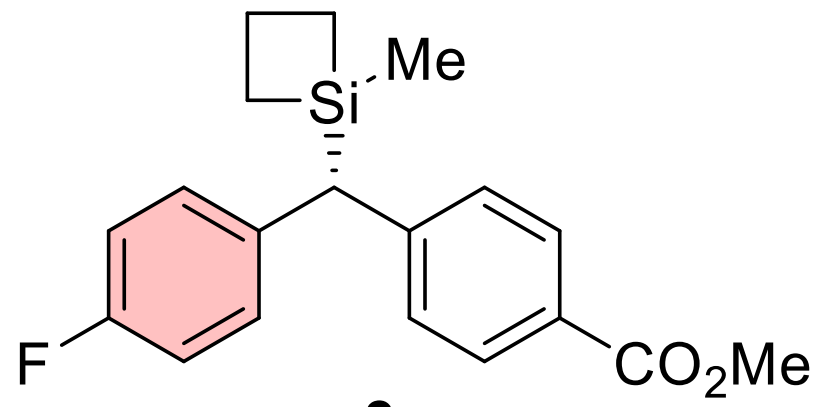

3ea

$C_{19}H_{21}FO_2Si$

M_W = 328.46

Suitable crystals for X-ray analysis were grown by slow evaporation of a solution of the sample in *n*-pentane. The absolute configuration of (*S*)-**3ba** was confirmed unambiguously by X-ray diffraction analysis, and other compounds were assigned by analogy. CCDC 2563365 contains the supplementary crystallographic data for this compound. Comparison of the HPLC traces of the measured crystal can be found below under Figure S26.

Figure S13. Molecular structure of (*R*)-**3ea**.

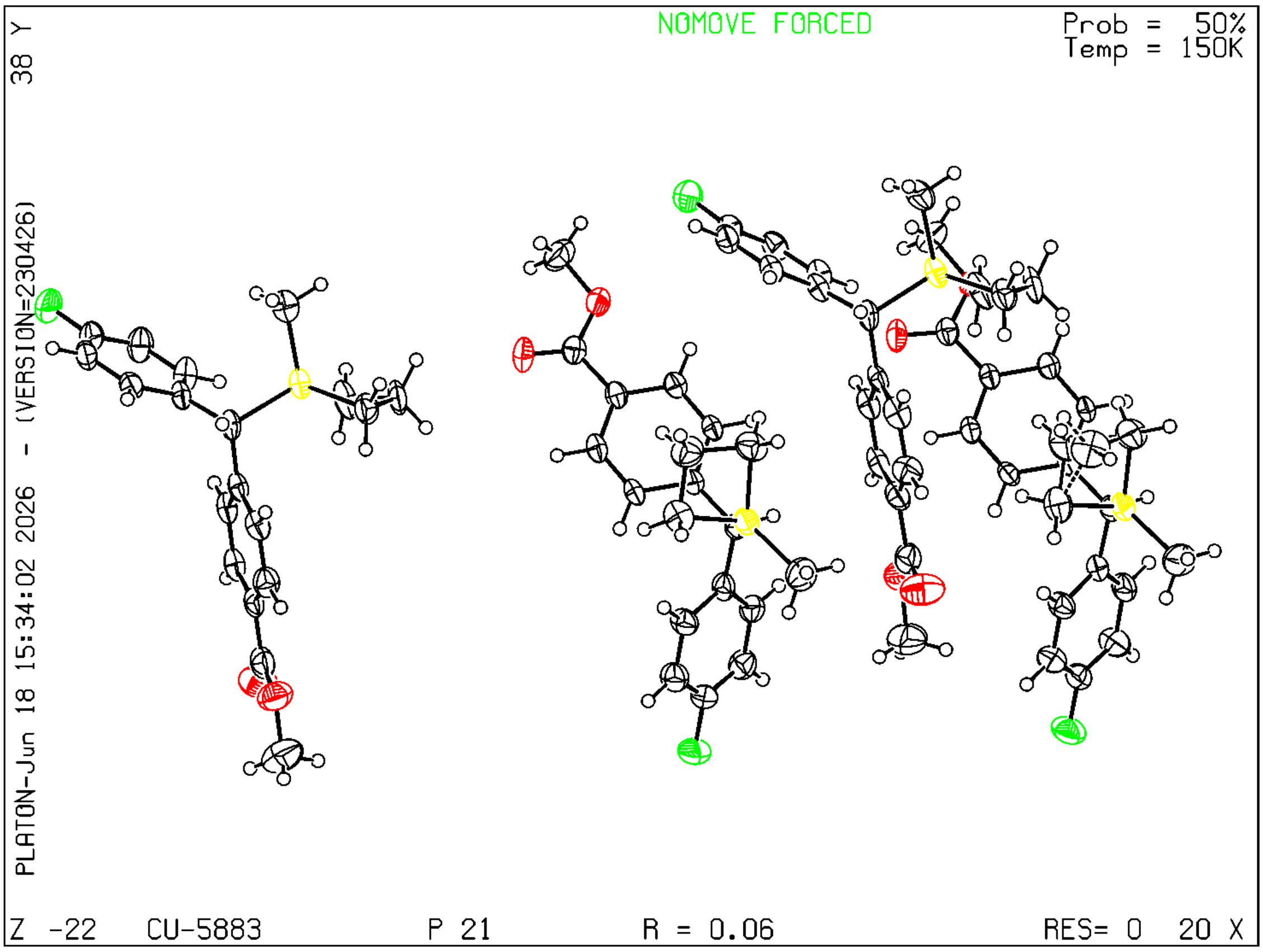

Table S10. Crystal data and structure refinement for cu-5883.

Identification code	CU-5883	
Empirical formula	$C_{19}H_{21}FO_2Si$	
Formula weight	328.45	
Temperature	150.01(10) K	
Wavelength	1.54184 Å	
Crystal system	Monoclinic	
Space group	$P2_1$ (No 4)	
Unit cell dimensions	a = 8.1653(2) Å	a= 90°.
	b = 25.8691(8) Å	b= 92.871(2)°.
	c = 16.8174(4) Å	g = 90°.
Volume	3547.86(16) $Å^3$	
Z	8	
Density (calculated)	1.230 Mg/m^3	
Absorption coefficient	1.307 mm^{-1}	
F(000)	1392	
Crystal size	0.582 x 0.512 x 0.126 mm^3	
Theta range for data collection	2.631 to 72.601°.	
Index ranges	-9<=h<=9, -27<=k<=31, -19<=l<=20	
Reflections collected	13229	
Independent reflections	9739 [R(int) = 0.0397]	
Completeness to theta = 67.684°	99.5 %	
Absorption correction	Semi-empirical from equivalents	
Max. and min. transmission	1.00000 and 0.44292	
Refinement method	Full-matrix least-squares on F^2	
Data / restraints / parameters	9739 / 7 / 847	
Goodness-of-fit on F^2	1.023	
Final R indices [I>2sigma(I)]	R1 = 0.0611, wR2 = 0.1592	
R indices (all data)	R1 = 0.0754, wR2 = 0.1754	
Absolute structure parameter	0.15(3)	
Extinction coefficient	n/a	
Largest diff. peak and hole	0.386 and -0.375 e.$Å^{-3}$	

Table S11. Atomic coordinates (x 10^4) and equivalent isotropic displacement parameters ($Å^2$x 10^3) for cu-5883. U(eq) is defined as one third of the trace of the orthogonalized U^{ij} tensor.

	x	y	z	U(eq)
Si(1)	-4001(2)	6247(1)	7672(1)	36(1)
Si(2)	6759(2)	4040(1)	4998(1)	39(1)
Si(3)	10983(2)	6141(1)	2634(1)	38(1)
F(1)	-10118(5)	7499(2)	9145(3)	67(1)
F(2)	842(5)	2502(2)	3554(2)	61(1)
F(3)	4913(5)	7374(2)	4072(3)	62(1)
O(1)	-7256(7)	3593(2)	7428(3)	64(1)
O(2)	-9192(7)	3965(2)	6653(3)	69(2)
O(3)	2612(6)	6537(2)	4983(3)	49(1)
O(4)	1167(5)	6122(2)	5886(2)	50(1)
O(5)	6310(6)	3746(2)	1784(3)	64(1)
O(6)	8356(8)	3374(2)	2524(4)	81(2)
C(1)	-5563(6)	5963(2)	8358(3)	35(1)
C(2)	-6232(6)	5454(2)	8045(3)	34(1)
C(3)	-7657(6)	5421(3)	7550(3)	39(1)
C(4)	-8226(7)	4951(3)	7270(3)	41(1)
C(5)	-7409(7)	4496(3)	7459(3)	38(1)
C(6)	-5950(7)	4523(3)	7947(4)	45(1)
C(7)	-5398(7)	4994(3)	8239(3)	40(1)
C(8)	-8052(8)	4007(3)	7129(4)	50(2)
C(9)	-7901(13)	3102(3)	7123(6)	93(3)
C(10)	-6851(6)	6359(2)	8557(3)	36(1)
C(11)	-7628(7)	6663(3)	7969(4)	48(2)
C(12)	-8753(7)	7050(3)	8162(4)	52(2)
C(13)	-9053(7)	7117(3)	8957(4)	48(2)
C(14)	-8313(7)	6828(3)	9539(4)	46(2)
C(15)	-7202(7)	6442(2)	9347(3)	39(1)
C(16)	-4420(7)	6196(3)	6564(3)	53(2)
C(17)	-2567(7)	6039(3)	6527(4)	52(2)
C(18)	-2214(7)	5848(3)	7391(4)	47(2)
C(19)	-3404(8)	6906(3)	8036(4)	56(2)
C(20)	4992(7)	4203(2)	4253(3)	34(1)

C(21)	4148(6)	4696(2)	4503(3)	31(1)
C(22)	4699(7)	5173(2)	4240(3)	35(1)
C(23)	4049(6)	5633(2)	4483(3)	34(1)
C(24)	2792(6)	5629(2)	5027(3)	32(1)
C(25)	2209(6)	5162(2)	5281(3)	35(1)
C(26)	2862(6)	4695(2)	5027(3)	34(1)
C(27)	2096(6)	6106(2)	5349(3)	36(1)
C(28)	2002(10)	7026(3)	5282(5)	63(2)
C(29)	3864(7)	3749(2)	4081(3)	37(1)
C(30)	3374(8)	3631(2)	3299(3)	42(1)
C(31)	2358(8)	3208(3)	3118(4)	49(2)
C(32)	1836(8)	2917(2)	3718(4)	45(2)
C(33)	2283(8)	3015(3)	4506(4)	48(2)
C(34)	3292(8)	3434(2)	4681(3)	43(1)
C(35)	7998(8)	4612(3)	5335(4)	52(2)
C(36)	6989(9)	4617(3)	6100(4)	58(2)
C(37)	6247(8)	4069(3)	6083(4)	52(2)
C(38)	7814(9)	3450(3)	4646(5)	61(2)
C(39)	9506(6)	5832(2)	3340(3)	35(1)
C(40)	8947(6)	5300(2)	3049(3)	34(1)
C(41)	7537(7)	5222(3)	2545(3)	40(1)
C(42)	7111(7)	4721(3)	2303(3)	45(2)
C(43)	8037(8)	4298(3)	2538(4)	44(1)
C(44)	9444(8)	4377(3)	3032(4)	49(2)
C(45)	9866(7)	4870(3)	3283(4)	46(2)
C(46)	7621(9)	3757(3)	2307(4)	54(2)
C(47)	5856(11)	3198(4)	1612(6)	89(3)
C(48)	8175(6)	6217(2)	3530(3)	35(1)
C(49)	7217(7)	6465(3)	2933(3)	42(1)
C(50)	6083(7)	6843(3)	3112(4)	42(1)
C(51)	5965(7)	6980(2)	3901(4)	45(2)
C(52)	6873(7)	6752(3)	4509(4)	45(2)
C(53)	7982(7)	6365(2)	4319(3)	38(1)
C(54)	10529(7)	6073(3)	1531(3)	53(2)
C(55)	12395(7)	5949(3)	1481(4)	54(2)
C(56)	12830(7)	5765(3)	2349(4)	47(2)
C(57)	11446(8)	6814(3)	2958(4)	56(2)
Si(4)	11548(2)	4009(1)	-86(1)	42(1)

F(4)	5504(5)	2546(2)	-1569(2)	67(1)
O(7)	7507(5)	6526(2)	24(3)	45(1)
O(8)	6115(5)	6108(2)	947(2)	47(1)
C(58)	9766(6)	4204(2)	-804(3)	35(1)
C(59)	8971(6)	4694(2)	-522(3)	30(1)
C(60)	7717(6)	4687(2)	14(3)	33(1)
C(61)	7072(6)	5151(2)	290(3)	35(1)
C(62)	7659(6)	5620(2)	48(3)	30(1)
C(63)	8903(7)	5632(2)	-504(3)	36(1)
C(64)	9535(6)	5173(2)	-770(3)	35(1)
C(65)	6996(6)	6093(2)	388(3)	35(1)
C(66)	6920(10)	7008(3)	345(5)	62(2)
C(67)	8593(6)	3762(2)	-997(3)	35(1)
C(68)	8083(7)	3420(2)	-419(3)	40(1)
C(69)	7051(8)	3016(2)	-606(4)	44(1)
C(70)	6537(8)	2948(2)	-1385(4)	44(1)
C(71)	6994(9)	3264(3)	-1979(4)	52(2)
C(72)	8036(8)	3677(3)	-1779(3)	43(1)
C(73)	12926(8)	4544(3)	277(5)	57(2)
C(74)	12780(40)	4369(13)	1072(15)	68(6)
C(74A)	11930(20)	4512(7)	1086(8)	66(4)
C(75)	11125(8)	4001(3)	1008(4)	56(2)
C(76)	12538(9)	3426(3)	-501(5)	60(2)

Table S12. Bond lengths [Å] and angles [°] for cu-5883.

Si(1)-C(19)	1.867(7)	C(6)-C(7)	1.380(9)
Si(1)-C(18)	1.868(6)	C(10)-C(15)	1.390(7)
Si(1)-C(16)	1.882(6)	C(10)-C(11)	1.392(8)
Si(1)-C(1)	1.909(5)	C(11)-C(12)	1.406(9)
Si(1)-C(17)	2.365(6)	C(12)-C(13)	1.382(9)
Si(2)-C(38)	1.862(7)	C(13)-C(14)	1.350(10)
Si(2)-C(35)	1.866(7)	C(14)-C(15)	1.398(9)
Si(2)-C(37)	1.893(6)	C(16)-C(17)	1.571(8)
Si(2)-C(20)	1.911(6)	C(17)-C(18)	1.548(9)
Si(2)-C(36)	2.379(7)	C(20)-C(29)	1.513(8)
Si(3)-C(57)	1.859(7)	C(20)-C(21)	1.519(7)
Si(3)-C(56)	1.876(6)	C(21)-C(22)	1.392(7)
Si(3)-C(54)	1.882(6)	C(21)-C(26)	1.404(7)
Si(3)-C(39)	1.910(5)	C(22)-C(23)	1.374(8)
Si(3)-C(55)	2.358(6)	C(23)-C(24)	1.408(7)
F(1)-C(13)	1.364(7)	C(24)-C(25)	1.375(8)
F(2)-C(32)	1.366(7)	C(24)-C(27)	1.472(8)
F(3)-C(51)	1.372(7)	C(25)-C(26)	1.398(8)
O(1)-C(8)	1.339(9)	C(29)-C(30)	1.389(8)
O(1)-C(9)	1.457(10)	C(29)-C(34)	1.394(8)
O(2)-C(8)	1.202(8)	C(30)-C(31)	1.396(9)
O(3)-C(27)	1.352(7)	C(31)-C(32)	1.345(9)
O(3)-C(28)	1.458(8)	C(32)-C(33)	1.381(9)
O(4)-C(27)	1.209(6)	C(33)-C(34)	1.385(9)
O(5)-C(46)	1.351(9)	C(35)-C(36)	1.562(10)
O(5)-C(47)	1.491(10)	C(36)-C(37)	1.539(10)
O(6)-C(46)	1.206(9)	C(39)-C(48)	1.521(8)
C(1)-C(2)	1.510(8)	C(39)-C(40)	1.522(8)
C(1)-C(10)	1.517(8)	C(40)-C(45)	1.389(9)
C(2)-C(3)	1.399(7)	C(40)-C(41)	1.410(7)
C(2)-C(7)	1.401(8)	C(41)-C(42)	1.397(9)
C(3)-C(4)	1.375(9)	C(42)-C(43)	1.376(9)
C(4)-C(5)	1.381(9)	C(43)-C(44)	1.399(9)
C(5)-C(6)	1.414(8)	C(43)-C(46)	1.487(10)
C(5)-C(8)	1.469(9)	C(44)-C(45)	1.380(9)

C(48)-C(53)	1.396(8)	C(19)-Si(1)-C(18)	113.2(3)
C(48)-C(49)	1.397(8)	C(19)-Si(1)-C(16)	114.8(4)
C(49)-C(50)	1.390(8)	C(18)-Si(1)-C(16)	79.4(3)
C(50)-C(51)	1.382(9)	C(19)-Si(1)-C(1)	109.0(3)
C(51)-C(52)	1.366(9)	C(18)-Si(1)-C(1)	119.6(3)
C(52)-C(53)	1.397(9)	C(16)-Si(1)-C(1)	118.5(3)
C(54)-C(55)	1.563(8)	C(19)-Si(1)-C(17)	110.0(3)
C(55)-C(56)	1.559(9)	C(18)-Si(1)-C(17)	40.8(3)
Si(4)-C(76)	1.863(7)	C(16)-Si(1)-C(17)	41.5(2)
Si(4)-C(73)	1.867(7)	C(1)-Si(1)-C(17)	141.0(3)
Si(4)-C(75)	1.889(7)	C(38)-Si(2)-C(35)	119.6(3)
Si(4)-C(58)	1.911(6)	C(38)-Si(2)-C(37)	117.9(3)
Si(4)-C(74)	2.34(2)	C(35)-Si(2)-C(37)	79.5(3)
Si(4)-C(74A)	2.369(14)	C(38)-Si(2)-C(20)	108.6(3)
F(4)-C(70)	1.365(7)	C(35)-Si(2)-C(20)	113.8(3)
O(7)-C(65)	1.351(7)	C(37)-Si(2)-C(20)	115.3(3)
O(7)-C(66)	1.450(8)	C(38)-Si(2)-C(36)	137.9(3)
O(8)-C(65)	1.213(6)	C(35)-Si(2)-C(36)	41.0(3)
C(58)-C(59)	1.511(8)	C(37)-Si(2)-C(36)	40.2(3)
C(58)-C(67)	1.515(8)	C(20)-Si(2)-C(36)	113.5(3)
C(59)-C(64)	1.394(7)	C(57)-Si(3)-C(56)	114.0(3)
C(59)-C(60)	1.398(7)	C(57)-Si(3)-C(54)	113.8(4)
C(60)-C(61)	1.397(8)	C(56)-Si(3)-C(54)	79.7(3)
C(61)-C(62)	1.373(8)	C(57)-Si(3)-C(39)	109.6(3)
C(62)-C(63)	1.411(7)	C(56)-Si(3)-C(39)	118.8(3)
C(62)-C(65)	1.467(8)	C(54)-Si(3)-C(39)	118.3(3)
C(63)-C(64)	1.378(8)	C(57)-Si(3)-C(55)	109.8(3)
C(67)-C(72)	1.387(8)	C(56)-Si(3)-C(55)	41.3(3)
C(67)-C(68)	1.393(8)	C(54)-Si(3)-C(55)	41.4(2)
C(68)-C(69)	1.369(8)	C(39)-Si(3)-C(55)	140.6(3)
C(69)-C(70)	1.367(9)	C(8)-O(1)-C(9)	113.9(6)
C(70)-C(71)	1.357(9)	C(27)-O(3)-C(28)	116.1(5)
C(71)-C(72)	1.396(9)	C(46)-O(5)-C(47)	109.2(7)
C(73)-C(74)	1.42(3)	C(2)-C(1)-C(10)	115.0(4)
C(73)-C(74A)	1.620(16)	C(2)-C(1)-Si(1)	111.5(3)
C(74)-C(75)	1.65(3)	C(10)-C(1)-Si(1)	111.4(4)
C(74A)-C(75)	1.480(16)	C(3)-C(2)-C(7)	117.8(6)
		C(3)-C(2)-C(1)	122.4(5)

C(7)-C(2)-C(1)	119.7(5)	C(25)-C(26)-C(21)	119.9(5)
C(4)-C(3)-C(2)	121.0(6)	O(4)-C(27)-O(3)	122.2(6)
C(3)-C(4)-C(5)	121.5(5)	O(4)-C(27)-C(24)	125.0(6)
C(4)-C(5)-C(6)	118.4(6)	O(3)-C(27)-C(24)	112.8(4)
C(4)-C(5)-C(8)	119.1(6)	C(30)-C(29)-C(34)	117.8(6)
C(6)-C(5)-C(8)	122.5(6)	C(30)-C(29)-C(20)	119.7(5)
C(7)-C(6)-C(5)	120.0(6)	C(34)-C(29)-C(20)	122.5(5)
C(6)-C(7)-C(2)	121.4(5)	C(29)-C(30)-C(31)	121.2(6)
O(2)-C(8)-O(1)	121.5(7)	C(32)-C(31)-C(30)	118.8(6)
O(2)-C(8)-C(5)	125.5(7)	C(31)-C(32)-F(2)	119.7(6)
O(1)-C(8)-C(5)	113.0(5)	C(31)-C(32)-C(33)	122.6(6)
C(15)-C(10)-C(11)	118.8(6)	F(2)-C(32)-C(33)	117.6(6)
C(15)-C(10)-C(1)	119.6(5)	C(32)-C(33)-C(34)	118.2(6)
C(11)-C(10)-C(1)	121.5(5)	C(33)-C(34)-C(29)	121.4(6)
C(10)-C(11)-C(12)	121.2(6)	C(36)-C(35)-Si(2)	87.4(4)
C(13)-C(12)-C(11)	117.6(6)	C(37)-C(36)-C(35)	101.6(5)
C(14)-C(13)-F(1)	120.0(6)	C(37)-C(36)-Si(2)	52.6(3)
C(14)-C(13)-C(12)	122.5(6)	C(35)-C(36)-Si(2)	51.6(3)
F(1)-C(13)-C(12)	117.5(6)	C(36)-C(37)-Si(2)	87.1(4)
C(13)-C(14)-C(15)	119.9(6)	C(48)-C(39)-C(40)	117.0(4)
C(10)-C(15)-C(14)	120.0(6)	C(48)-C(39)-Si(3)	109.7(4)
C(17)-C(16)-Si(1)	85.9(4)	C(40)-C(39)-Si(3)	111.5(3)
C(18)-C(17)-C(16)	100.4(4)	C(45)-C(40)-C(41)	117.9(6)
C(18)-C(17)-Si(1)	52.1(3)	C(45)-C(40)-C(39)	119.0(5)
C(16)-C(17)-Si(1)	52.6(3)	C(41)-C(40)-C(39)	123.1(5)
C(17)-C(18)-Si(1)	87.1(4)	C(42)-C(41)-C(40)	119.5(6)
C(29)-C(20)-C(21)	115.1(4)	C(43)-C(42)-C(41)	121.9(5)
C(29)-C(20)-Si(2)	112.7(4)	C(42)-C(43)-C(44)	118.5(6)
C(21)-C(20)-Si(2)	110.0(4)	C(42)-C(43)-C(46)	123.9(6)
C(22)-C(21)-C(26)	117.7(5)	C(44)-C(43)-C(46)	117.6(6)
C(22)-C(21)-C(20)	119.8(4)	C(45)-C(44)-C(43)	120.1(6)
C(26)-C(21)-C(20)	122.4(5)	C(44)-C(45)-C(40)	122.1(6)
C(23)-C(22)-C(21)	122.5(5)	O(6)-C(46)-O(5)	123.1(7)
C(22)-C(23)-C(24)	119.5(5)	O(6)-C(46)-C(43)	126.2(7)
C(25)-C(24)-C(23)	118.9(5)	O(5)-C(46)-C(43)	110.7(7)
C(25)-C(24)-C(27)	118.4(5)	C(53)-C(48)-C(49)	118.1(5)
C(23)-C(24)-C(27)	122.8(5)	C(53)-C(48)-C(39)	119.7(5)
C(24)-C(25)-C(26)	121.5(5)	C(49)-C(48)-C(39)	122.0(5)

C(50)-C(49)-C(48)	121.5(6)
C(51)-C(50)-C(49)	117.9(6)
C(52)-C(51)-F(3)	119.0(6)
C(52)-C(51)-C(50)	123.1(6)
F(3)-C(51)-C(50)	117.8(6)
C(51)-C(52)-C(53)	118.1(6)
C(48)-C(53)-C(52)	121.3(5)
C(55)-C(54)-Si(3)	85.8(3)
C(56)-C(55)-C(54)	100.9(4)
C(56)-C(55)-Si(3)	52.6(3)
C(54)-C(55)-Si(3)	52.8(3)
C(55)-C(56)-Si(3)	86.2(4)
C(76)-Si(4)-C(73)	117.2(3)
C(76)-Si(4)-C(75)	117.3(4)
C(73)-Si(4)-C(75)	80.0(3)
C(76)-Si(4)-C(58)	107.9(3)
C(73)-Si(4)-C(58)	116.1(3)
C(75)-Si(4)-C(58)	116.6(3)
C(76)-Si(4)-C(74)	117.2(10)
C(73)-Si(4)-C(74)	37.4(7)
C(75)-Si(4)-C(74)	44.5(8)
C(58)-Si(4)-C(74)	134.7(9)
C(76)-Si(4)-C(74A)	135.5(6)
C(73)-Si(4)-C(74A)	43.0(4)
C(75)-Si(4)-C(74A)	38.6(4)
C(58)-Si(4)-C(74A)	116.5(5)
C(65)-O(7)-C(66)	115.4(5)
C(59)-C(58)-C(67)	115.0(4)
C(59)-C(58)-Si(4)	110.3(4)
C(67)-C(58)-Si(4)	112.9(4)
C(64)-C(59)-C(60)	117.8(5)
C(64)-C(59)-C(58)	119.9(4)
C(60)-C(59)-C(58)	122.2(5)
C(61)-C(60)-C(59)	120.3(5)
C(62)-C(61)-C(60)	121.1(5)
C(61)-C(62)-C(63)	119.2(5)
C(61)-C(62)-C(65)	118.8(5)
C(63)-C(62)-C(65)	121.9(5)
C(64)-C(63)-C(62)	119.1(5)
C(63)-C(64)-C(59)	122.4(5)
O(8)-C(65)-O(7)	122.2(5)
O(8)-C(65)-C(62)	124.9(5)
O(7)-C(65)-C(62)	112.8(4)
C(72)-C(67)-C(68)	117.7(5)
C(72)-C(67)-C(58)	119.7(5)
C(68)-C(67)-C(58)	122.5(5)
C(69)-C(68)-C(67)	121.8(5)
C(70)-C(69)-C(68)	118.3(6)
C(71)-C(70)-F(4)	118.9(5)
C(71)-C(70)-C(69)	123.0(6)
F(4)-C(70)-C(69)	118.2(5)
C(70)-C(71)-C(72)	118.1(5)
C(67)-C(72)-C(71)	121.0(5)
C(74)-C(73)-Si(4)	89.7(10)
C(74A)-C(73)-Si(4)	85.3(6)
C(73)-C(74)-C(75)	103.2(12)
C(73)-C(74)-Si(4)	52.9(7)
C(75)-C(74)-Si(4)	53.1(6)
C(75)-C(74A)-C(73)	102.2(9)
C(75)-C(74A)-Si(4)	52.9(5)
C(73)-C(74A)-Si(4)	51.8(5)
C(74A)-C(75)-Si(4)	88.5(6)
C(74)-C(75)-Si(4)	82.4(10)

Table S13. Anisotropic displacement parameters ($Å^2 x 10^3$) for cu-5883. The anisotropic displacement factor exponent takes the form: $-2\pi^2[h^2 a^{*2}U^{11} + ... + 2 h k a^* b^* U^{12}]$

	U^{11}	U^{22}	U^{33}	U^{23}	U^{13}	U^{12}
Si(1)	27(1)	52(1)	30(1)	4(1)	5(1)	3(1)
Si(2)	35(1)	41(1)	41(1)	4(1)	7(1)	0(1)
Si(3)	26(1)	56(1)	32(1)	4(1)	4(1)	-6(1)
F(1)	48(2)	65(3)	89(3)	-18(2)	23(2)	7(2)
F(2)	68(3)	51(2)	64(2)	-13(2)	8(2)	-22(2)
F(3)	48(2)	55(2)	82(3)	-4(2)	16(2)	5(2)
O(1)	62(3)	50(3)	78(3)	-16(3)	0(3)	-3(2)
O(2)	72(3)	72(4)	62(3)	-7(3)	-12(3)	-21(3)
O(3)	55(3)	40(2)	52(3)	0(2)	21(2)	7(2)
O(4)	42(2)	65(3)	44(2)	-1(2)	19(2)	13(2)
O(5)	66(3)	62(3)	65(3)	-15(3)	7(2)	-18(3)
O(6)	93(4)	53(3)	95(4)	4(3)	-14(3)	-1(3)
C(1)	31(3)	54(4)	20(2)	2(2)	6(2)	1(2)
C(2)	31(3)	50(3)	23(2)	4(2)	7(2)	-3(2)
C(3)	26(3)	57(4)	33(3)	3(3)	8(2)	1(2)
C(4)	31(3)	63(4)	29(3)	5(3)	8(2)	1(3)
C(5)	37(3)	55(4)	24(3)	-4(2)	12(2)	-9(3)
C(6)	40(3)	49(4)	47(3)	5(3)	5(3)	7(3)
C(7)	32(3)	54(4)	34(3)	4(3)	2(2)	-4(3)
C(8)	49(3)	63(4)	39(3)	0(3)	15(3)	-5(3)
C(9)	109(8)	56(5)	116(8)	-16(6)	12(6)	11(5)
C(10)	31(3)	49(3)	27(3)	0(2)	10(2)	-2(2)
C(11)	39(3)	70(4)	35(3)	0(3)	6(2)	13(3)
C(12)	38(3)	65(4)	53(4)	2(3)	6(3)	15(3)
C(13)	32(3)	51(4)	62(4)	-9(3)	14(3)	0(3)
C(14)	39(3)	58(4)	44(3)	-11(3)	18(3)	-12(3)
C(15)	37(3)	45(3)	36(3)	0(3)	8(2)	-7(2)
C(16)	35(3)	94(6)	30(3)	10(3)	6(2)	-6(3)
C(17)	42(3)	76(5)	42(3)	-5(3)	26(3)	-8(3)
C(18)	34(3)	57(4)	50(3)	-3(3)	11(3)	0(3)
C(19)	48(4)	61(4)	60(4)	-3(4)	15(3)	-2(3)
C(20)	38(3)	38(3)	27(2)	1(2)	7(2)	0(2)

C(21)	30(3)	35(3)	27(2)	3(2)	5(2)	0(2)
C(22)	35(3)	38(3)	33(3)	4(2)	13(2)	-3(2)
C(23)	33(3)	38(3)	32(3)	5(2)	5(2)	-1(2)
C(24)	29(3)	42(3)	26(3)	5(2)	2(2)	1(2)
C(25)	29(3)	50(3)	26(2)	3(2)	6(2)	-2(2)
C(26)	27(3)	43(3)	30(3)	4(2)	8(2)	-6(2)
C(27)	28(3)	45(3)	35(3)	-1(2)	-1(2)	4(2)
C(28)	75(5)	46(4)	69(5)	-8(4)	15(4)	15(4)
C(29)	41(3)	42(3)	29(3)	5(2)	8(2)	3(3)
C(30)	51(3)	42(3)	32(3)	-2(3)	8(2)	-1(3)
C(31)	54(4)	56(4)	37(3)	-9(3)	4(3)	-4(3)
C(32)	44(3)	39(3)	54(4)	-10(3)	10(3)	-12(3)
C(33)	54(4)	50(4)	40(3)	2(3)	5(3)	-4(3)
C(34)	53(3)	45(3)	30(3)	3(3)	3(3)	-10(3)
C(35)	34(3)	51(4)	69(4)	3(3)	-4(3)	-2(3)
C(36)	53(4)	61(5)	58(4)	-14(4)	-3(3)	-4(3)
C(37)	46(3)	65(4)	44(3)	4(3)	-2(3)	-4(3)
C(38)	57(4)	53(4)	75(5)	6(4)	12(4)	15(3)
C(39)	28(3)	56(4)	21(2)	2(2)	4(2)	1(2)
C(40)	31(3)	51(4)	21(2)	1(2)	7(2)	-12(2)
C(41)	32(3)	58(4)	32(3)	5(3)	6(2)	-5(3)
C(42)	34(3)	72(4)	29(3)	-2(3)	8(2)	-19(3)
C(43)	43(3)	52(4)	39(3)	3(3)	11(3)	-11(3)
C(44)	51(4)	48(4)	48(4)	3(3)	1(3)	-4(3)
C(45)	37(3)	61(4)	40(3)	9(3)	0(2)	-2(3)
C(46)	58(4)	62(5)	44(4)	-13(3)	15(3)	-11(4)
C(47)	82(6)	72(6)	113(7)	-27(6)	7(5)	-22(5)
C(48)	27(2)	49(3)	29(3)	7(2)	3(2)	-7(2)
C(49)	33(3)	56(4)	36(3)	4(3)	4(2)	-1(3)
C(50)	30(3)	49(4)	48(3)	8(3)	3(2)	-3(2)
C(51)	35(3)	40(3)	60(4)	-2(3)	14(3)	-4(3)
C(52)	44(3)	52(4)	40(3)	-2(3)	14(3)	-9(3)
C(53)	34(3)	48(4)	34(3)	3(3)	7(2)	-7(2)
C(54)	37(3)	92(5)	31(3)	12(3)	3(2)	-18(3)
C(55)	36(3)	90(6)	39(3)	-6(3)	21(3)	-14(3)
C(56)	33(3)	62(4)	46(3)	-1(3)	6(2)	-4(3)
C(57)	35(3)	61(4)	71(5)	0(4)	8(3)	-12(3)
Si(4)	31(1)	48(1)	47(1)	-1(1)	10(1)	-3(1)

F(4)	79(3)	66(3)	57(2)	-13(2)	10(2)	-38(2)
O(7)	53(3)	38(2)	46(2)	-4(2)	19(2)	3(2)
O(8)	39(2)	61(3)	43(2)	-6(2)	18(2)	4(2)
C(58)	33(3)	41(3)	31(3)	-2(2)	10(2)	-5(2)
C(59)	27(2)	37(3)	29(2)	2(2)	8(2)	-5(2)
C(60)	28(3)	36(3)	35(3)	3(2)	8(2)	-8(2)
C(61)	28(3)	45(3)	31(3)	-1(2)	9(2)	-3(2)
C(62)	26(2)	41(3)	24(2)	0(2)	4(2)	-4(2)
C(63)	41(3)	39(3)	29(3)	3(2)	9(2)	-2(2)
C(64)	35(3)	40(3)	30(3)	0(2)	16(2)	-6(2)
C(65)	28(2)	45(3)	33(3)	0(2)	3(2)	0(2)
C(66)	70(5)	47(4)	71(5)	-16(4)	14(4)	10(4)
C(67)	30(3)	40(3)	36(3)	1(2)	8(2)	-1(2)
C(68)	47(3)	44(3)	31(3)	6(2)	3(2)	-8(3)
C(69)	45(3)	42(3)	46(3)	7(3)	1(3)	-4(3)
C(70)	42(3)	45(3)	44(3)	-5(3)	8(3)	-11(3)
C(71)	63(4)	59(4)	32(3)	-3(3)	-1(3)	-21(3)
C(72)	50(3)	49(4)	32(3)	4(3)	11(2)	-6(3)
C(73)	33(3)	58(4)	79(5)	-8(4)	1(3)	-5(3)
C(74)	43(11)	97(13)	61(9)	-22(9)	-32(10)	-15(10)
C(74A)	44(8)	93(10)	59(6)	-13(6)	-9(7)	-15(7)
C(75)	43(3)	74(5)	51(4)	5(4)	4(3)	3(4)
C(76)	49(4)	51(4)	81(5)	2(4)	0(4)	10(3)

8.4 Crystal Data of (*R*)-5da

Methyl (*R*)-4-((4-(trifluoromethyl)phenyl)(trimethylgermyl)methyl)benzoate (**5da**)

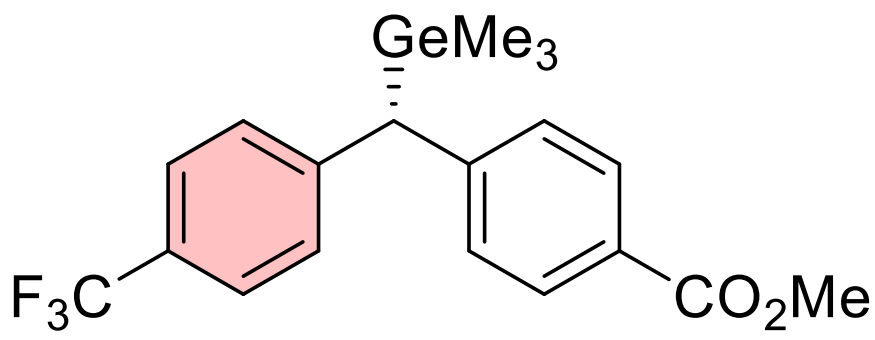

5da

$C_{19}H_{21}F_3GeO_2$

M_W = 411.00

Suitable crystals for X-ray analysis were grown by slow evaporation of a solution of the sample in *n*-pentane. The absolute configuration of (*R*)-**5da** was confirmed unambiguously by X-ray diffraction analysis, and other compounds were assigned by analogy. CCDC 2563297 contains the supplementary crystallographic data for this compound. Comparison of the HPLC traces of the measured crystal can be found below under Figure S43.

Figure S14. Molecular structure of (*R*)-**5da**.

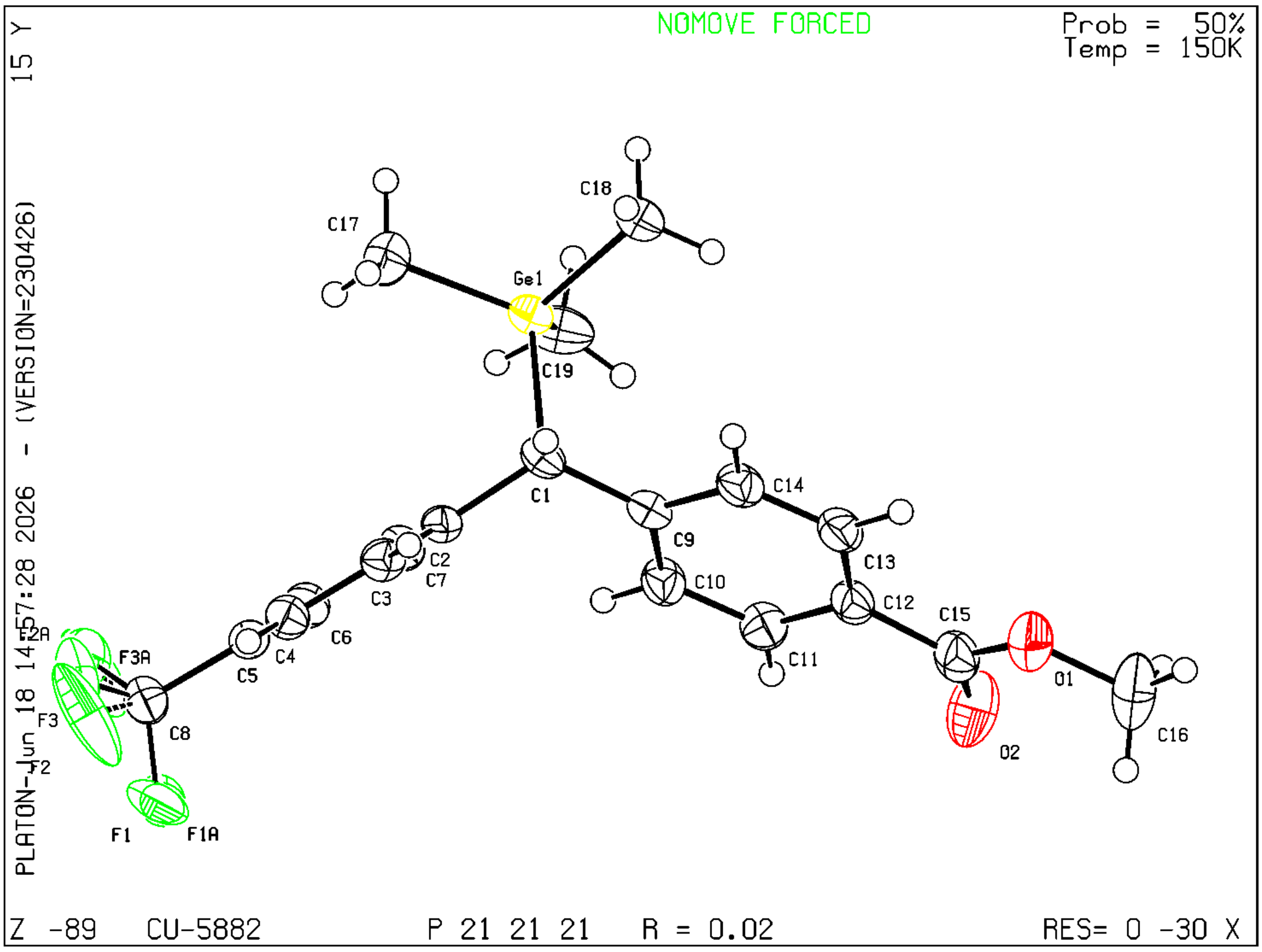

Table S14. Crystal data and structure refinement for cu-5882.

Identification code	CU-5882	
Empirical formula	$C_{19}H_{21}F_3GeO_2$	
Formula weight	410.95	
Temperature	150.01(10) K	
Wavelength	1.54184 Å	
Crystal system	Orthorhombic	
Space group	$P2_12_12_1$ (No. 19)	
Unit cell dimensions	a = 8.43450(10) Å	a= 90°.
	b = 10.92890(10) Å	b= 90°.
	c = 20.5813(3) Å	g = 90°.
Volume	1897.18(4) $Å^3$	
Z	4	
Density (calculated)	1.439 Mg/m^3	
Absorption coefficient	2.533 mm^{-1}	
F(000)	840	
Crystal size	0.329 x 0.168 x 0.098 mm^3	
Theta range for data collection	4.296 to 72.390°.	
Index ranges	-9<=h<=10, -11<=k<=13, -18<=l<=25	
Reflections collected	7909	
Independent reflections	3583 [R(int) = 0.0173]	
Completeness to theta = 67.684°	100.0 %	
Absorption correction	Semi-empirical from equivalents	
Max. and min. transmission	1.00000 and 0.45772	
Refinement method	Full-matrix least-squares on F^2	
Data / restraints / parameters	3583 / 36 / 258	
Goodness-of-fit on F^2	1.071	
Final R indices [I>2sigma(I)]	R1 = 0.0220, wR2 = 0.0556	
R indices (all data)	R1 = 0.0232, wR2 = 0.0566	
Absolute structure parameter	-0.007(12)	
Extinction coefficient	n/a	
Largest diff. peak and hole	0.192 and -0.250 e.$Å^{-3}$	

Table S15. Atomic coordinates (x 10^4) and equivalent isotropic displacement parameters ($Å^2$x 10^3) for cu-5882. U(eq) is defined as one third of the trace of the orthogonalized U^{ij} tensor.

	x	y	z	U(eq)
Ge(1)	5775(1)	7241(1)	3551(1)	29(1)
F(1)	-1282(19)	11390(20)	3006(16)	93(7)
F(2)	400(40)	12688(15)	3287(9)	94(6)
F(3)	320(30)	11607(14)	2375(5)	79(4)
F(1A)	-1160(18)	11636(19)	3190(10)	96(5)
F(2A)	880(20)	12708(11)	3183(7)	79(3)
F(3A)	595(16)	11742(11)	2398(5)	71(3)
O(1)	1467(3)	3083(2)	5796(1)	51(1)
O(2)	-109(3)	2875(2)	4928(1)	60(1)
C(1)	4039(3)	7723(2)	4166(1)	28(1)
C(2)	3011(3)	8713(2)	3870(1)	26(1)
C(3)	2830(3)	9822(2)	4187(1)	30(1)
C(4)	1948(4)	10770(3)	3918(1)	32(1)
C(5)	1229(3)	10609(3)	3317(1)	31(1)
C(6)	1383(3)	9507(3)	2996(1)	33(1)
C(7)	2264(3)	8569(2)	3264(1)	32(1)
C(8)	288(4)	11619(3)	3016(2)	41(1)
C(9)	3219(3)	6601(2)	4425(1)	28(1)
C(10)	1874(4)	6090(3)	4138(1)	35(1)
C(11)	1178(4)	5042(3)	4388(1)	35(1)
C(12)	1784(3)	4488(3)	4942(1)	32(1)
C(13)	3131(4)	4982(3)	5232(1)	36(1)
C(14)	3829(3)	6020(3)	4976(1)	34(1)
C(15)	938(4)	3399(2)	5202(1)	39(1)
C(16)	708(6)	2027(3)	6079(2)	68(1)
C(17)	6648(4)	8739(3)	3189(2)	49(1)
C(18)	7380(4)	6364(3)	4044(2)	38(1)
C(19)	4974(4)	6134(3)	2885(2)	48(1)

Table S16. Bond lengths [Å] and angles [°] for cu-5882.

Ge(1)-C(17)	1.943(3)	C(15)-O(1)-C(16)	115.2(3)
Ge(1)-C(18)	1.945(3)	C(9)-C(1)-C(2)	117.4(2)
Ge(1)-C(19)	1.948(3)	C(9)-C(1)-Ge(1)	110.22(17)
Ge(1)-C(1)	2.007(2)	C(2)-C(1)-Ge(1)	110.54(16)
F(1)-C(8)	1.349(16)	C(3)-C(2)-C(7)	117.7(2)
F(2)-C(8)	1.298(14)	C(3)-C(2)-C(1)	119.9(2)
F(3)-C(8)	1.320(11)	C(7)-C(2)-C(1)	122.3(2)
F(1A)-C(8)	1.273(14)	C(2)-C(3)-C(4)	121.6(2)
F(2A)-C(8)	1.336(13)	C(5)-C(4)-C(3)	119.6(3)
F(3A)-C(8)	1.306(10)	C(6)-C(5)-C(4)	119.8(3)
O(1)-C(15)	1.344(4)	C(6)-C(5)-C(8)	119.7(3)
O(1)-C(16)	1.443(4)	C(4)-C(5)-C(8)	120.5(3)
O(2)-C(15)	1.195(4)	C(5)-C(6)-C(7)	120.4(2)
C(1)-C(9)	1.505(4)	C(6)-C(7)-C(2)	120.9(3)
C(1)-C(2)	1.515(3)	F(1A)-C(8)-F(3A)	117.5(12)
C(2)-C(3)	1.386(4)	F(2)-C(8)-F(3)	115.9(14)
C(2)-C(7)	1.406(4)	F(1A)-C(8)-F(2A)	106.0(11)
C(3)-C(4)	1.390(4)	F(3A)-C(8)-F(2A)	94.9(10)
C(4)-C(5)	1.389(4)	F(2)-C(8)-F(1)	104.2(14)
C(5)-C(6)	1.381(4)	F(3)-C(8)-F(1)	90(2)
C(5)-C(8)	1.494(4)	F(1A)-C(8)-C(5)	113.8(8)
C(6)-C(7)	1.382(4)	F(2)-C(8)-C(5)	116.7(7)
C(9)-C(10)	1.396(4)	F(3A)-C(8)-C(5)	112.1(6)
C(9)-C(14)	1.397(4)	F(3)-C(8)-C(5)	113.5(7)
C(10)-C(11)	1.387(4)	F(2A)-C(8)-C(5)	110.6(7)
C(11)-C(12)	1.388(4)	F(1)-C(8)-C(5)	113.0(9)
C(12)-C(13)	1.392(4)	C(10)-C(9)-C(14)	117.5(3)
C(12)-C(15)	1.488(4)	C(10)-C(9)-C(1)	123.3(2)
C(13)-C(14)	1.383(4)	C(14)-C(9)-C(1)	119.2(2)
		C(11)-C(10)-C(9)	121.1(3)
C(17)-Ge(1)-C(18)	110.57(15)	C(10)-C(11)-C(12)	120.7(3)
C(17)-Ge(1)-C(19)	112.68(16)	C(11)-C(12)-C(13)	118.9(3)
C(18)-Ge(1)-C(19)	107.58(15)	C(11)-C(12)-C(15)	117.9(3)
C(17)-Ge(1)-C(1)	107.31(13)	C(13)-C(12)-C(15)	123.2(3)
C(18)-Ge(1)-C(1)	107.90(11)	C(14)-C(13)-C(12)	120.2(3)
C(19)-Ge(1)-C(1)	110.72(12)	C(13)-C(14)-C(9)	121.7(3)

O(2)-C(15)-O(1)	123.5(3)	O(1)-C(15)-C(12)	111.9(3)
O(2)-C(15)-C(12)	124.6(3)		

Table S17. Anisotropic displacement parameters ($Å^2$x 10^3) for cu-5882. The anisotropic displacement factor exponent takes the form: $-2\pi^2[h^2 a^{*2}U^{11} + ... + 2 h k a^* b^* U^{12}]$

	U^{11}	U^{22}	U^{33}	U^{23}	U^{13}	U^{12}
Ge(1)	24(1)	32(1)	31(1)	0(1)	-2(1)	3(1)
F(1)	25(5)	83(7)	169(17)	77(9)	-11(7)	-1(5)
F(2)	164(15)	46(7)	72(6)	-30(5)	-62(8)	60(8)
F(3)	132(11)	72(6)	33(5)	-14(5)	-30(6)	61(6)
F(1A)	55(7)	118(10)	116(7)	65(6)	39(6)	58(7)
F(2A)	107(7)	35(4)	96(5)	22(4)	-12(4)	10(4)
F(3A)	67(4)	86(5)	60(5)	46(4)	22(4)	28(4)
O(1)	56(1)	45(1)	52(1)	17(1)	0(1)	0(1)
O(2)	87(2)	41(1)	51(1)	1(1)	-6(1)	-23(1)
C(1)	25(1)	30(1)	28(1)	-3(1)	-7(1)	4(1)
C(2)	21(1)	27(1)	30(1)	1(1)	-1(1)	0(1)
C(3)	31(1)	33(1)	26(1)	-1(1)	2(1)	1(1)
C(4)	35(1)	29(1)	34(1)	-1(1)	5(1)	3(1)
C(5)	25(1)	32(1)	34(1)	7(1)	4(1)	2(1)
C(6)	29(1)	37(1)	33(1)	3(1)	-6(1)	0(1)
C(7)	30(1)	30(1)	35(1)	-3(1)	-6(1)	0(1)
C(8)	41(2)	39(2)	43(2)	11(1)	6(1)	10(1)
C(9)	24(1)	30(1)	31(1)	-3(1)	-2(1)	6(1)
C(10)	34(1)	35(1)	35(1)	4(1)	-7(1)	0(1)
C(11)	34(2)	34(1)	38(1)	-1(1)	-5(1)	-2(1)
C(12)	35(2)	28(1)	34(1)	-1(1)	4(1)	6(1)
C(13)	33(1)	40(1)	34(1)	6(1)	0(1)	8(1)
C(14)	29(1)	39(1)	33(1)	2(1)	-5(1)	4(1)
C(15)	49(2)	28(1)	40(1)	-1(1)	8(1)	9(1)
C(16)	97(3)	44(2)	64(2)	22(2)	4(2)	-5(2)
C(17)	51(2)	44(2)	51(2)	13(2)	10(2)	2(2)
C(18)	29(1)	41(2)	45(2)	5(1)	-2(1)	6(1)
C(19)	38(2)	62(2)	45(2)	-23(2)	-3(1)	8(2)

9. HPLC Traces

Methyl (*S*)-4-((1-methylsiletan-1-yl)(phenyl)methyl)benzoate (3aa)

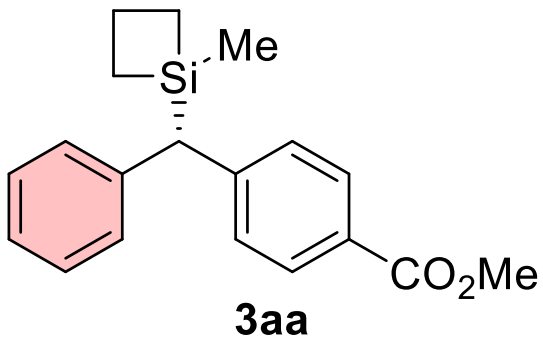

3aa
$C_{19}H_{22}O_2Si$
M_W = 310.47

The enantiomeric ratio of **3aa** was determined by HPLC analysis on a chiral stationary phase (*Daicel Chiralcel* OD-H column, column temperature 20 °C, solvent *n*-heptane:isopropanol = 99.9:0.1, flow rate 1.2 mL/min): t_R = 25.9 min (major), t_R = 33.2 min (minor). The minor enantiomer was not found in the HPLC trace of the enantioenriched sample for this compound.

Figure S15. Methyl 4-((1-methylsiletan-1-yl)(phenyl)methyl)benzoate (*rac*-**3aa**).

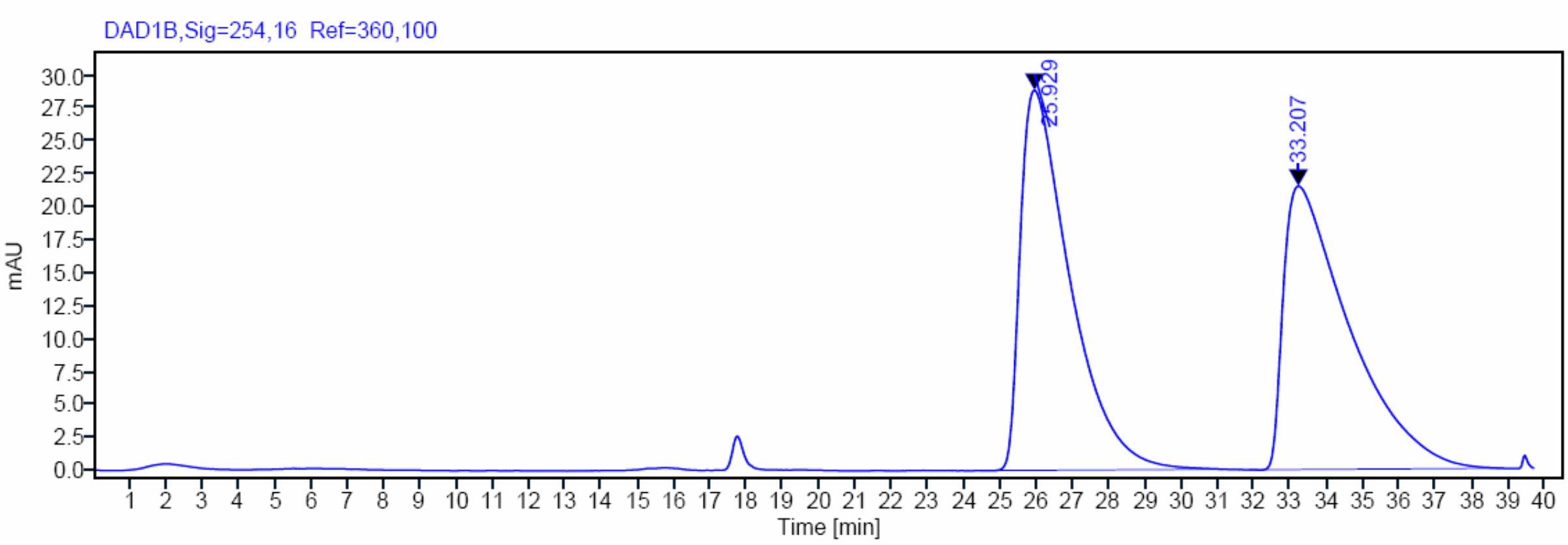

Signal: DAD1B,Sig=254,16 Ref=360,100

RT [min]	Width [min]	Area	Height	Area%
25.929	6.72	2748.76	28.88	50.31
33.207	6.99	2714.58	21.53	49.69
	**Sum**	**5463.34**		

Figure S16. Methyl (*S*)-4-((1-methylsiletan-1-yl)(phenyl)methyl)benzoate [(*S*)-**3aa**].

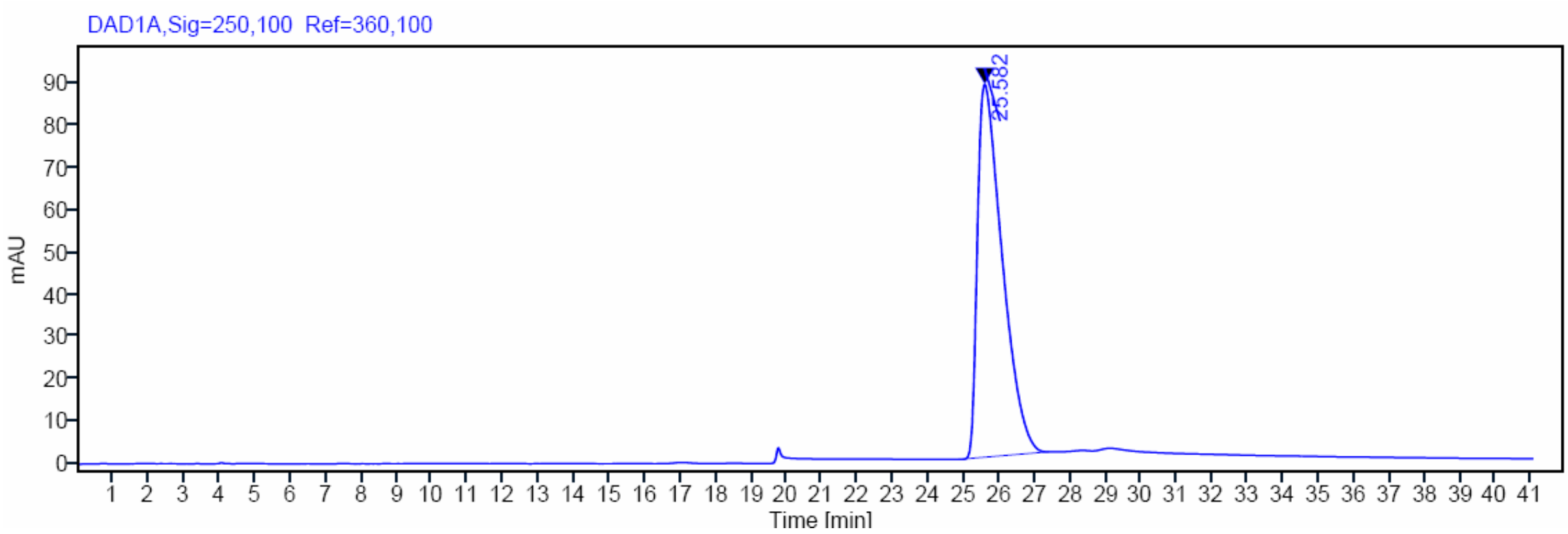

Signal: DAD1A,Sig=250,100 Ref=360,100

RT [min]	Width [min]	Area	Height	Area%
25.582	2.58	4280.51	88.20	100.00
	**Sum**	**4280.51**		

Figure S17. Measured crystal of Methyl (*S*)-4-((1-methylsiletan-1-yl)(phenyl)methyl)-benzoate [(*S*)-**3aa**].

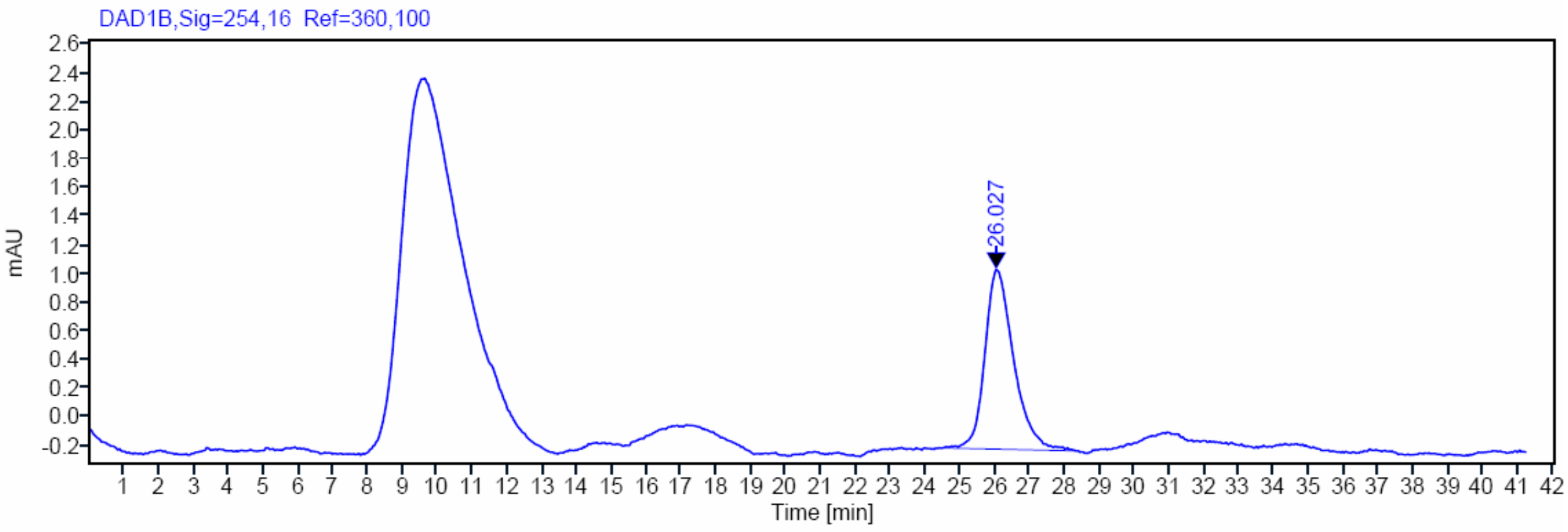

Methyl (*S*)-4-((1-methylsiletan-1-yl)(*p*-tolyl)methyl)benzoate (**3ba**)

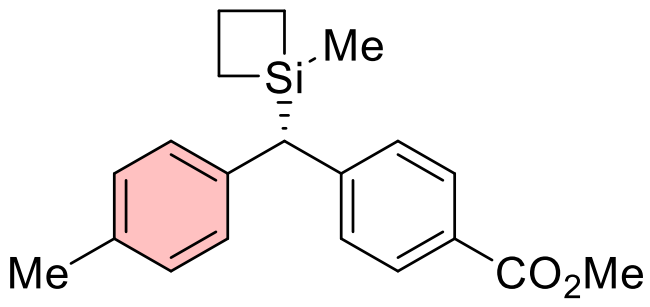

3ba
$C_{20}H_{24}O_2Si$
M_W = 324.50

The enantiomeric ratio of **3ba** was determined by HPLC analysis on a chiral stationary phase (*Daicel Chiralcel* OD-H column, column temperature 20 °C, solvent *n*-heptane:isopropanol = 100:0, flow rate 0.6 mL/min): t_R = 45.8 min (major), t_R = 51.2 min (minor).

Figure S18. Methyl 4-((1-methylsiletan-1-yl)(*p*-tolyl)methyl)benzoate (*rac*-**3ba**).

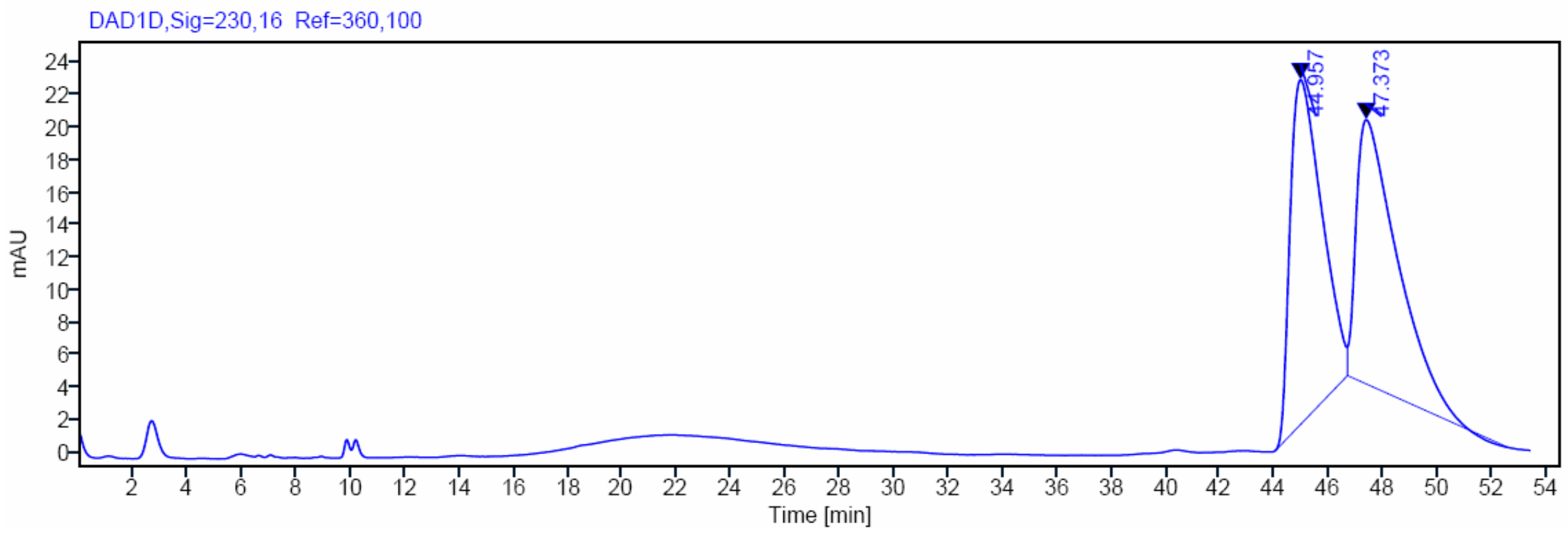

Signal: DAD1C,Sig=210,8 Ref=360,100

RT [min]	Width [min]	Area	Height	Area%
44.956	2.64	3110.29	38.65	49.35
47.374	5.03	3192.21	29.53	50.65
	**Sum**	**6302.50**		

Figure S19. Methyl (*S*)-4-((1-methylsiletan-1-yl)(*p*-tolyl)methyl)benzoate [(*S*)-**3ba**].

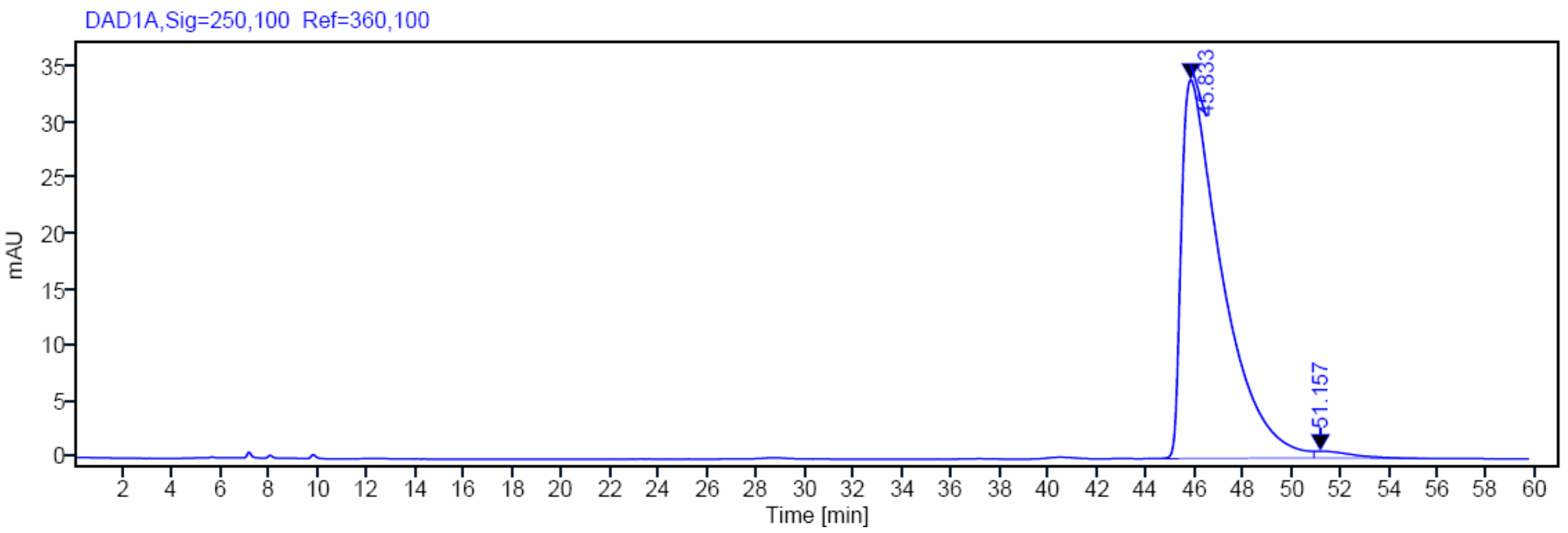

Signal: DAD1A,Sig=250,100 Ref=360,100

RT [min]	Width [min]	Area	Height	Area%
45.833	6.95	3943.91	34.00	98.40
51.157	5.25	64.32	0.61	1.60
	**Sum**	**4008.23**		

The crystal that was measured by X-ray analysis was also measured by HPLC under the same conditions. Since retention times shifted, a new measurement of the racemic compound is also included:

Figure S20. Methyl 4-((1-methylsiletan-1-yl)(*p*-tolyl)methyl)benzoate (*rac*-**3ba**).

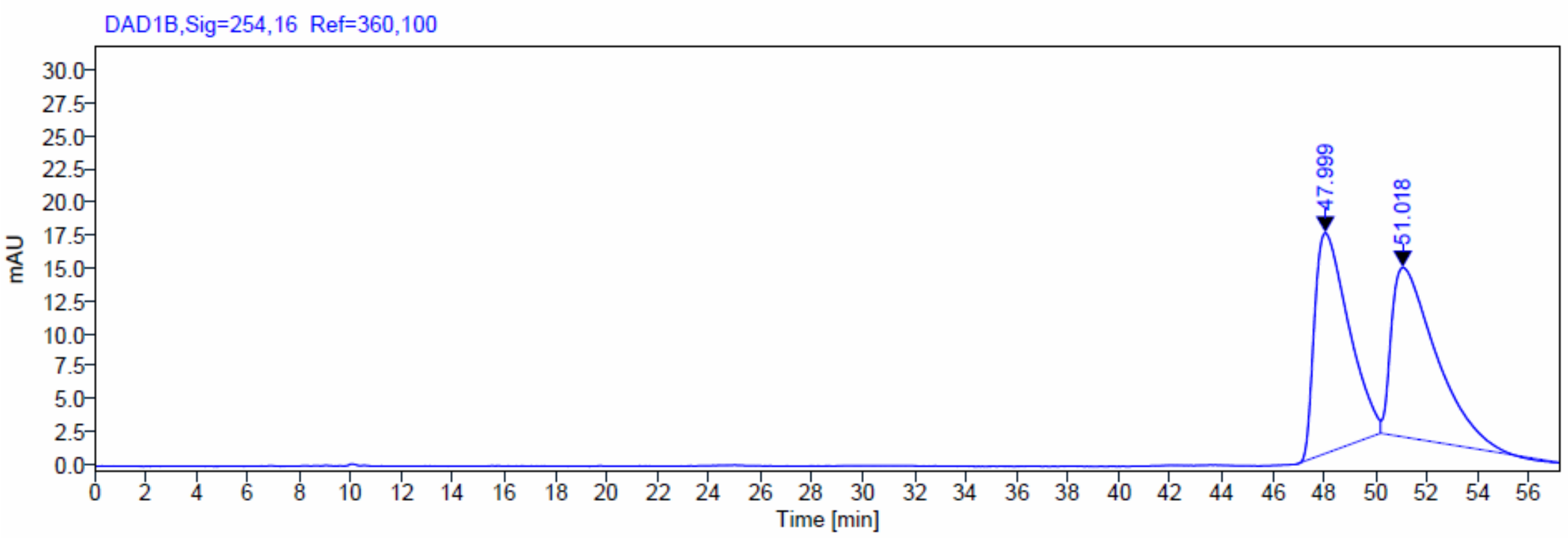

Signal:	DAD1B,Sig=254,16 Ref=360,100			
RT [min]	Width [min]	Area	Height	Area%
47.999	3.30	1545.14	16.75	49.65
51.018	7.07	1567.17	12.85	50.35
	**Sum**	**3112.32**		

Figure S21. Measured crystal of methyl (*S*)-4-((1-methylsiletan-1-yl)(*p*-tolyl)methyl)benzoate [(*S*)-**3ba**].

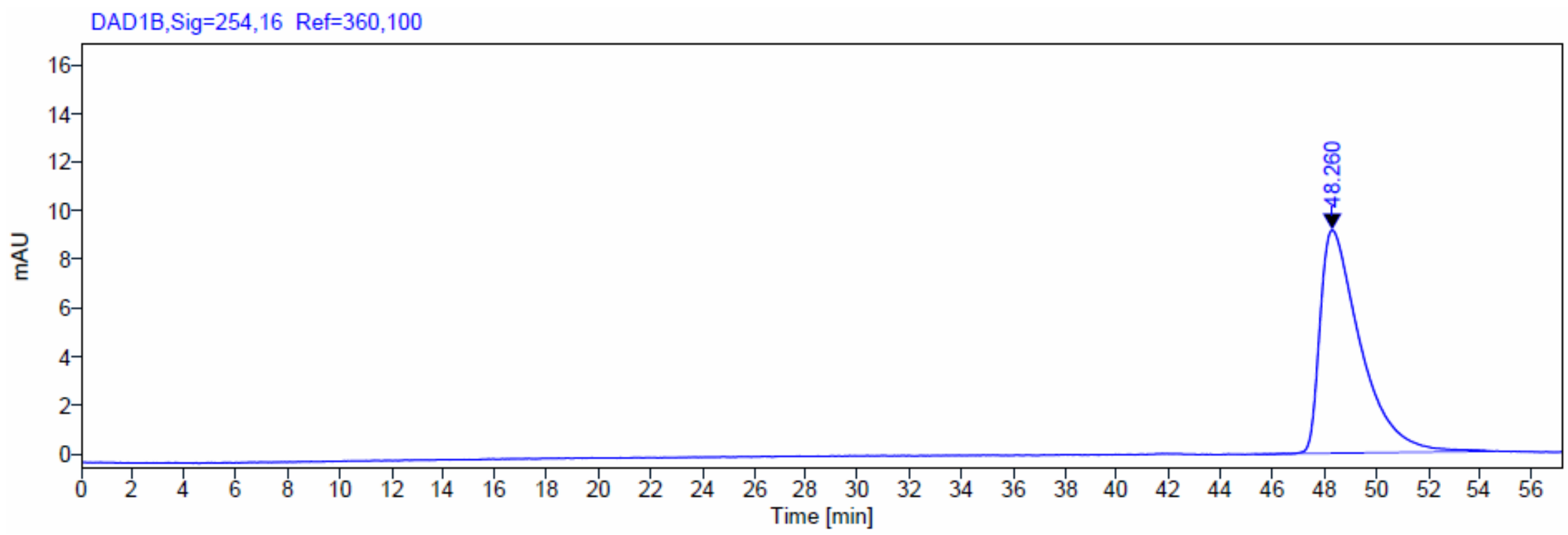

Signal:	DAD1B,Sig=254,16 Ref=360,100			
RT [min]	Width [min]	Area	Height	Area%
48.260	9.22	992.43	9.18	100.00
	**Sum**	**992.43**		

The retention time of the measured crystal corresponds to the same major enantiomer obtained in the cross-electrophile coupling reaction.

Methyl (*R*)-4-((1-methylsiletan-1-yl)(*m*-tolyl)methyl)benzoate (3ca)

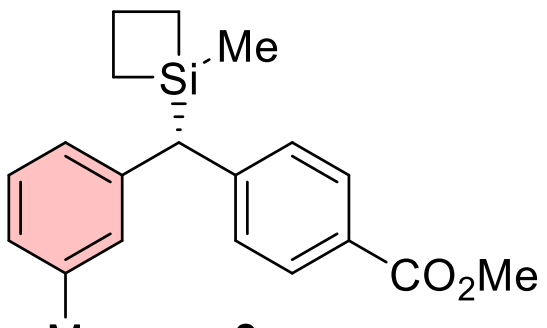

3ca
$C_{20}H_{24}O_2Si$
M_W = 324.50

The enantiomeric ratio of **3ca** was determined by HPLC analysis on a chiral stationary phase (*Daicel Chiralpak* AD-H column, column temperature 20 °C, solvent *n*-heptane:isopropanol = 99.9:0.1, flow rate 1.1 mL/min): t_R = 14.9 min (minor), t_R = 17.6 min (major).

Figure S22. Methyl 4-((1-methylsiletan-1-yl)(*m*-tolyl)methyl)benzoate (*rac*-**3ca**).

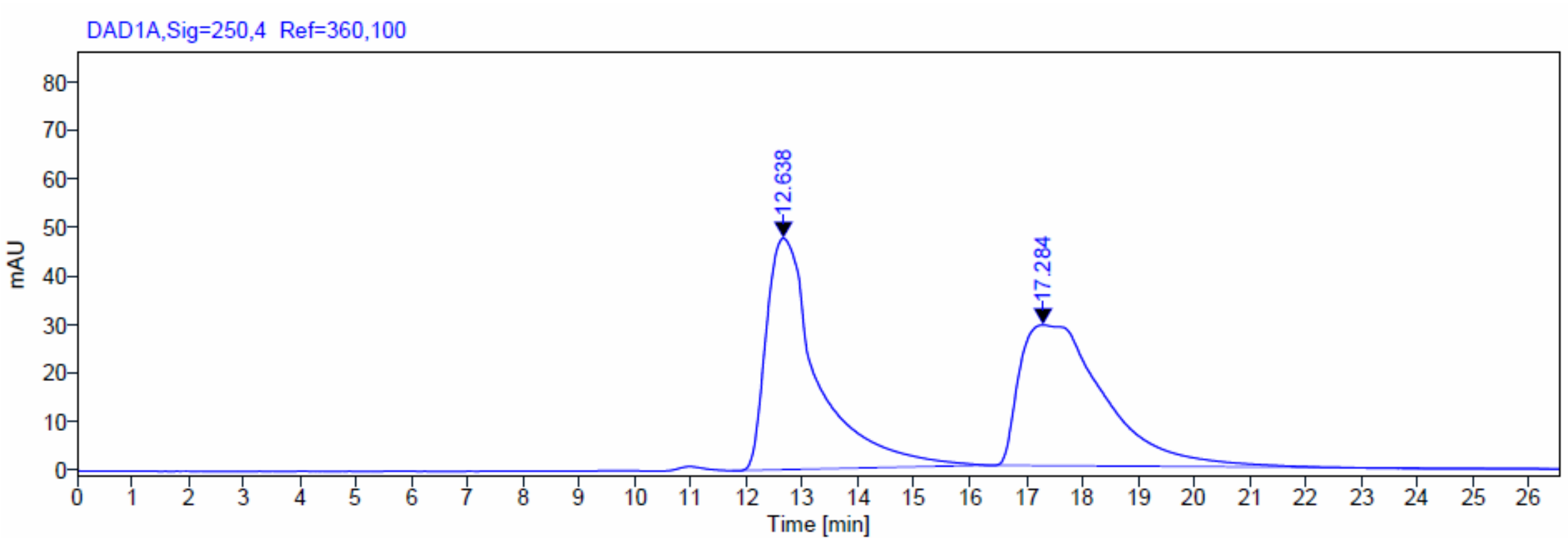

Signal: DAD1A,Sig=250,4 Ref=360,100

RT [min]	Width [min]	Area	Height	Area%
12.638	4.54	3064.33	47.71	50.06
17.284	10.39	3057.15	29.00	49.94
	**Sum**	**6121.48**		

Figure S23. Methyl (*R*)-4-((1-methylsiletan-1-yl)(*m*-tolyl)methyl)benzoate [(*R*)-**3ca**].

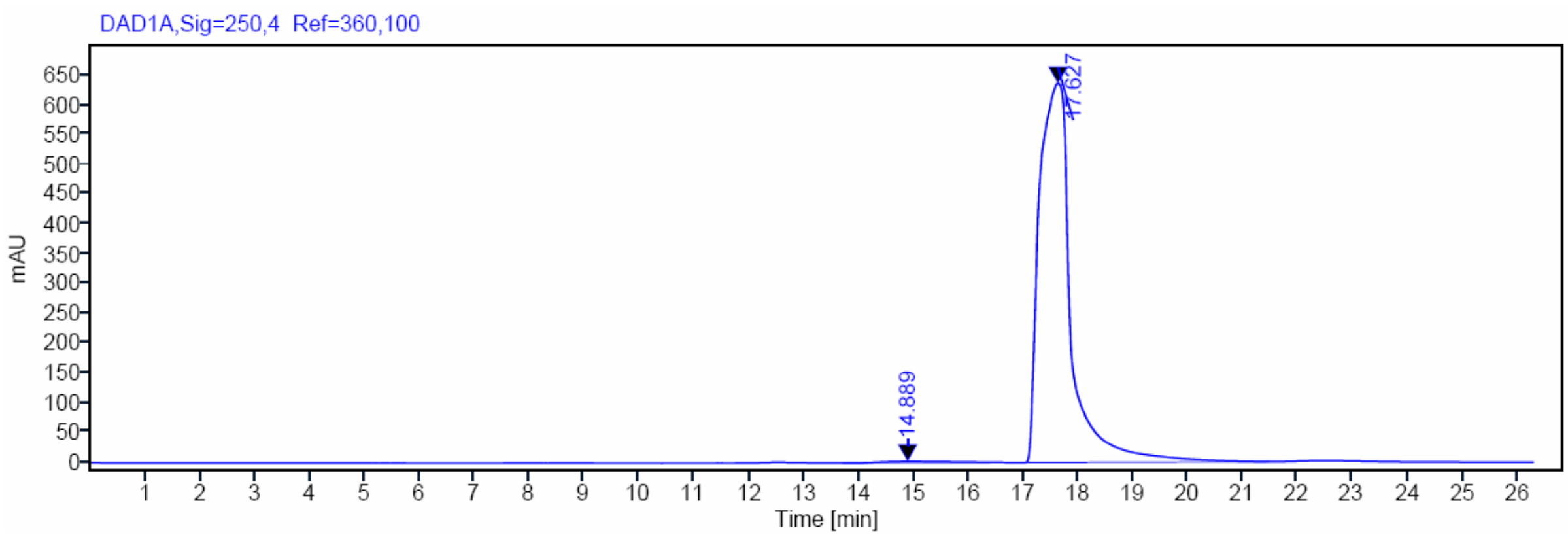

Signal: DAD1A,Sig=250,4 Ref=360,100

RT [min]	Width [min]	Area	Height	Area%
14.889	3.20	238.52	2.56	0.92
17.627	4.58	25703.16	636.72	99.08
	**Sum**	**25941.68**		

Methyl (*R*)-4-((4-fluorophenyl)(1-methylsiletan-1-yl)methyl)benzoate (3ea)

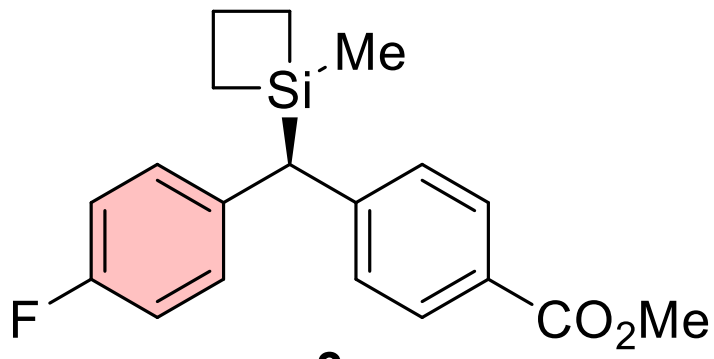

3ea

$C_{19}H_{21}FO_2Si$

M_W = 328.46

The enantiomeric ratio of **3ea** was determined by HPLC analysis on a chiral stationary phase (*Daicel Chiralpak* AD-H column, column temperature 20 °C, solvent *n*-heptane:isopropanol = 99.9:0.1, flow rate 1.1 mL/min): t_R = 26.6 min (minor), t_R = 33.3 min (major).

Figure S24. Methyl 4-((4-fluorophenyl)(1-methylsiletan-1-yl)methyl)benzoate (*rac*-**3ea**).

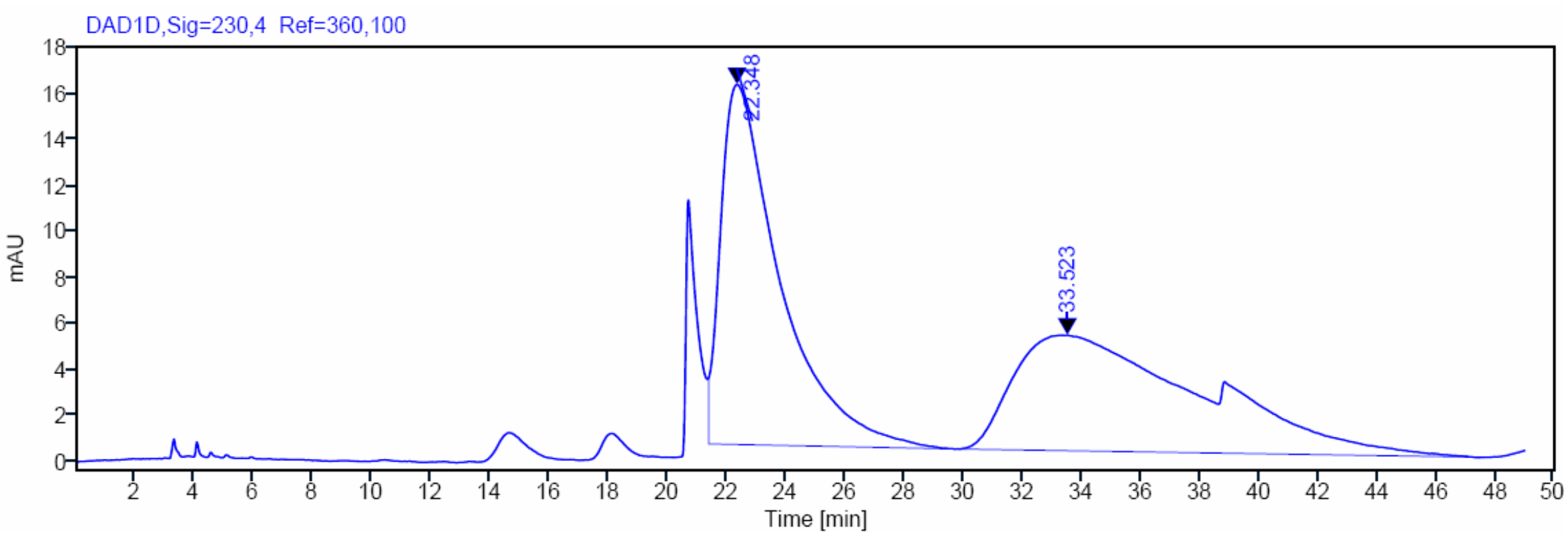

Signal: DAD1D,Sig=230,4 Ref=360,100

RT [min]	Width [min]	Area	Height	Area%
22.348	8.37	2191.59	15.69	49.54
33.523	17.79	2232.53	5.04	50.46
	**Sum**	**4424.13**		

Figure S25. Methyl (*R*)-4-((4-fluorophenyl)(1-methylsiletan-1-yl)methyl)benzoate [(*R*)-**3ea**].

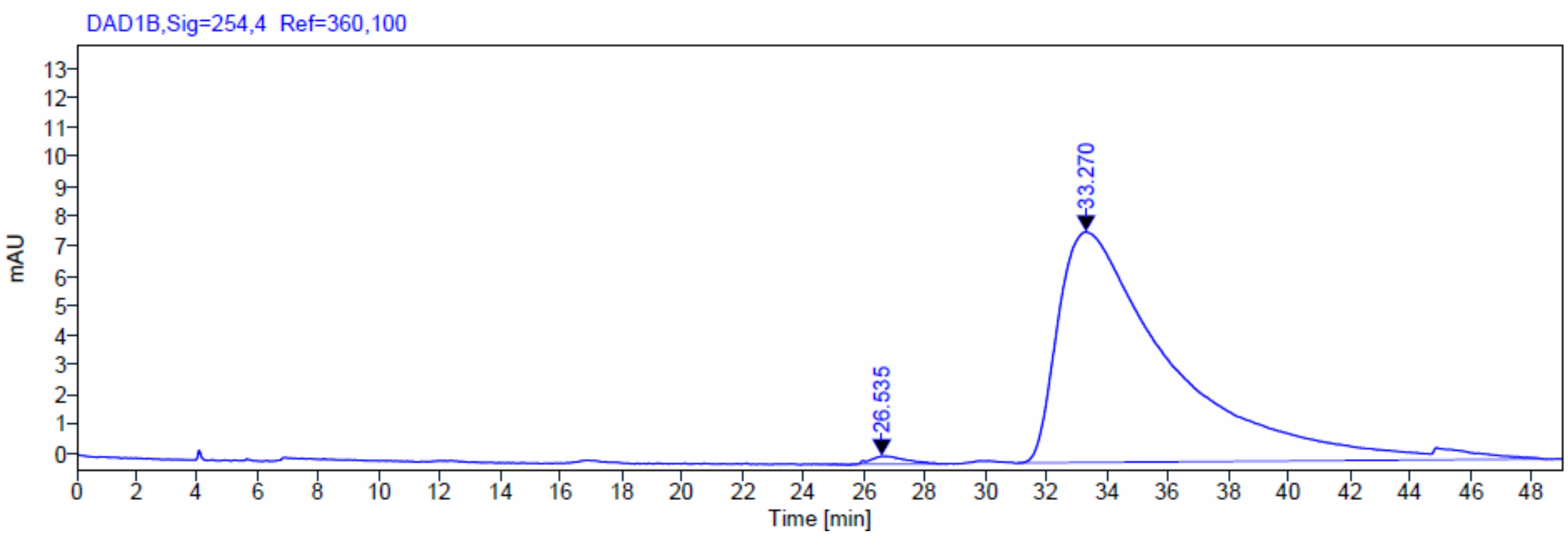

Signal: DAD1B,Sig=254,4 Ref=360,100

RT [min]	Width [min]	Area	Height	Area%
26.535	3.77	21.24	0.27	1.04
33.270	17.33	2026.49	7.78	98.96
	**Sum**	**2047.72**		

Figure S26. Measured crystal of methyl (*R*)-4-((4-fluorophenyl)(1-methylsiletan-1-yl)methyl)benzoate [(*R*)-**3ea**].

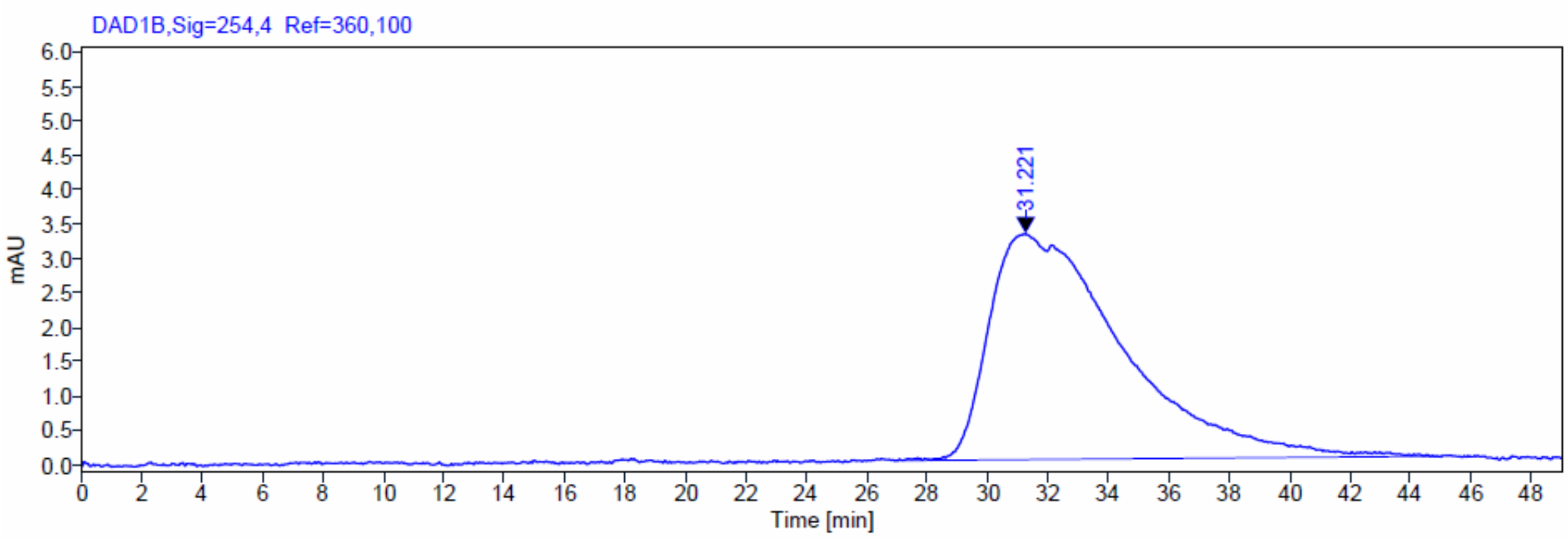

Signal: DAD1B,Sig=254,4 Ref=360,100

RT [min]	Width [min]	Area	Height	Area%
31.221	17.96	1015.06	3.29	100.00
	**Sum**	**1015.06**		

The retention time of the measured crystal corresponds to the same major enantiomer obtained in the cross-electrophile coupling reaction.

Methyl (*R*)-4-((4-chlorophenyl)(1-methylsiletan-1-yl)methyl)benzoate (3fa)

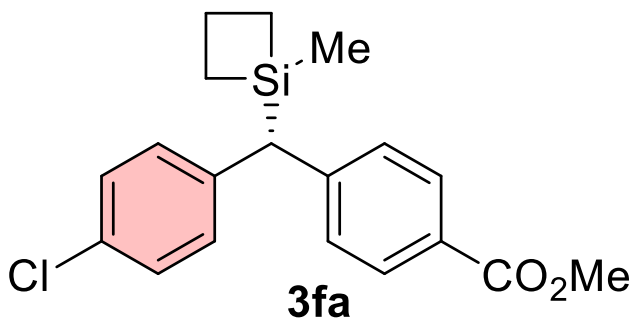

3fa

$C_{19}H_{21}ClO_2Si$

M_W = 344.91

The enantiomeric ratio of **3fa** was determined by HPLC analysis on a chiral stationary phase (*Daicel Chiralpak* AD-H column, column temperature 20 °C, solvent *n*-heptane:isopropanol = 99.9:0.1, flow rate 1.1 mL/min): t_R = 30.4 min (minor), t_R = 49.4 min (major).

Figure S27. Methyl 4-((4-chlorophenyl)(1-methylsiletan-1-yl)methyl)benzoate (*rac*-**3fa**).

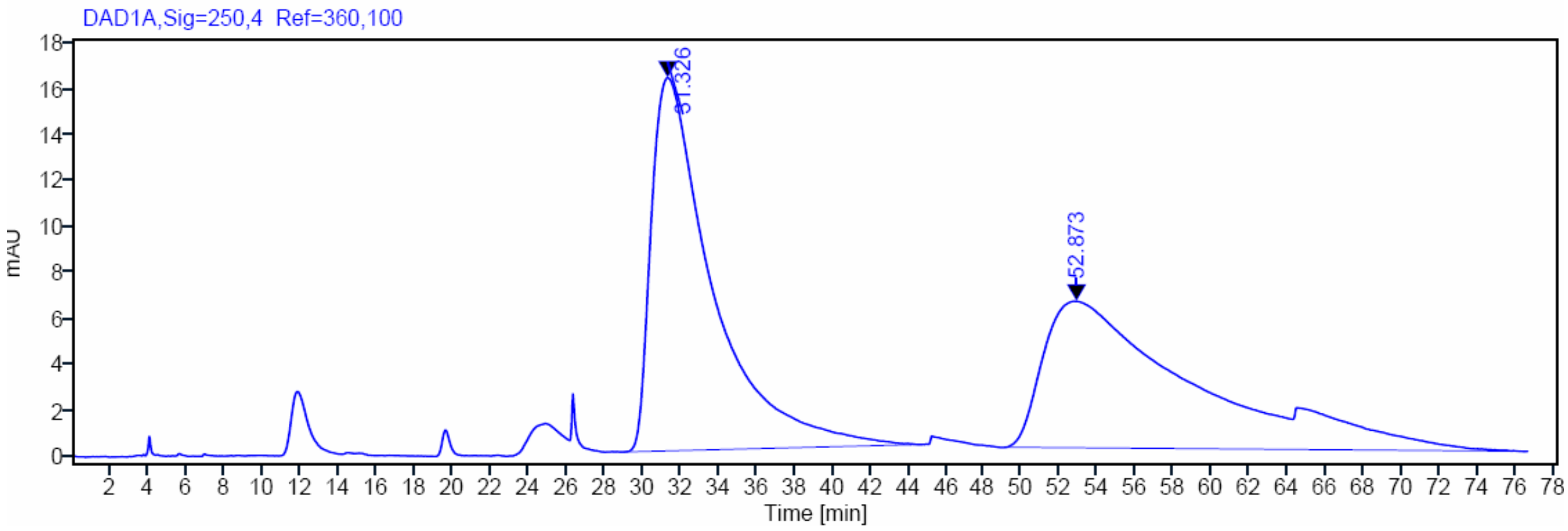

Signal: DAD1A,Sig=250,4 Ref=360,100

RT [min]	Width [min]	Area	Height	Area%
31.326	15.86	3646.38	16.26	50.81
52.873	27.58	3529.57	6.39	49.19
	**Sum**	**7175.95**		

Figure S28. Methyl (*R*)-4-((4-chlorophenyl)(1-methylsiletan-1-yl)methyl)benzoate [(*R*)-**3fa**].

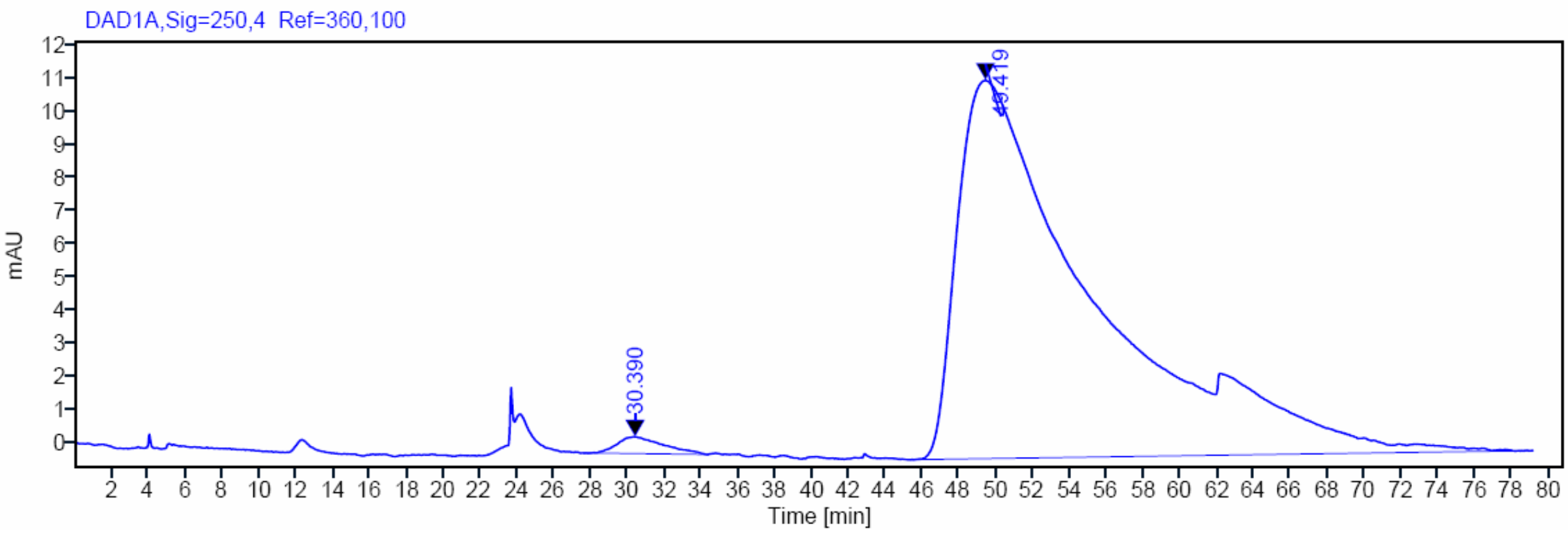

Signal: DAD1A,Sig=250,4 Ref=360,100

RT [min]	Width [min]	Area	Height	Area%
30.390	6.80	87.88	0.51	1.47
49.419	33.88	5879.17	11.44	98.53
	**Sum**	**5967.05**		

Methyl (*R*)-4-((3-methoxyphenyl)(1-methylsiletan-1-yl)methyl)benzoate (**3ga**)

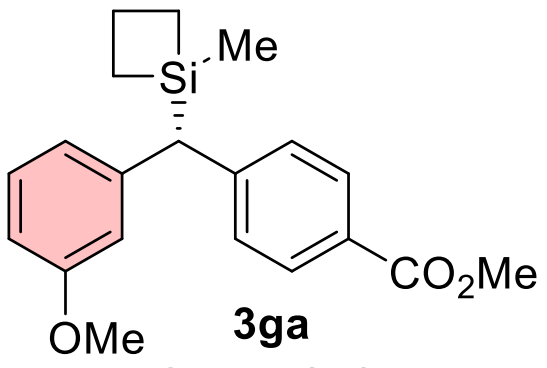

$C_{20}H_{24}O_3Si$
M_W = 340.49

The enantiomeric ratio of **3ga** was determined by HPLC analysis on a chiral stationary phase (*Daicel Chiralpak* AD-H column, column temperature 20 °C, solvent *n*-heptane:isopropanol = 98:2, flow rate 1.1 mL/min): t_R = 8.4 min (minor), t_R = 9.2 min (major).

Figure S29. Methyl 4-((3-methoxyphenyl)(1-methylsiletan-1-yl)methyl)benzoate (*rac*-**3ga**).

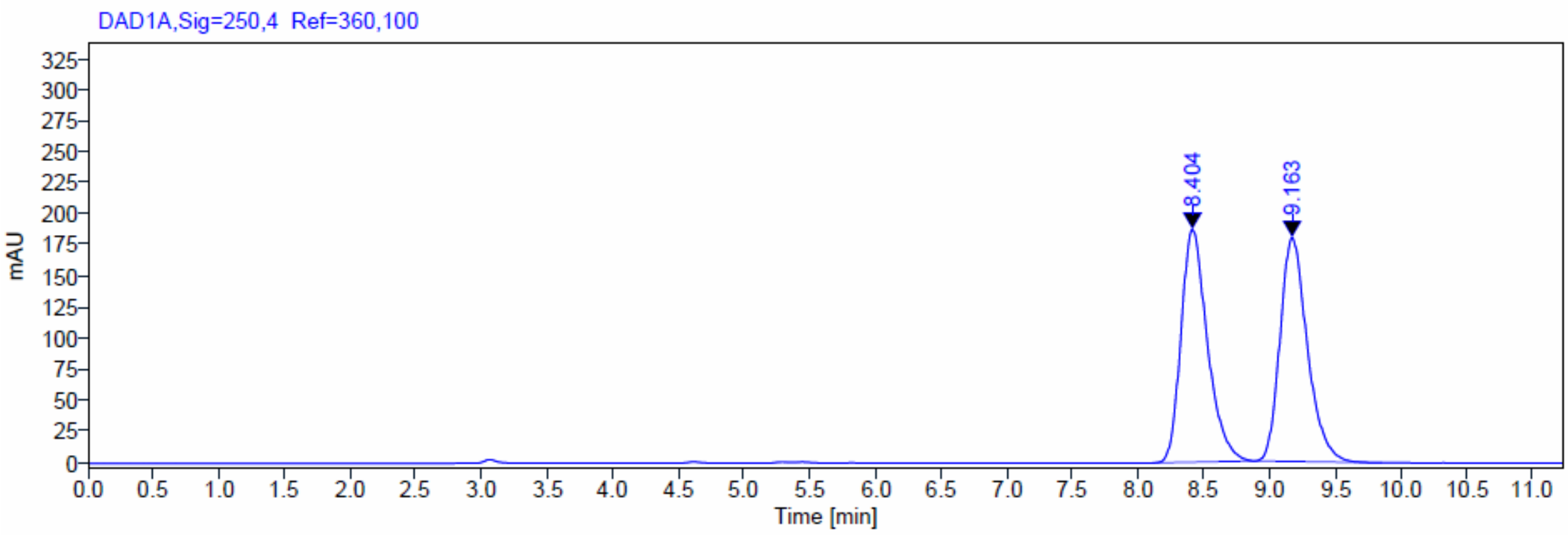

Signal: DAD1A,Sig=250,4 Ref=360,100

RT [min]	Width [min]	Area	Height	Area%
8.404	0.82	2611.94	186.90	50.09
9.163	0.96	2603.02	179.91	49.91
	**Sum**	**5214.96**		

Figure S30. Methyl (*R*)-4-((3-methoxyphenyl)(1-methylsiletan-1-yl)methyl)benzoate [(*R*)-**3ga**].

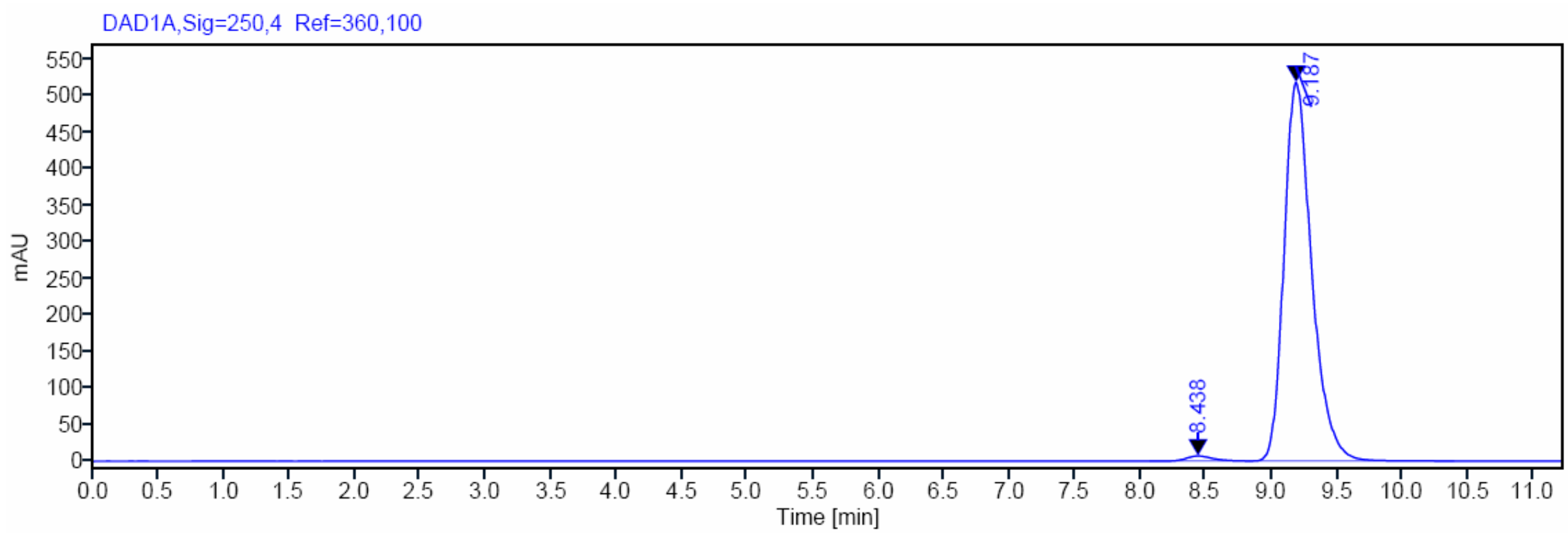

Signal: DAD1A,Sig=250,4 Ref=360,100

RT [min]	Width [min]	Area	Height	Area%
8.438	0.76	94.62	6.91	1.23
9.187	1.56	7616.89	518.18	98.77
	**Sum**	**7711.51**		

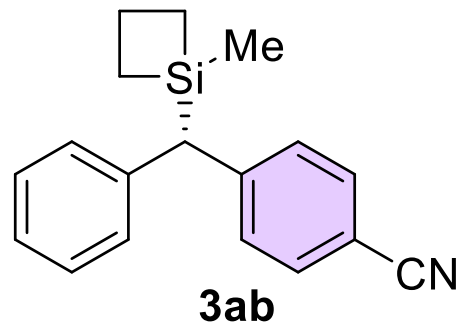

3ab
$C_{18}H_{19}NSi$
M_W = 277.44

(*S*)-4-((1-Methylsiletan-1-yl)(phenyl)methyl)benzonitrile (**3ab**)

The enantiomeric ratio of **3ab** was determined by HPLC analysis on a chiral stationary phase (*Daicel Chiralpak* AD-H column, column temperature 20 °C, solvent *n*-heptane:isopropanol = 99.9:0.1, flow rate 1.1 mL/min): t_R = 31.3 min (minor), t_R = 33.1 min (major).

Figure S31. 4-((1-Methylsiletan-1-yl)(phenyl)methyl)benzonitrile (*rac*-**3ab**).

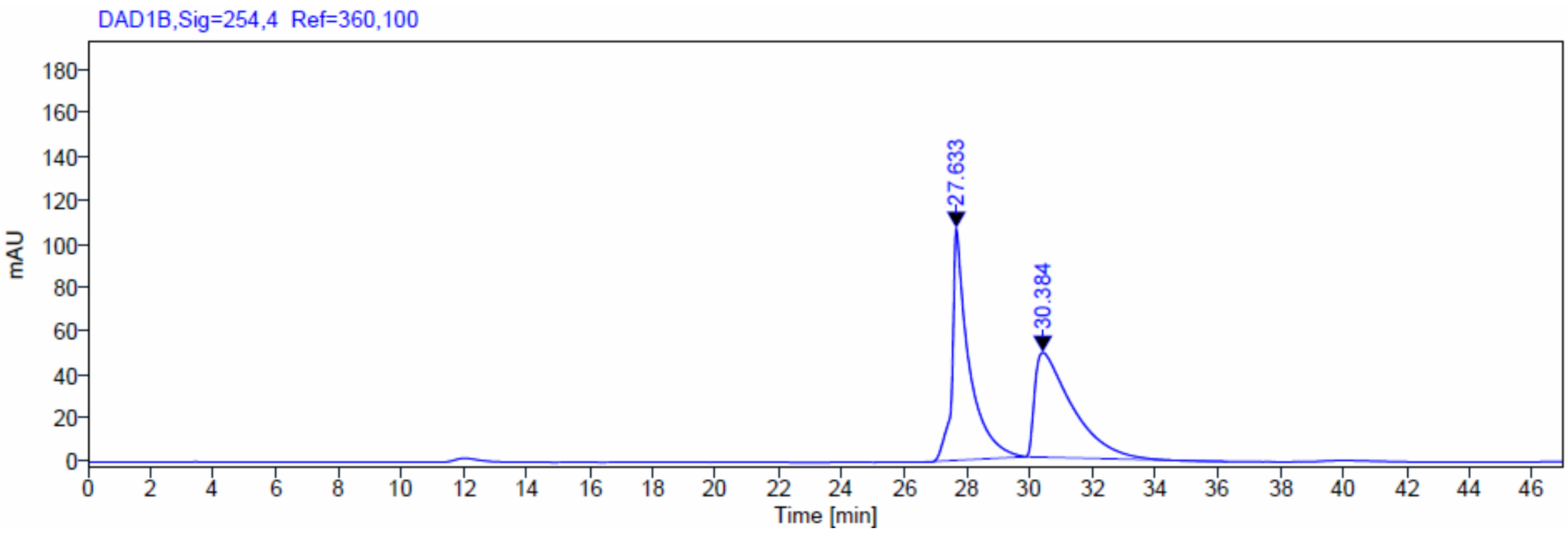

Signal: DAD1B,Sig=254,4 Ref=360,100

RT [min]	Width [min]	Area	Height	Area%
27.633	3.38	3936.33	106.68	49.66
30.384	6.43	3990.31	48.14	50.34
	**Sum**	**7926.64**		

Figure S32. (*S*)-4-((1-Methylsiletan-1-yl)(phenyl)methyl)benzonitrile [(*S*)-**3ab**].

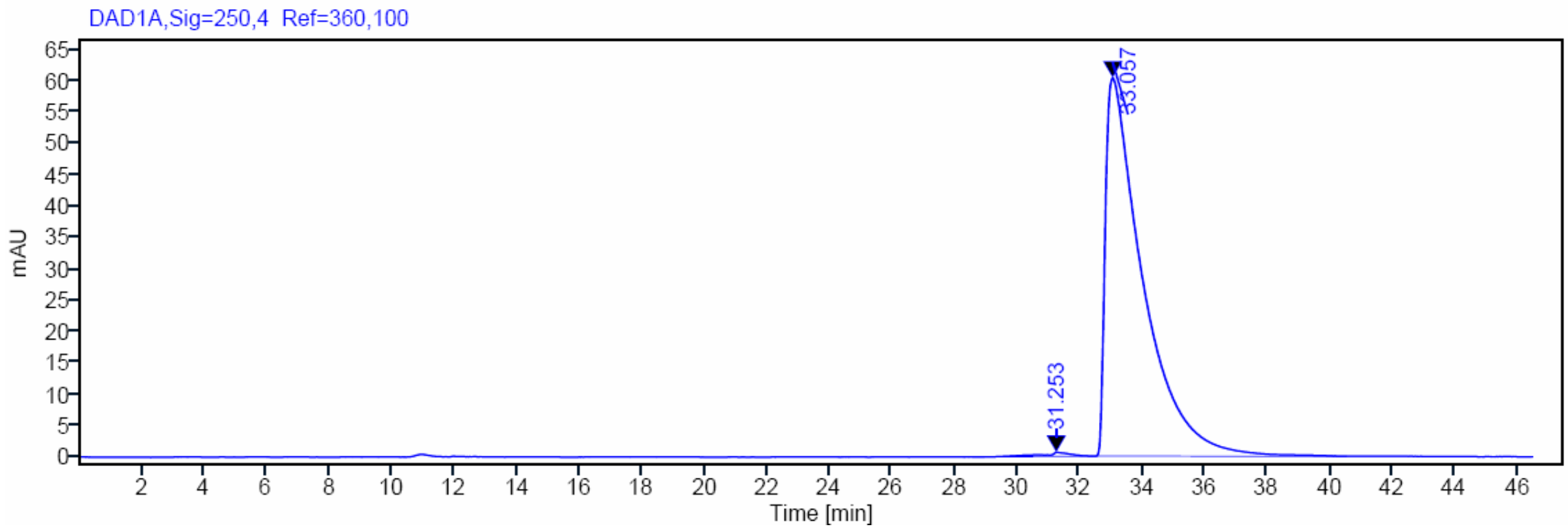

Signal: DAD1A,Sig=250,4 Ref=360,100

RT [min]	Width [min]	Area	Height	Area%
31.253	3.84	41.12	0.63	0.81
33.057	8.14	5020.96	60.34	99.19
	**Sum**	**5062.08**		

Methyl (*S*)-6-((1-methylsiletan-1-yl)(phenyl)methyl)-2-naphthoate (**3ae**)

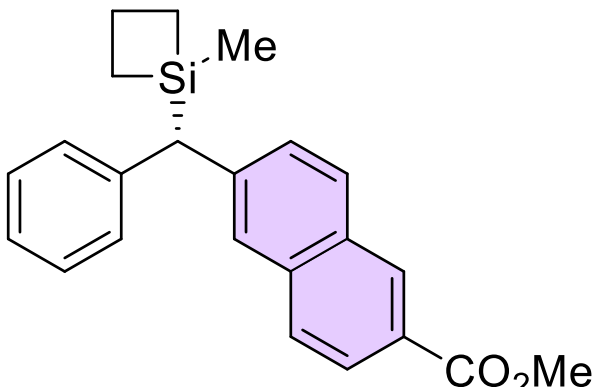

3ae
$C_{23}H_{24}O_2Si$
M_W = 360.53

The enantiomeric ratio of **3ae** was determined by HPLC analysis on a chiral stationary phase (*Daicel Chiralcel* OD-H column, column temperature 20 °C, solvent *n*-heptane:isopropanol = 100:0, flow rate 0.6 mL/min): t_R = 110.2 min (minor), t_R = 120.9 min (major). The minor enantiomer was not found in the HPLC trace of the enantioenriched sample for this compound.

Figure S33. Methyl 6-((1-methylsiletan-1-yl)(phenyl)methyl)-2-naphthoate (*rac*-**3ae**).

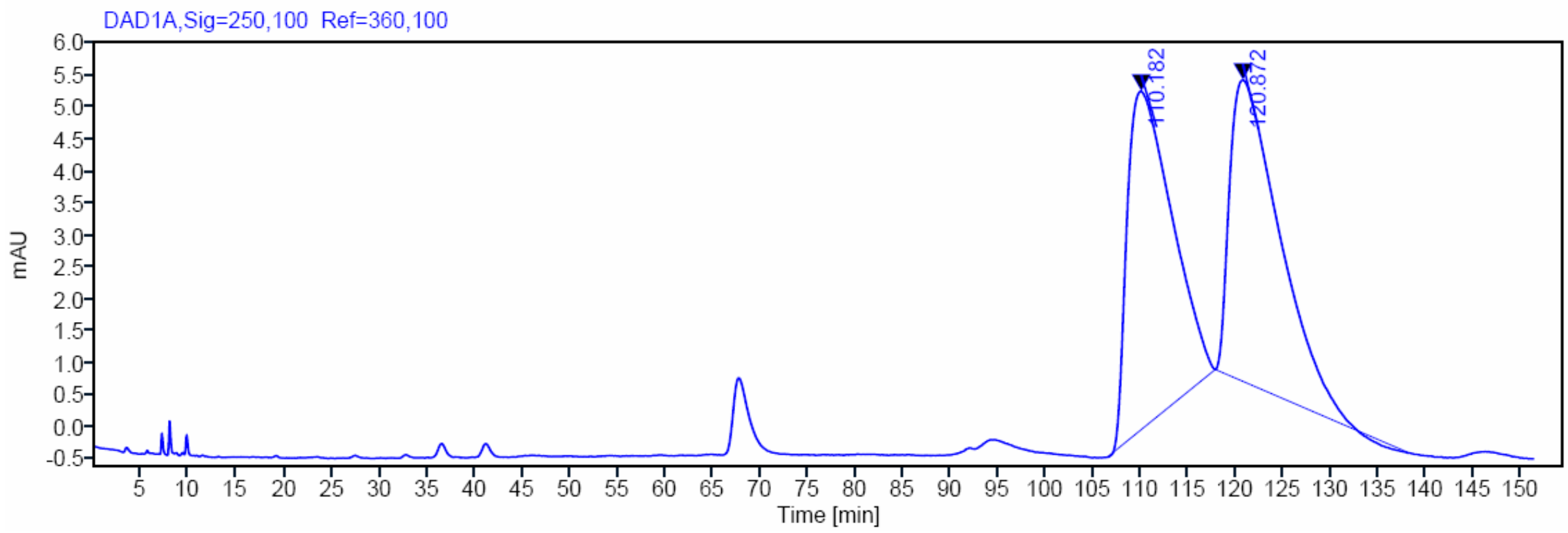

Signal:	DAD1A,Sig=250,100 Ref=360,100			
**RT [min]**	**Width [min]**	**Area**	**Height**	**Area%**
110.182	11.35	1720.29	5.29	49.78
120.872	20.42	1735.74	4.71	50.22
	**Sum**	**3456.03**		

Figure S34. Methyl (*S*)-6-((1-methylsiletan-1-yl)(phenyl)methyl)-2-naphthoate [(*S*)-**3ae**].

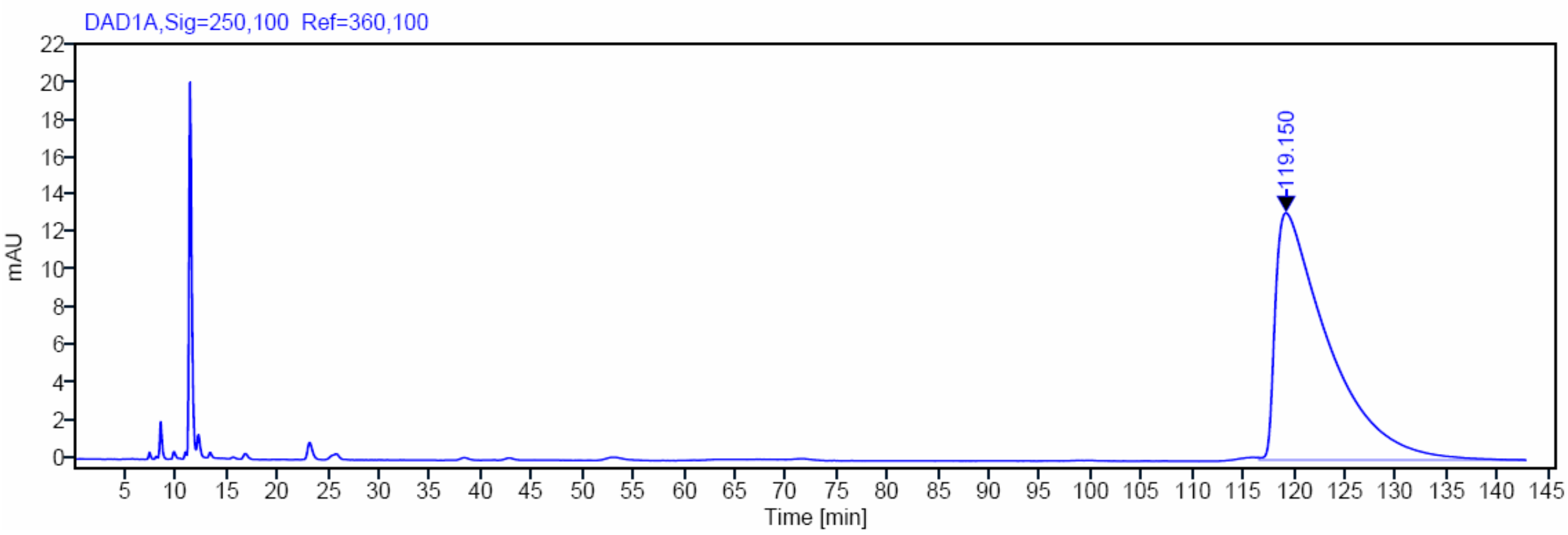

Signal:	DAD1A,Sig=250,100 Ref=360,100			
**RT [min]**	**Width [min]**	**Area**	**Height**	**Area%**
119.150	26.18	4739.70	13.11	100.00
	**Sum**	**4739.70**		

Methyl (*S*)-4-(phenyl(trimethylgermyl)methyl)benzoate (5aa)

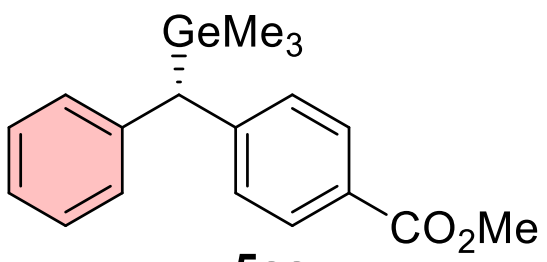

5aa
$C_{18}H_{22}GeO_2$
M_W = 343.00

The enantiomeric ratio of **5aa** was determined by HPLC analysis on a chiral stationary phase (*Daicel Chiralcel* OD-H column, column temperature 20 °C, solvent *n*-heptane:isopropanol = 99.9:0.1, flow rate 1.2 mL/min): t_R = 20.0 min (major), t_R = 26.2 min (minor).

Figure S35. Methyl 4-(phenyl(trimethylgermyl)methyl)benzoate (*rac*-**5aa**).

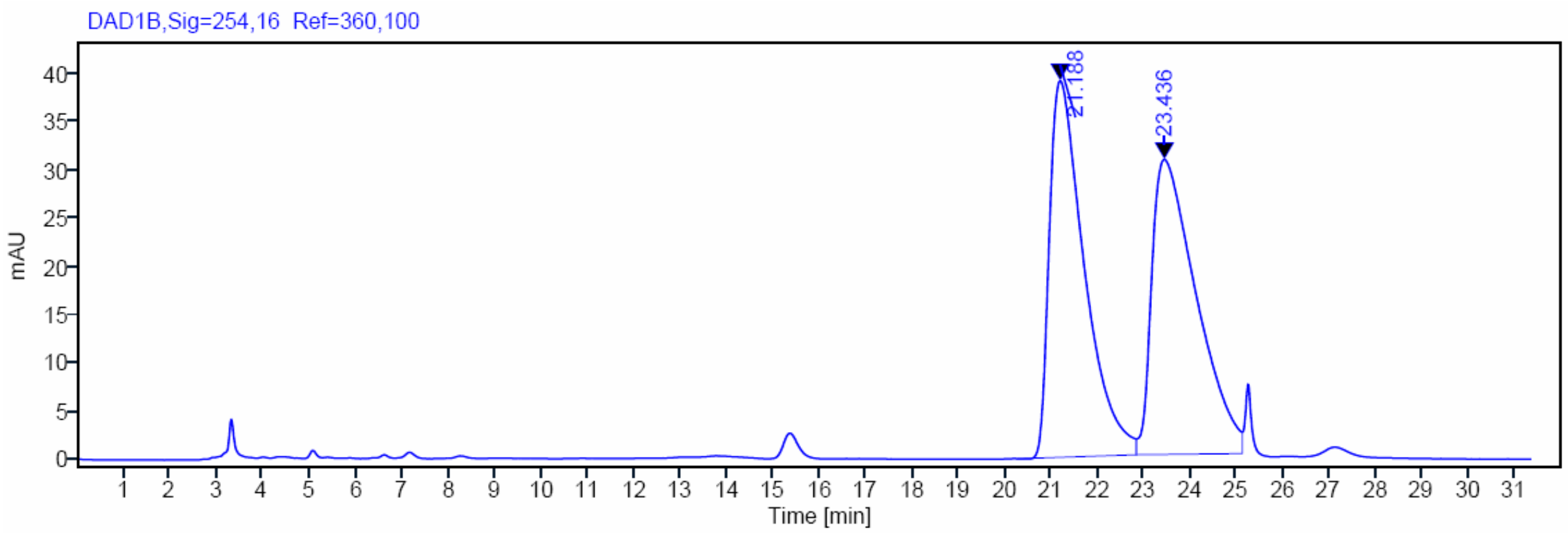

Signal: DAD1B,Sig=254,16 Ref=360,100

RT [min]	Width [min]	Area	Height	Area%
21.188	2.57	2046.43	39.13	50.01
23.436	2.28	2045.38	30.65	49.99
	**Sum**	**4091.81**		

Figure S36. Methyl (*S*)-4-(phenyl(trimethylgermyl)methyl)benzoate [(*S*)-**5aa**].

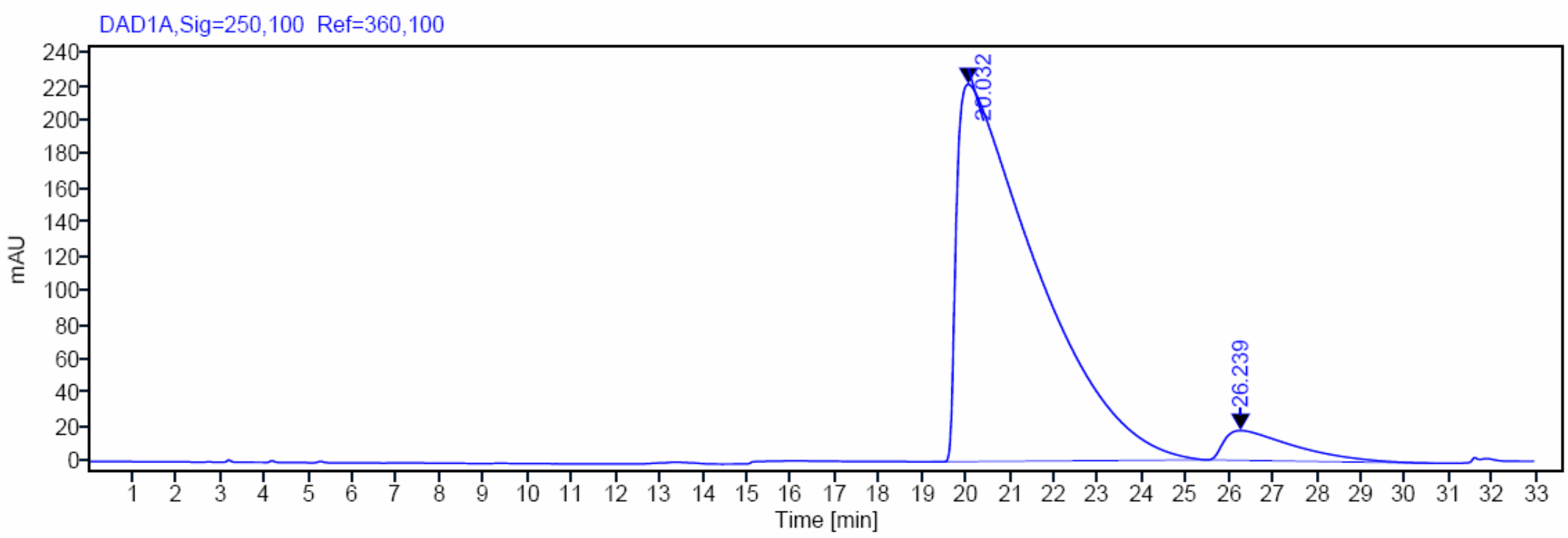

Signal: DAD1A,Sig=250,100 Ref=360,100

RT [min]	Width [min]	Area	Height	Area%
20.032	6.26	27979.62	221.54	93.51
26.239	5.26	1942.00	17.48	6.49
	**Sum**	**29921.63**		

Methyl (*S*)-4-(*p*-tolyl(trimethylgermyl)methyl)benzoate (5ba)

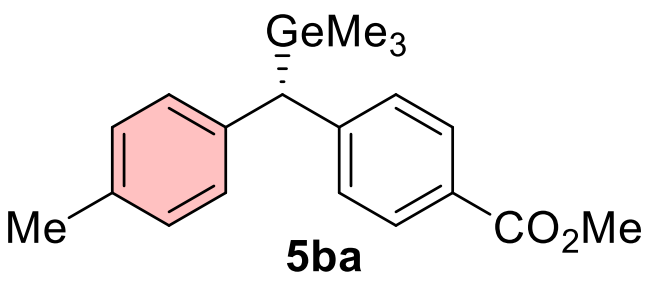

5ba
$C_{19}H_{24}GeO_2$
M_W = 357.03

The enantiomeric ratio of **5ba** was determined by HPLC analysis on a chiral stationary phase (*Daicel Chiralpak* AD-H column, column temperature 40 °C, solvent *n*-heptane:isopropanol = 99.9:0.1, flow rate 1.2 mL/min): t_R = 17.1 min (minor), t_R = 25.3 min (major).

Figure S37. Methyl 4-(*p*-tolyl(trimethylgermyl)methyl)benzoate (*rac*-**5ba**).

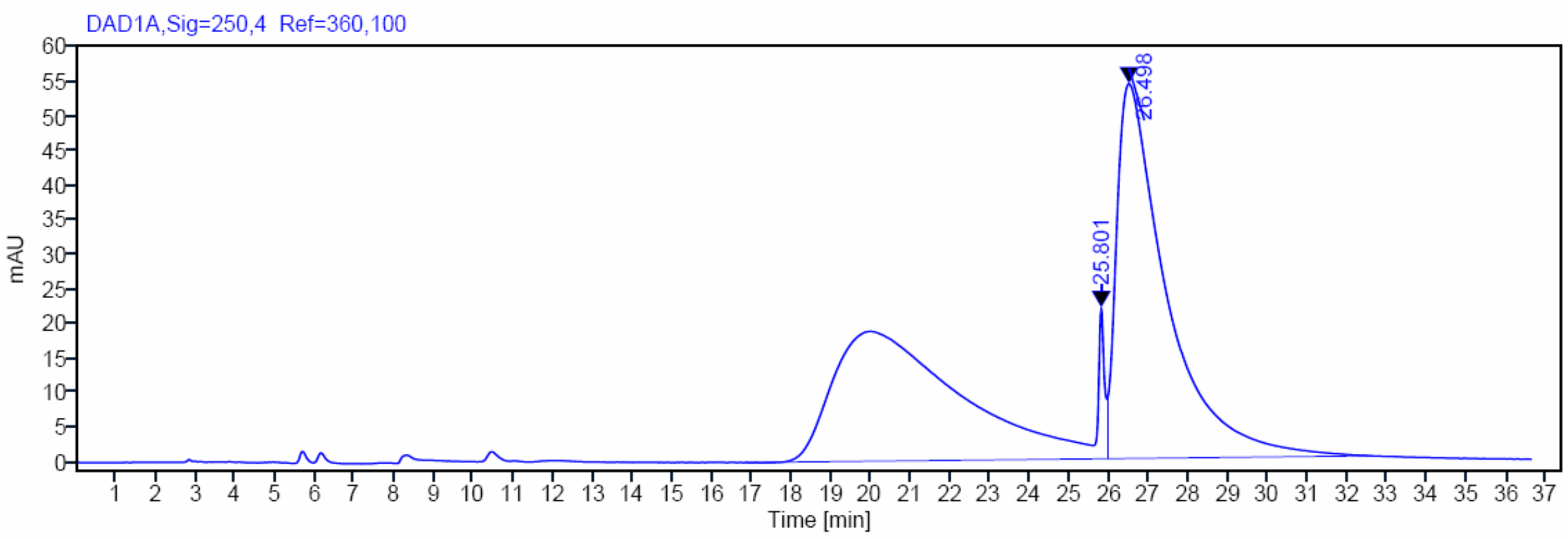

Signal: DAD1A,Sig=250,4 Ref=360,100

RT [min]	Width [min]	Area	Height	Area%
25.801	9.15	4404.36	21.84	48.23
26.498	6.65	4728.22	54.22	51.77
	**Sum**	**9132.58**		

Figure S38. Methyl (*S*)-4-(*p*-tolyl(trimethylgermyl)methyl)benzoate [(*S*)-**5ba**].

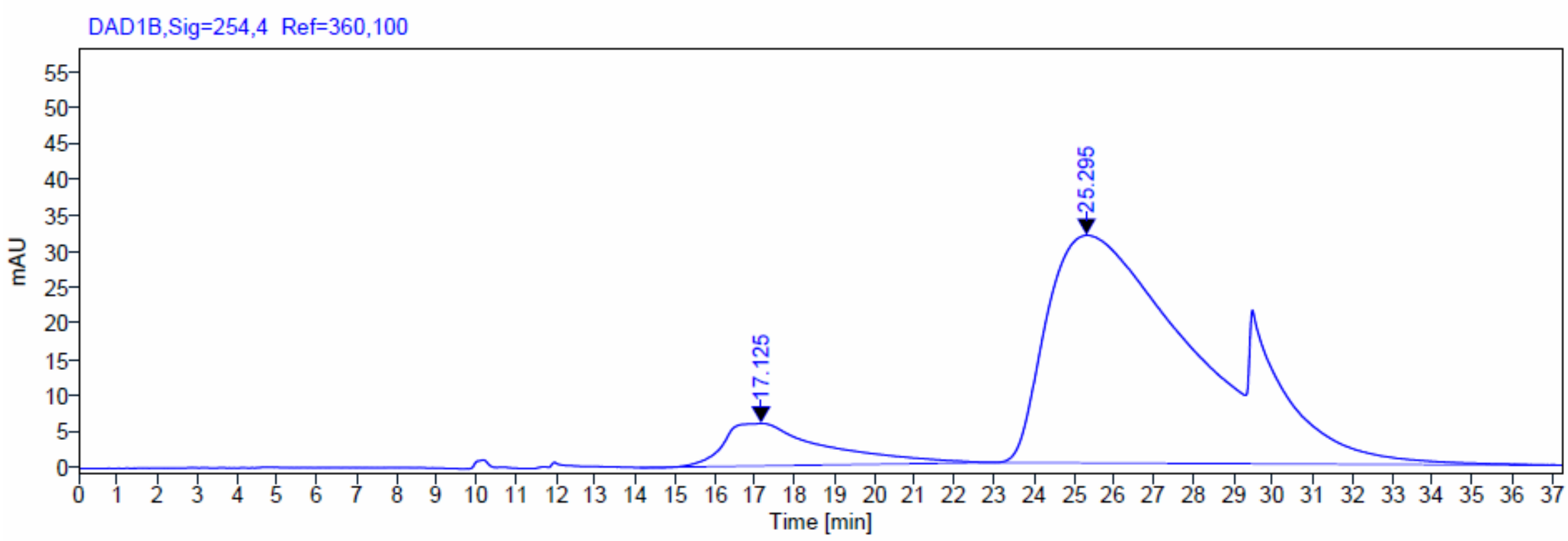

Signal: DAD1B,Sig=254,4 Ref=360,100

RT [min]	Width [min]	Area	Height	Area%
17.125	8.94	1029.51	5.90	10.66
25.295	14.49	8632.54	31.64	89.34
	**Sum**	**9662.05**		

Methyl (*R*)-4-((4-(trifluoromethyl)phenyl)(trimethylgermyl)methyl)benzoate (**5ca**)

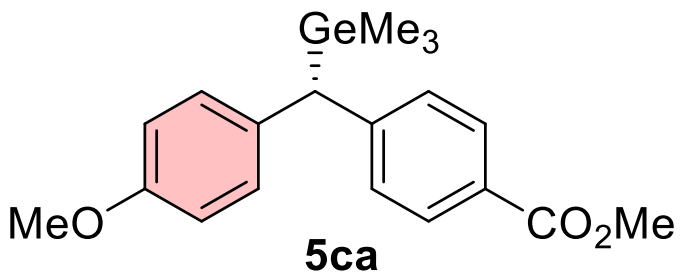

5ca
$C_{19}H_{24}GeO_3$
M_W = 373.03

The enantiomeric ratio of **5ca** was determined by HPLC analysis on a chiral stationary phase (*Daicel Chiralpak* AD-H column, column temperature 20 °C, solvent *n*-heptane:isopropanol = 98:2, flow rate 0.6 mL/min): t_R = 17.3 min (minor), t_R = 29.2 min (major).

Figure S39. Methyl 4-((4-(trifluoromethyl)phenyl)(trimethylgermyl)methyl)benzoate (*rac*-**5ca**).

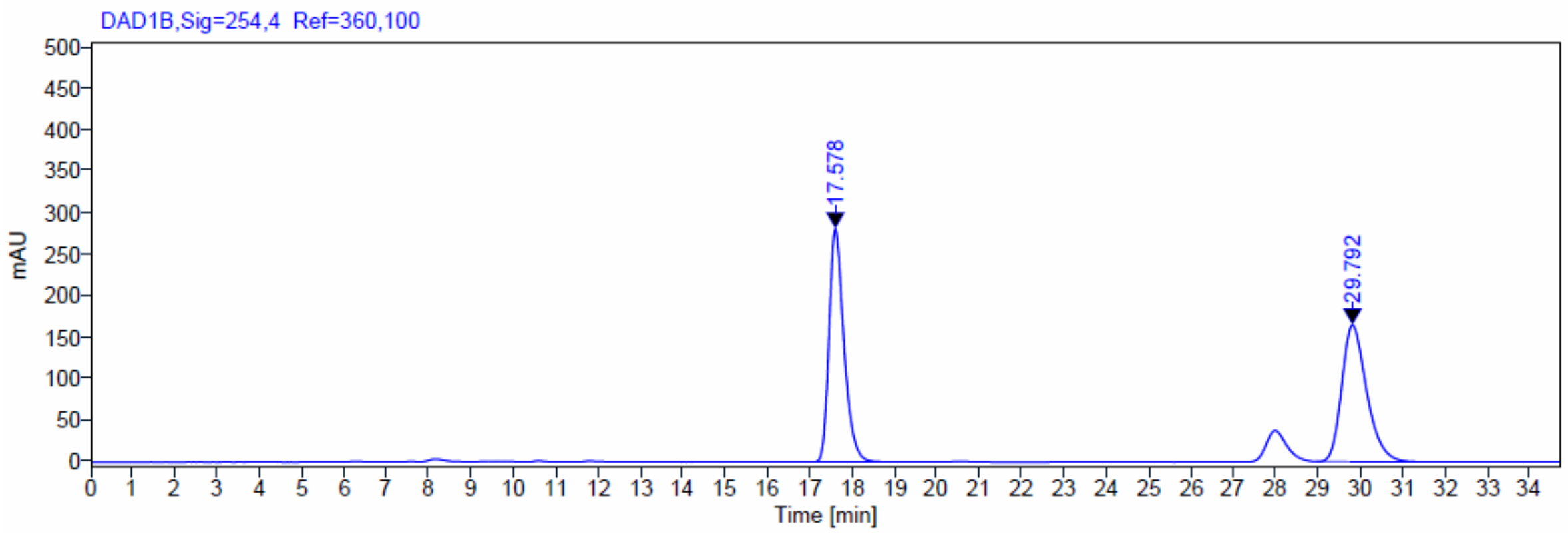

Signal:	DAD1B,Sig=254,4 Ref=360,100			
RT [min]	Width [min]	Area	Height	Area%
17.578	2.11	6847.82	280.97	50.43
29.792	2.87	6732.13	164.78	49.57
	Sum	13579.95		

Figure S40. Methyl (*R*)-4-((4-(trifluoromethyl)phenyl)(trimethylgermyl)methyl)benzoate [(*R*)-**5ca**].

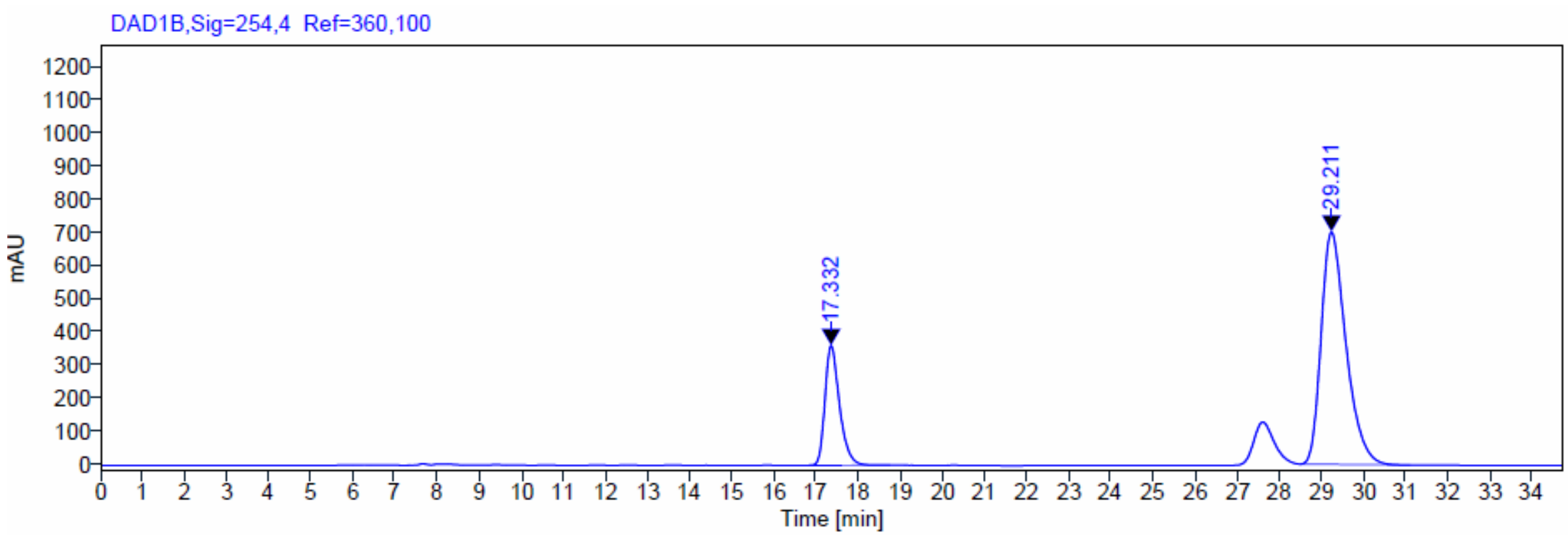

Signal:	DAD1B,Sig=254,4 Ref=360,100			
RT [min]	Width [min]	Area	Height	Area%
17.332	2.42	8674.14	361.86	23.16
29.211	2.60	28776.92	702.52	76.84
	Sum	37451.06		

Methyl (*R*)-4-((4-(trifluoromethyl)phenyl)(trimethylgermyl)methyl)benzoate (**5da**)

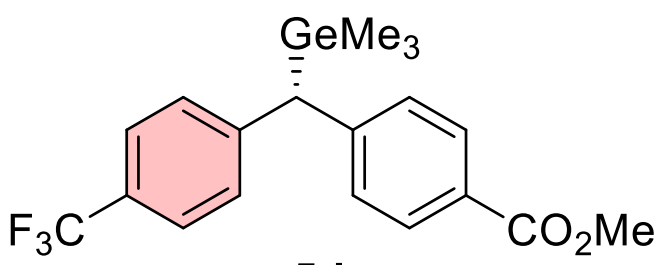

5da
$C_{19}H_{21}F_3GeO_2$
M_W = 411.00

The enantiomeric ratio of **5da** was determined by HPLC analysis on a chiral stationary phase (*Daicel Chiralpak* AD-H column, column temperature 40 °C, solvent *n*-heptane:isopropanol = 99.9:0.1, flow rate 1.2 mL/min): t_R = 21.9 min (minor), t_R = 28.2 min (major).

Figure S41. Methyl 4-((4-(trifluoromethyl)phenyl)(trimethylgermyl)methyl)benzoate (*rac*-**5da**).

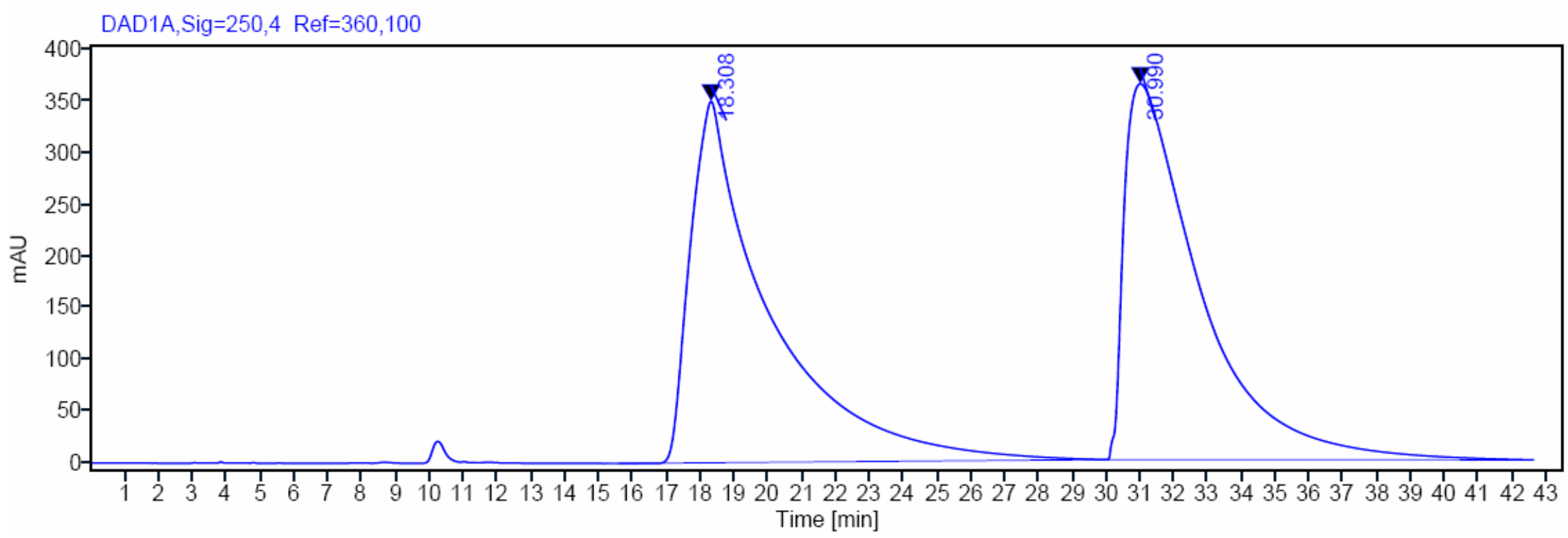

Signal:	DAD1A,Sig=250,4 Ref=360,100			
**RT [min]**	**Width [min]**	**Area**	**Height**	**Area%**
18.308	14.46	56865.52	350.79	50.03
30.990	12.56	56798.17	364.74	49.97
	**Sum**	**113663.69**		

Figure S42. Methyl (*R*)-4-((4-(trifluoromethyl)phenyl)(trimethylgermyl)methyl)benzoate [(*R*)-**5da**].

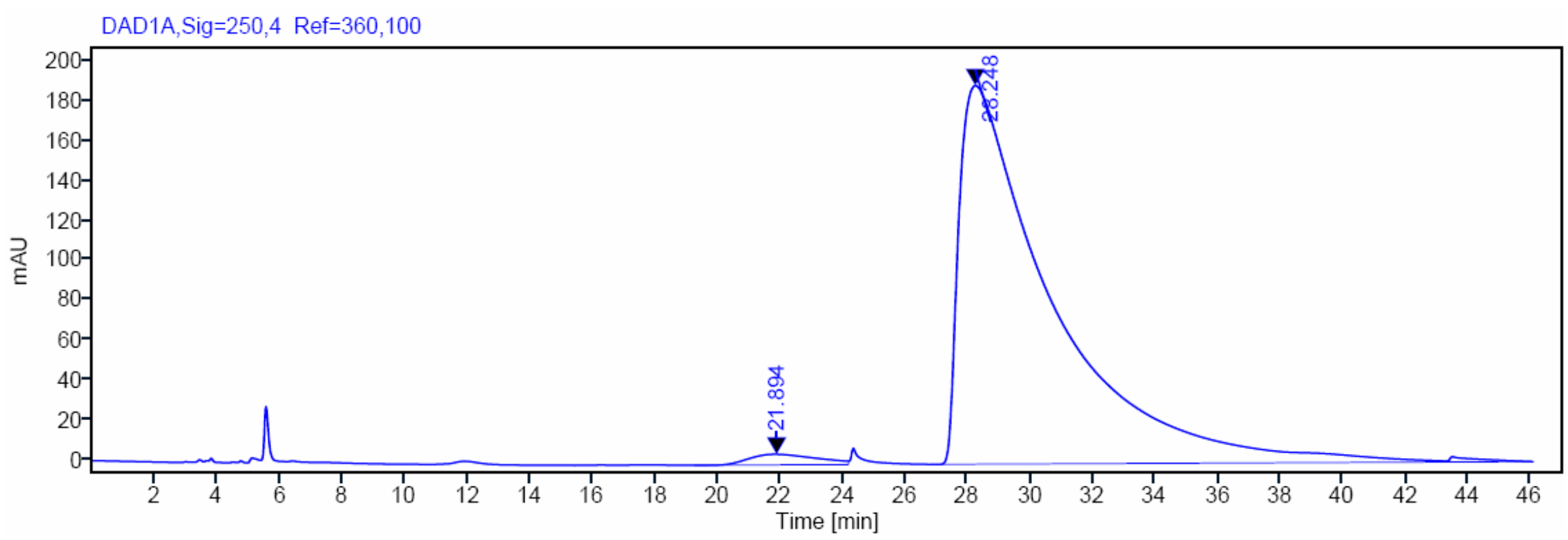

Signal:	DAD1A,Sig=250,4 Ref=360,100			
**RT [min]**	**Width [min]**	**Area**	**Height**	**Area%**
21.894	4.93	798.73	5.32	1.99
28.248	19.26	39397.15	189.69	98.01
	**Sum**	**40195.88**		

Figure S43. Measured crystal of methyl (*R*)-4-((4-(trifluoromethyl)phenyl)(trimethylgermyl)methyl)benzoate [(*R*)-**5da**].

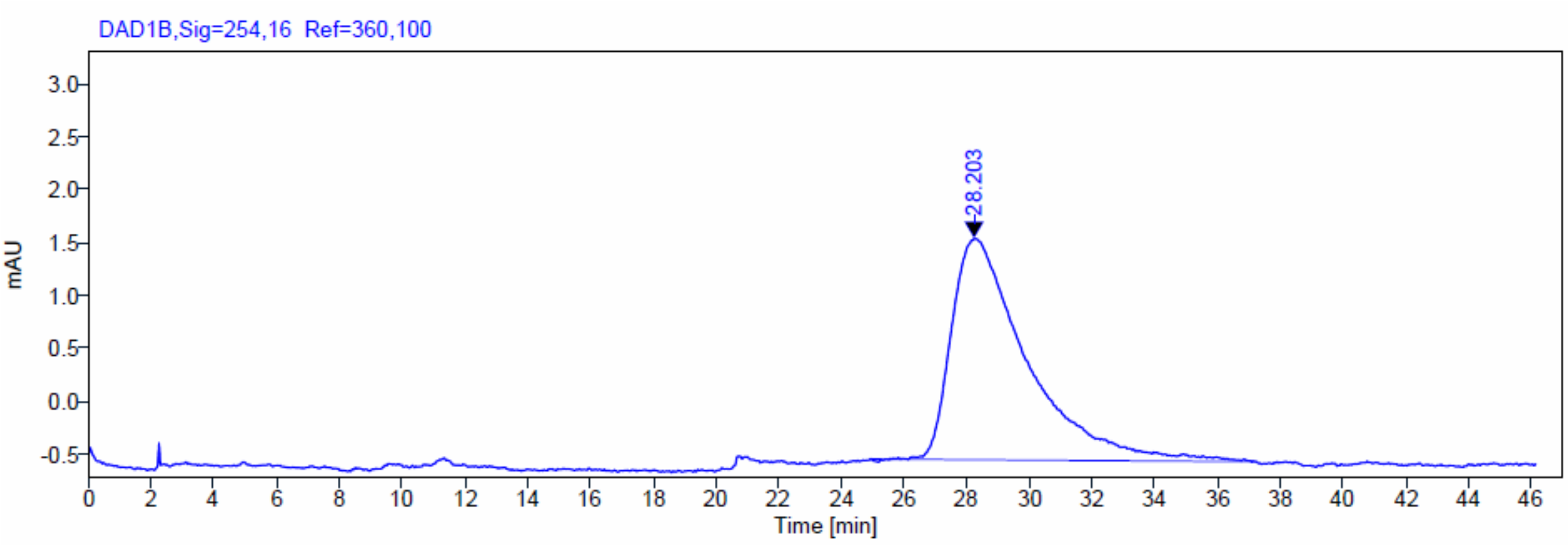

Signal: DAD1B,Sig=254,16 Ref=360,100

RT [min]	Width [min]	Area	Height	Area%
28.203	12.26	348.68	2.09	100.00
	**Sum**	**348.68**		

The retention time of the measured crystal corresponds to the same major enantiomer obtained in the cross-electrophile coupling reaction.

Methyl (*S*)-4-(phenyl(triethylgermyl)methyl)benzoate (9aa)

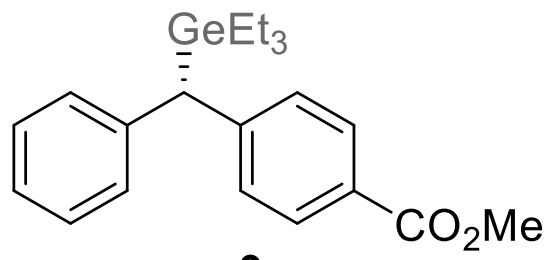

9aa

$C_{21}H_{28}GeO_2$

M_W = 385.08

The enantiomeric ratio of **9aa** was determined by HPLC analysis on a chiral stationary phase (*Daicel Chiralpak* AD-H column, column temperature 10 °C, solvent *n*-heptane:isopropanol = 99.9:0.1, flow rate 0.7 mL/min): t_R = 23.9 min (minor), t_R = 27.7 min (major).

Figure S44. Methyl (*R*)-4-(phenyl(triethylgermyl)methyl)benzoate (*rac*-**9aa**).

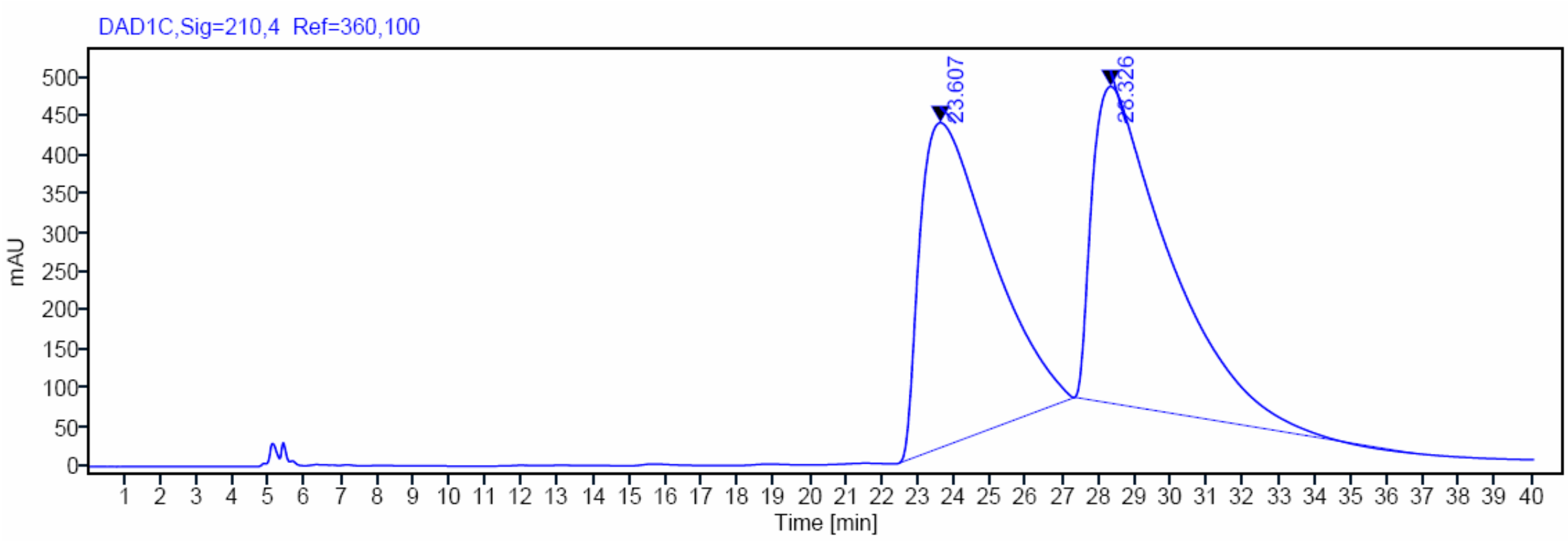

**Signal:**	DAD1C,Sig=210,4 Ref=360,100			
**RT [min]**	**Width [min]**	**Area**	**Height**	**Area%**
23.607	4.89	57706.52	417.57	48.74
28.326	9.39	60678.91	406.87	51.26
	**Sum**	**118385.43**		

Figure S45. Methyl (*S*)-4-(phenyl(triethylgermyl)methyl)benzoate [(*S*)-**9aa**].

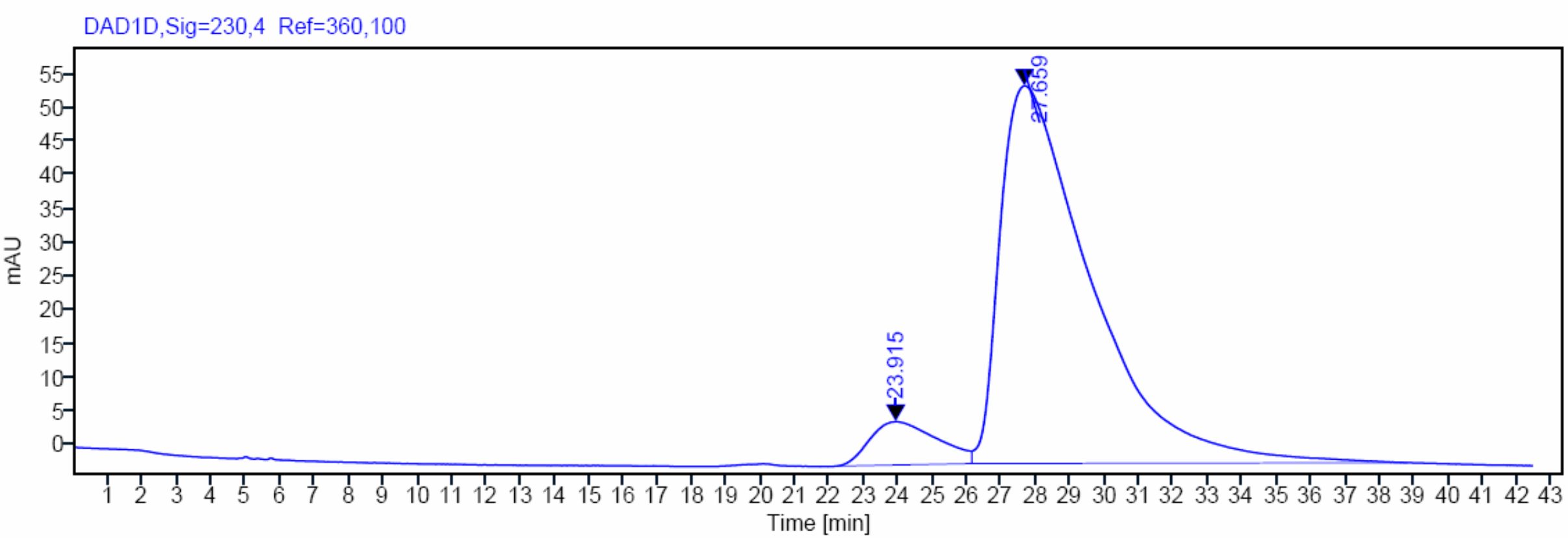

**Signal:**	DAD1D,Sig=230,4 Ref=360,100			
**RT [min]**	**Width [min]**	**Area**	**Height**	**Area%**
23.915	4.32	875.01	6.43	7.84
27.659	12.68	10292.89	55.94	92.16
	**Sum**	**11167.90**		

(*S*)-4-(Phenyl(trimethylgermyl)methyl)benzonitrile (**5ab**)

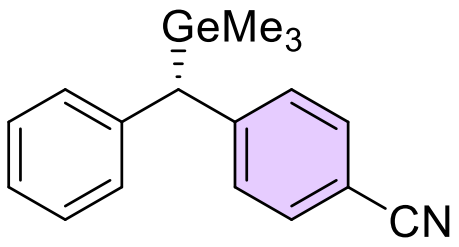

5ab
$C_{17}H_{19}GeN$
M_W = 309.98

The enantiomeric ratio of **5ab** was determined by HPLC analysis on a chiral stationary phase (*Daicel Chiralpak* AD-H column, column temperature 20 °C, solvent *n*-heptane:isopropanol = 99.9:0.1, flow rate 1.1 mL/min): t_R = 24.2 min (major), t_R = 33.7 min (minor).

Figure S46. 4-(Phenyl(trimethylgermyl)methyl)benzonitrile (*rac*-**5ab**).

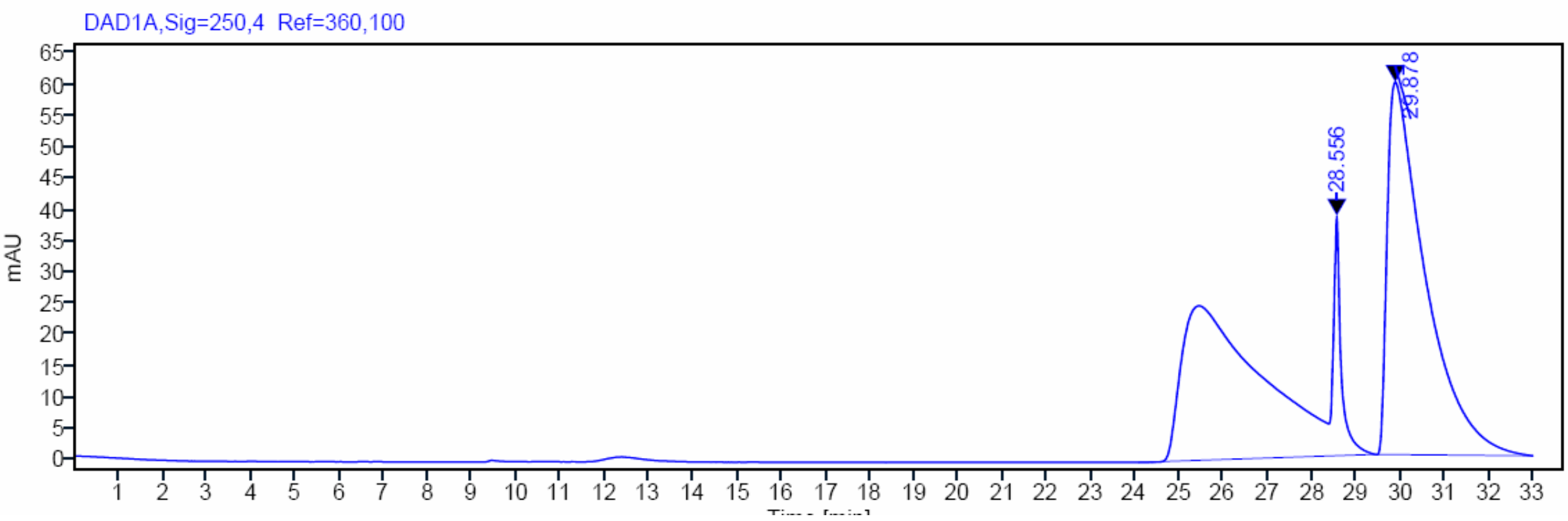

Signal: DAD1A,Sig=250,4 Ref=360,100

RT [min]	Width [min]	Area	Height	Area%
28.556	5.36	3532.93	38.29	49.84
29.878	3.57	3555.92	59.43	50.16
	**Sum**	**7088.85**		

Figure S47. (*S*)-4-(Phenyl(trimethylgermyl)methyl)benzonitrile [(*S*)-**5ab**].

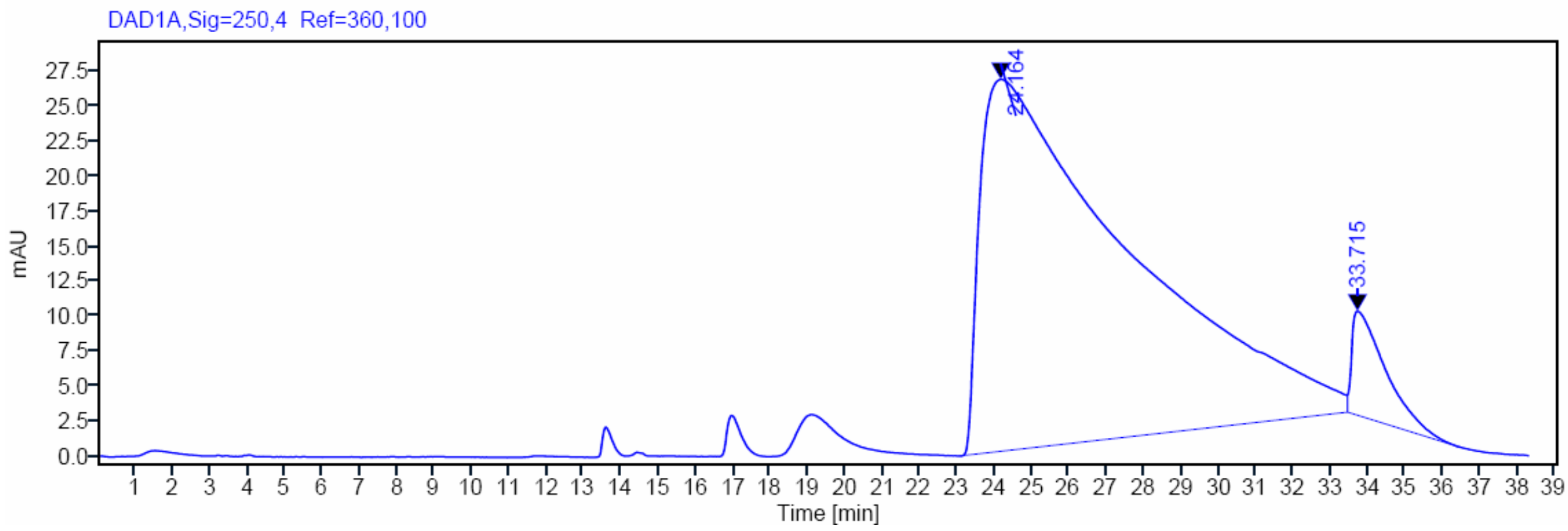

Signal: DAD1A,Sig=250,4 Ref=360,100

RT [min]	Width [min]	Area	Height	Area%
24.164	10.40	7345.62	26.61	93.91
33.715	3.00	476.71	7.44	6.09
	**Sum**	**7822.34**		

(*S*)-Trimethyl(phenyl(4-(trifluoromethyl)phenyl)methyl)germane (5ah)

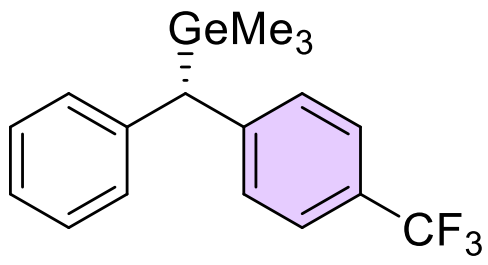

5ah
$C_{17}H_{19}F_3Ge$
M_W = 352.96

The enantiomeric ratio of **5ah** was determined by chiral GLC analysis (*Supelco Astec CHIRALDEX B-DM* column (30 m × 250 µm × 0.12 µm film thickness), column temperature 60 °C for 30 min, then 0.5 °C/min until 175 °C, flow rate 1.3 mL/min): t_R = 172.2 min (minor), t_R = 173.0 min (major).

Figure S48. Trimethyl(phenyl(4-(trifluoromethyl)phenyl)methyl)germane (*rac*-**5ah**).

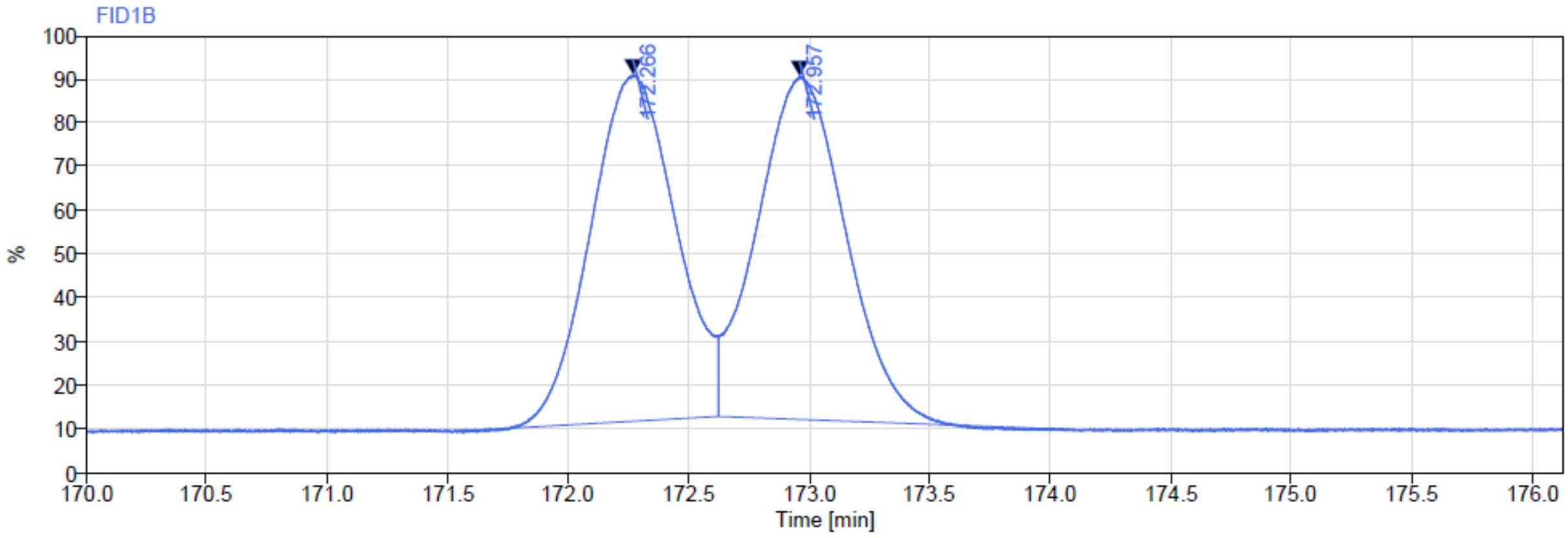

Signal: FID1B

RT [min]	Type	Width [min]	Area	Height	Area%
172.266	MM m	1.12	283.41	11.51	49.29
172.957	MM m	1.40	291.60	11.40	50.71
		**Sum**	**575.01**		

Figure S49. (*S*)-Trimethyl(phenyl(4-(trifluoromethyl)phenyl)methyl)germane [(*S*)-**5ah**].

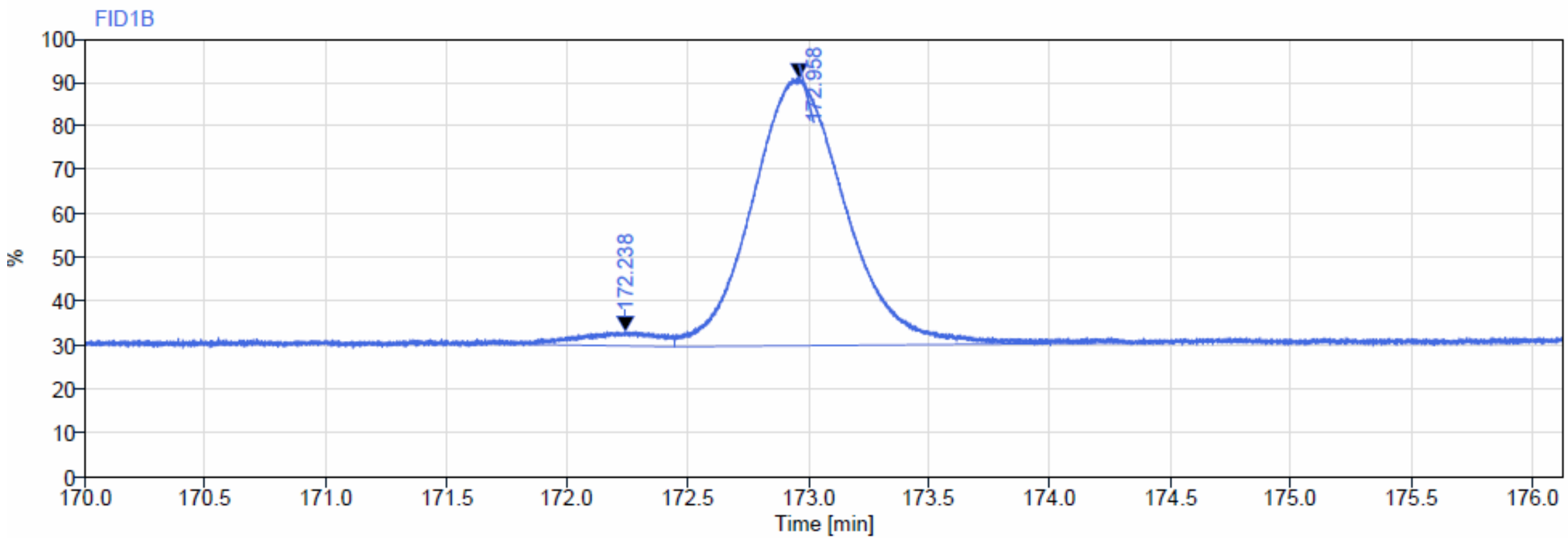

Signal: FID1B

RT [min]	Type	Width [min]	Area	Height	Area%
172.238	MM m	0.73	5.12	0.22	4.18
172.958	MM m	1.90	117.16	4.32	95.82
		**Sum**	**122.28**		

(*S*)-((3-Methoxyphenyl)(phenyl)methyl)trimethylgermane (5ai)

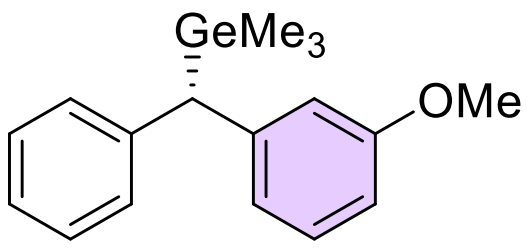

5ai
$C_{17}H_{22}GeO$
M_W = 314.99

The enantiomeric ratio of **5ai** was determined by HPLC analysis on a chiral stationary phase (*Daicel Chiralcel* AD-H column, column temperature 20 °C, solvent *n*-heptane:isopropanol = 100:0, flow rate 0.15 mL/min): t_R = 54.7 min (minor), t_R = 57.2 min (major).

Figure S50. ((3-Methoxyphenyl)(phenyl)methyl)trimethylgermane (*rac*-**5ai**).

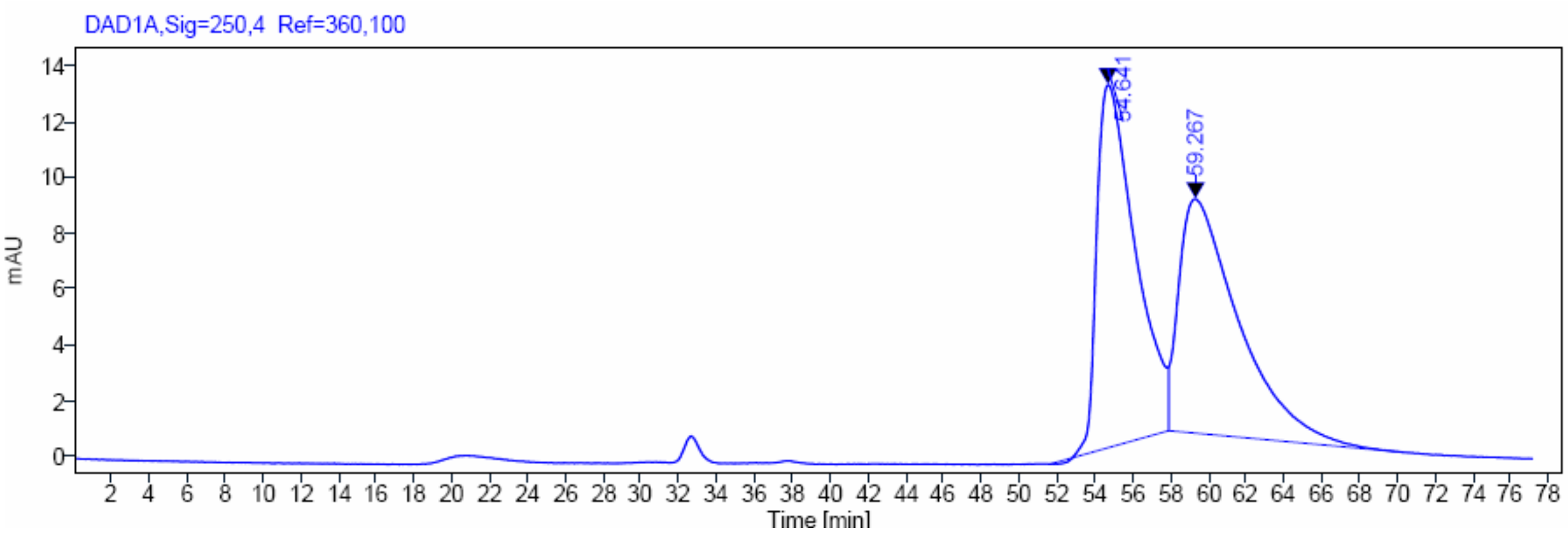

Signal: DAD1A,Sig=250,4 Ref=360,100

RT [min]	Width [min]	Area	Height	Area%
54.641	6.29	1839.23	12.99	49.77
59.267	12.46	1856.35	8.38	50.23
	**Sum**	**3695.58**		

Figure S51. (*S*)-((3-Methoxyphenyl)(phenyl)methyl)trimethylgermane [(*S*)-**5ai**].

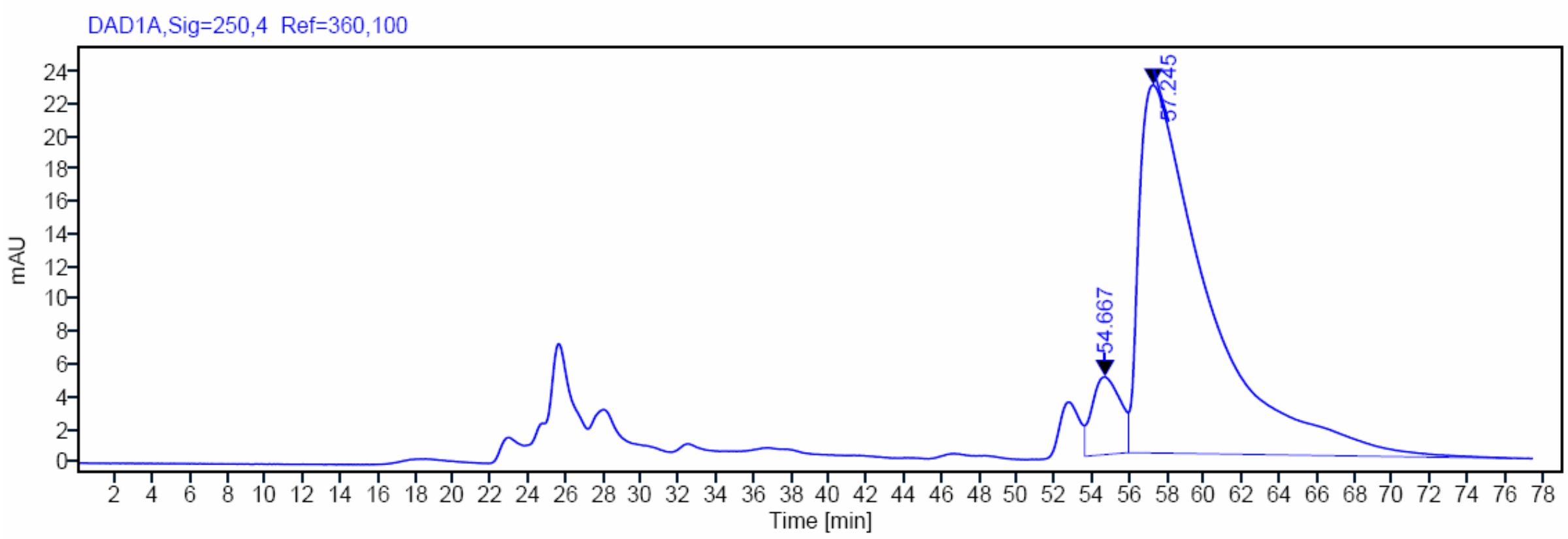

Signal: DAD1A,Sig=250,4 Ref=360,100

RT [min]	Width [min]	Area	Height	Area%
54.667	2.33	502.73	4.77	8.04
57.245	20.39	5748.19	22.58	91.96
	**Sum**	**6250.92**		

10. NMR Spectra

Figure S52. ^{1}**H NMR** (500 MHz, $CDCl_3$, 298 K) of **L2**.

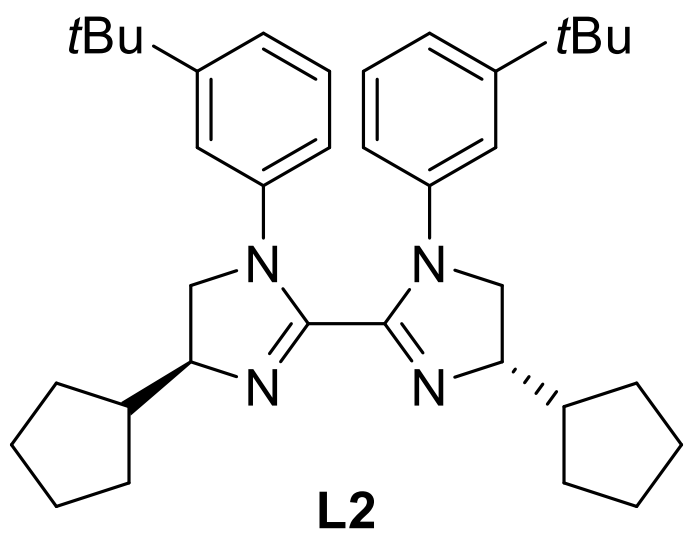

L2

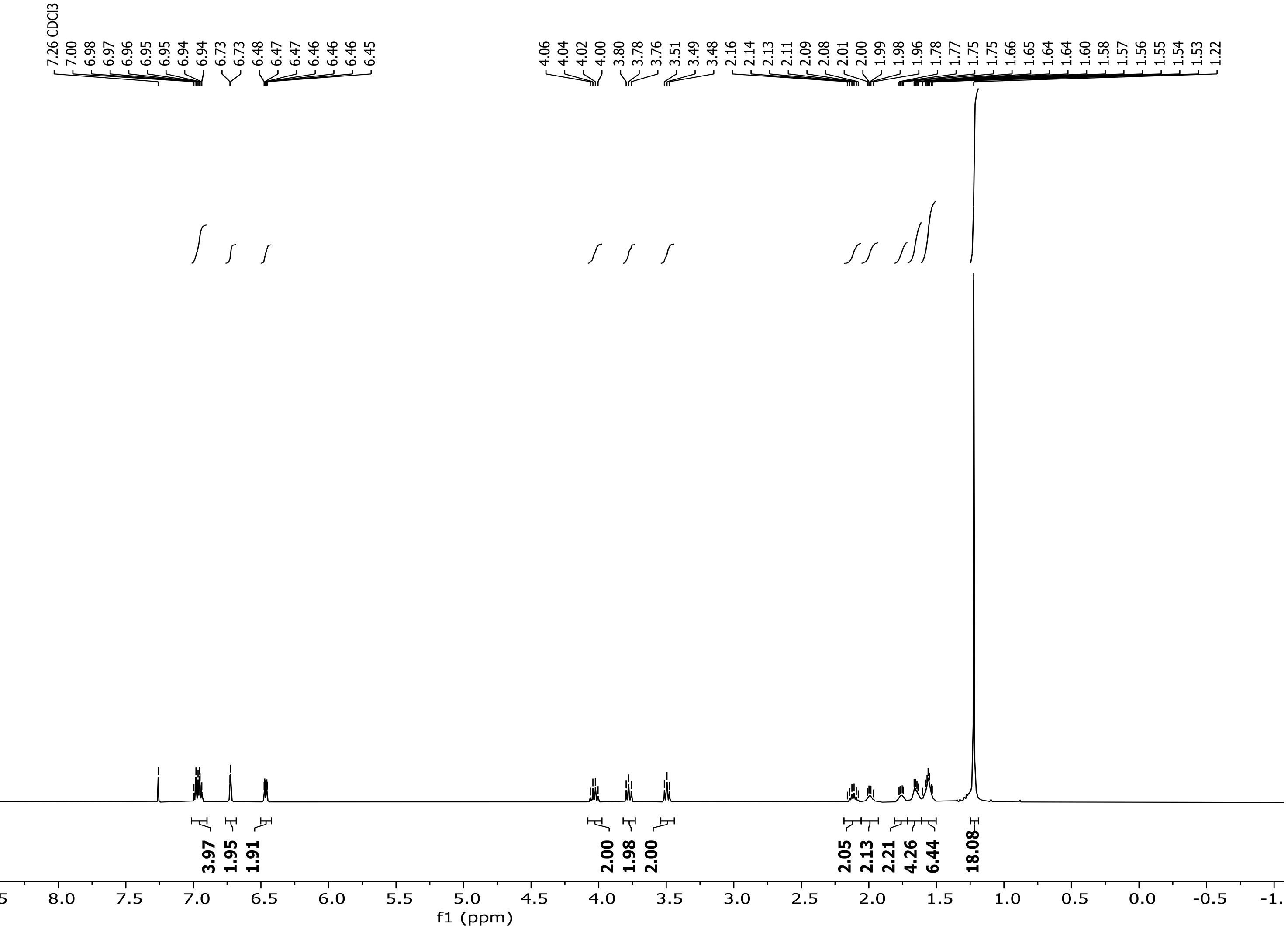

Figure S53. **^{1}H NMR** (500 MHz, $CDCl_3$, 298 K) of *rac*-**1a**.

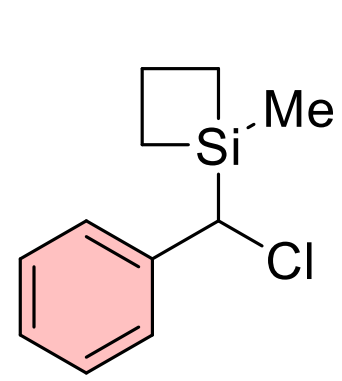

rac-**1a**
$C_{11}H_{15}ClSi$
M_W = 210.78

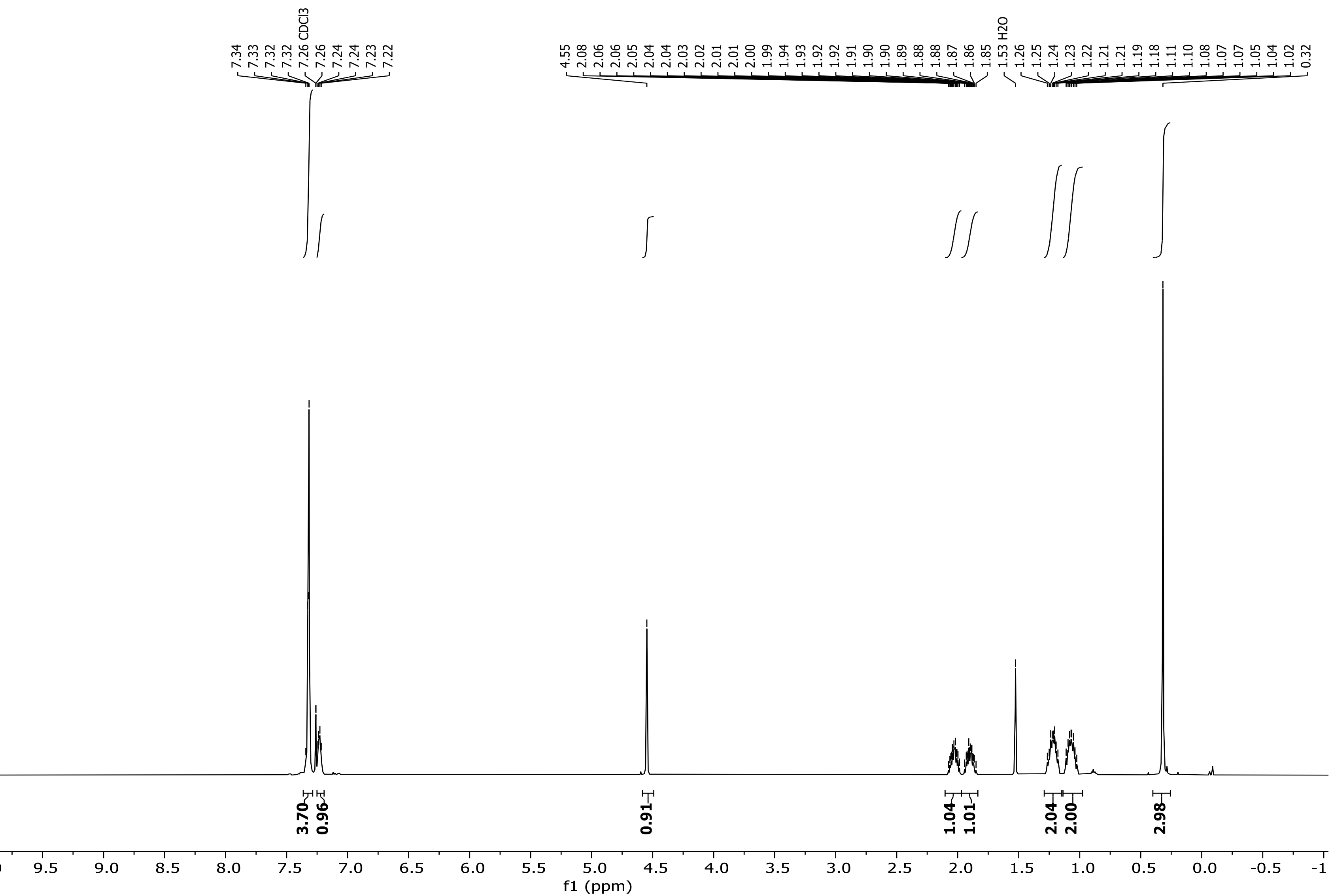

Figure S54. **^{13}C NMR** (125 MHz, $CDCl_3$, 298 K) of *rac*-**1a**.

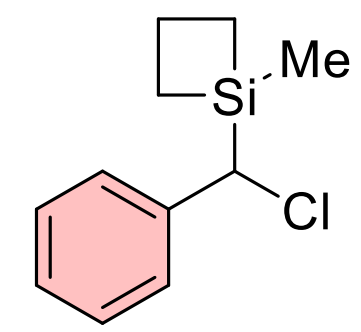

rac-**1a**
$C_{11}H_{15}ClSi$
M_W = 210.78

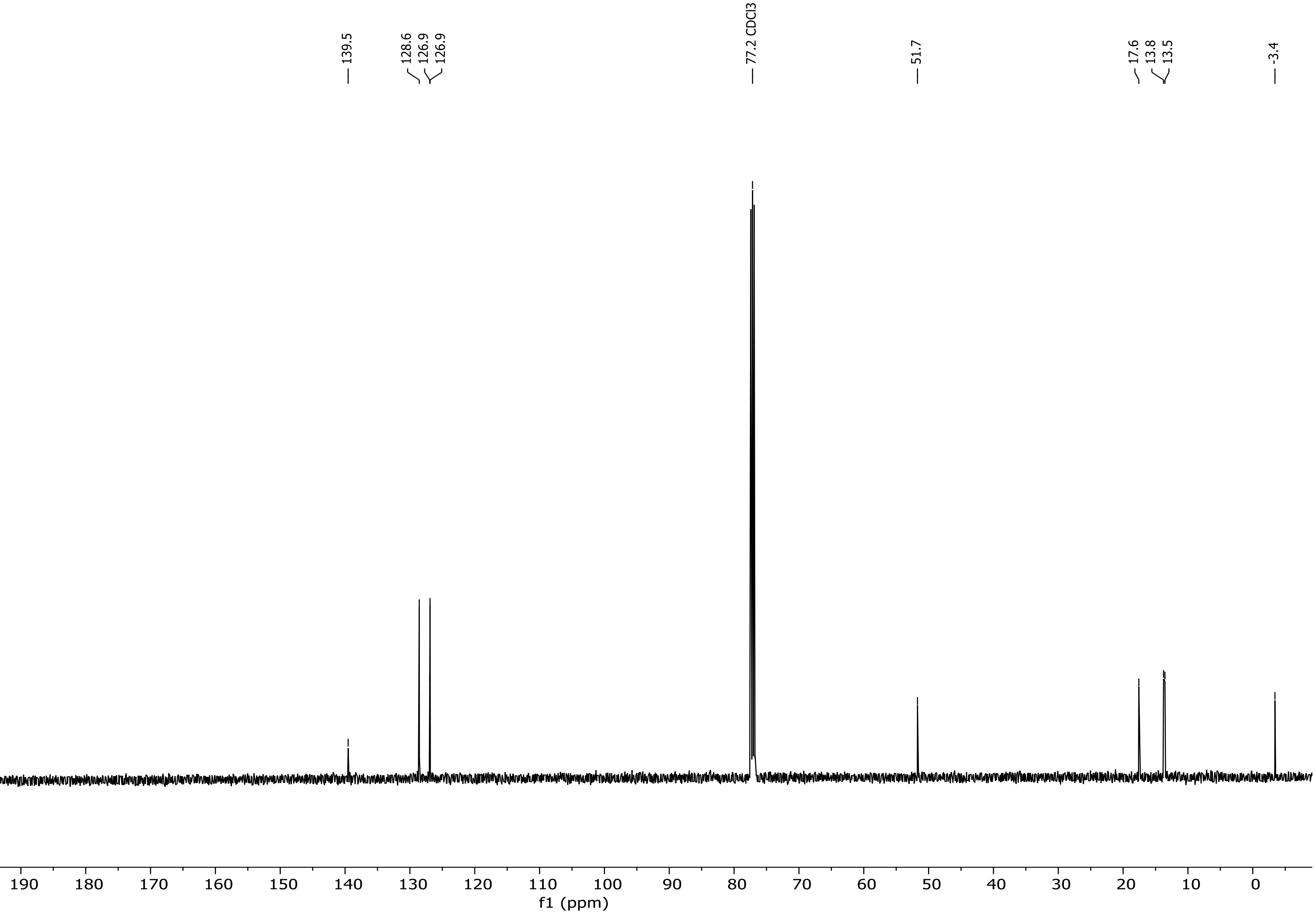

Figure S55. ^{29}Si NMR ($^{1}H/^{29}Si$ HMQC, 99 MHz, $CDCl_3$, optimized for *J* = 7 Hz) of *rac*-**1a.**

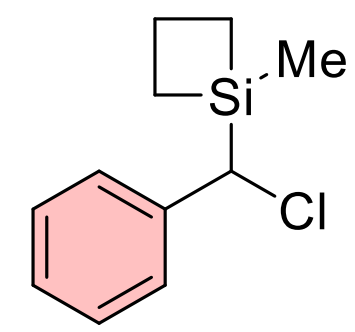

rac-**1a**
$C_{11}H_{15}ClSi$
M_W = 210.78

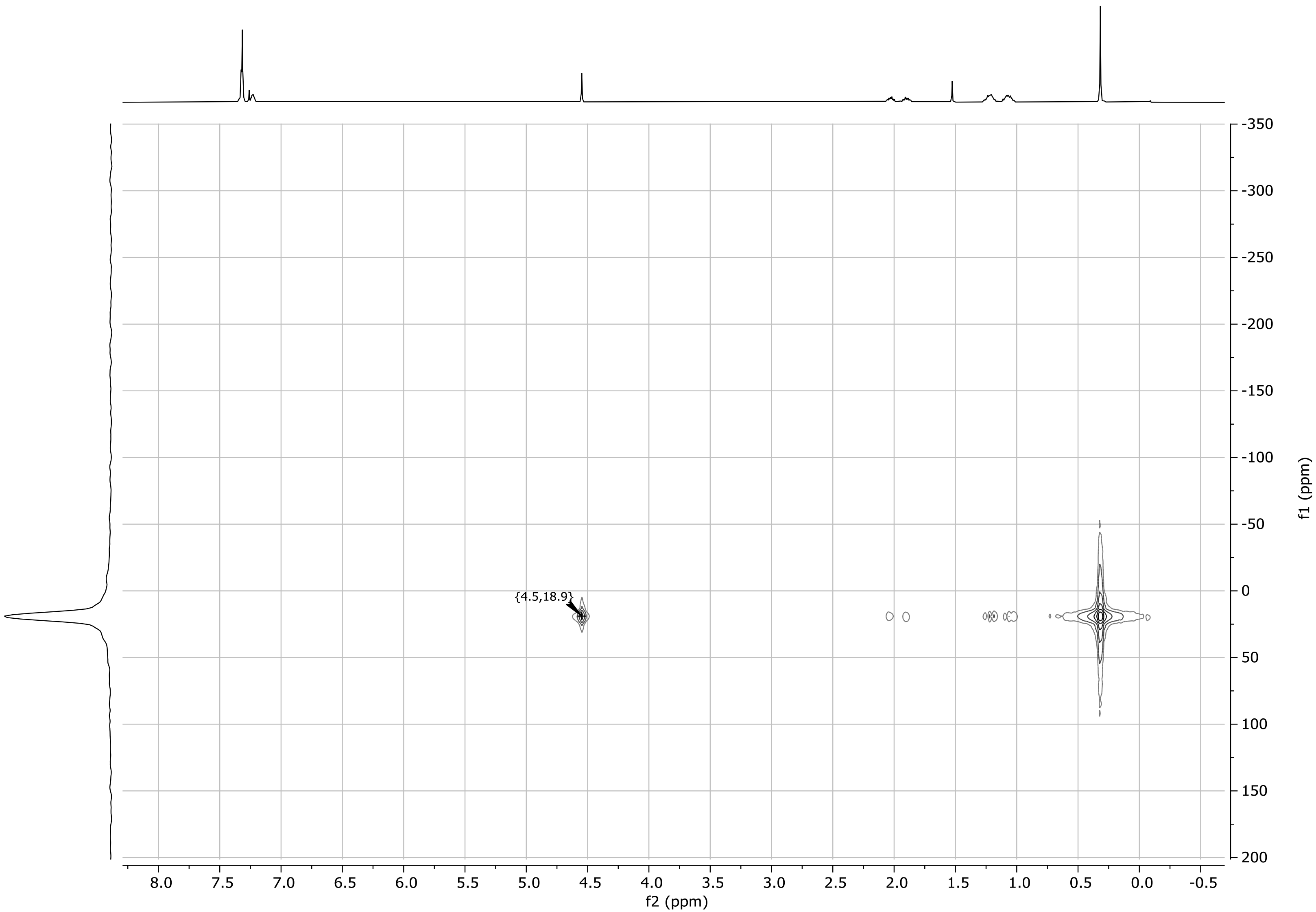

Figure S56. **¹H NMR** (500 MHz, $CDCl_3$, 298 K) of *rac*-**1b**.

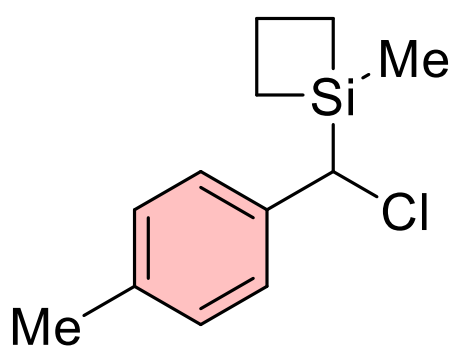

rac-**1b**
$C_{12}H_{17}ClSi$
M_W = 224.80

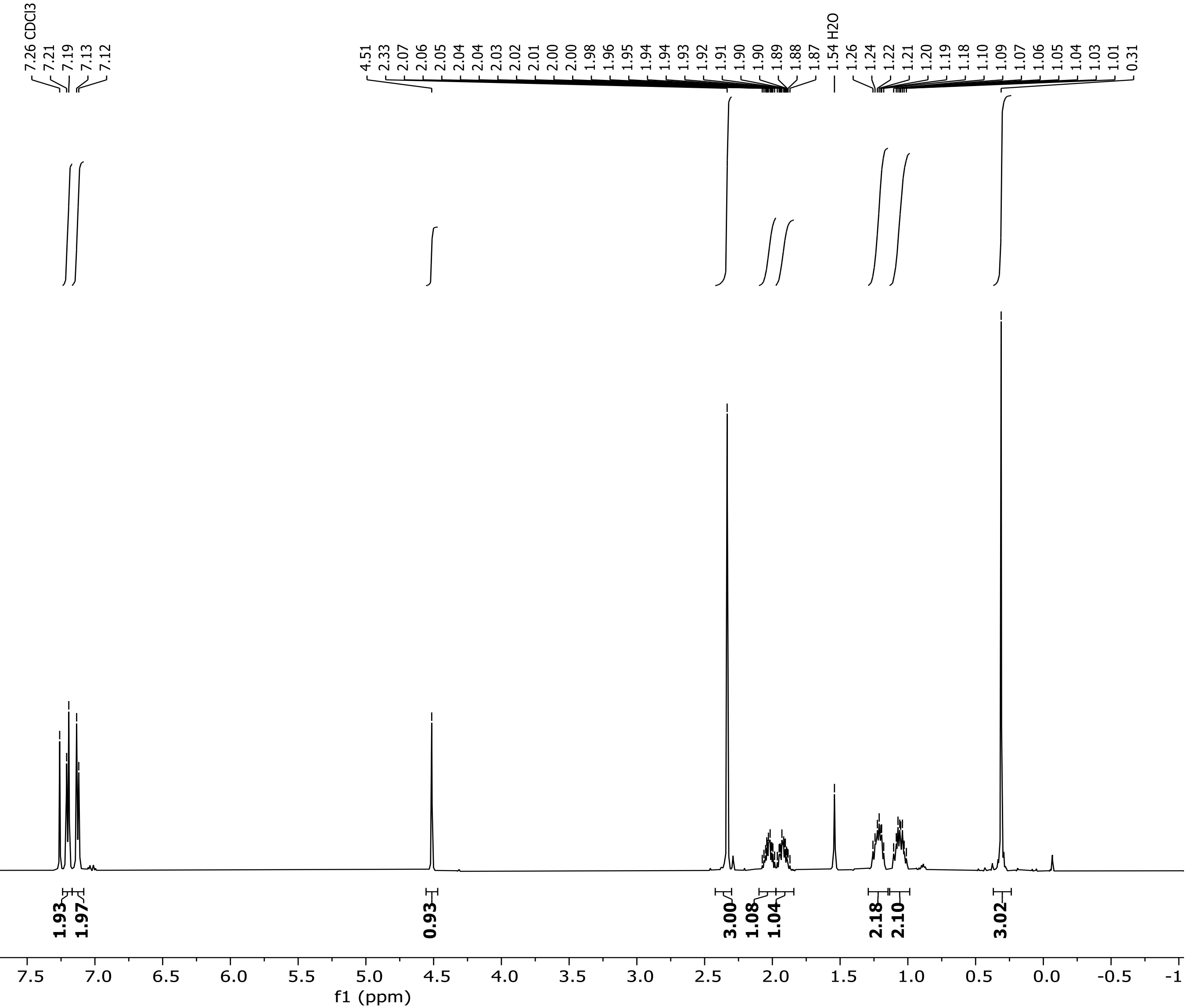

Figure S57. ^{13}**C NMR** (100 MHz, $CDCl_3$, 298 K) of *rac*-**1b**.

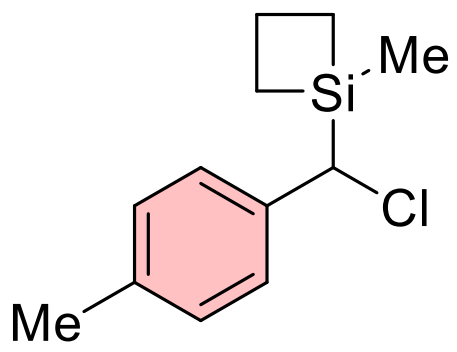

rac-**1b**

$C_{12}H_{17}ClSi$

M_W = 224.80

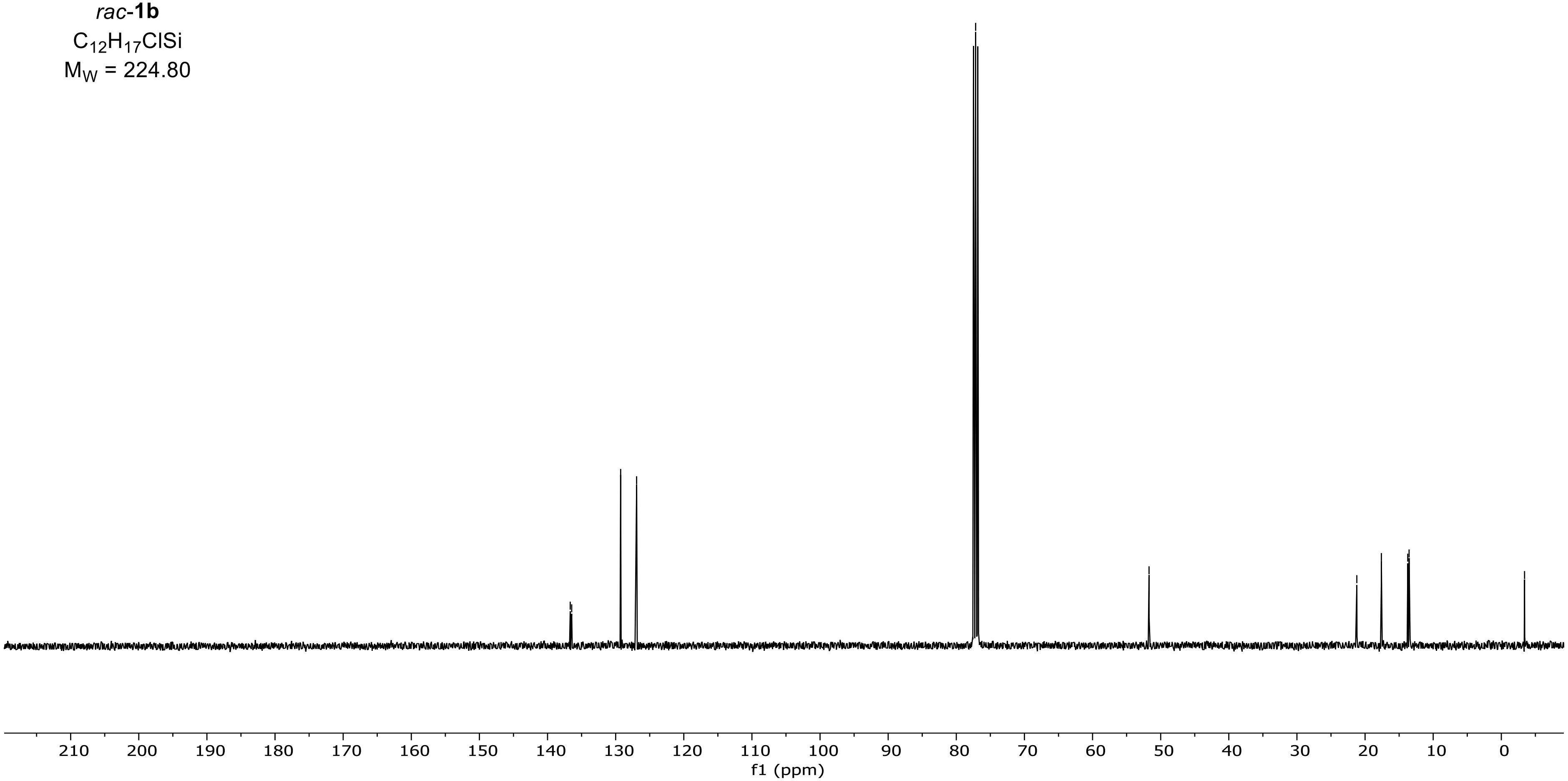

Figure S58. **^{29}Si NMR** (^{1}H/^{29}Si HMQC, 99 MHz, $CDCl_3$, optimized for J = 7 Hz) of *rac*-**1a.**

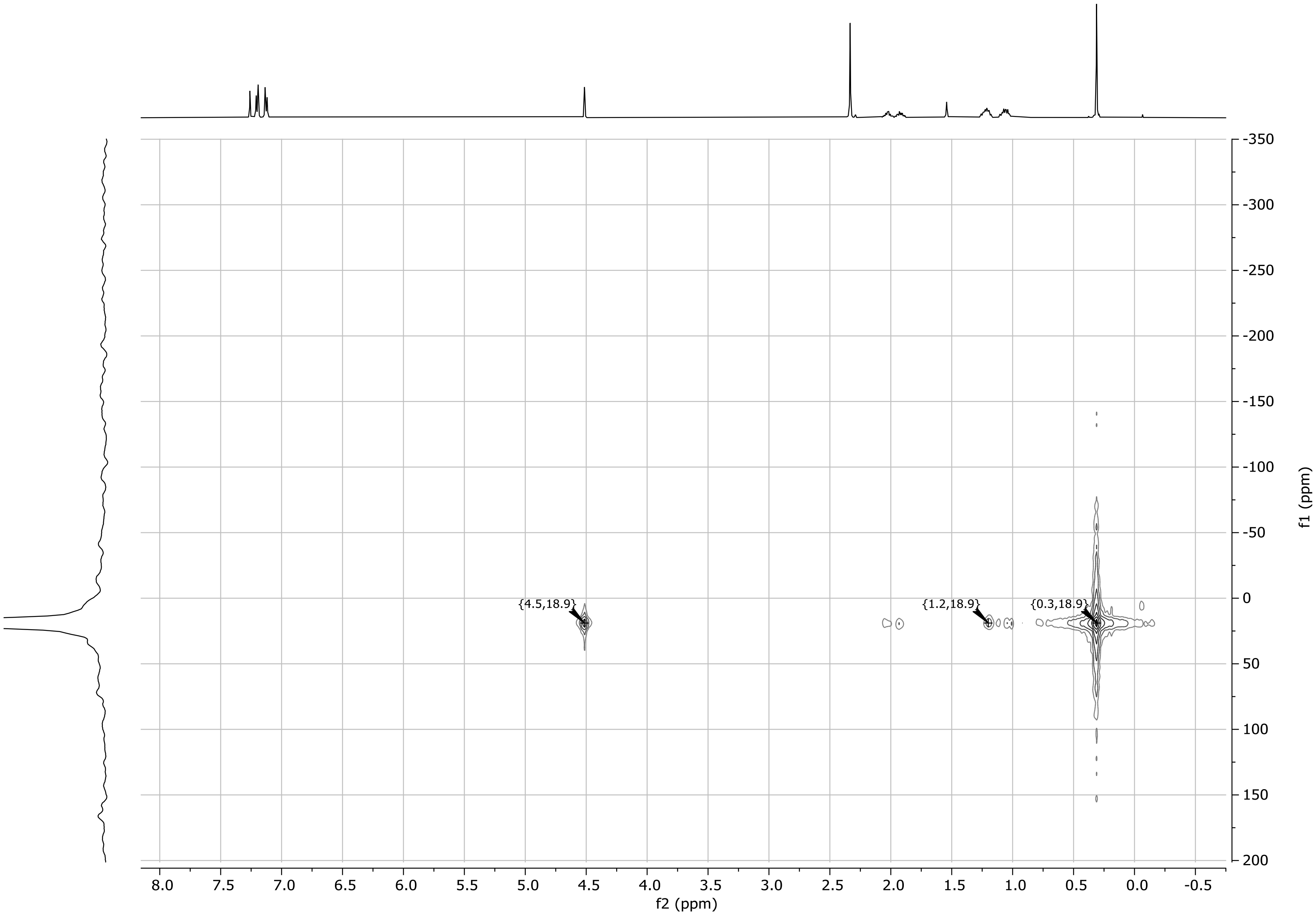

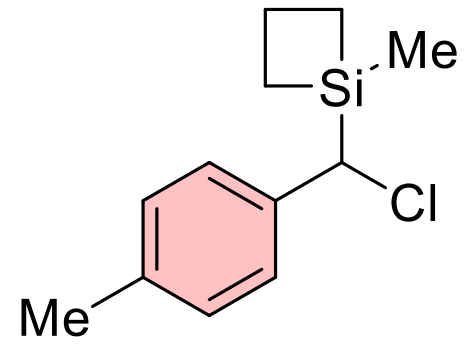

rac-**1b**

$C_{12}H_{17}ClSi$

M_W = 224.80

Figure S59. **^{1}H NMR** (500 MHz, $CDCl_3$, 298 K) of *rac*-**1c**.

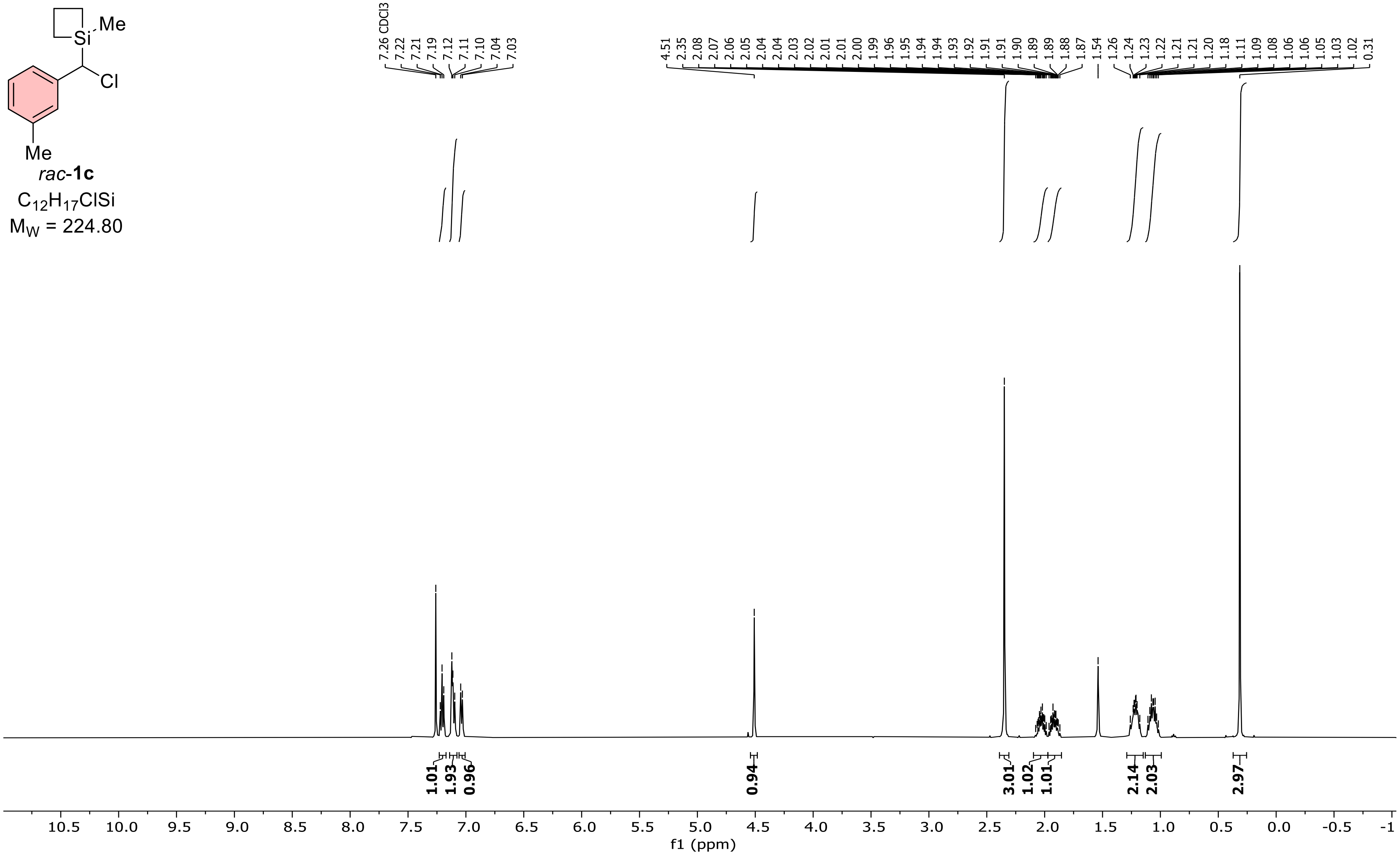

Figure S60. ^{13}**C NMR** (100 MHz, $CDCl_3$, 298 K) of *rac*-**1c**.

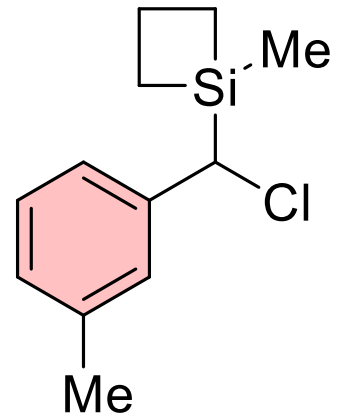

rac-**1c**
$C_{12}H_{17}ClSi$
M_W = 224.80

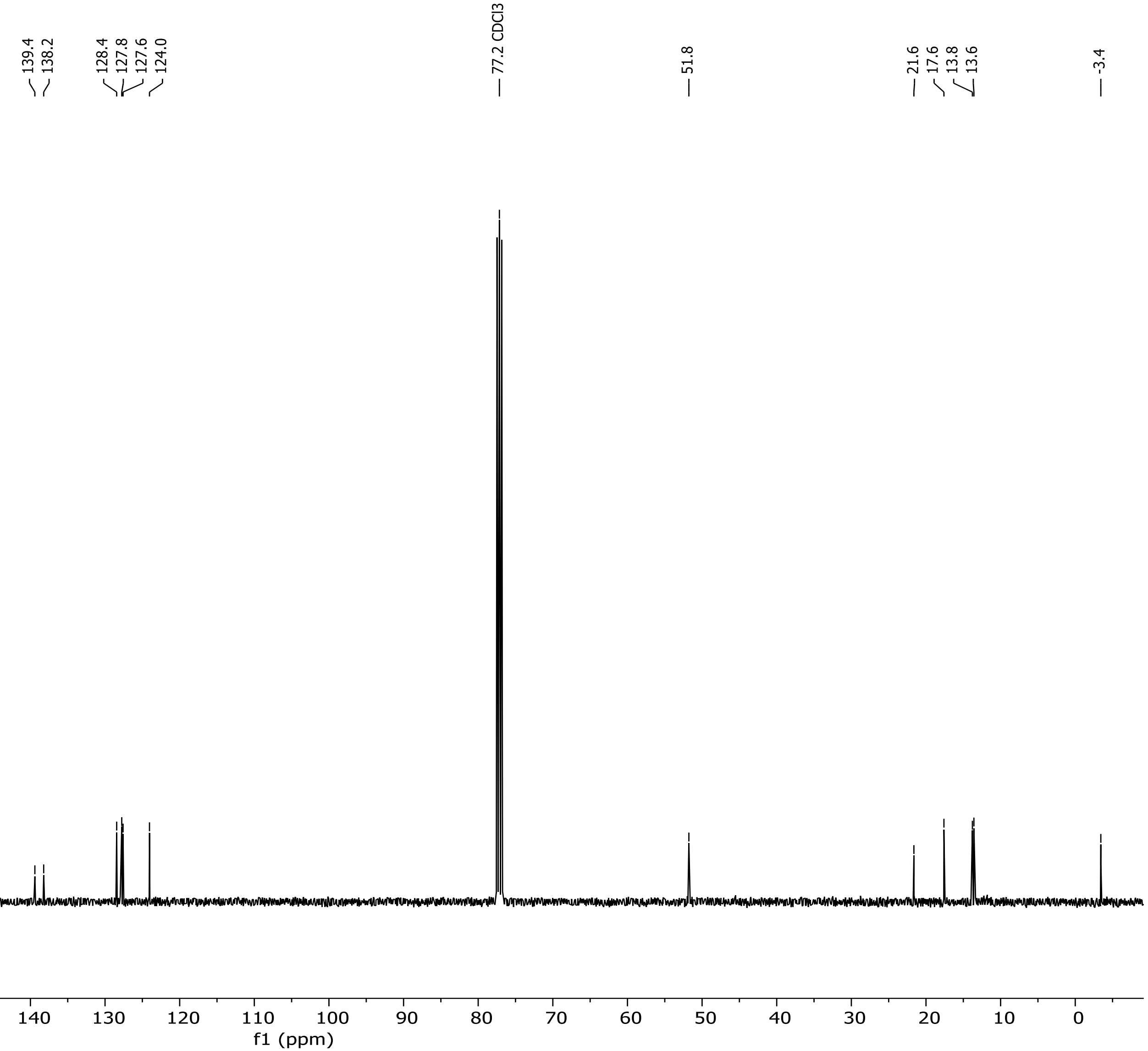

Figure S61. ^{29}Si NMR (^{1}H/^{29}Si HMQC, 99 MHz, $CDCl_3$, optimized for *J* = 7 Hz) of *rac*-**1c.**

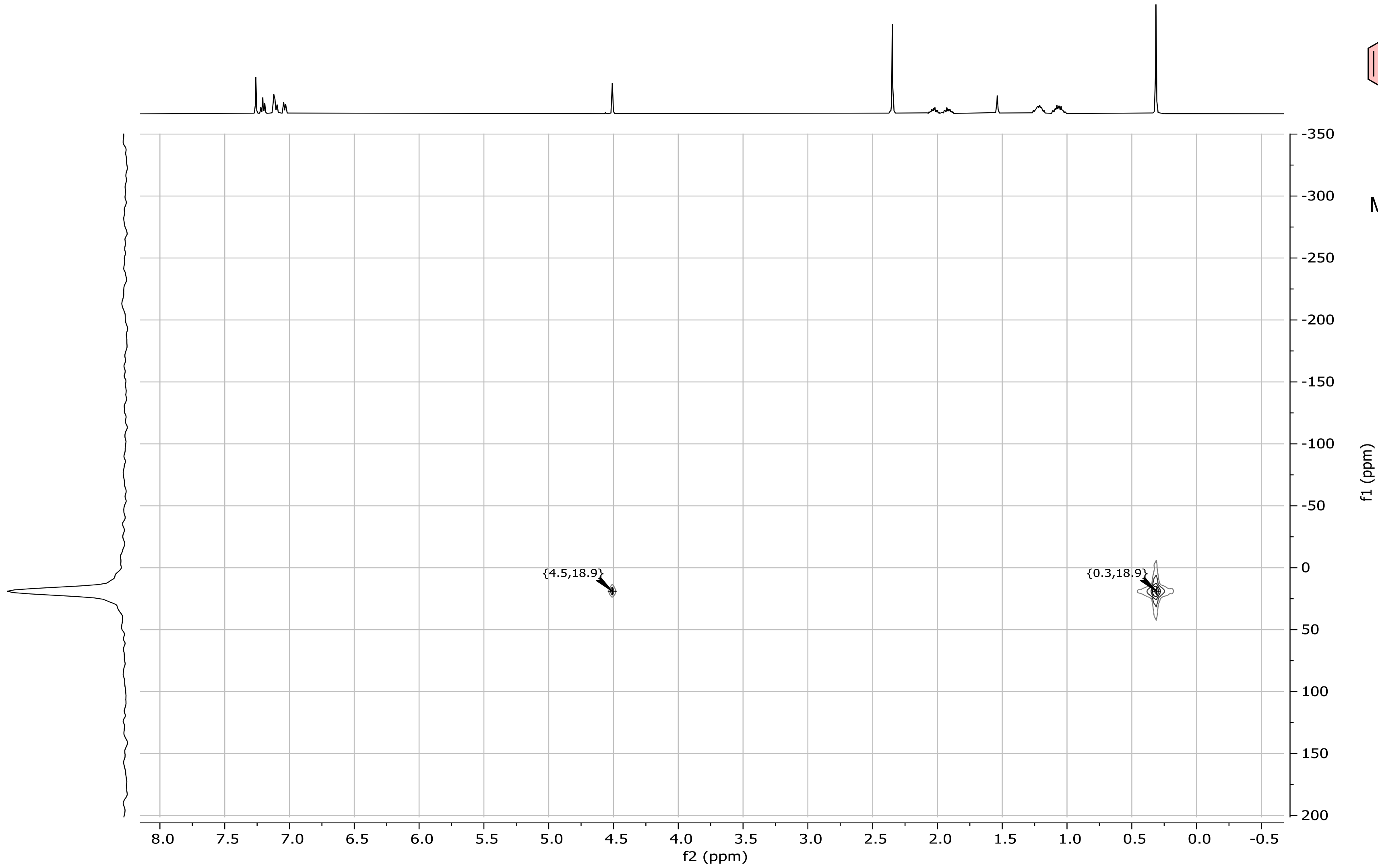

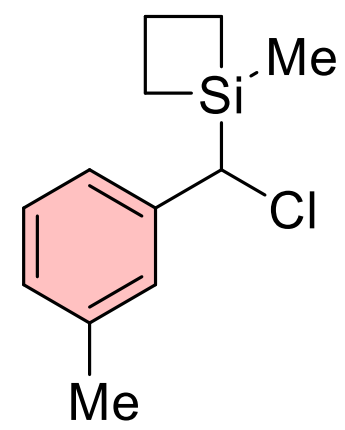

rac-**1c**

$C_{12}H_{17}ClSi$

M_W = 224.80

Figure S62. **^{1}H NMR** (500 MHz, $CDCl_3$, 298 K) of *rac*-**1d**.

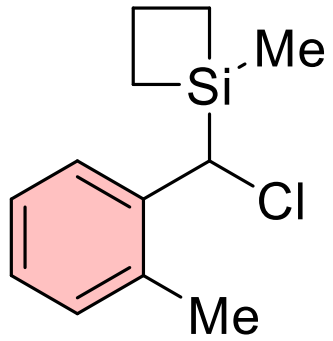

rac-**1d**
$C_{12}H_{17}ClSi$
M_W = 224.80

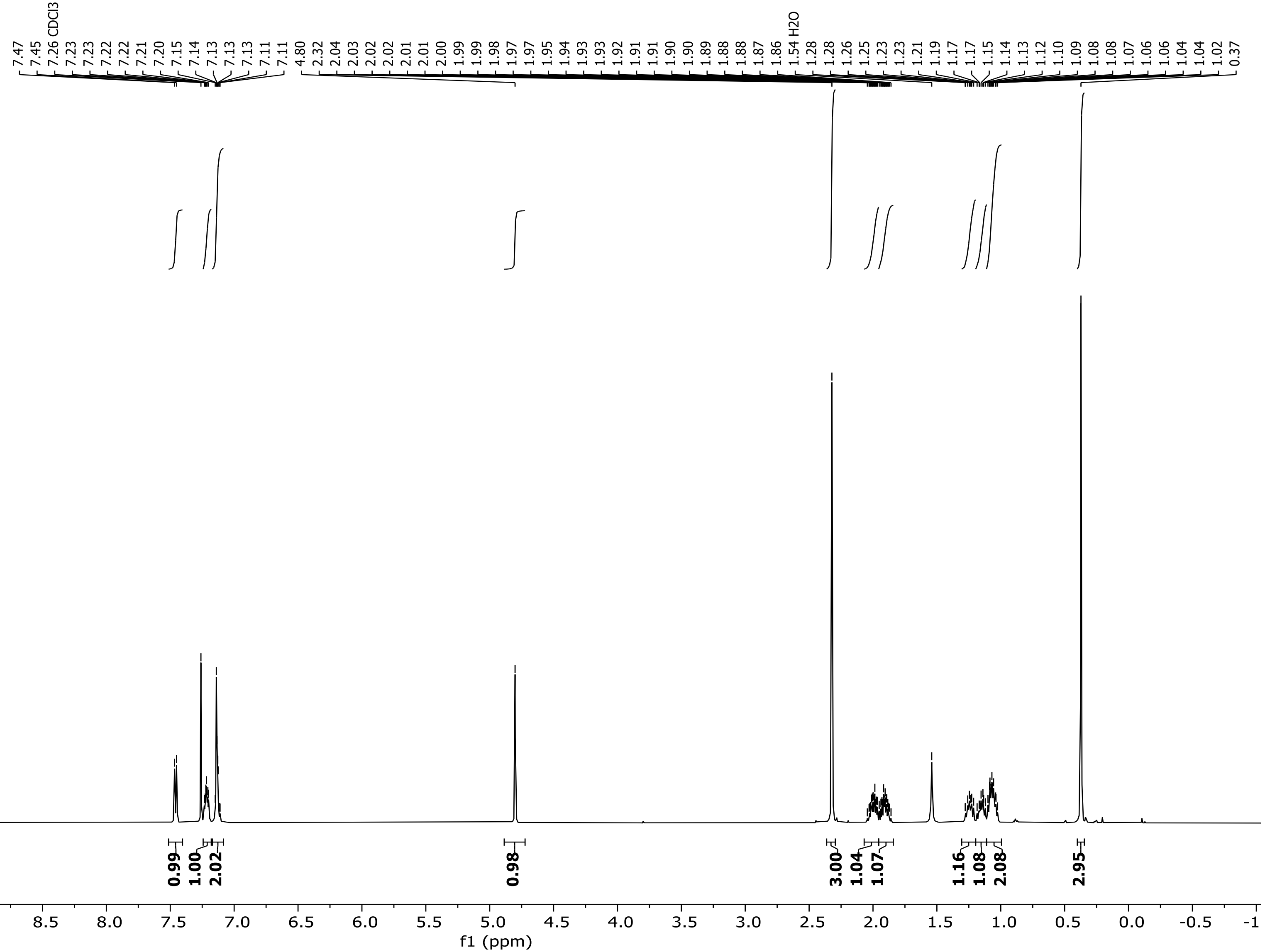

Figure S63. ^{13}**C NMR** (125 MHz, $CDCl_3$, 298 K) of *rac*-**1d**.

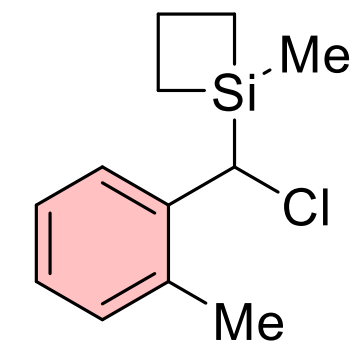

rac-**1d**

$C_{12}H_{17}ClSi$

M_W = 224.80

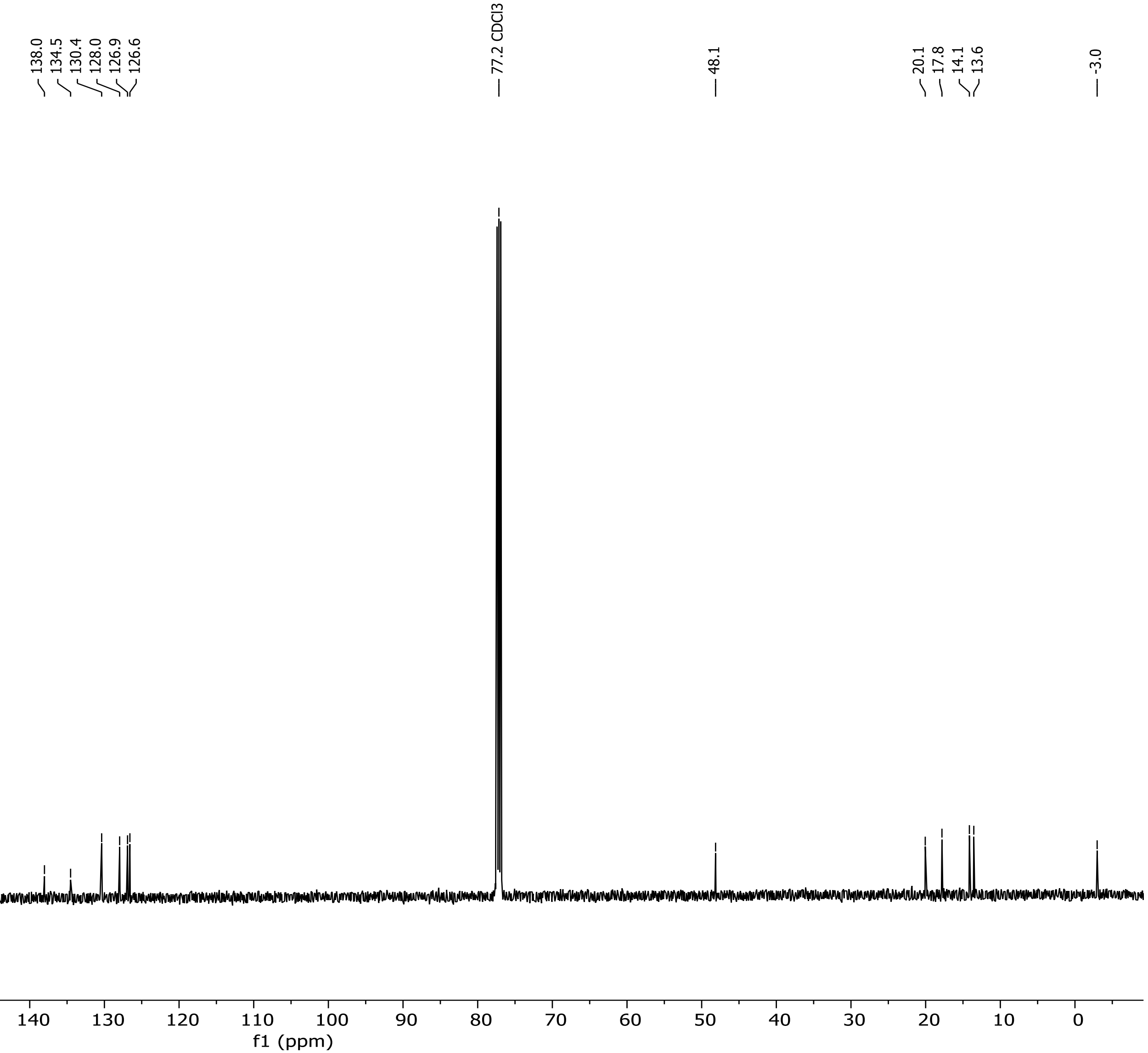

Figure S64. ^{29}**Si NMR** ($^1H/^{29}Si$ HMQC, 99 MHz, $CDCl_3$, optimized for J = 7 Hz) of *rac*-**1d.**

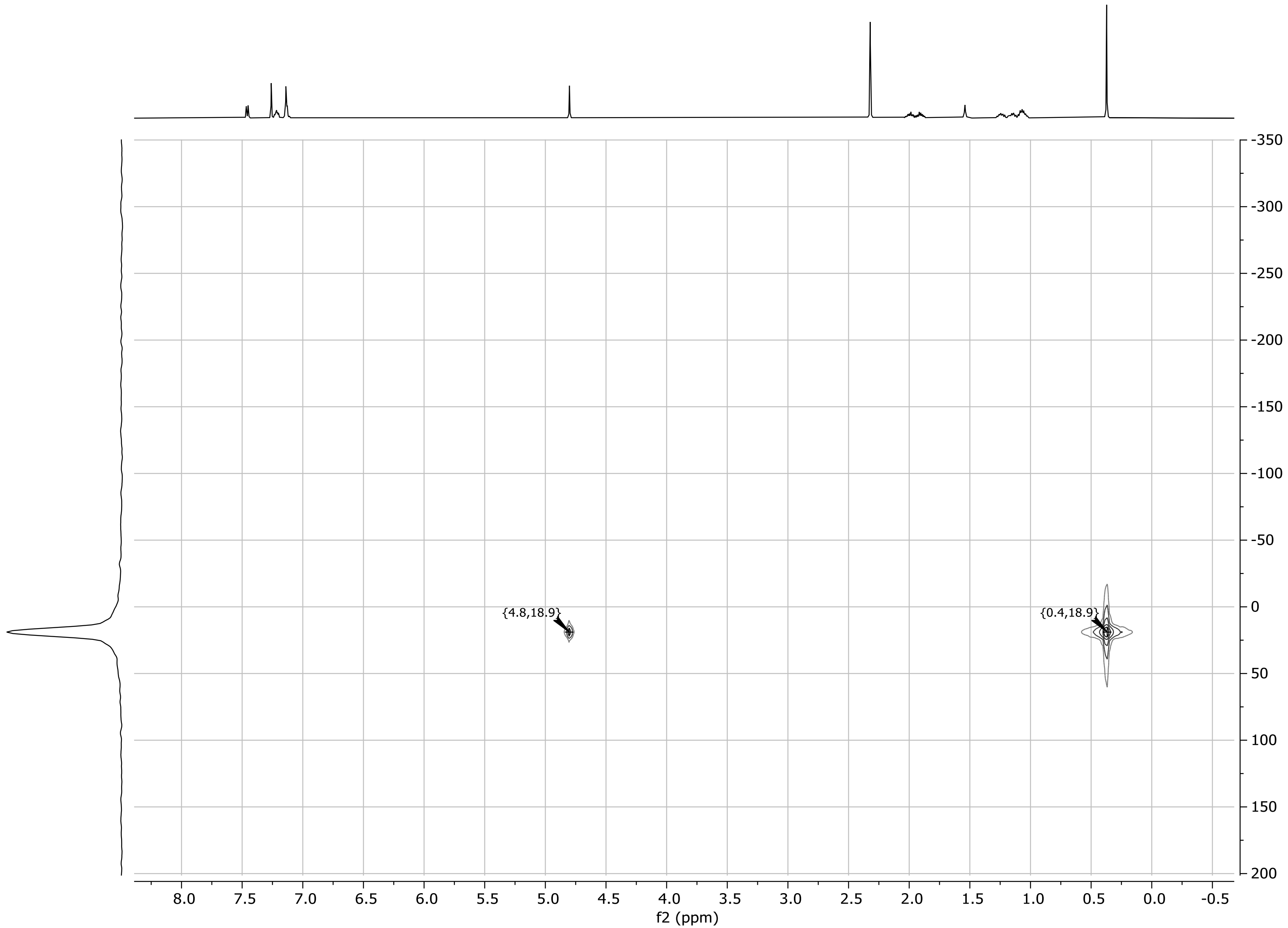

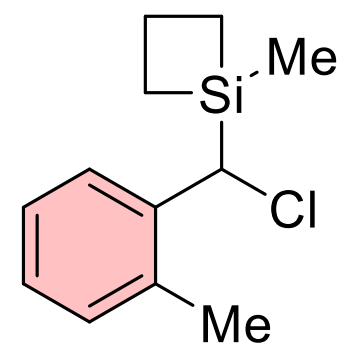

rac-**1d**

$C_{12}H_{17}ClSi$

M_W = 224.80

Figure S65. ^{1}**H NMR** (500 MHz, $CDCl_3$, 298 K) of *rac*-**1e**.

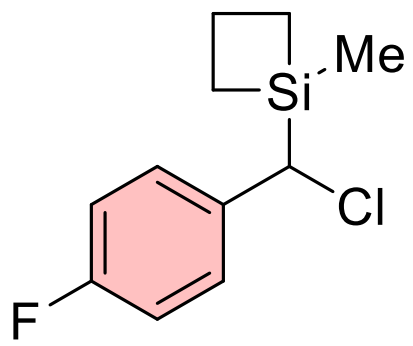

rac-**1e**
$C_{11}H_{14}ClFSi$
M_W = 228.77

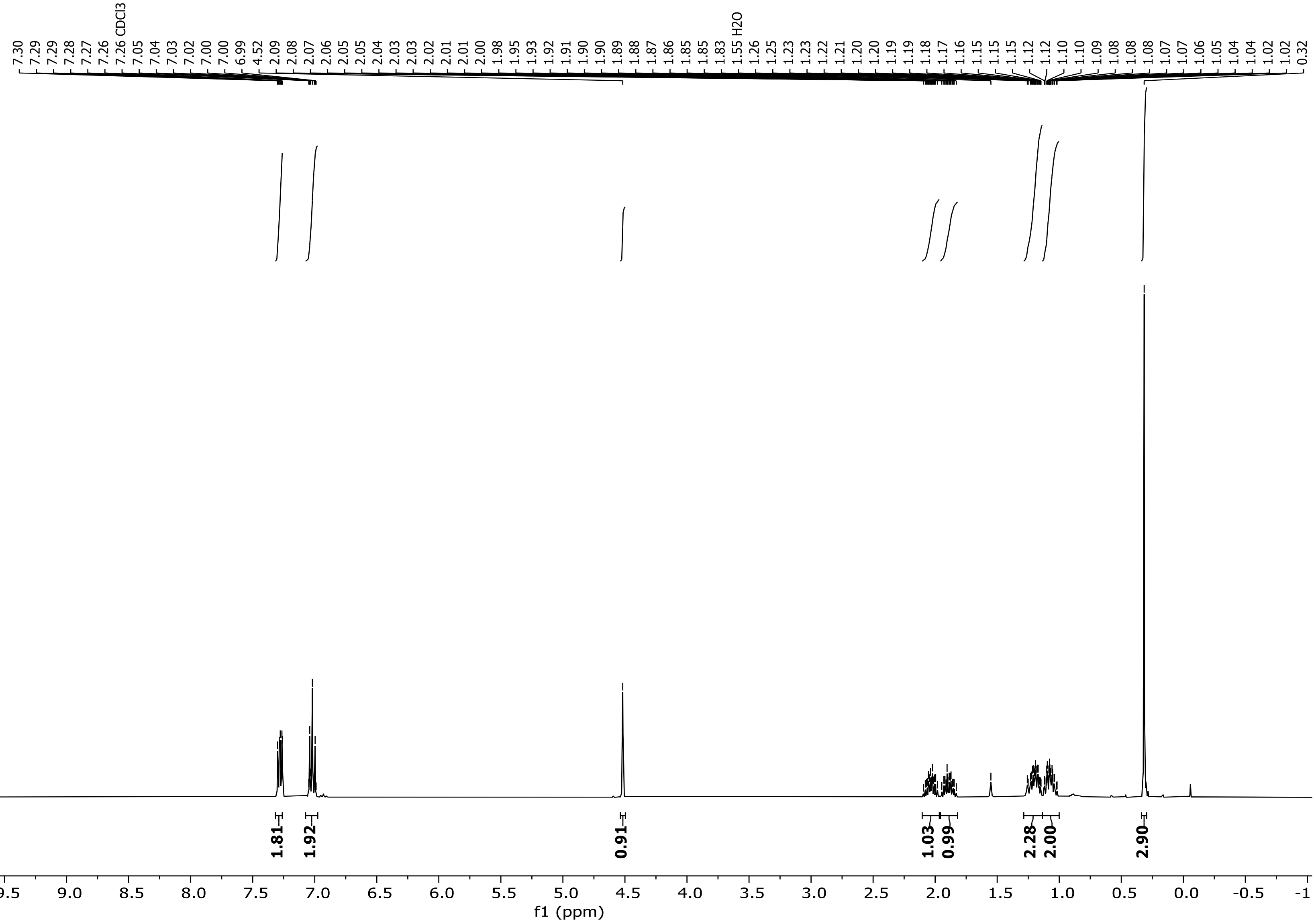

Figure S66. **^{13}C NMR** (125 MHz, $CDCl_3$, 298 K) of *rac*-**1e**.

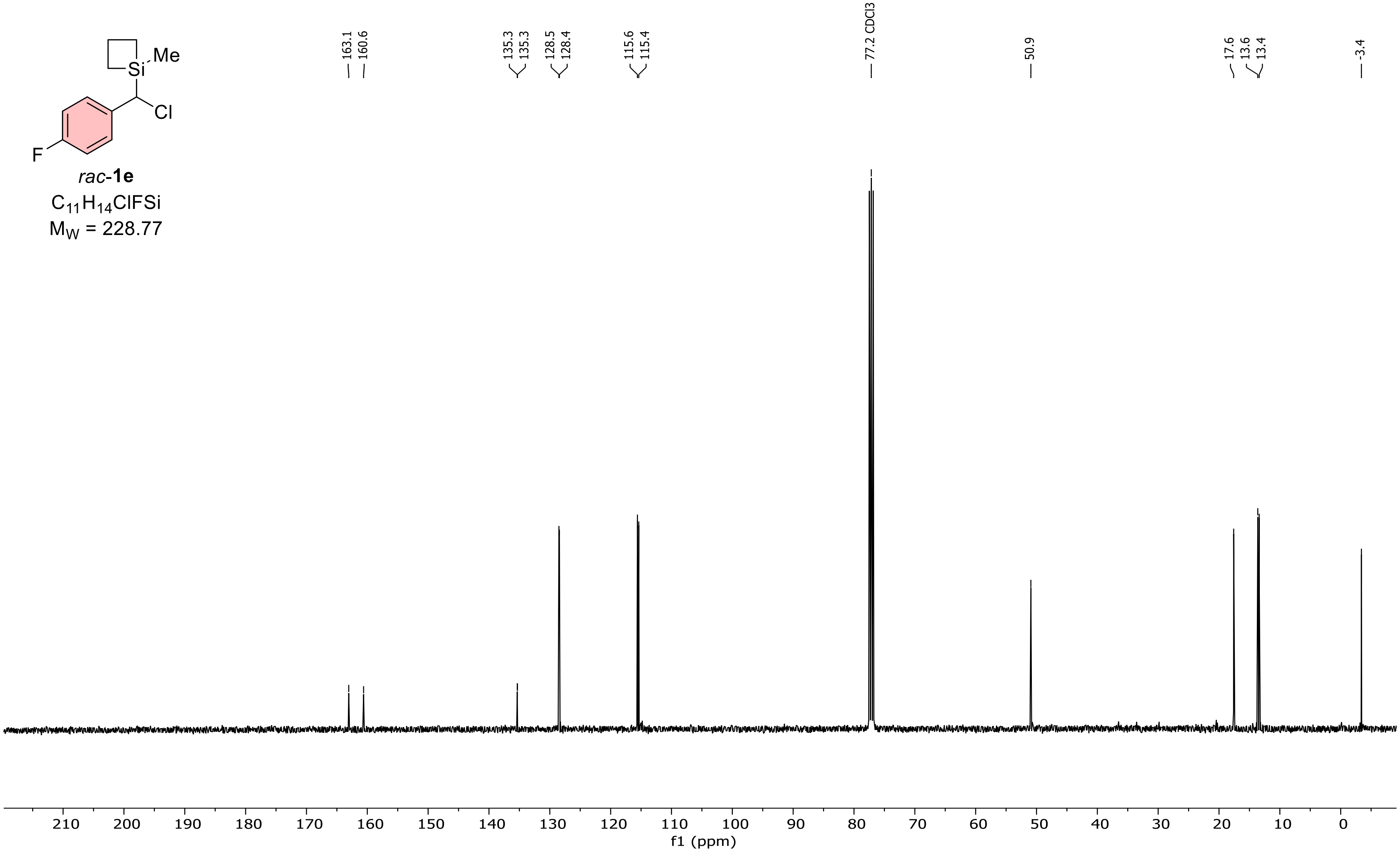

Figure S67. ^{29}Si NMR ($^{1}H/^{29}Si$ HMQC, 99 MHz, $CDCl_3$, optimized for *J* = 7 Hz) of *rac*-**1e.**

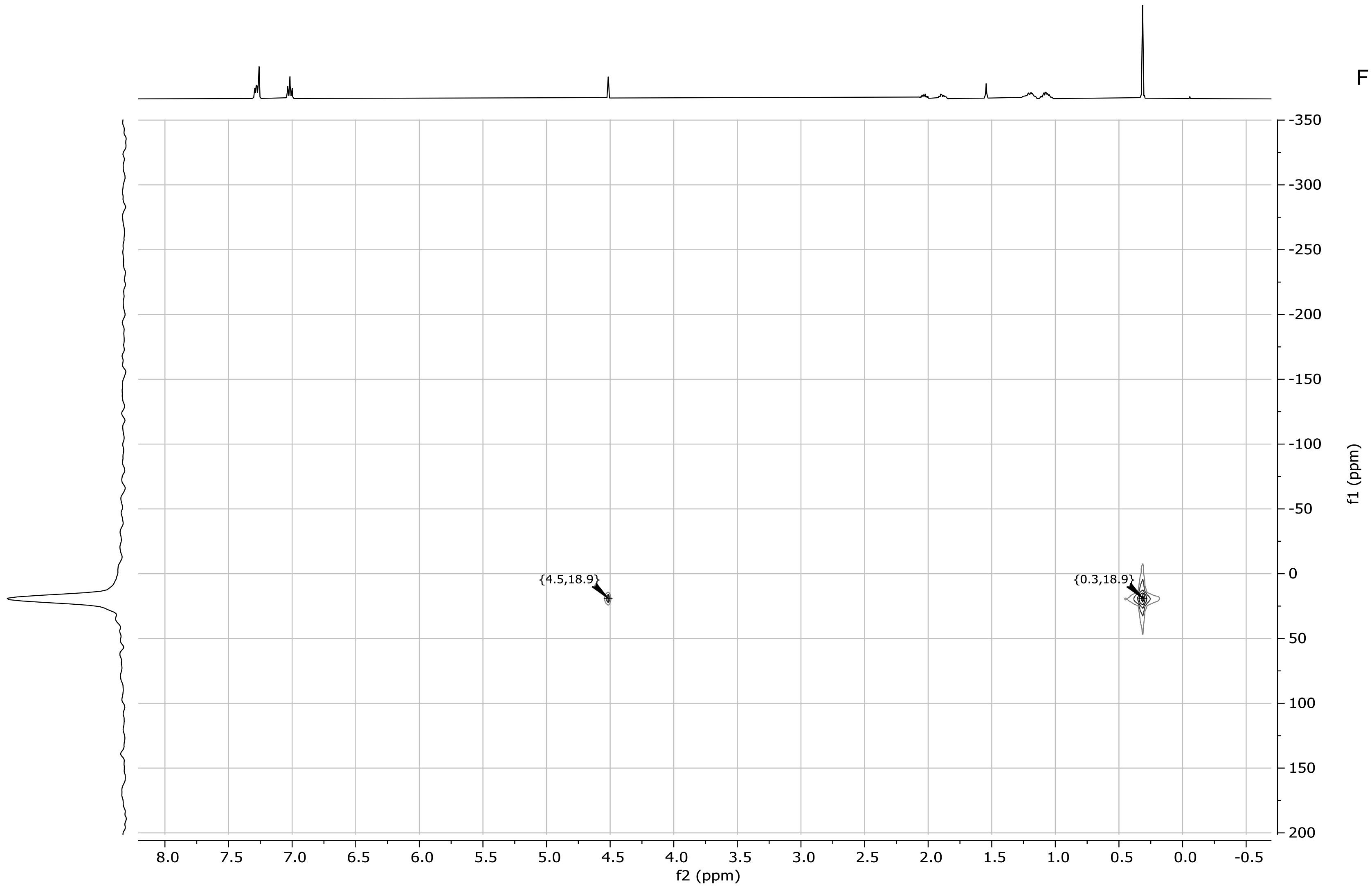

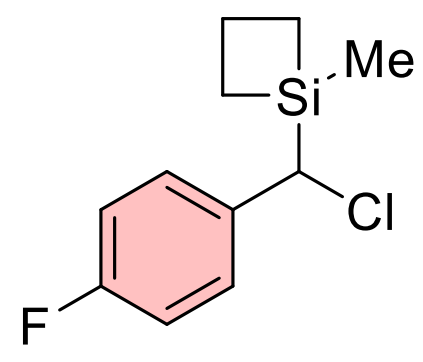

rac-**1e**
$C_{11}H_{14}ClFSi$
M_W = 228.77

Figure S68. **^{19}F NMR** (470 MHz, $CDCl_3$, 298 K) of *rac*-**1e.**

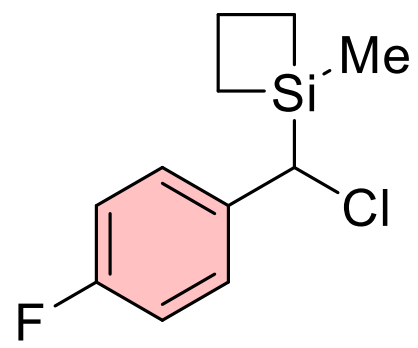

rac-**1e**
$C_{11}H_{14}ClFSi$
M_W = 228.77

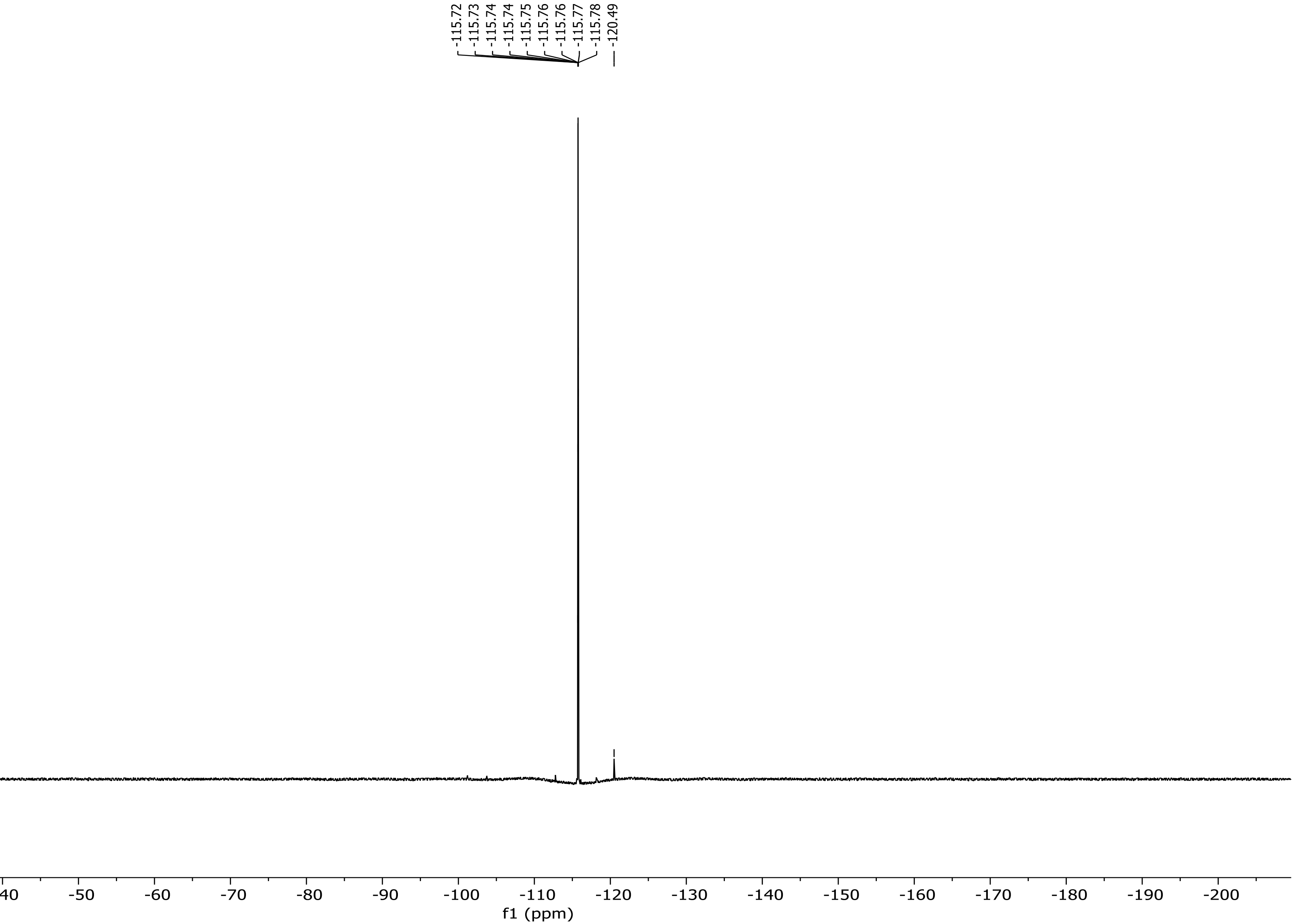

Figure S69. ^{1}**H NMR** (500 MHz, $CDCl_3$, 298 K) of *rac*-**1f**.

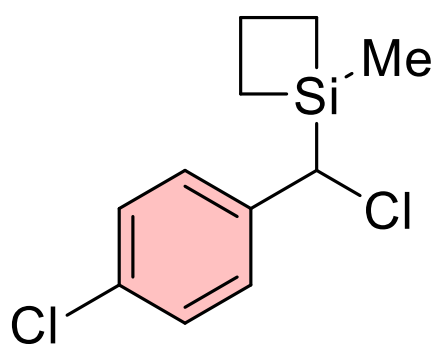

rac-**1f**

$C_{11}H_{14}Cl_2Si$

M_W = 245.22

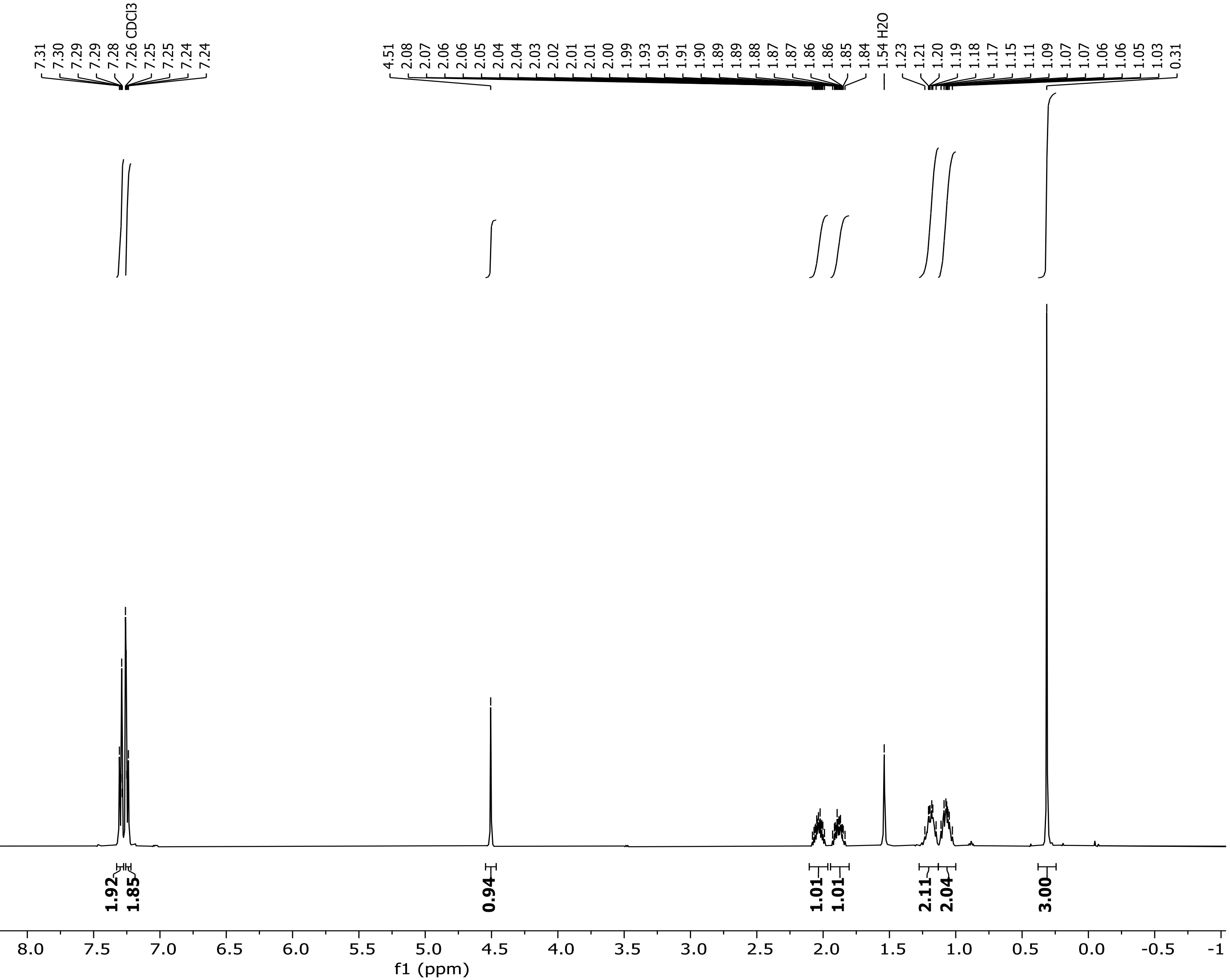

Figure S70. **^{13}C NMR** (100 MHz, $CDCl_3$, 298 K) of *rac*-**1f**.

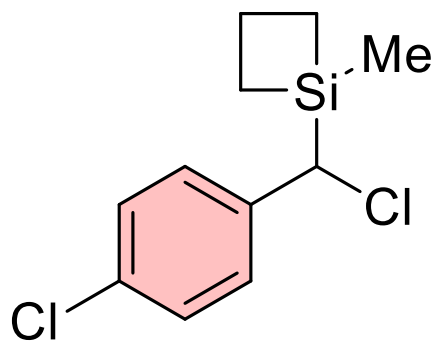

rac-**1f**
$C_{11}H_{14}Cl_2Si$
M_W = 245.22

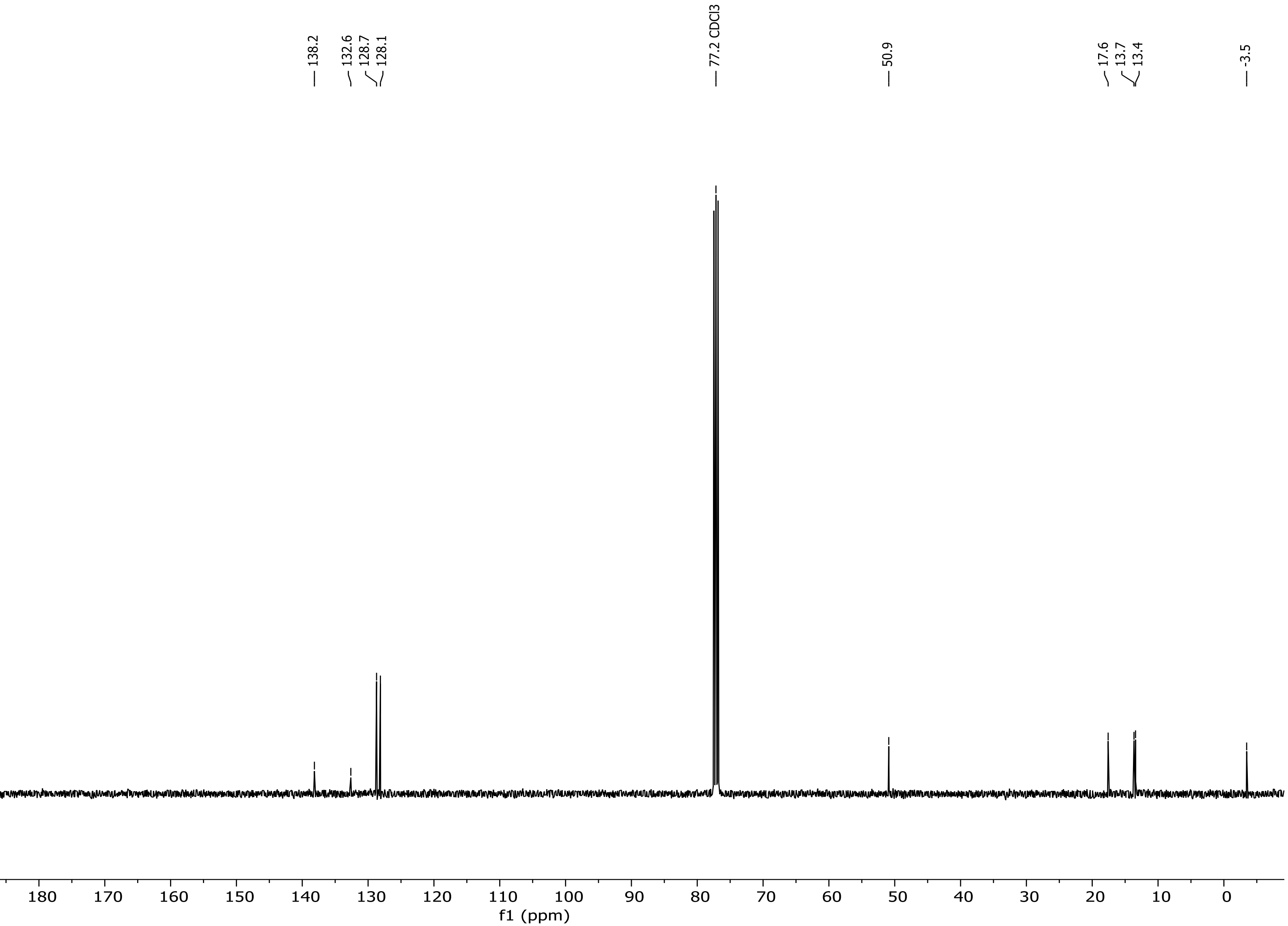

Figure S71. ^{29}**Si NMR** ($^{1}H/^{29}Si$ HMQC, 99 MHz, $CDCl_3$, optimized for J = 7 Hz) of *rac*-**1f.**

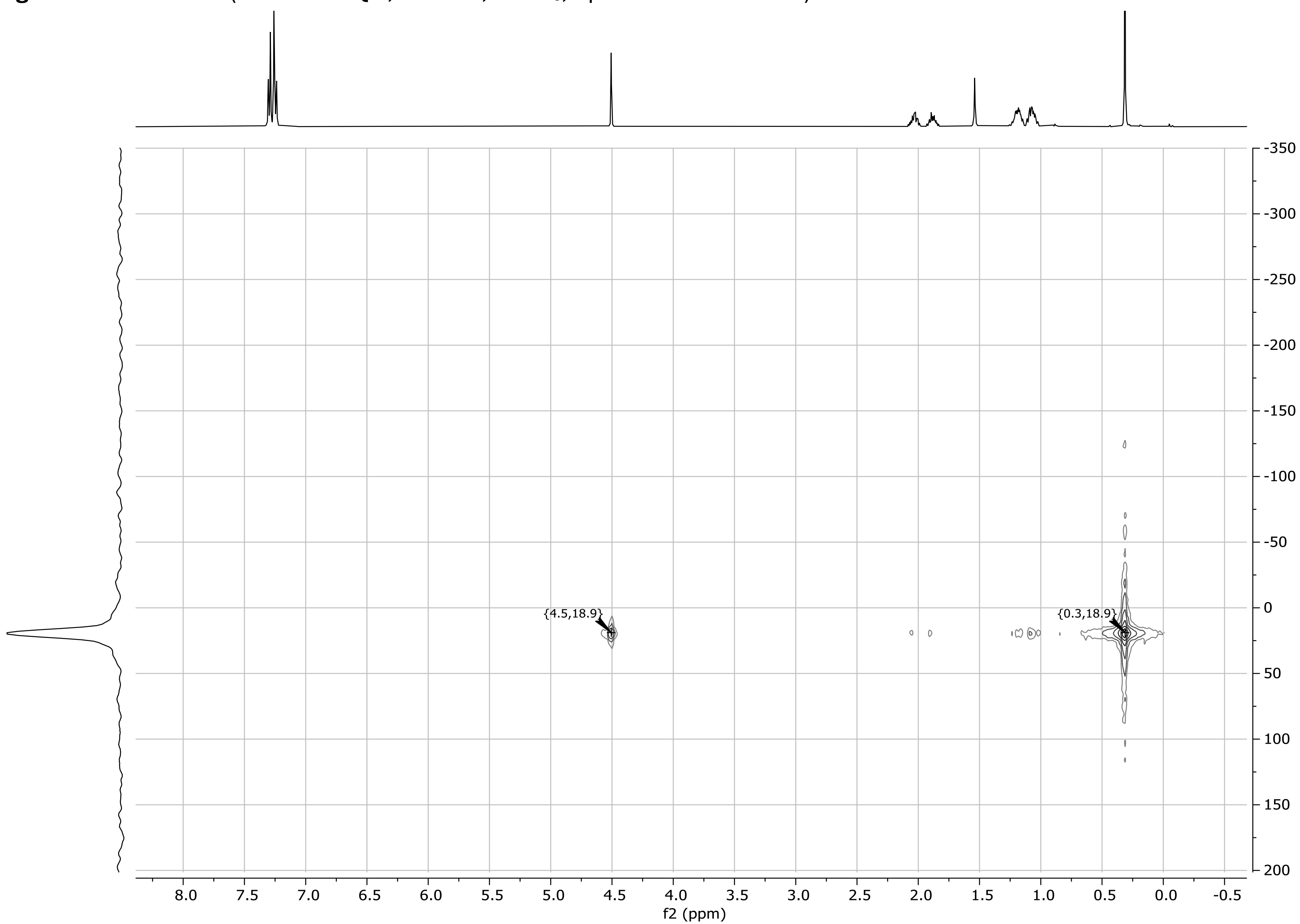

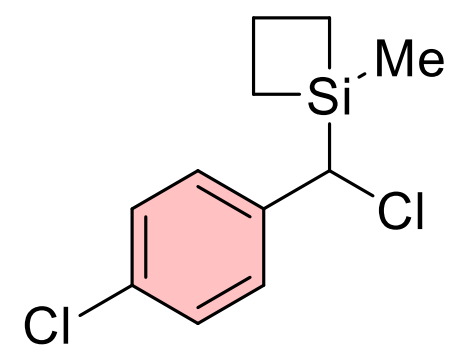

rac-**1f**
$C_{11}H_{14}Cl_2Si$
M_W = 245.22

Figure S72. [1]**H NMR** (500 MHz, $CDCl_3$, 298 K) of *rac*-**1g**.

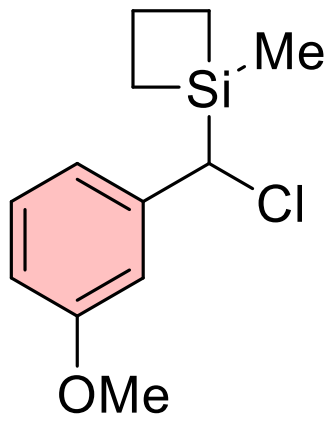

rac-**1g**
$C_{12}H_{17}ClOSi$
M_W = 240.80

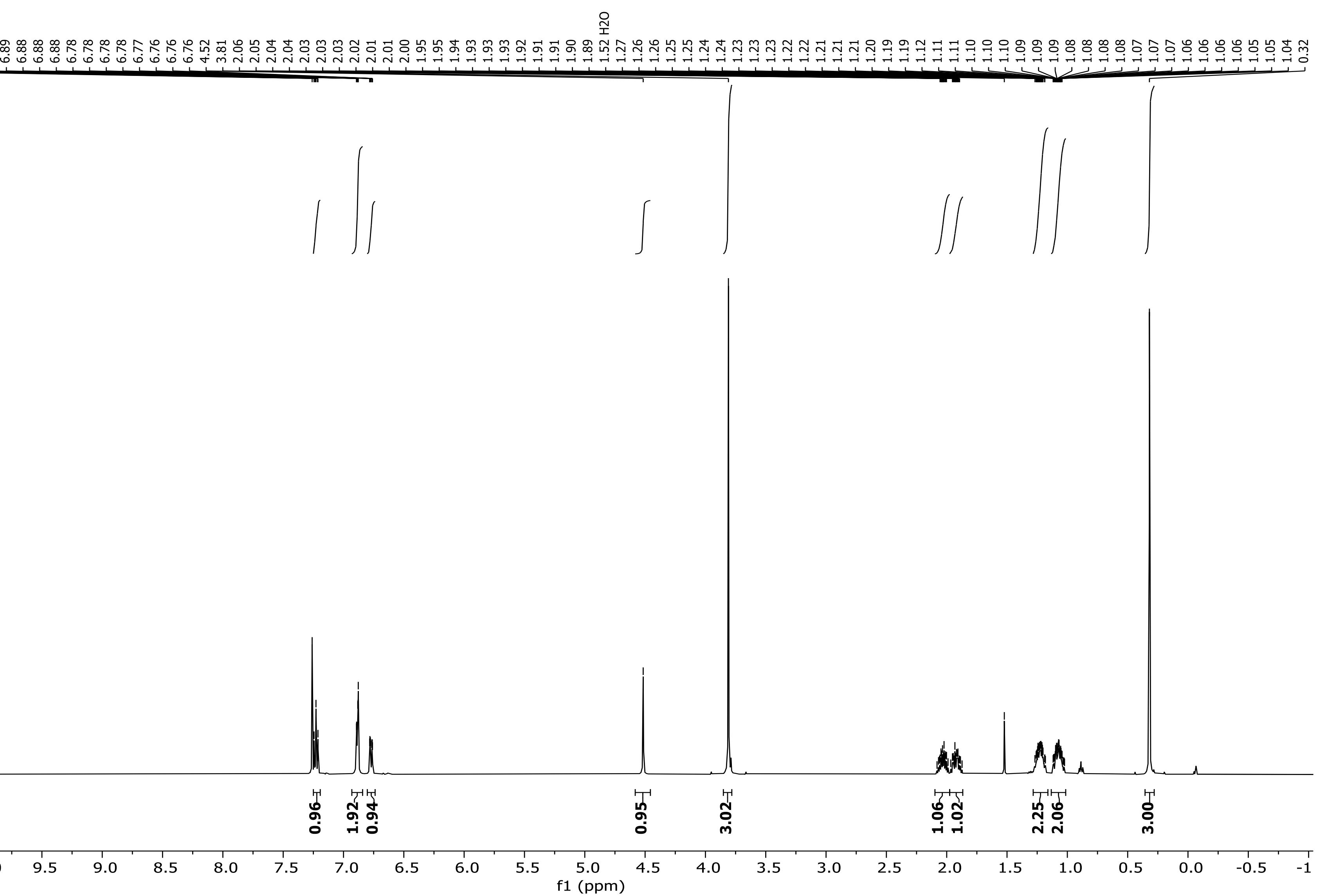

Figure S73. ^{13}**C NMR** (125 MHz, $CDCl_3$, 298 K) of *rac*-**1g**.

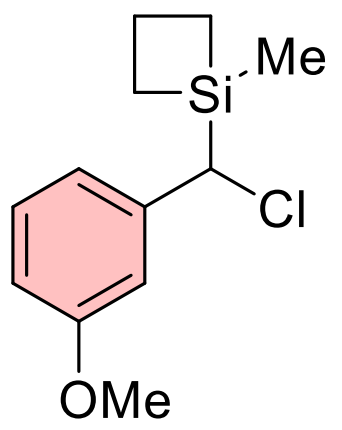

rac-**1g**
$C_{12}H_{17}ClOSi$
M_W = 240.80

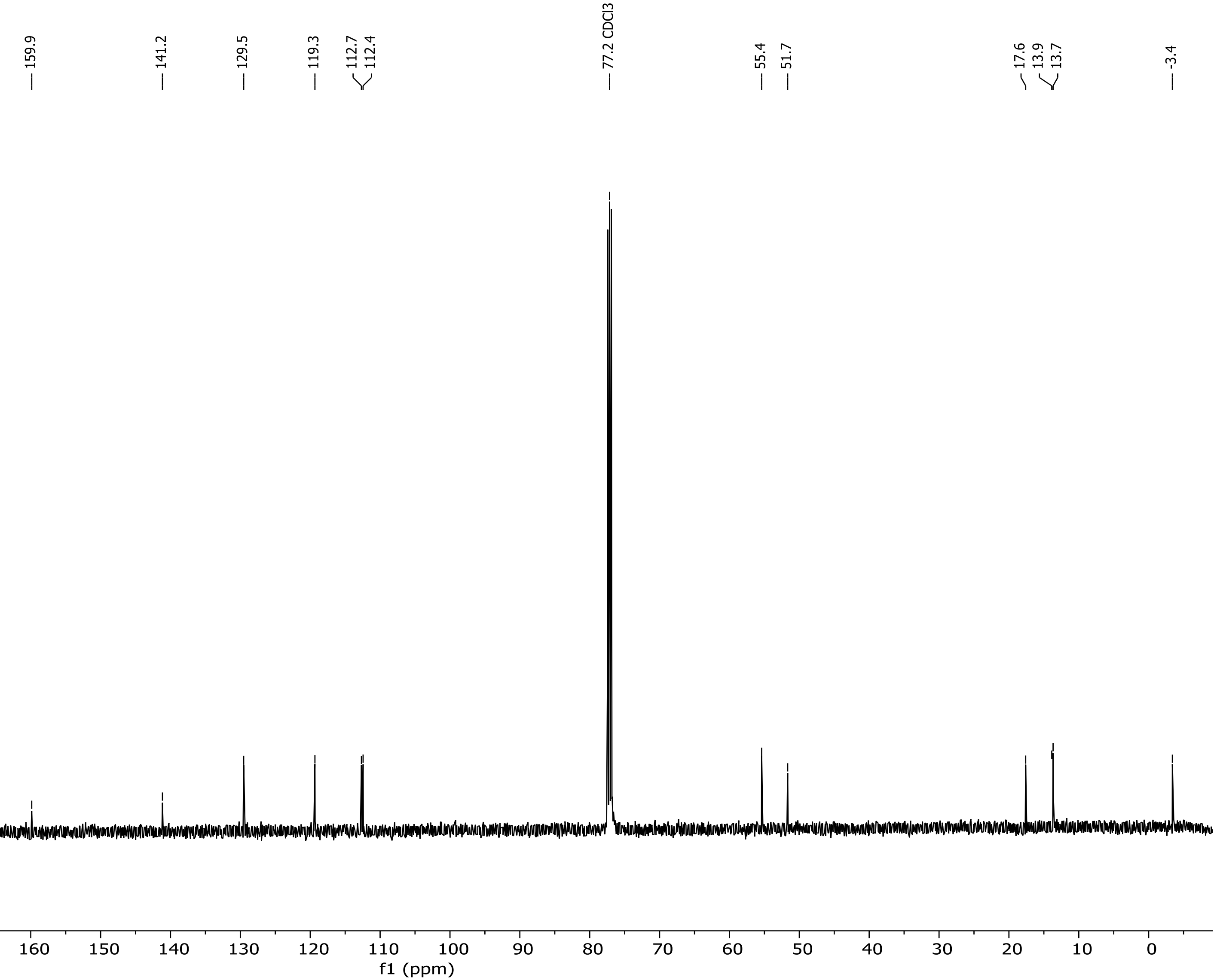

Figure S74. ^{29}Si NMR ($^1H/^{29}Si$ HMQC, 99 MHz, $CDCl_3$, optimized for J = 7 Hz) of *rac*-**1g.**

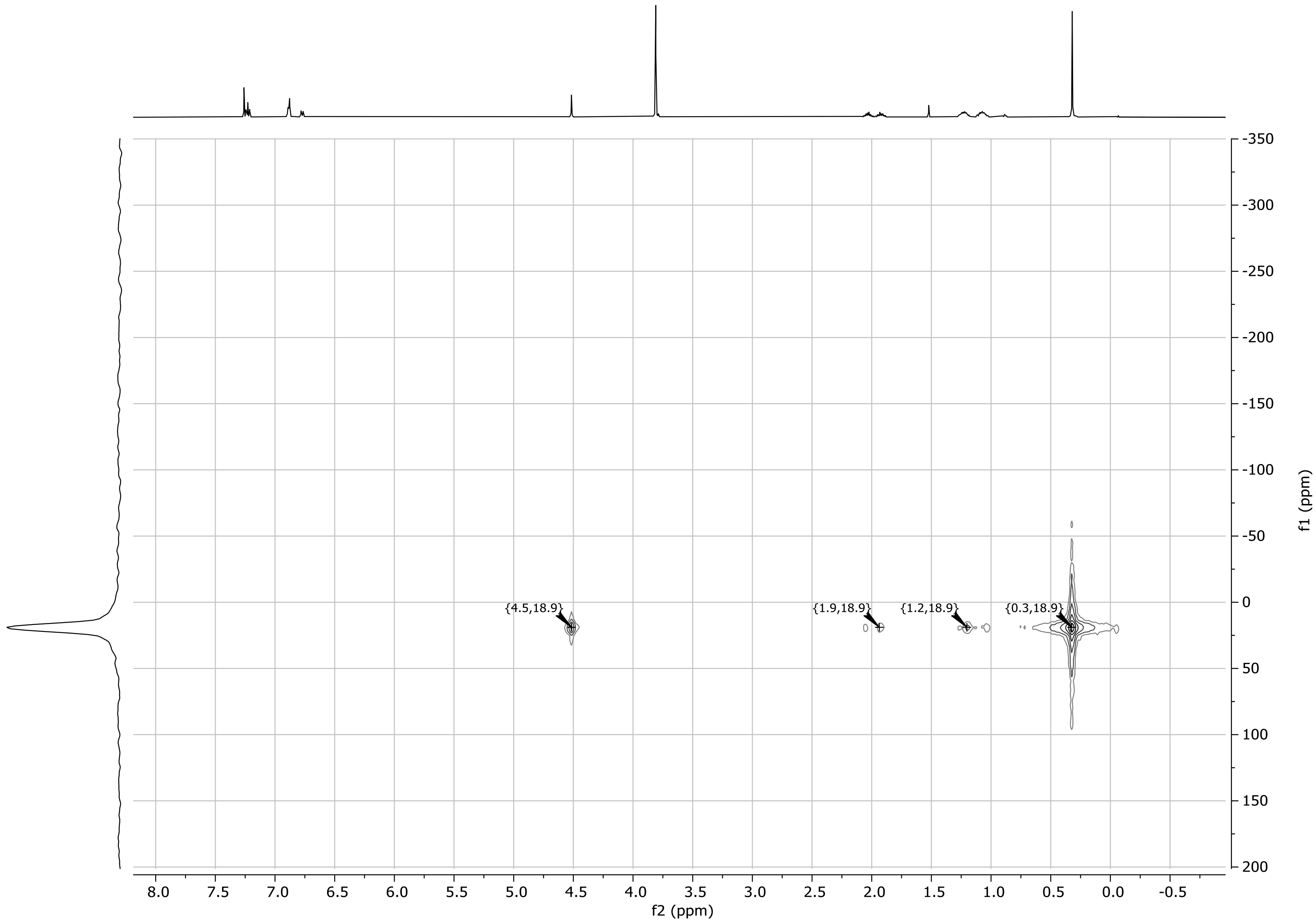

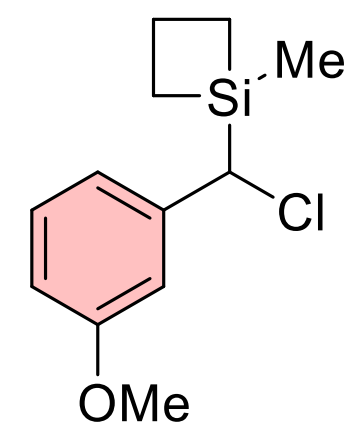

rac-**1g**
$C_{12}H_{17}ClOSi$
M_W = 240.80

Figure S75. ^{1}**H NMR** (500 MHz, $CDCl_3$, 298 K) of *rac*-**6a**.

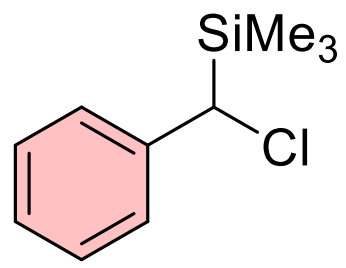

rac-**6a**
$C_{10}H_{15}ClSi$
M_W = 198.77

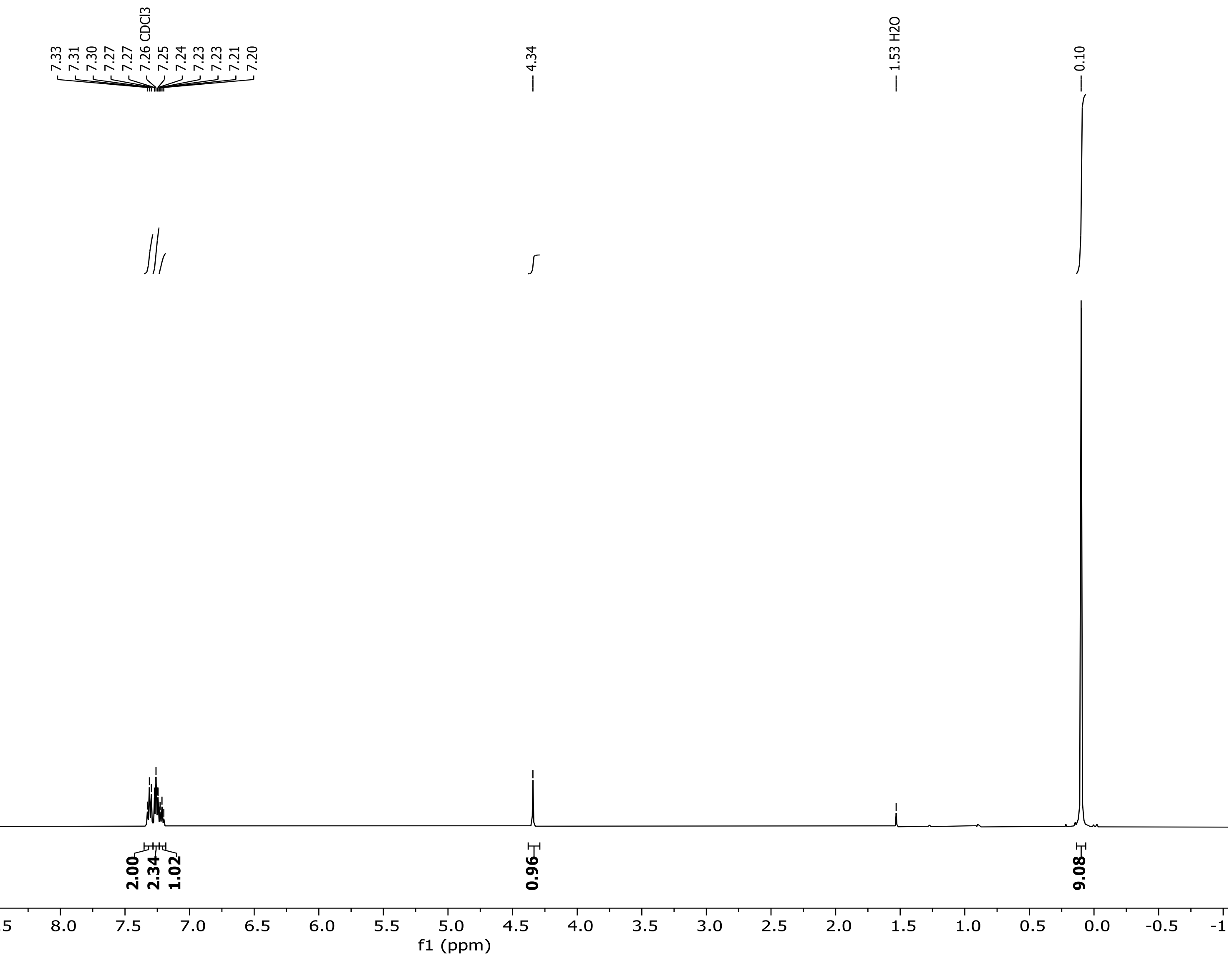

Figure S76. ^{13}**C NMR** (100 MHz, $CDCl_3$, 298 K) of *rac*-**6a**.

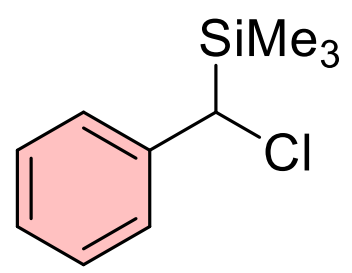

rac-**6a**
$C_{10}H_{15}ClSi$
M_W = 198.77

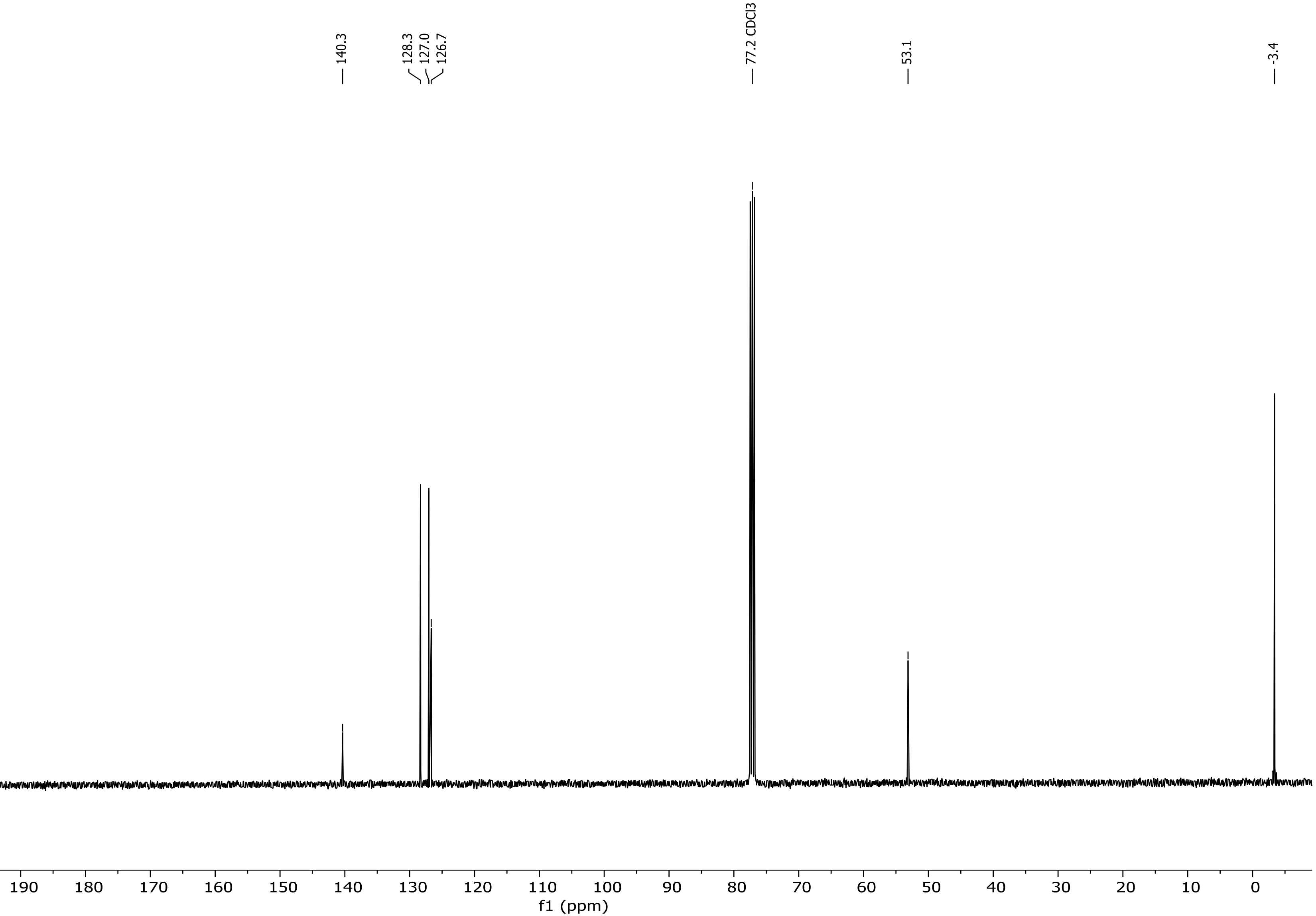

Figure S77. ^{29}**Si NMR** ($^{1}H/^{29}Si$ HMQC, 99 MHz, $CDCl_3$, optimized for J = 7 Hz) of *rac*-**6a.**

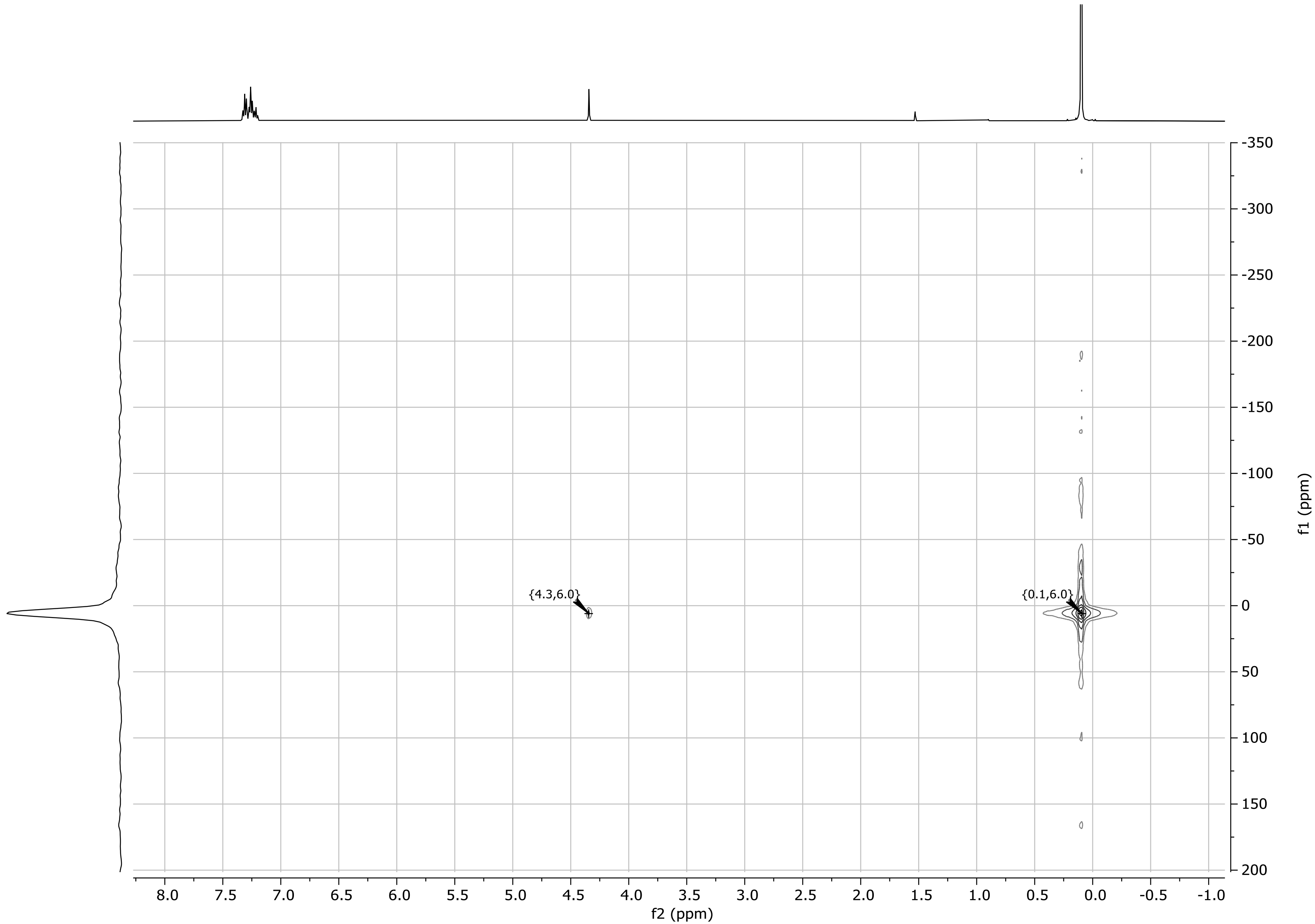

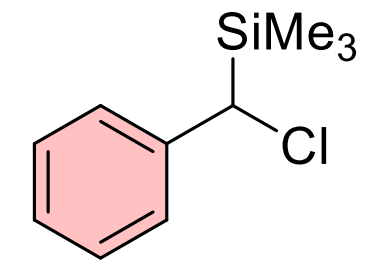

rac-**6a**

$C_{10}H_{15}ClSi$

M_W = 198.77

Figure S78. **^{1}H NMR** (500 MHz, $CDCl_3$, 298 K) of *rac*-**4a**.

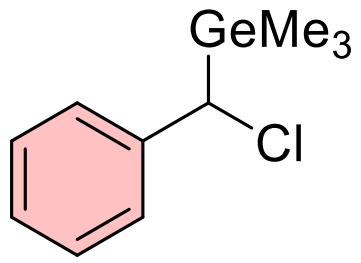

rac-**4a**
$C_{10}H_{15}ClGe$
M_W = 243.31

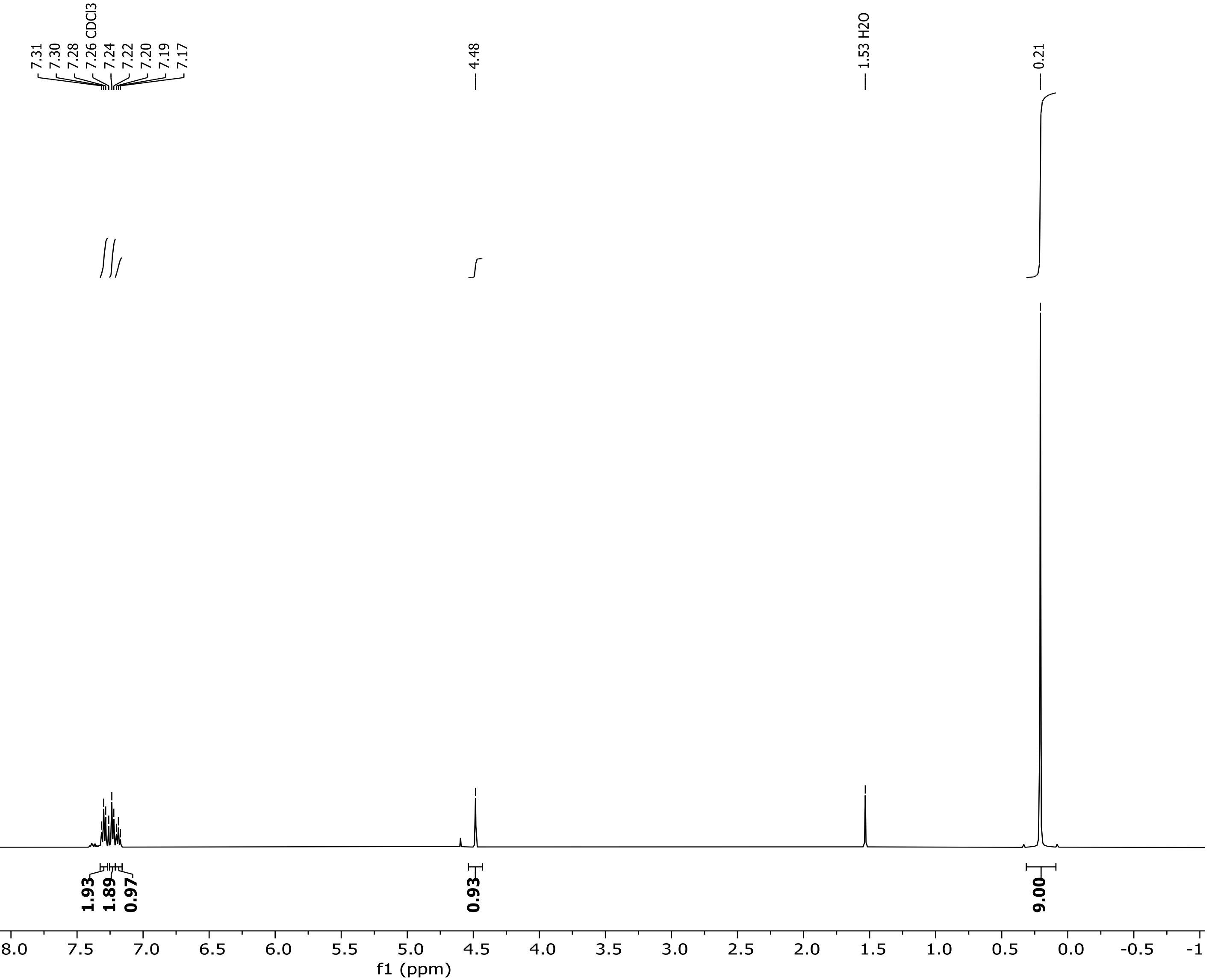

Figure S79. ^{13}C NMR (125 MHz, $CDCl_3$, 298 K) of *rac*-**4a**.

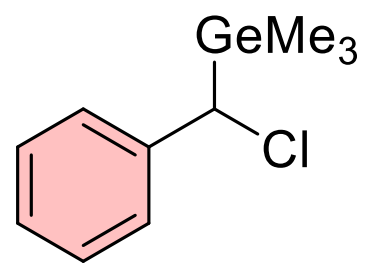

rac-**4a**
$C_{10}H_{15}ClGe$
M_W = 243.31

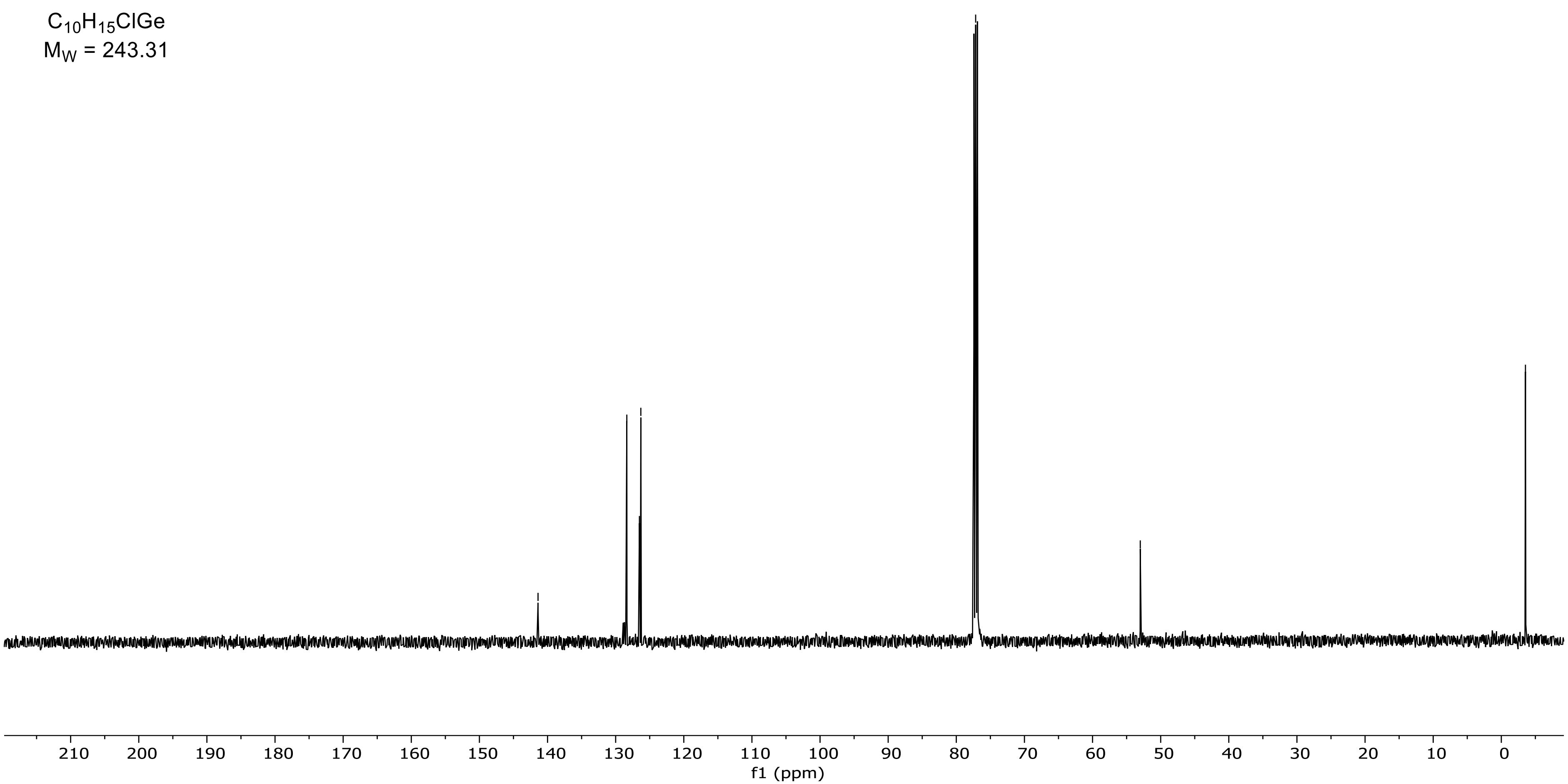

Figure S80. **¹H NMR** (500 MHz, $CDCl_3$, 298 K) of *rac*-**4b**.

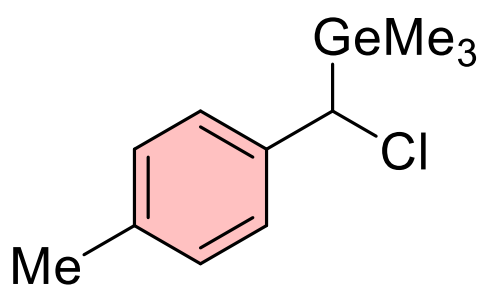

rac-**4b**
$C_{11}H_{17}ClGe$
M_W = 257.34

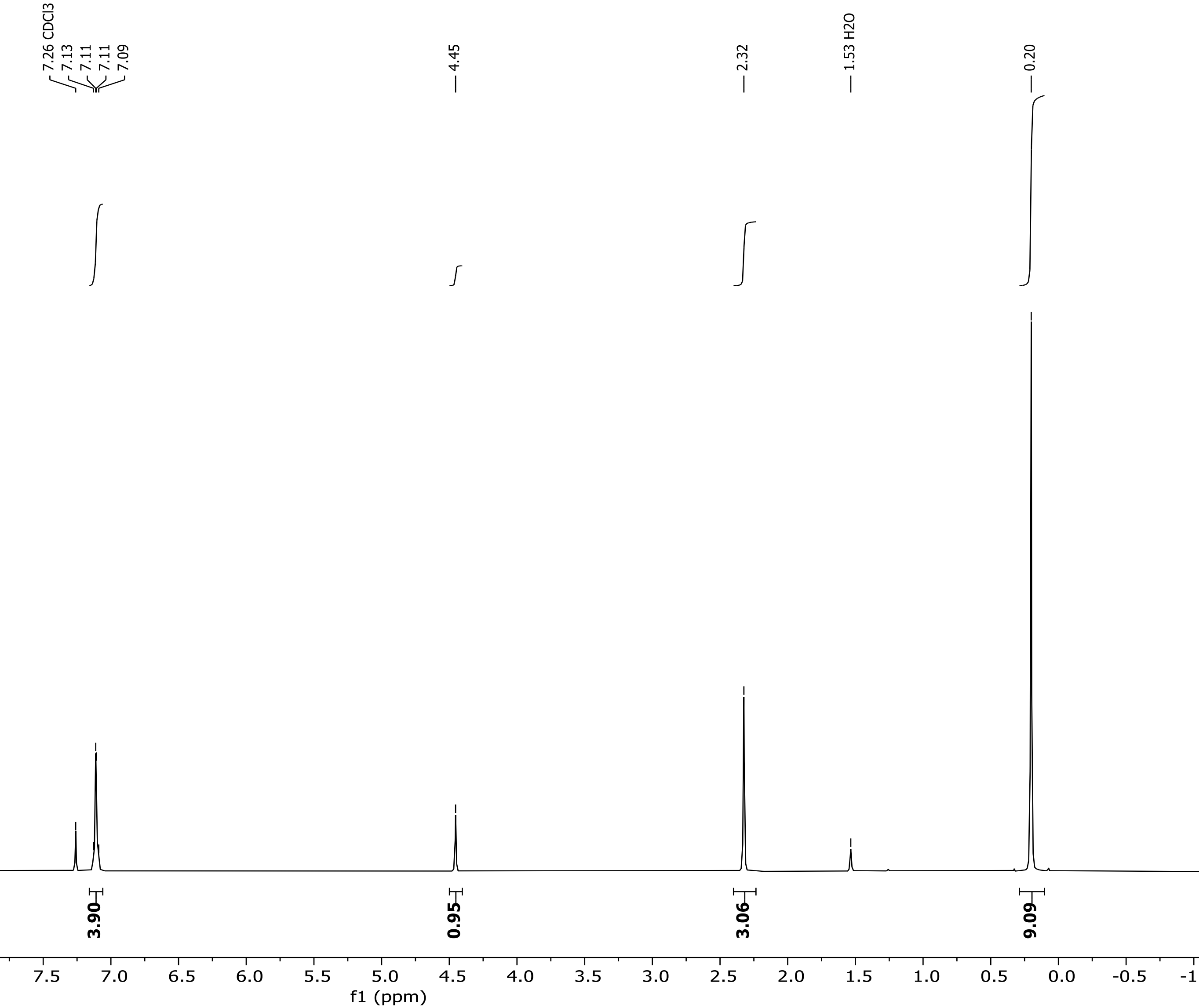

Figure S81. ^{13}**C NMR** (125 MHz, $CDCl_3$, 298 K) of *rac*-**4b**.

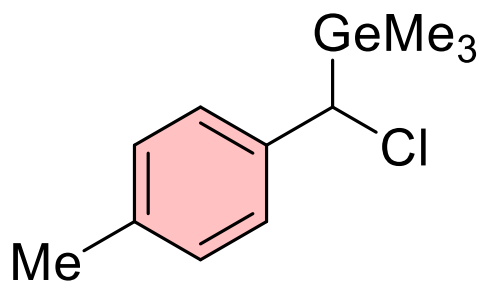

rac-**4b**
$C_{11}H_{17}ClGe$
M_W = 257.34

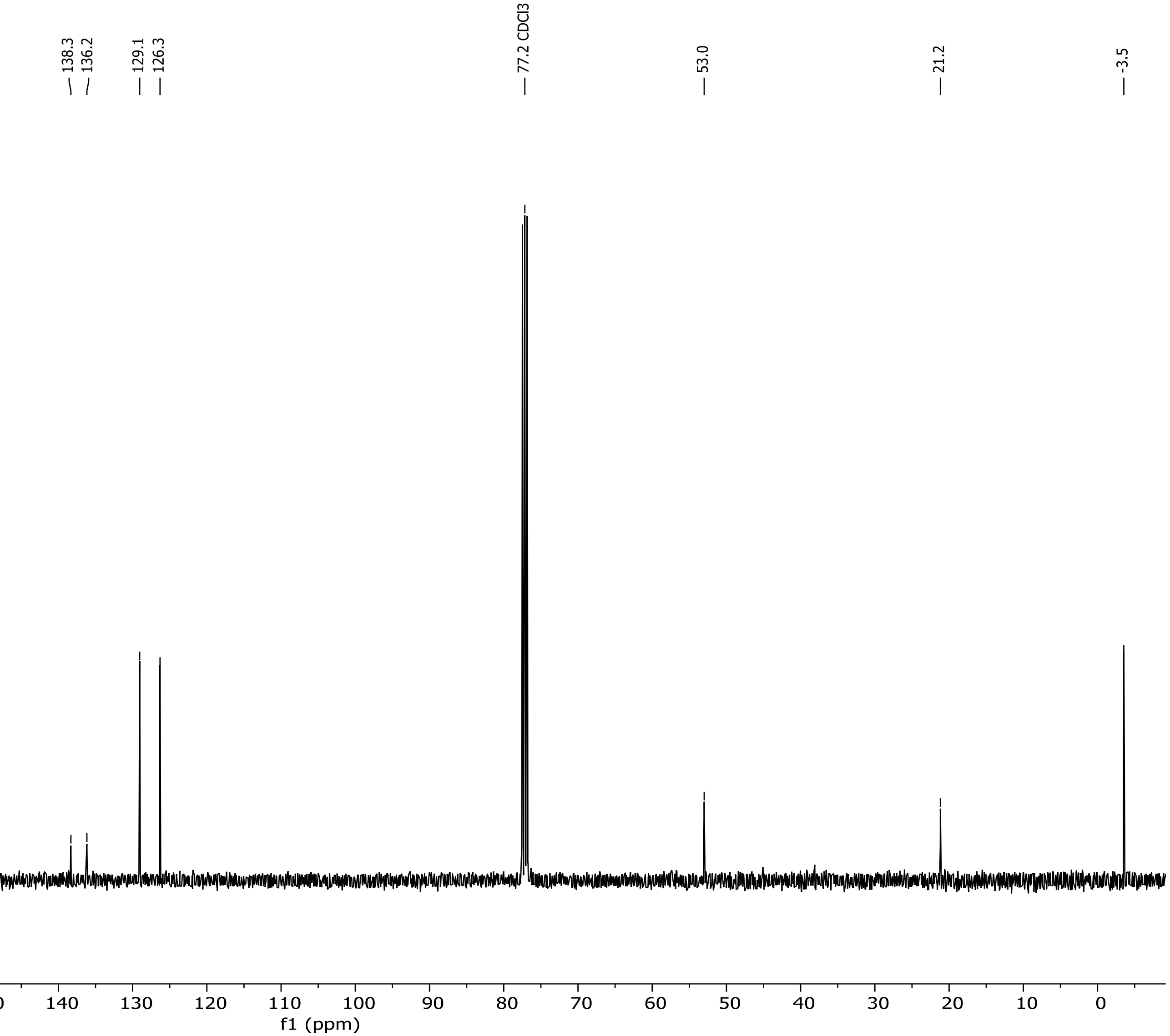

Figure S82. **^{1}H NMR** (500 MHz, $CDCl_3$, 298 K) of *rac*-**4c**.

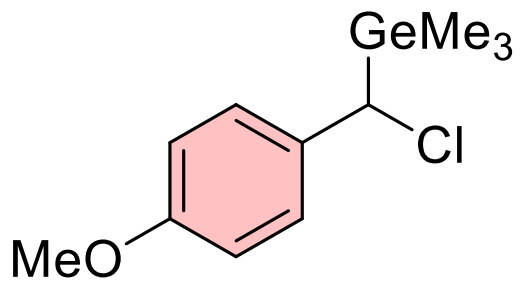

rac-**4c**
$C_{11}H_{17}ClGeO$
M_W = 273.34

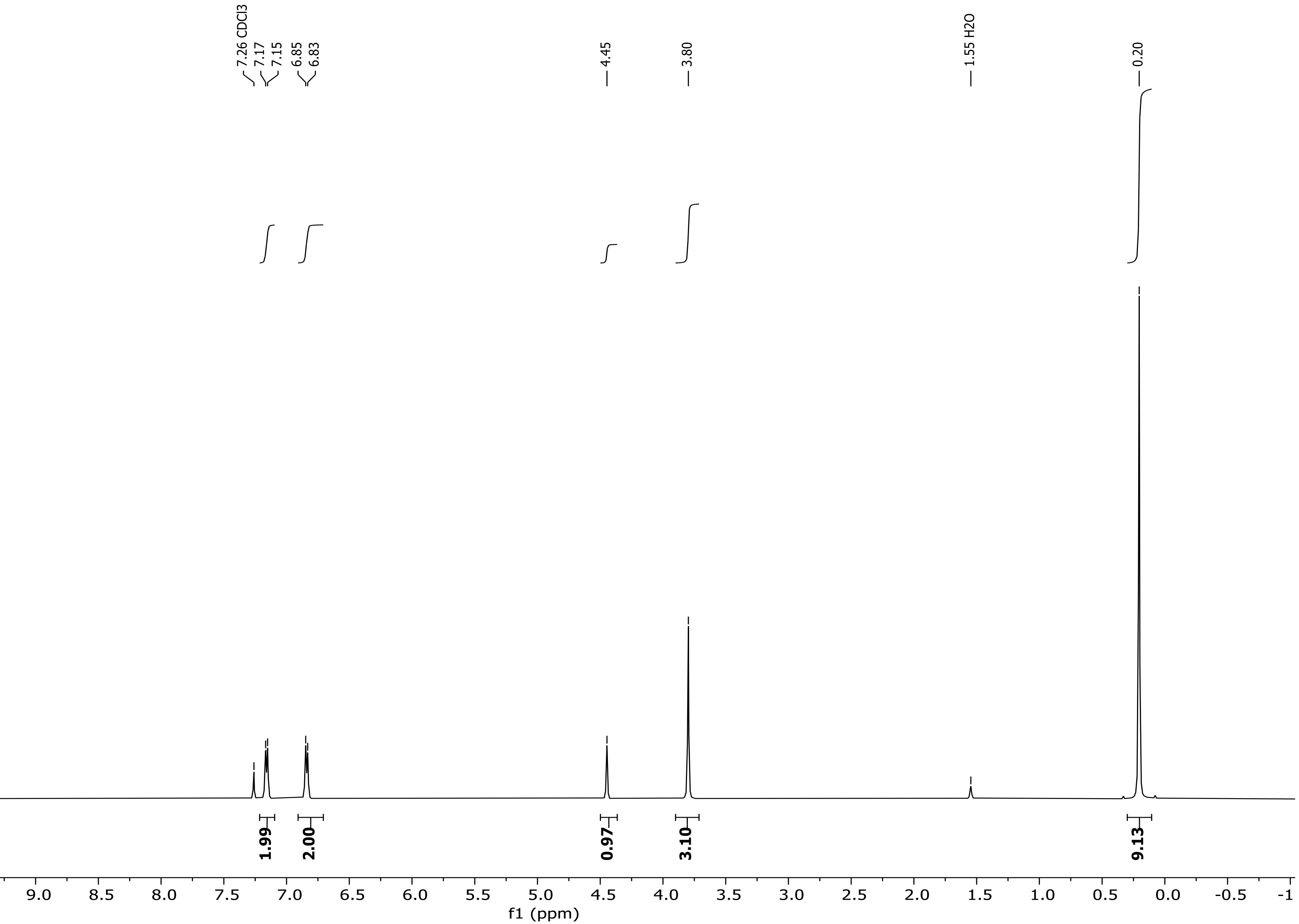

Figure S83. ^{13}**C NMR** (125 MHz, $CDCl_3$, 298 K) of *rac*-**4c**.

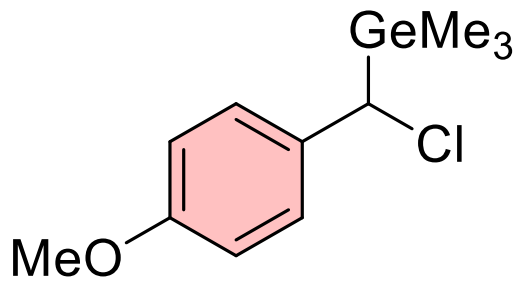

rac-**4c**
$C_{11}H_{17}ClGeO$
M_W = 273.34

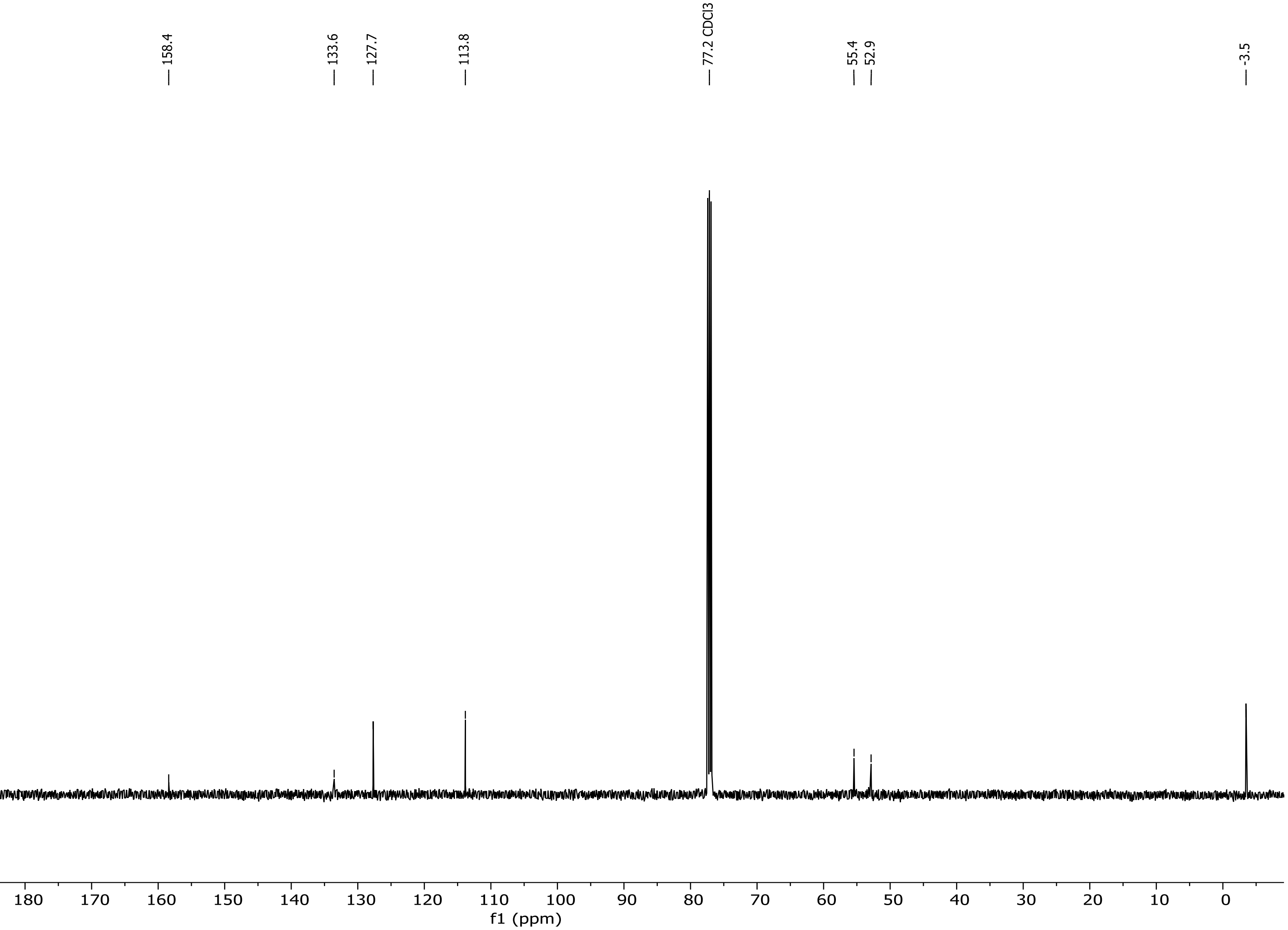

Figure S84. **¹H NMR** (400 MHz, $CDCl_3$, 298 K) of *rac*-**4d**.

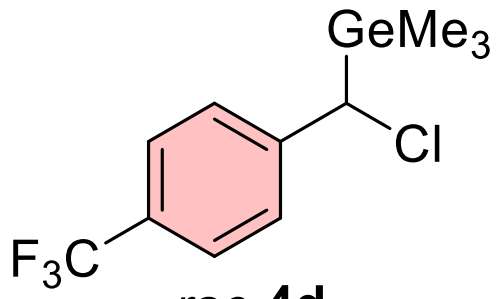

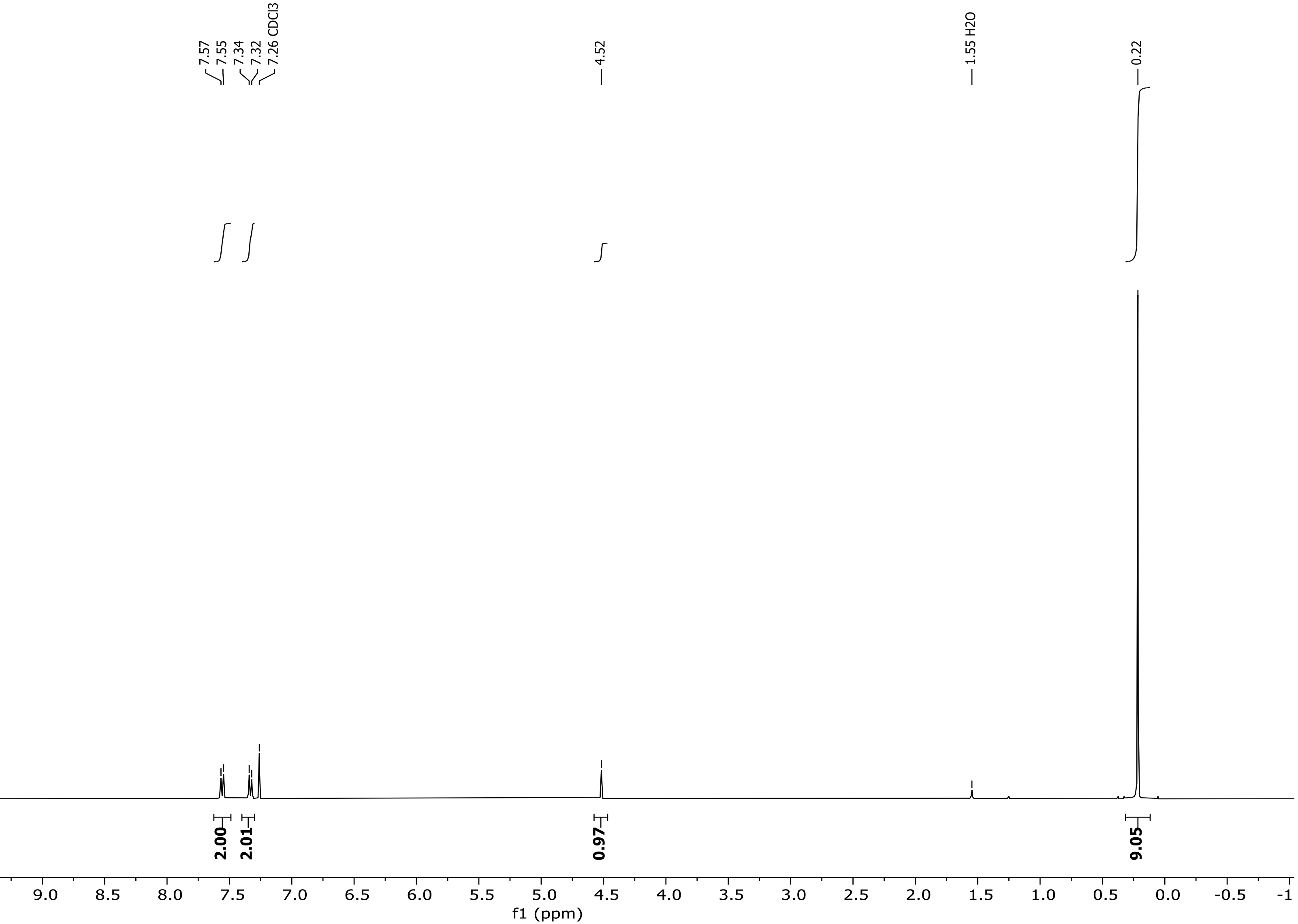

Figure S85. **^{13}C NMR** (125 MHz, $CDCl_3$, 298 K) of *rac*-**4d**.

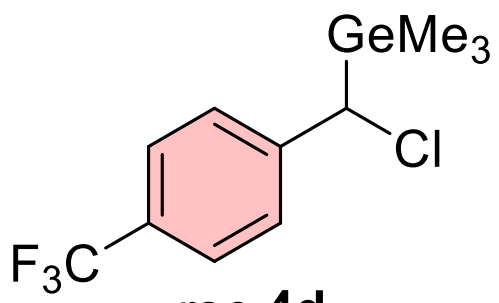

rac-**4d**
$C_{11}H_{14}ClF_3Ge$
M_W = 311.31

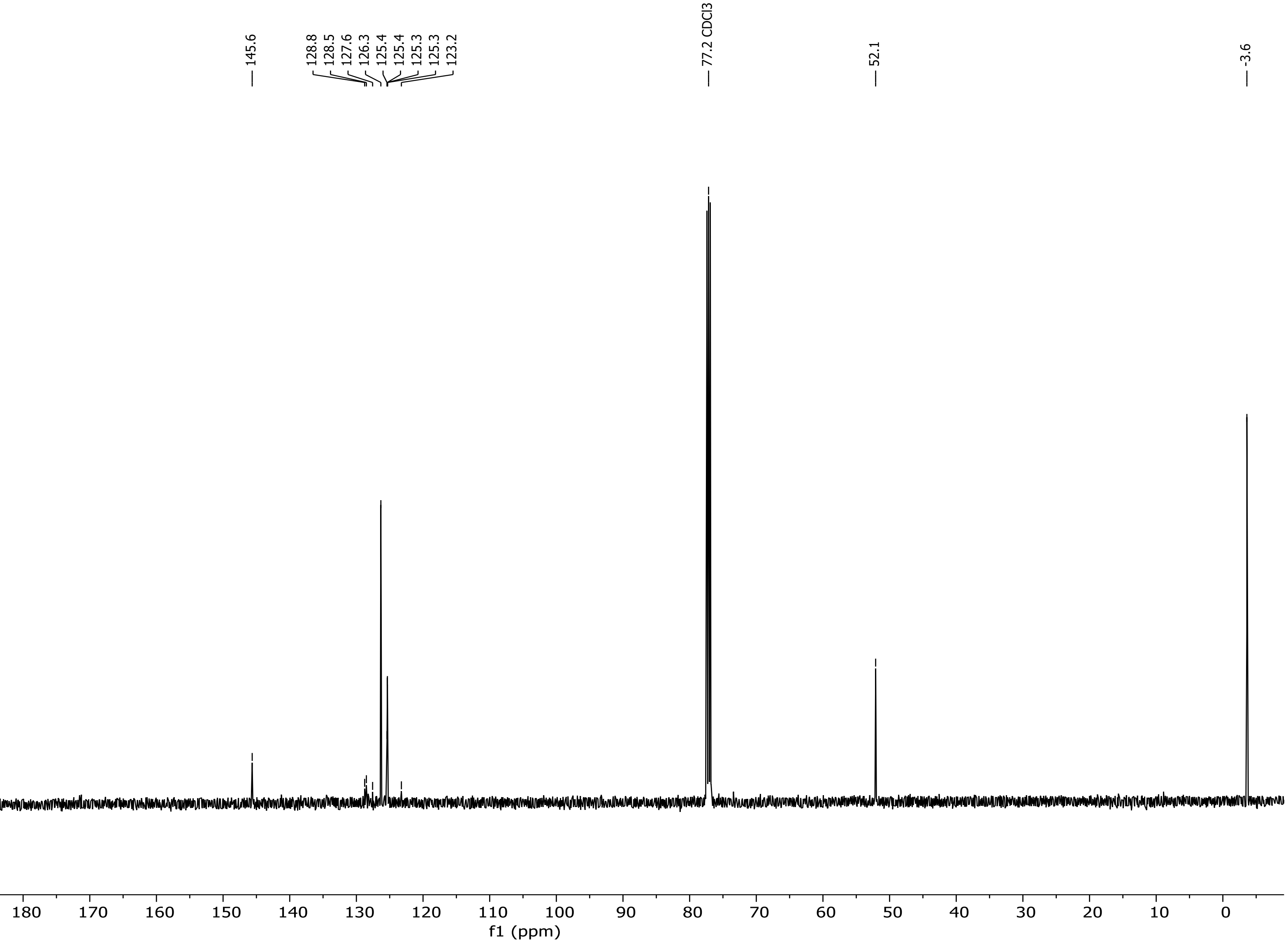

Figure S86. **^{19}F NMR** (470 MHz, $CDCl_3$, 298 K) of *rac*-**4d**.

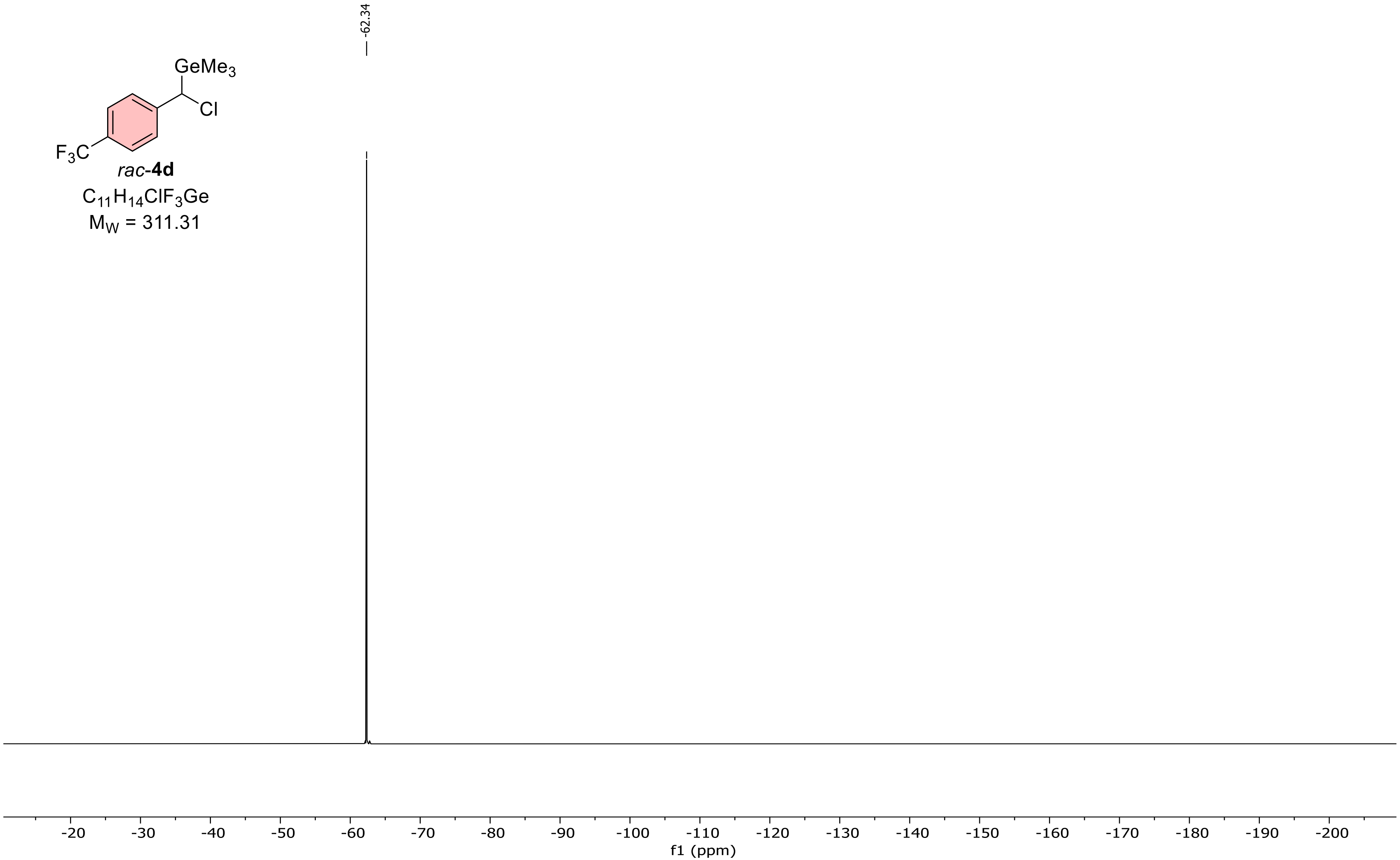

Figure S87. ¹H NMR (500 MHz, $CDCl_3$, 298 K) of *rac*-**8a**.

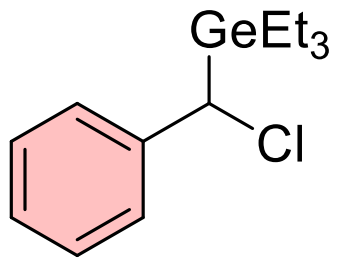

rac-**8a**
$C_{13}H_{21}ClGe$
M_W = 285.39

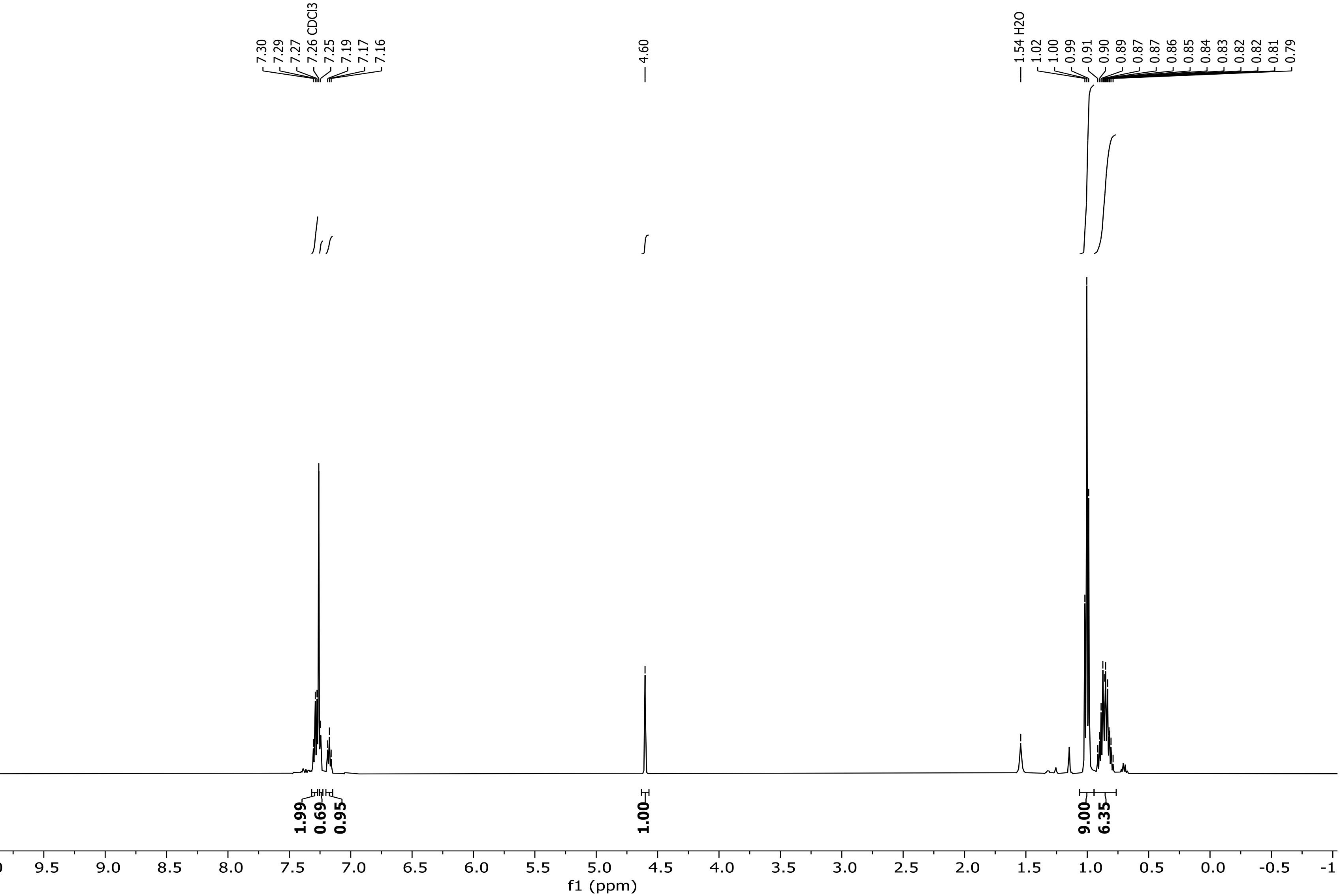

Figure S88. ^{13}C NMR (125 MHz, $CDCl_3$, 298 K) of *rac*-**8a**.

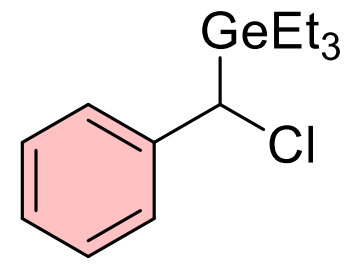

rac-**8a**
$C_{13}H_{21}ClGe$
M_W = 285.39

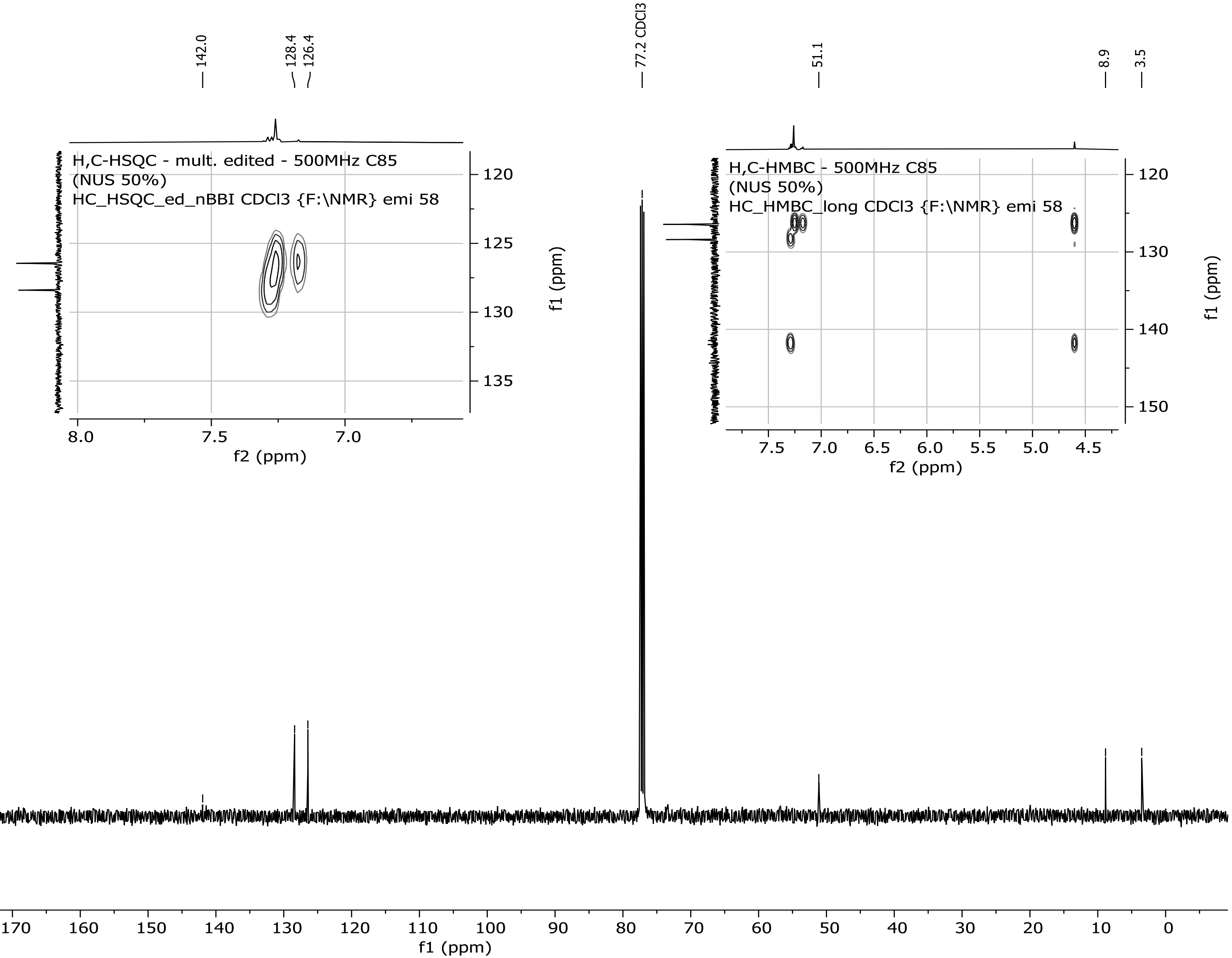

Figure S89. **^{1}H NMR** (500 MHz, $CDCl_3$, 298 K) of *rac*-**S4e**.

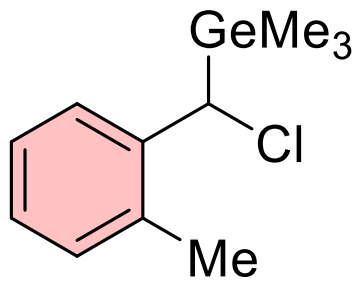

rac-**S4e**
$C_{11}H_{17}ClGe$
M_W = 257.34

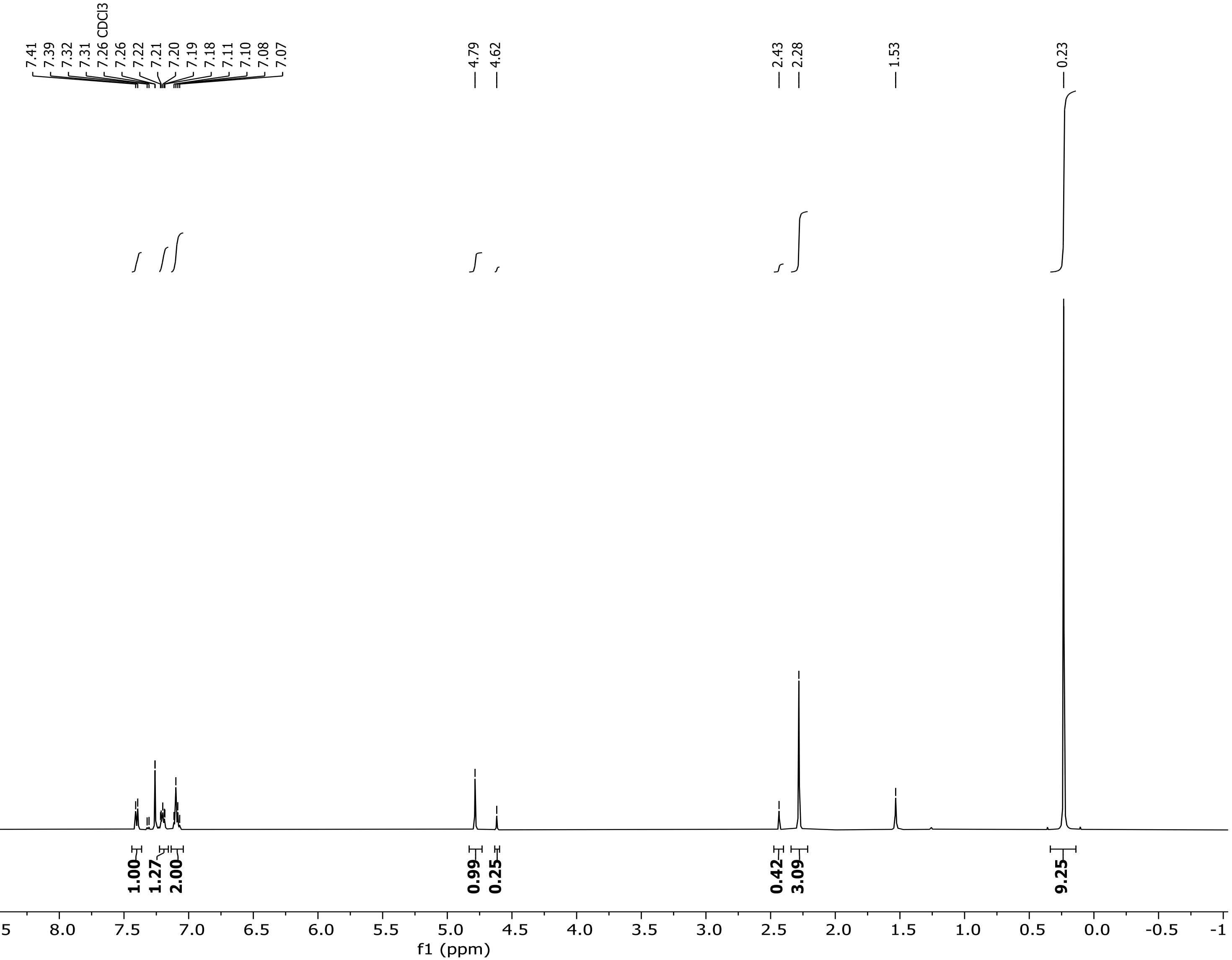

Figure S90. **^{13}C NMR** (100 MHz, $CDCl_3$, 298 K) of *rac*-**S4e**.

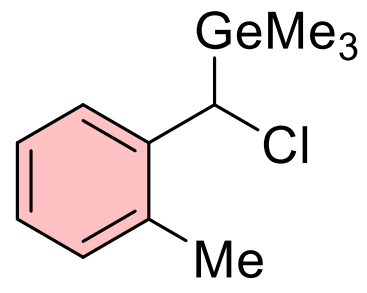

rac-**S4e**
$C_{11}H_{17}ClGe$
M_W = 257.34

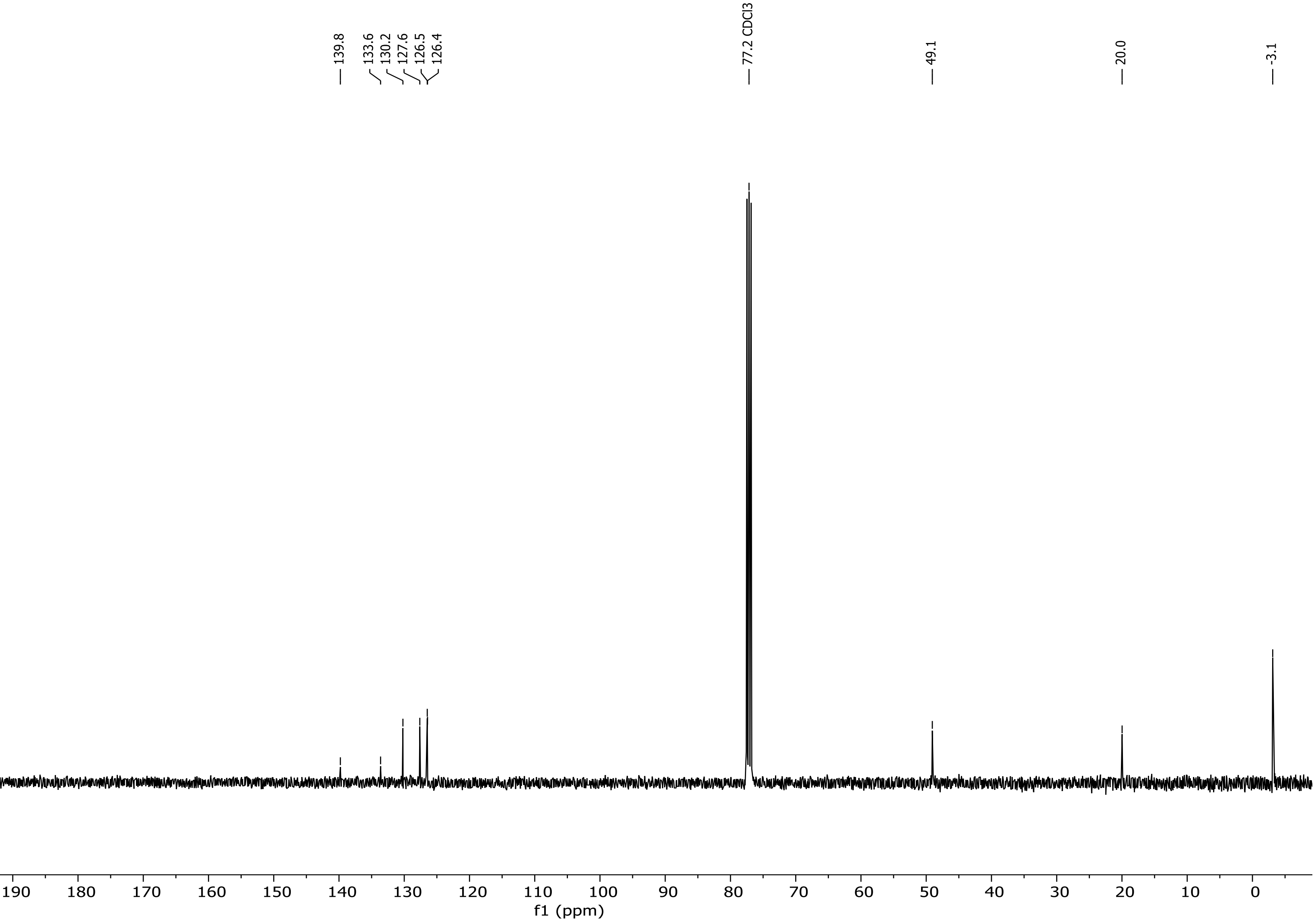

Figure S91. ^{1}**H NMR** (500 MHz, $CDCl_3$, 298 K) of *rac*-**S4f**.

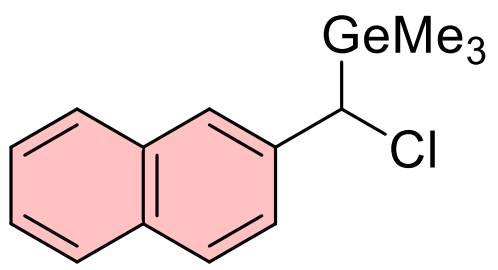

rac-**S4f**
$C_{14}H_{17}ClGe$
M_W = 293.37

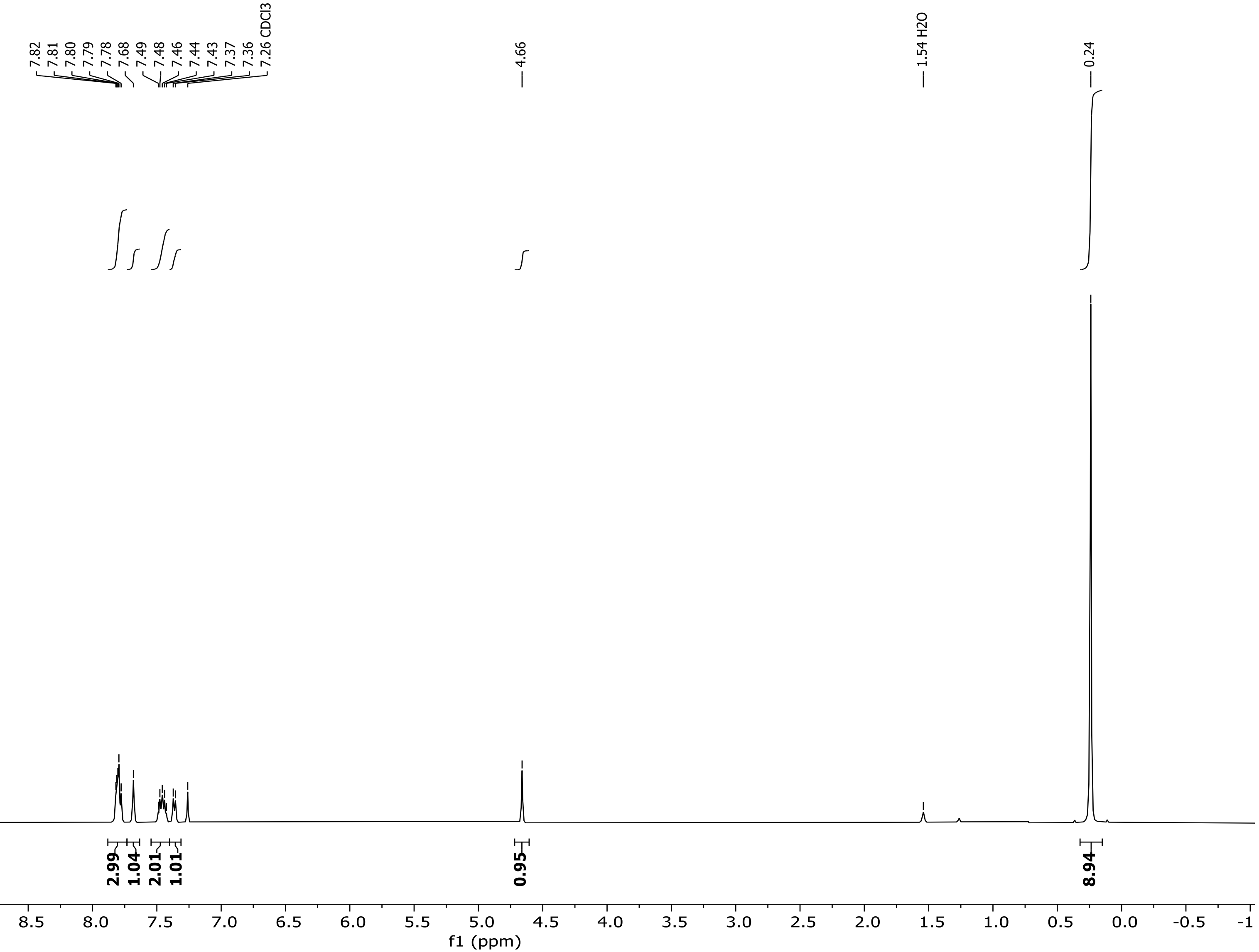

Figure S92. ^{13}**C NMR** (100 MHz, $CDCl_3$, 298 K) of *rac*-**S4f**.

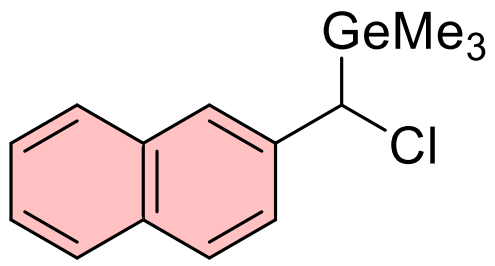

rac-**S4f**
$C_{14}H_{17}ClGe$
M_W = 293.37

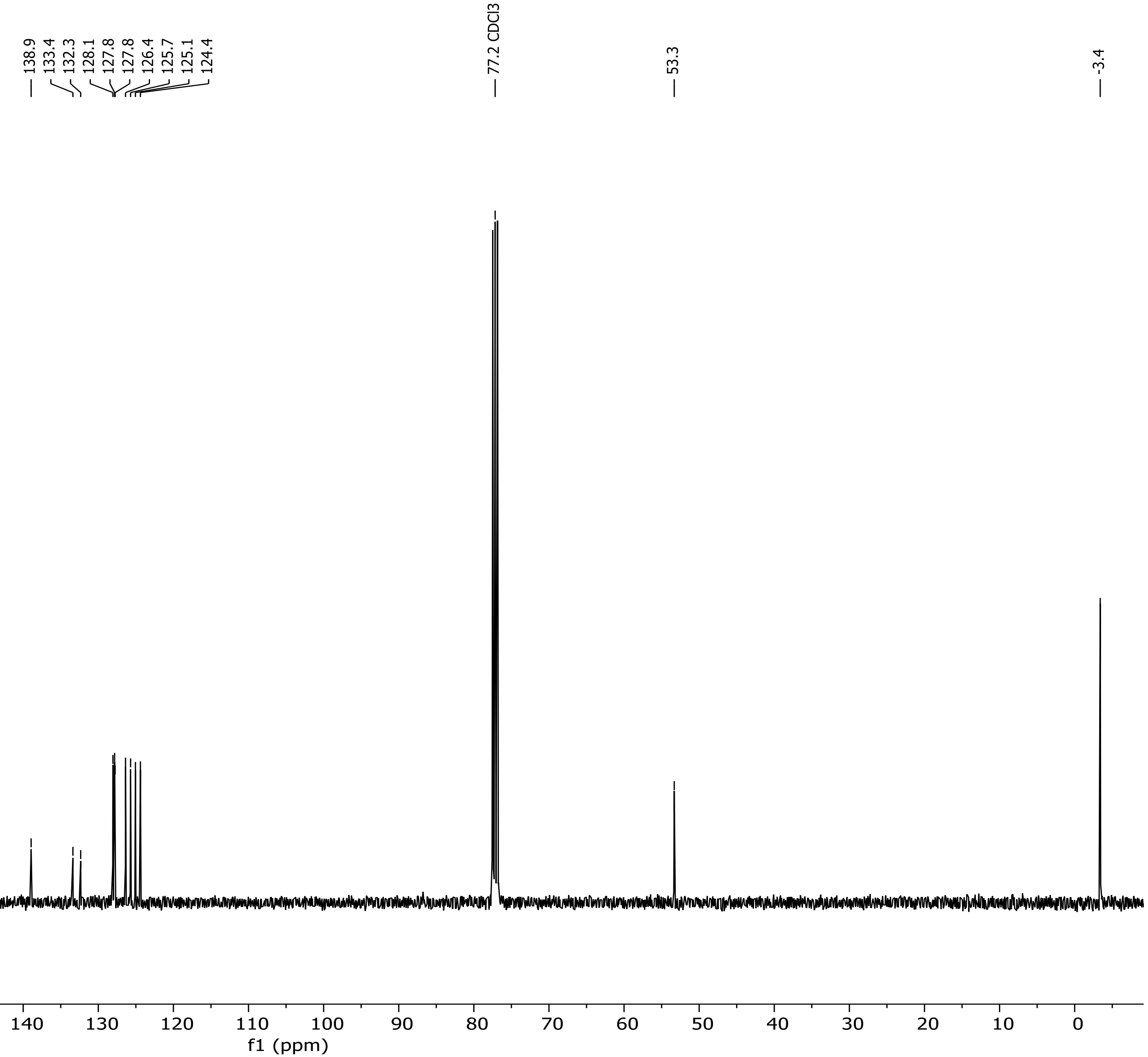

Figure S93. **^{1}H NMR** (400 MHz, $CDCl_3$, 298 K) of *rac*-**S4g**.

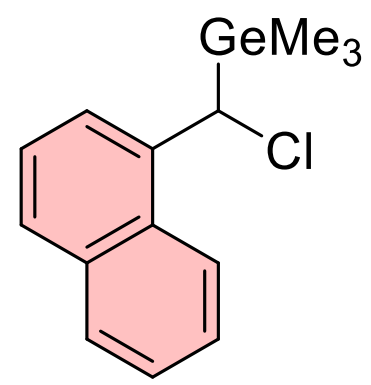

rac-**S4g**
$C_{14}H_{17}ClGe$
M_W = 293.37

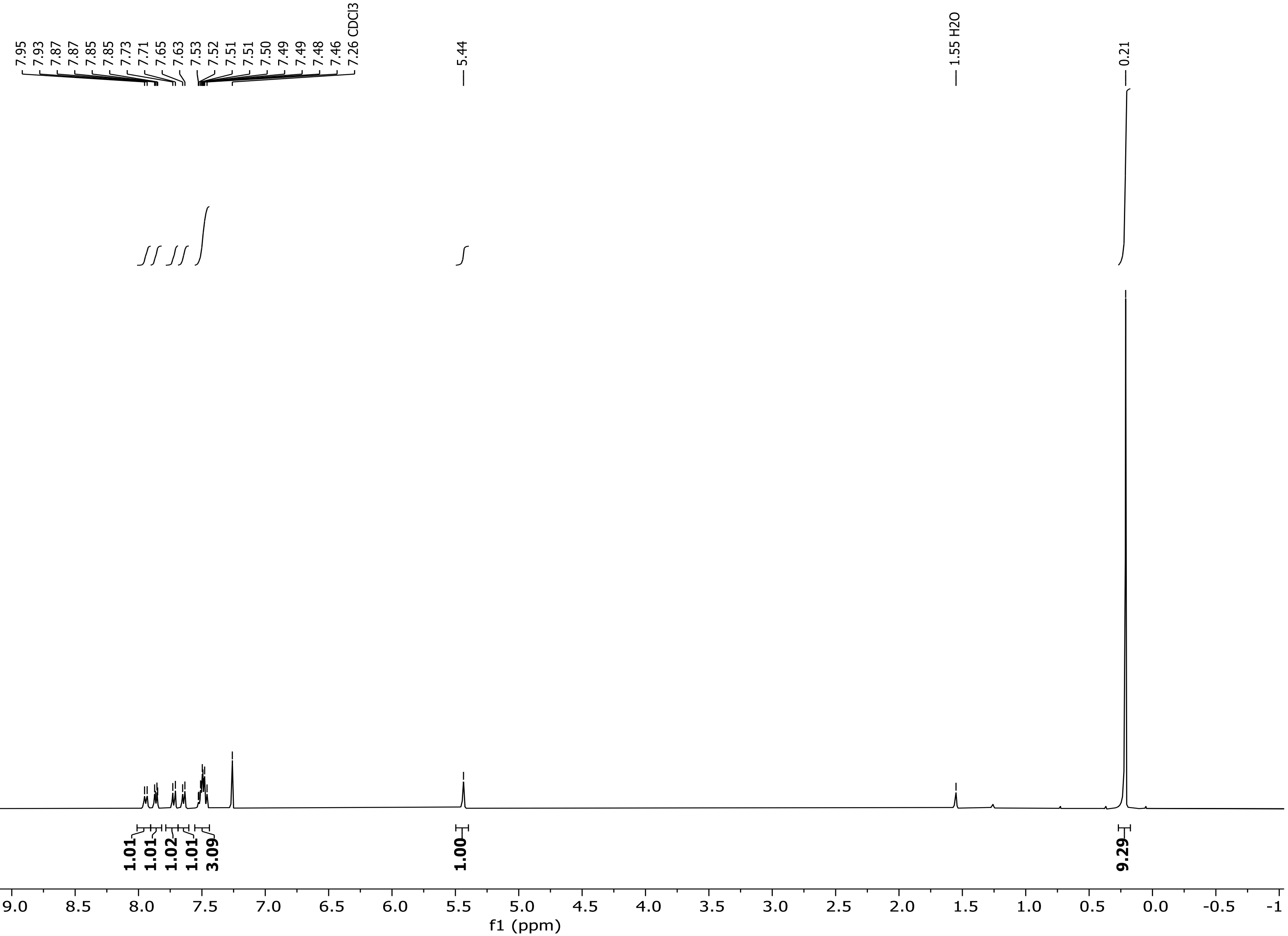

Figure S94. **^{13}C NMR** (100 MHz, $CDCl_3$, 298 K) of *rac*-**S4g**.

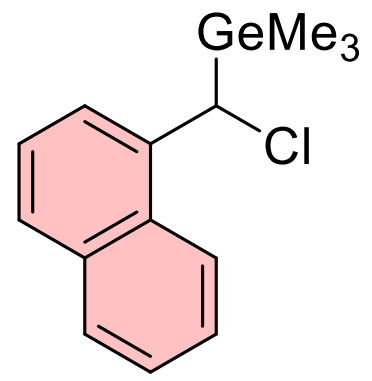

rac-**S4g**
$C_{14}H_{17}ClGe$
M_W = 293.37

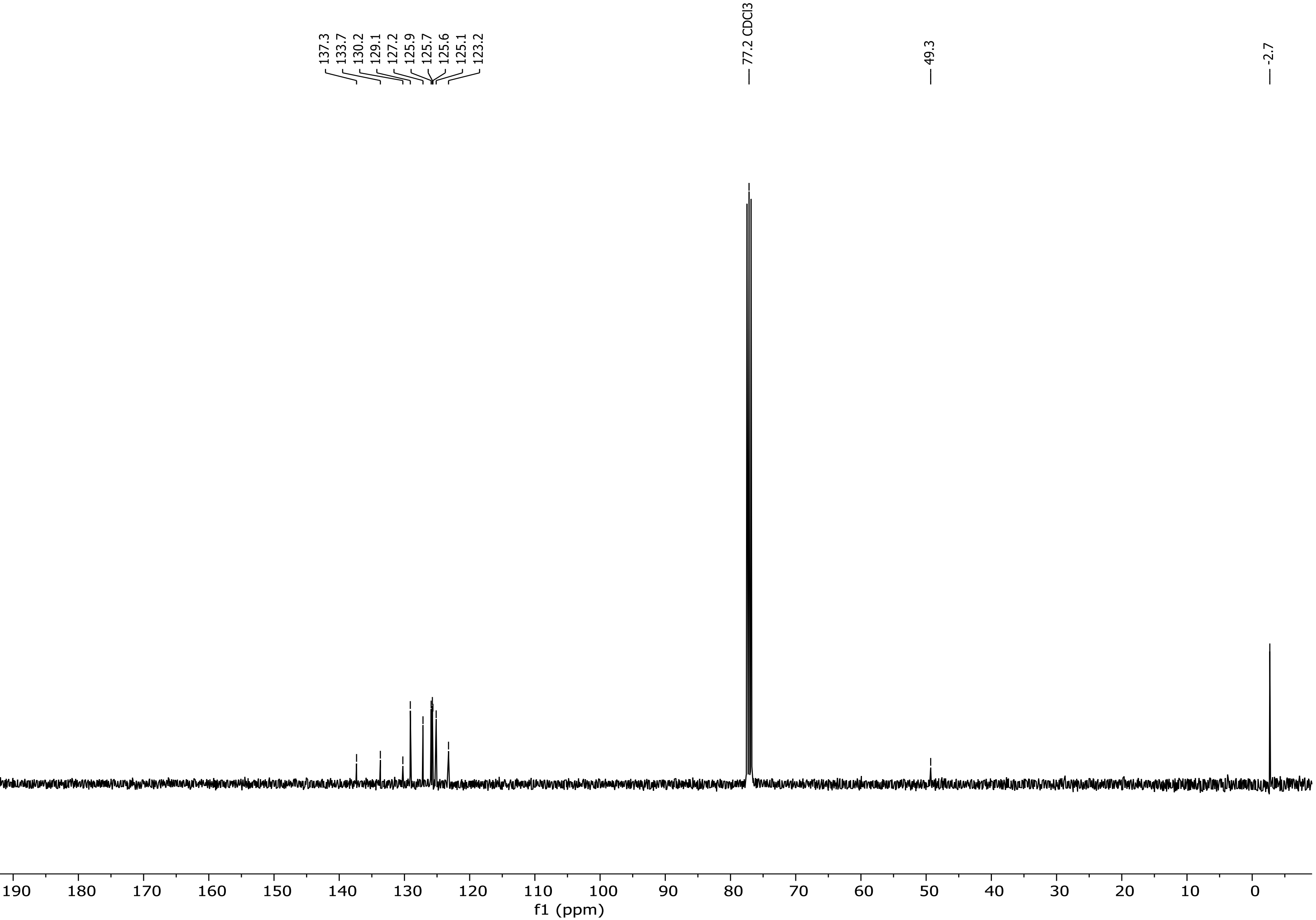

Figure S95. **¹H NMR** (500 MHz, $CDCl_3$, 298 K) of *rac*-**S4h**.

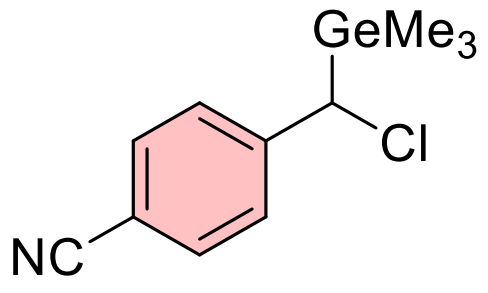

rac-**S4h**
$C_{11}H_{14}ClGeN$
M_W = 268.32

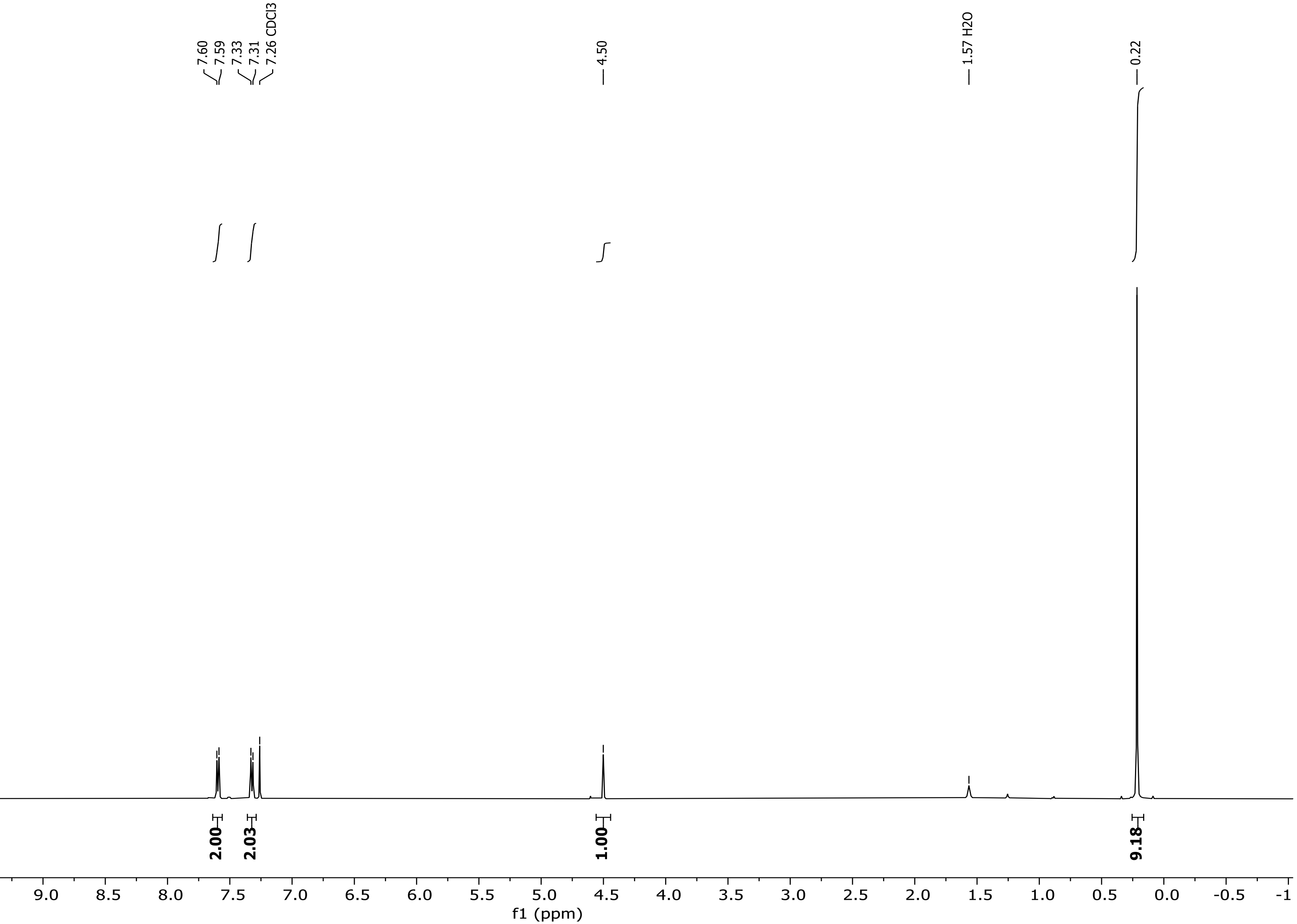

Figure S96. **^{13}C NMR** (100 MHz, $CDCl_3$, 298 K) of *rac*-**S4h**.

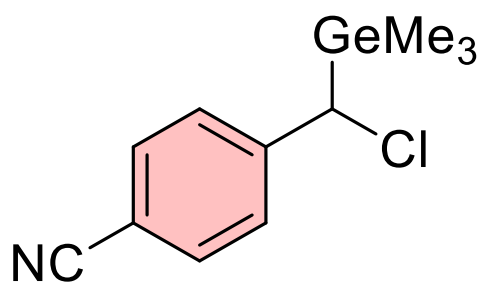

rac-**S4h**
$C_{11}H_{14}ClGeN$
M_W = 268.32

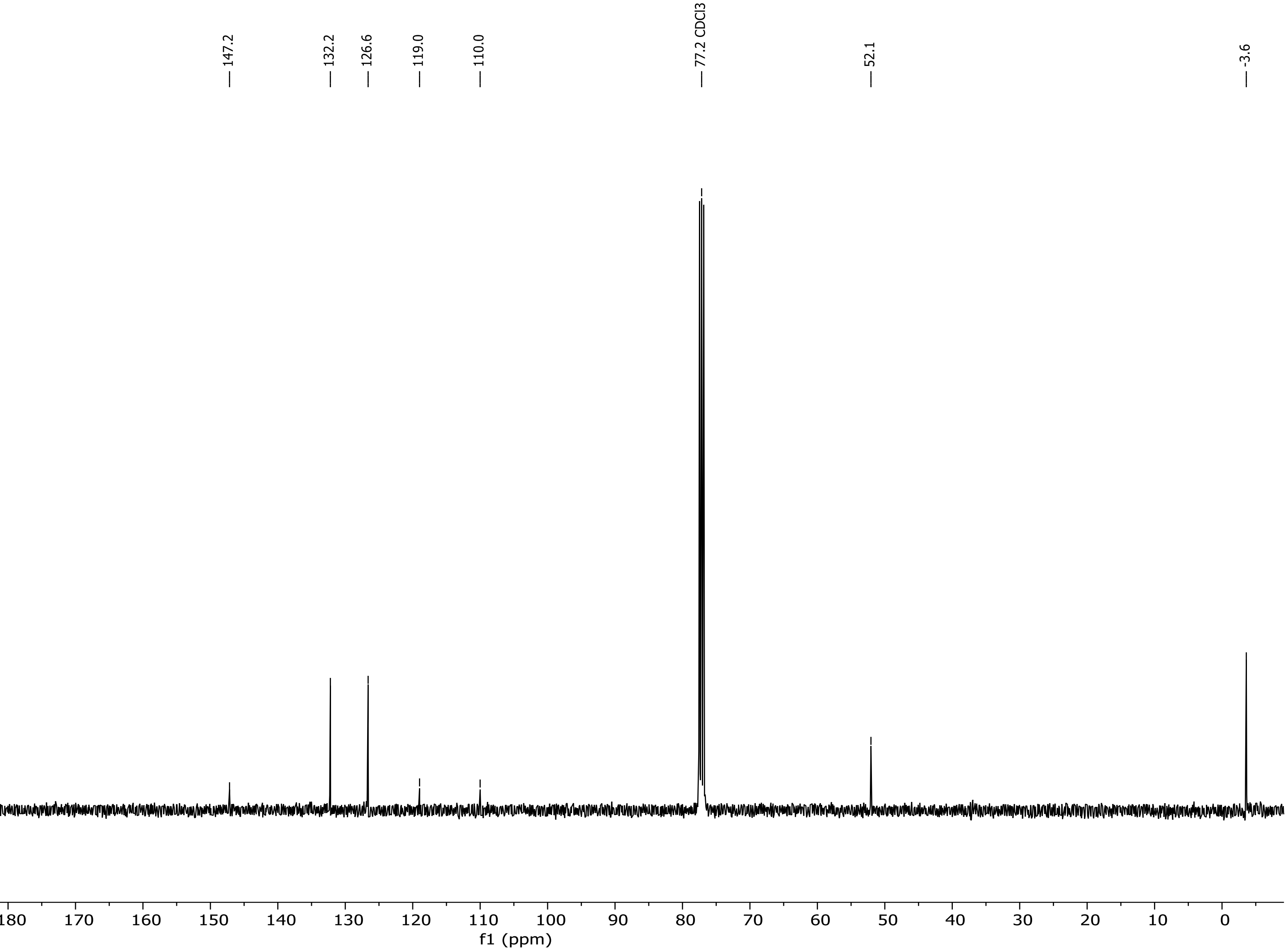

Figure S97. ^{1}**H NMR** (500 MHz, $CDCl_3$, 298 K) of *rac*-**S4i**.

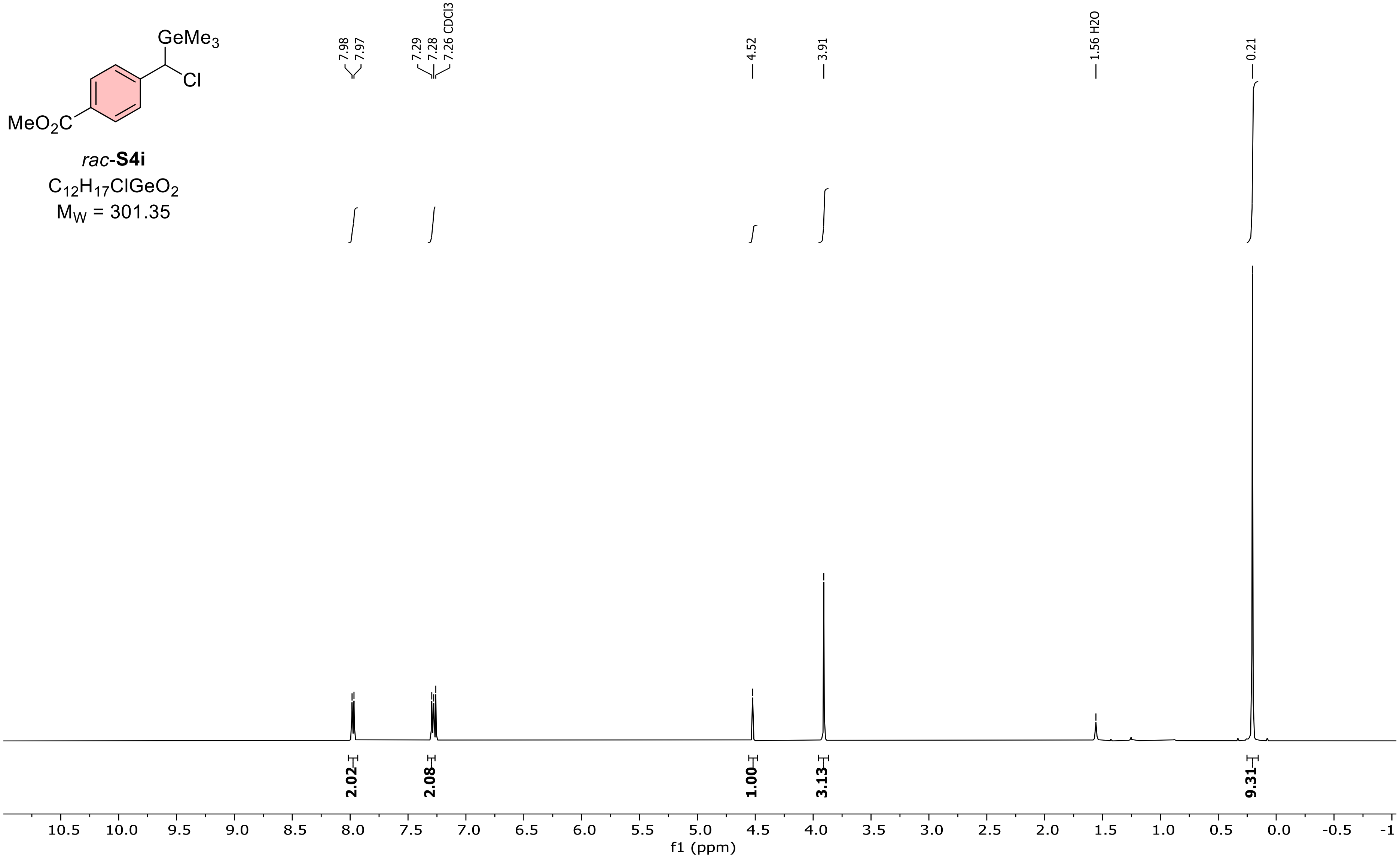

Figure S98. ^{13}**C NMR** (125 MHz, $CDCl_3$, 298 K) of *rac*-**S4i**.

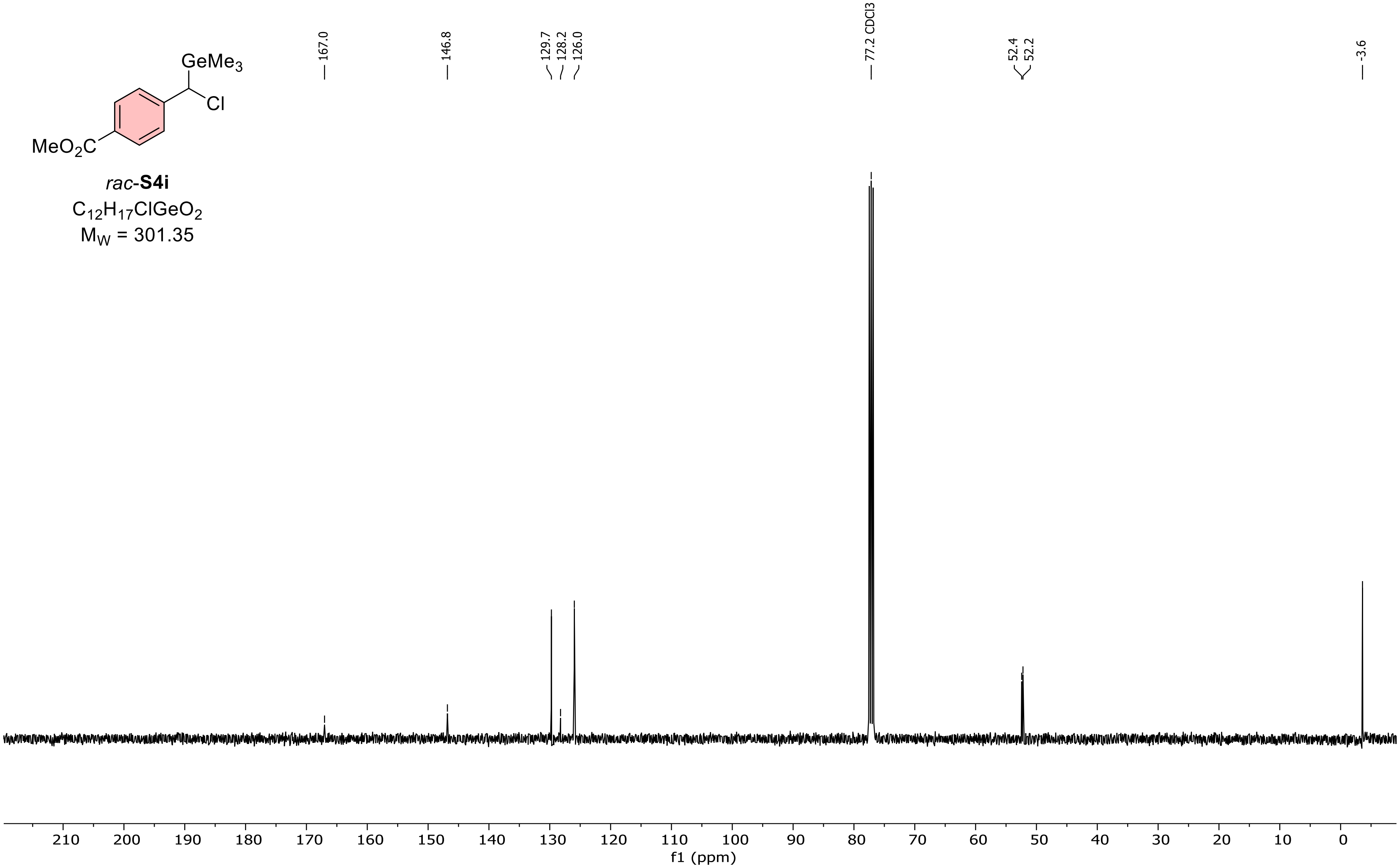

Figure S99. ^{1}**H NMR** (500 MHz, $CDCl_3$, 298 K) of **3aa**.

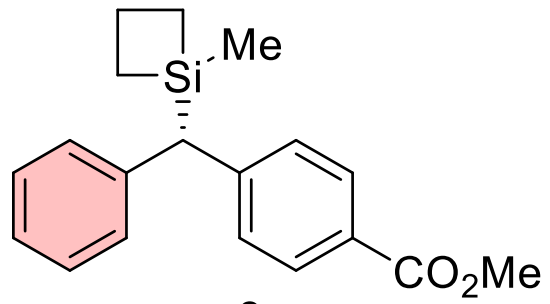

3aa
$C_{19}H_{22}O_2Si$
M_W = 310.47

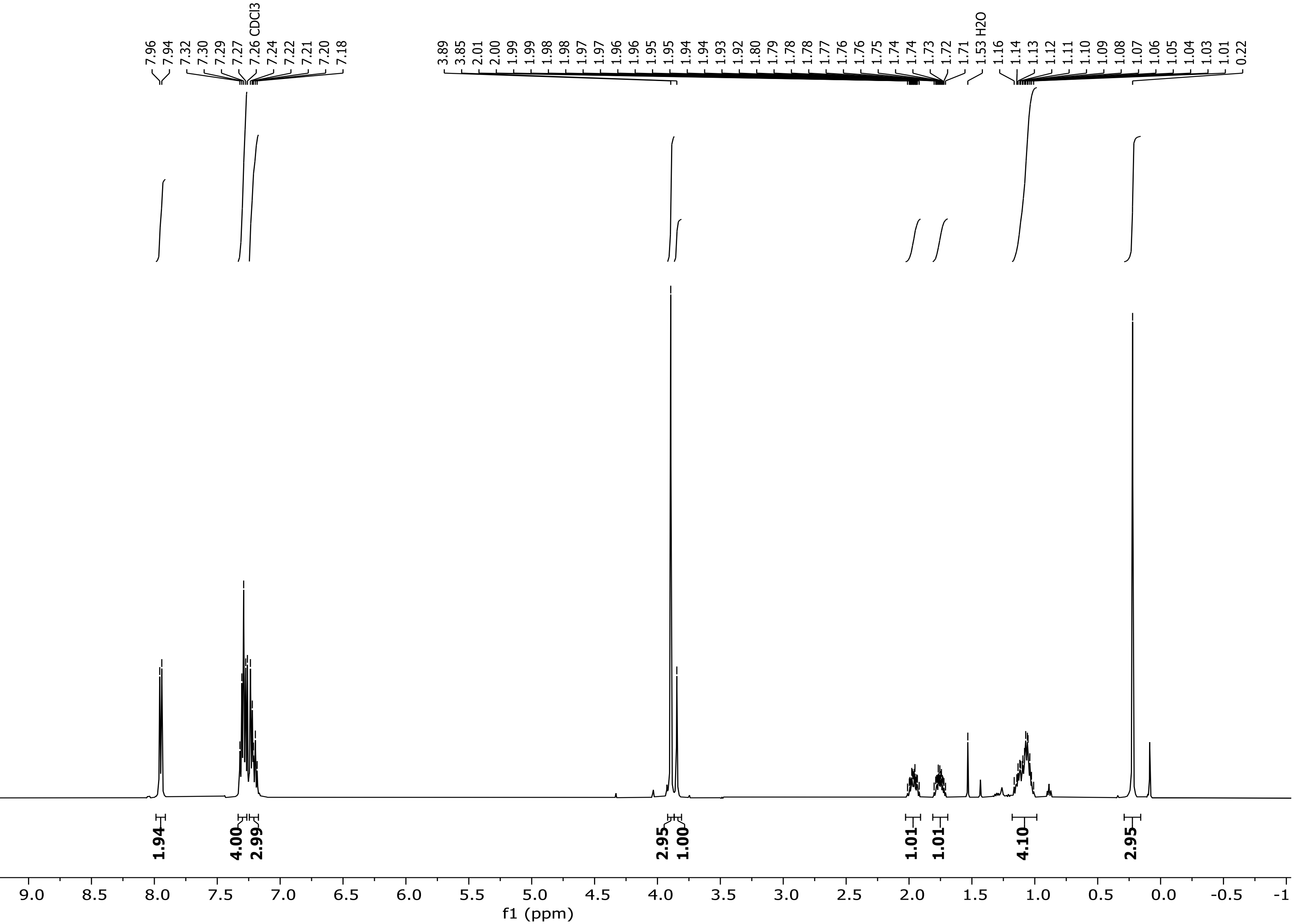

Figure S100. **^{13}C NMR** (100 MHz, $CDCl_3$, 298 K) of **3aa**.

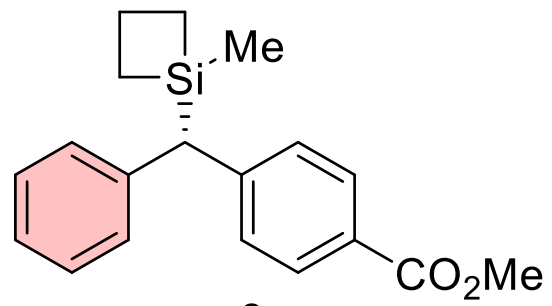

3aa
$C_{19}H_{22}O_2Si$
M_W = 310.47

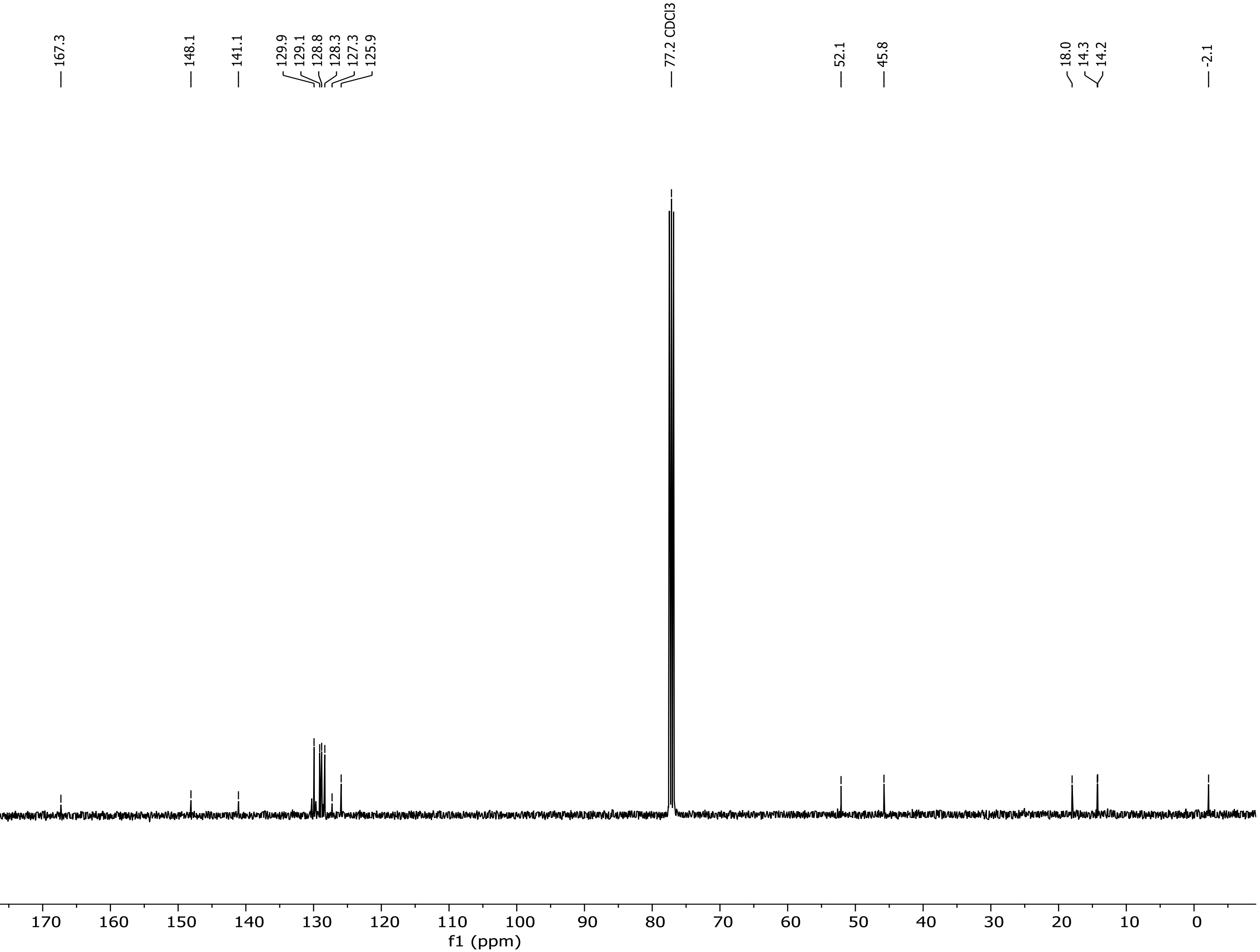

Figure S101. ^{29}Si NMR (^{1}H/^{29}Si HMQC, 99 MHz, $CDCl_3$, optimized for J = 7 Hz) of **3aa.**

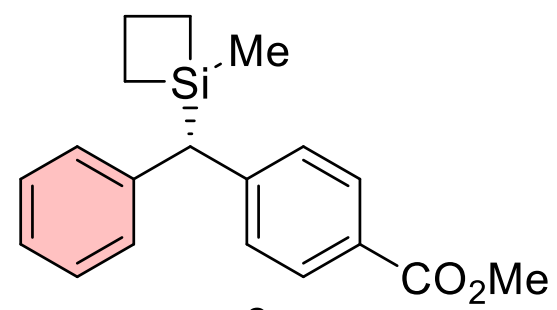

3aa
$C_{19}H_{22}O_2Si$
M_W = 310.47

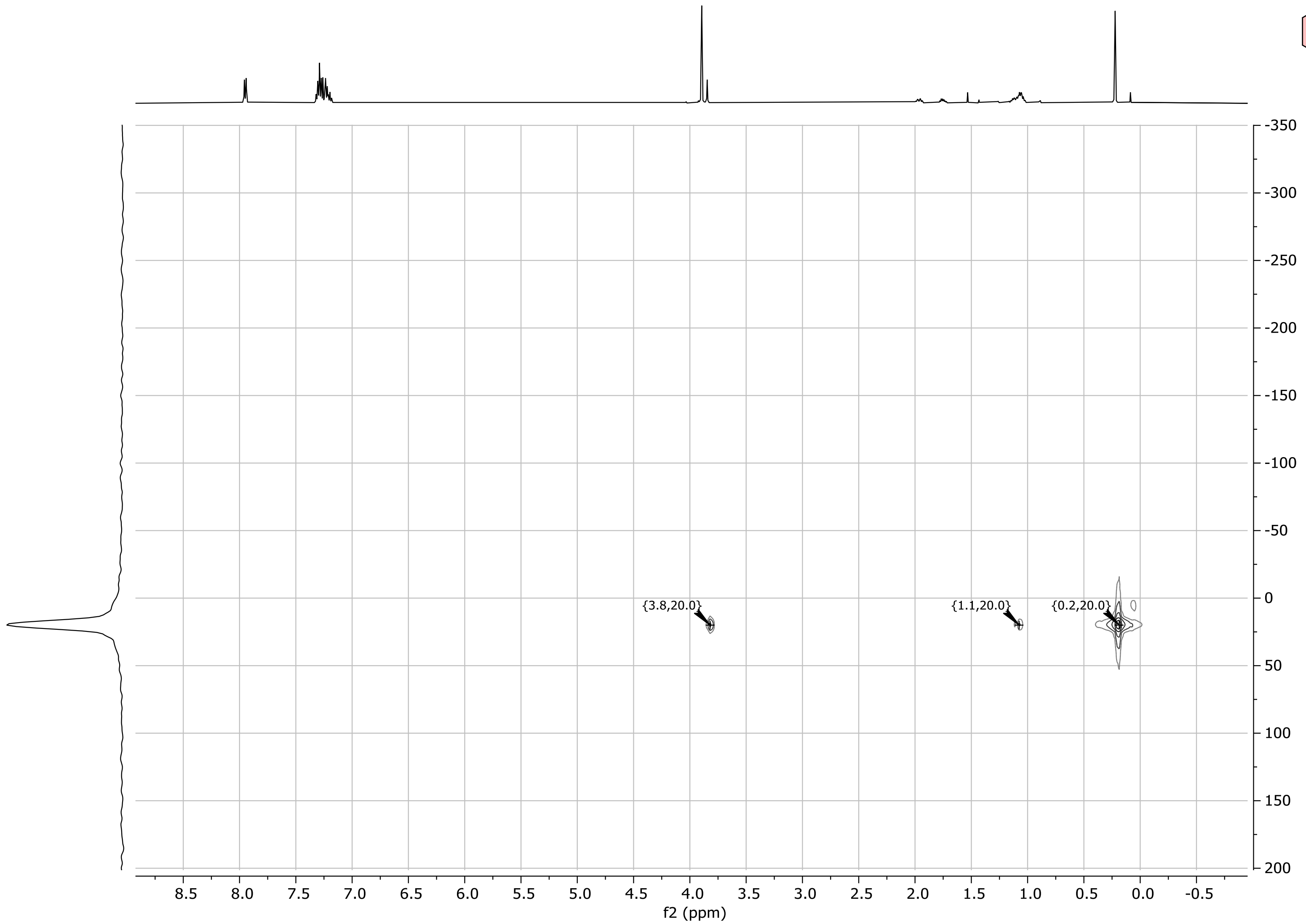

Figure S102. **^{1}H NMR** (500 MHz, $CDCl_3$, 298 K) of **3ba**.

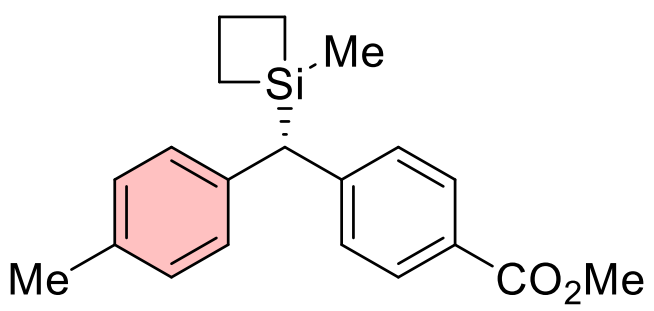

3ba

$C_{20}H_{24}O_2Si$

M_W = 324.50

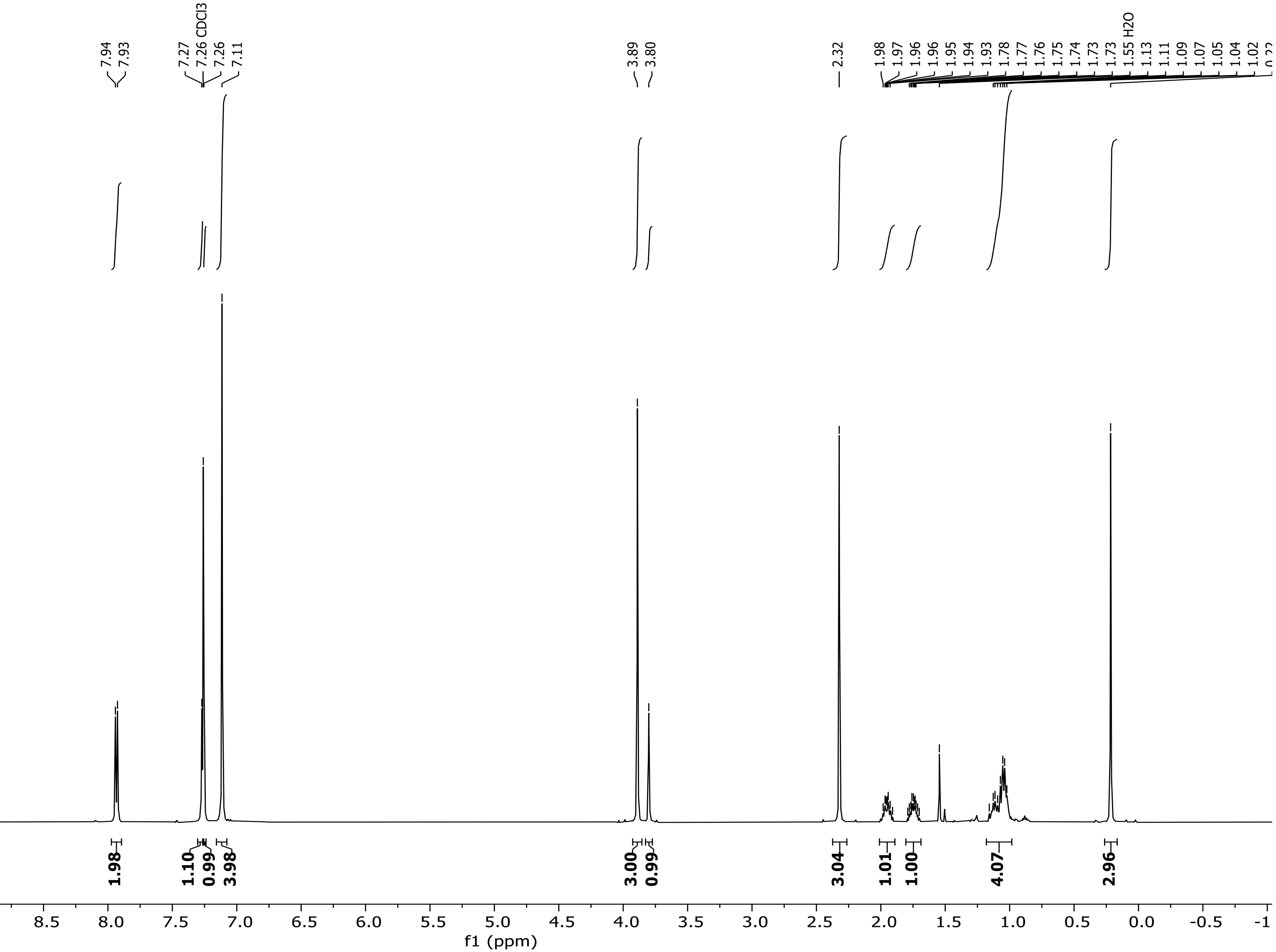

Figure S103. **^{13}C NMR** (125 MHz, $CDCl_3$, 298 K) of **3ba**.

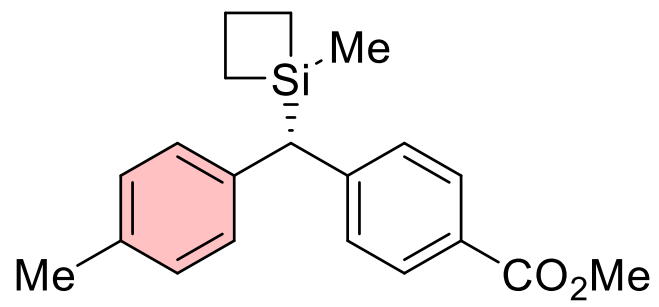

3ba
$C_{20}H_{24}O_2Si$
M_W = 324.50

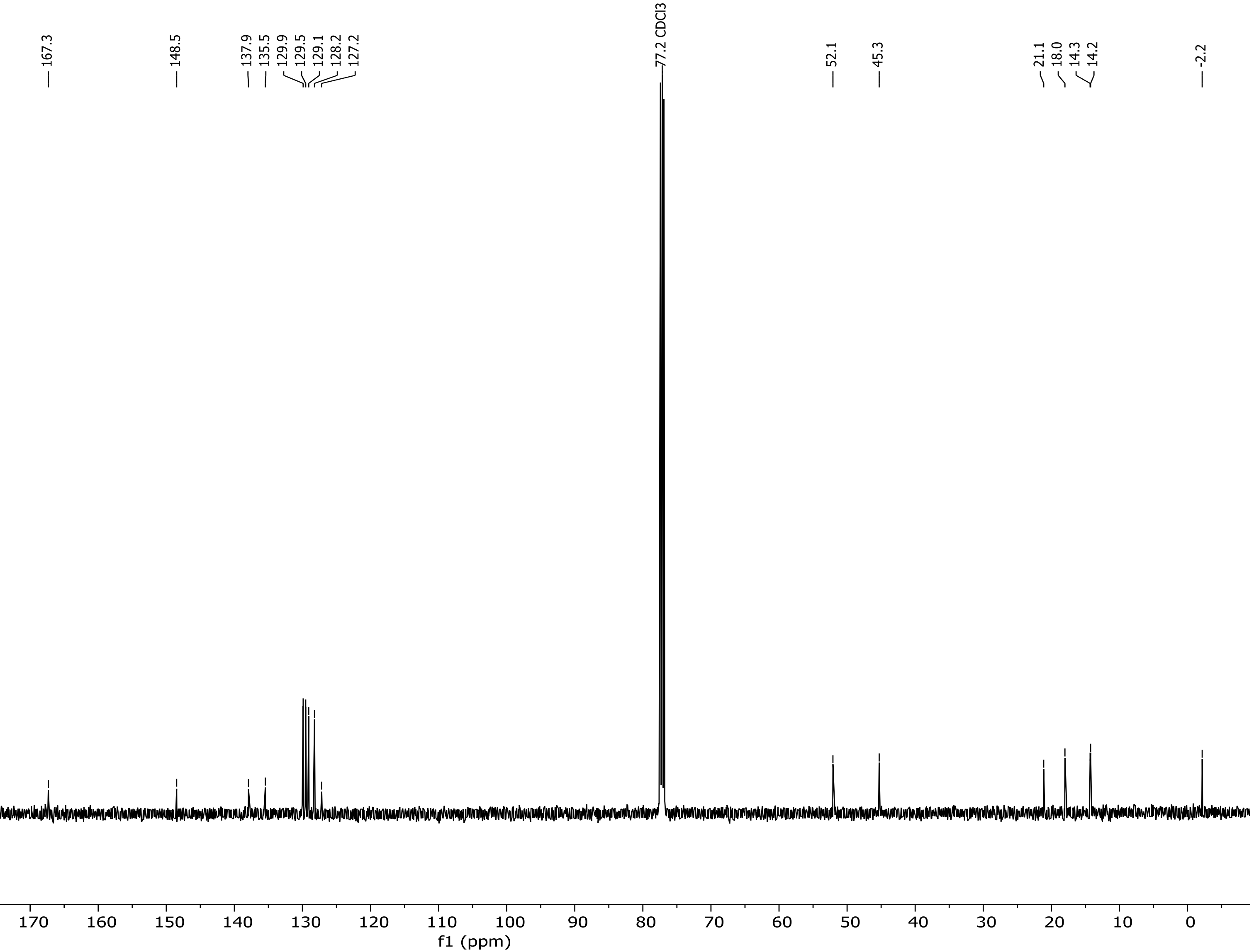

Figure S104. ^{29}Si NMR (^{1}H/^{29}Si HMQC, 99 MHz, $CDCl_3$, optimized for J = 7 Hz) of **3ba.**

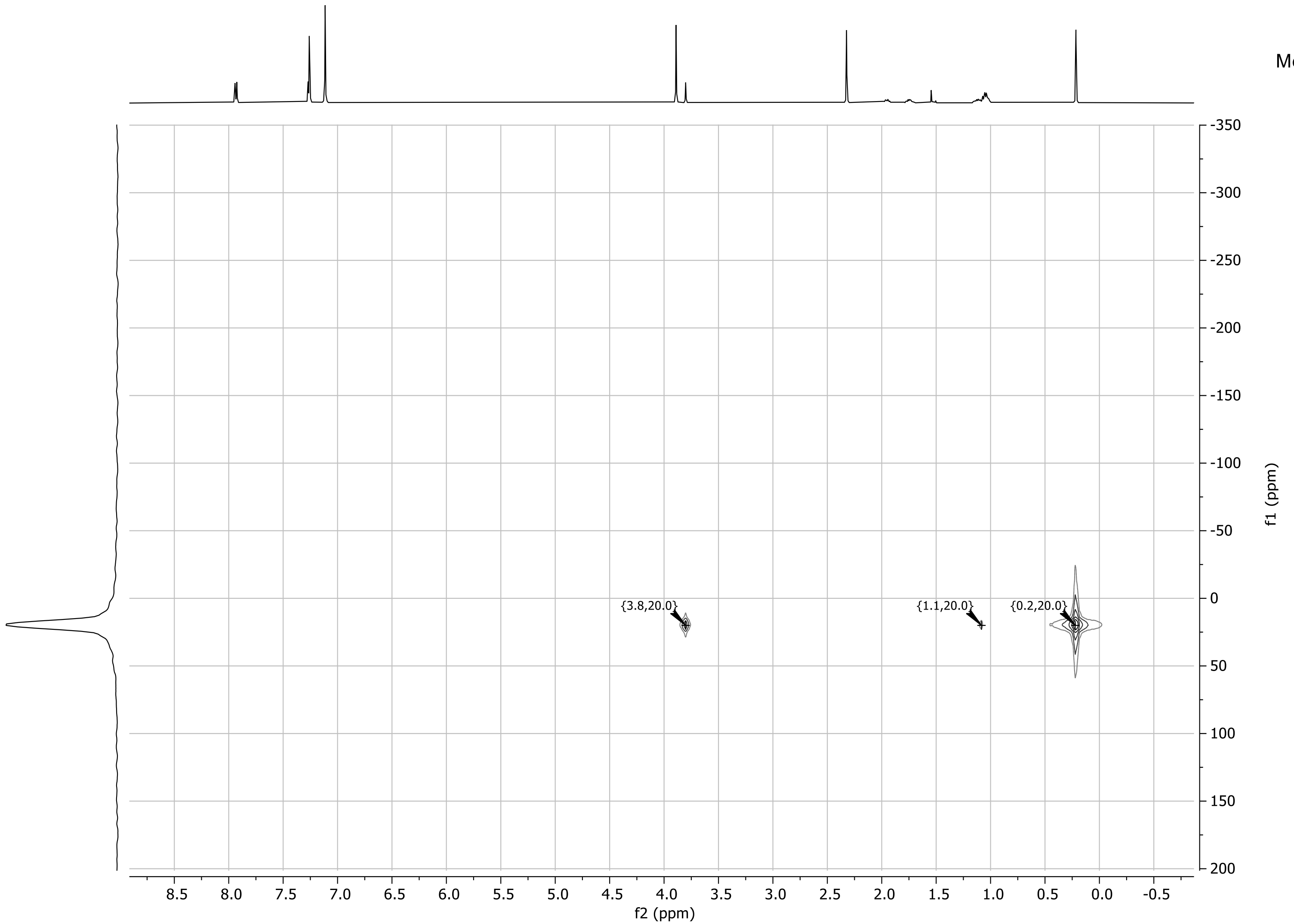

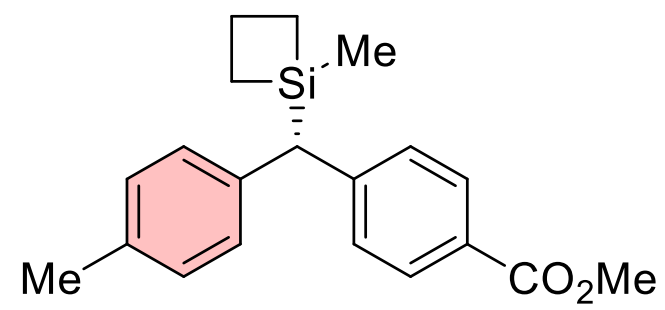

3ba

$C_{20}H_{24}O_2Si$

M_W = 324.50

Figure S105. **^{1}H NMR** (500 MHz, $CDCl_3$, 298 K) of **3ca**.

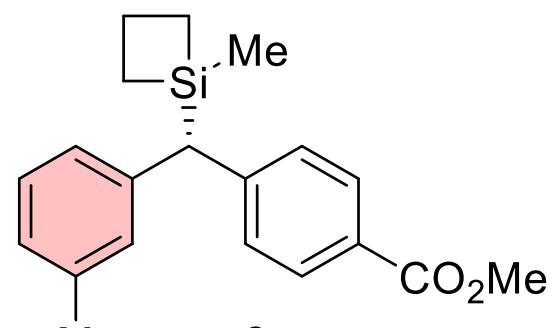

3ca

$C_{20}H_{24}O_2Si$

M_W = 324.50

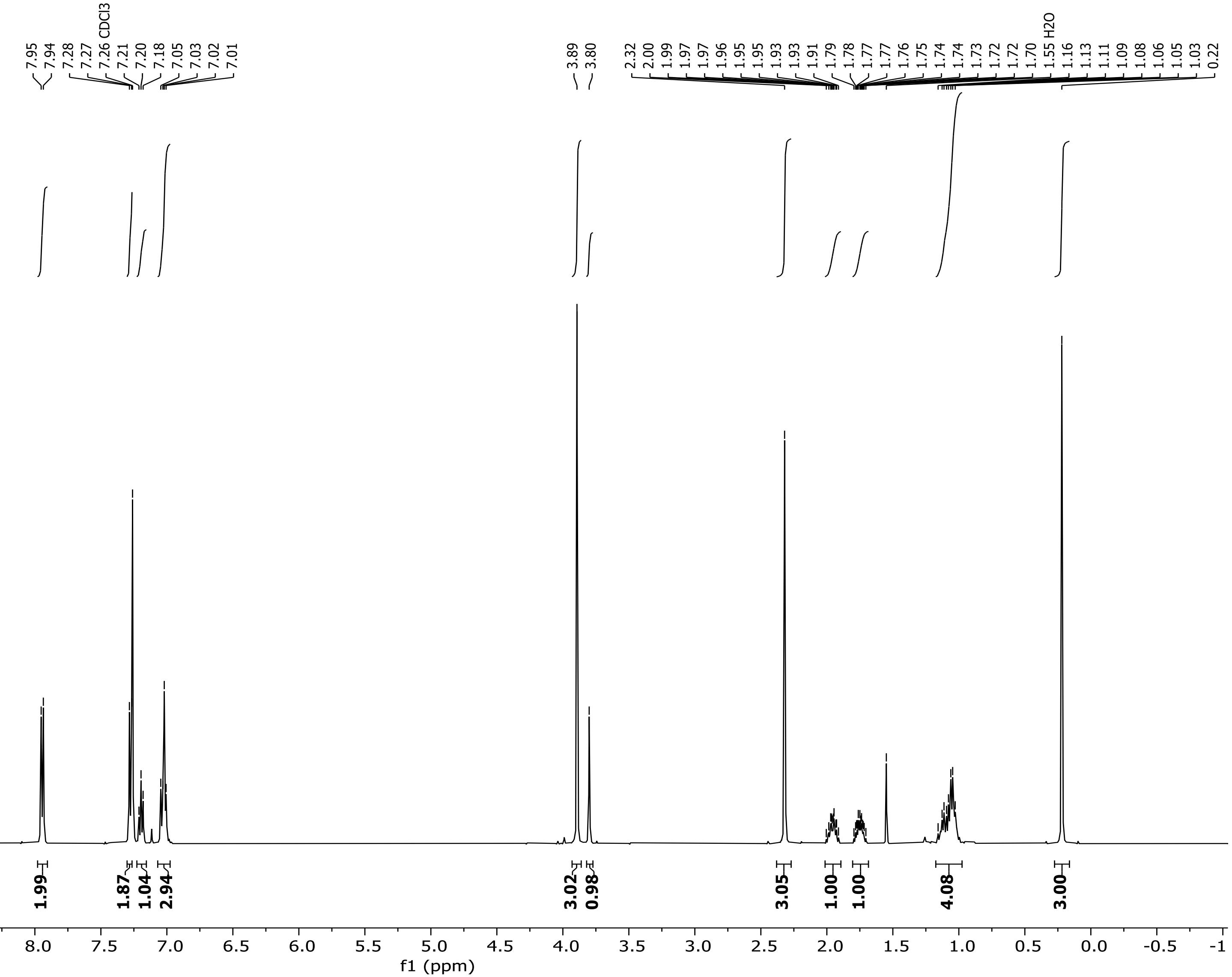

Figure S106. ^{13}C NMR (125 MHz, $CDCl_3$, 298 K) of **3ca**.

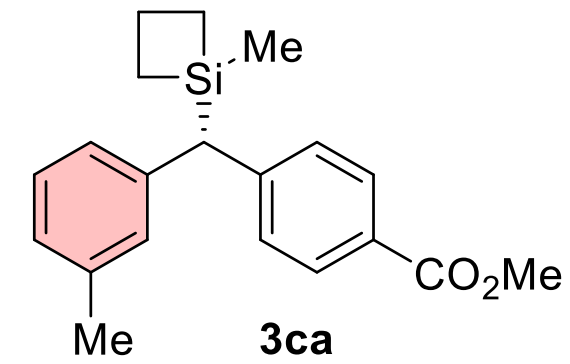

3ca

$C_{20}H_{24}O_2Si$

M_W = 324.50

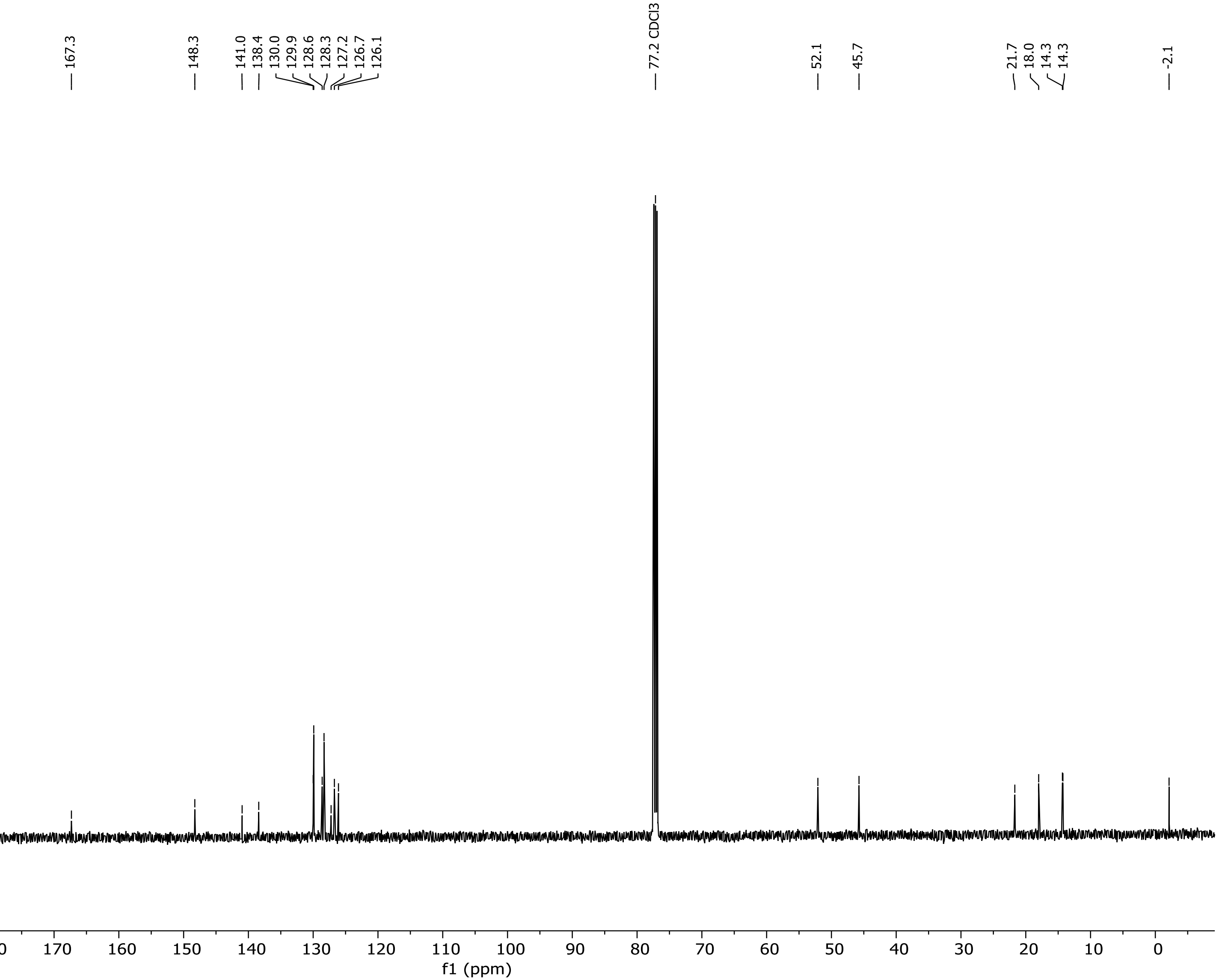

Figure S107. ^{29}Si NMR ($^1H/^{29}Si$ HMQC, 99 MHz, $CDCl_3$, optimized for J = 7 Hz) of **3ca.**

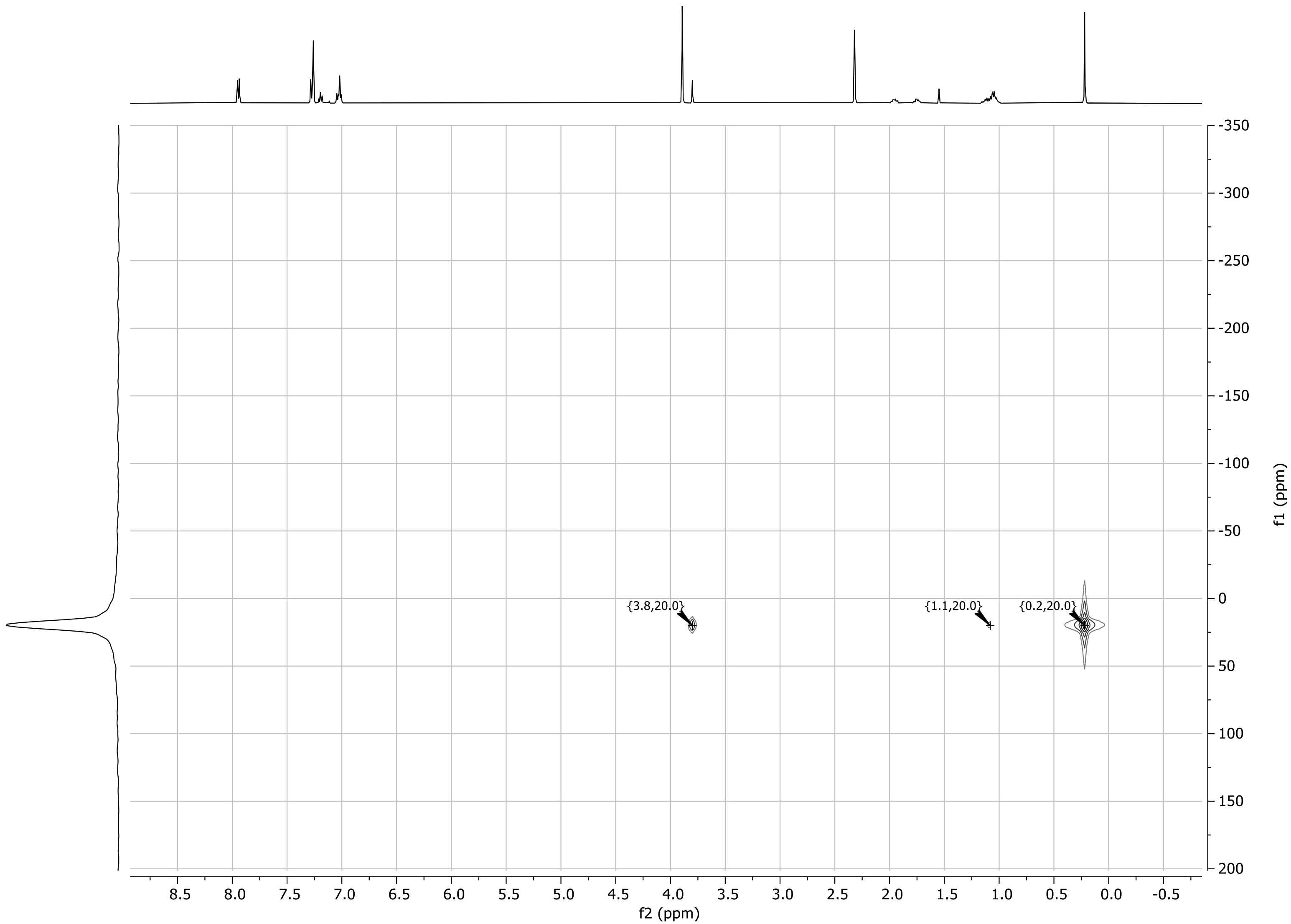

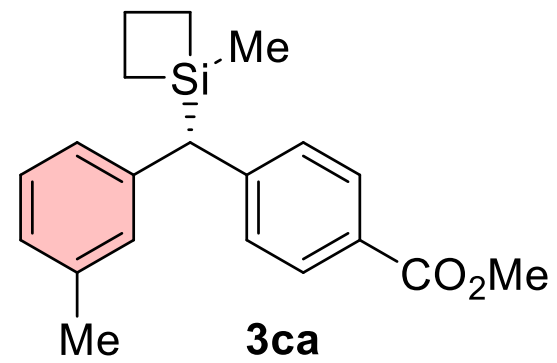

3ca

$C_{20}H_{24}O_2Si$

M_W = 324.50

Figure S108. **^{1}H NMR** (500 MHz, $CDCl_3$, 298 K) of **3ea**.

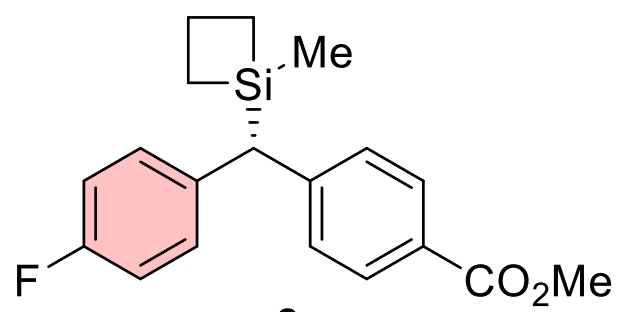

3ea

$C_{19}H_{21}FO_2Si$

M_W = 328.46

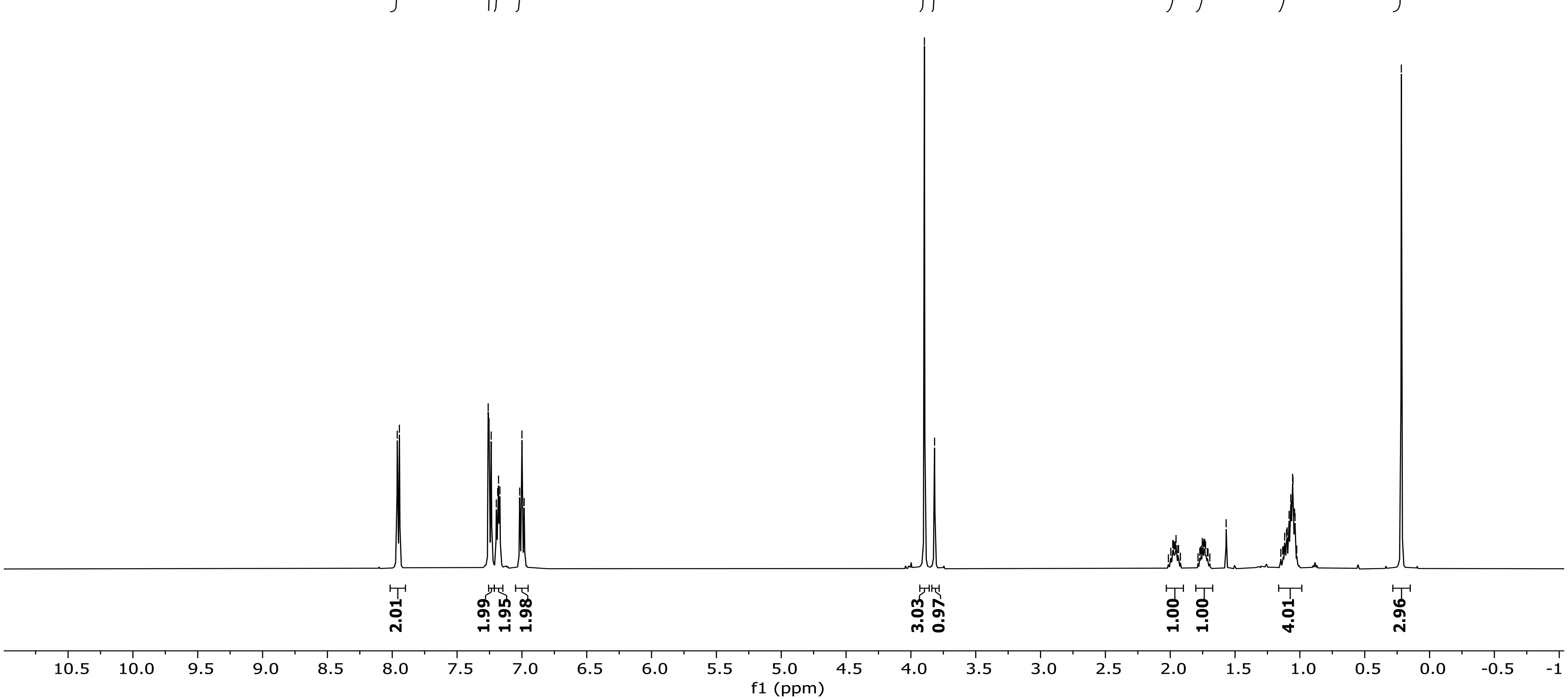

Figure S109. **^{13}C NMR** (125 MHz, $CDCl_3$, 298 K) of **3ea**.

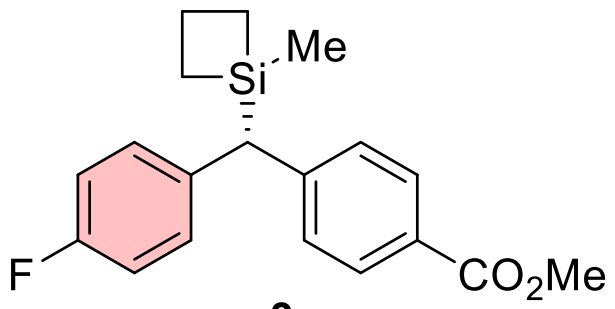

3ea
$C_{19}H_{21}FO_2Si$
M_W = 328.46

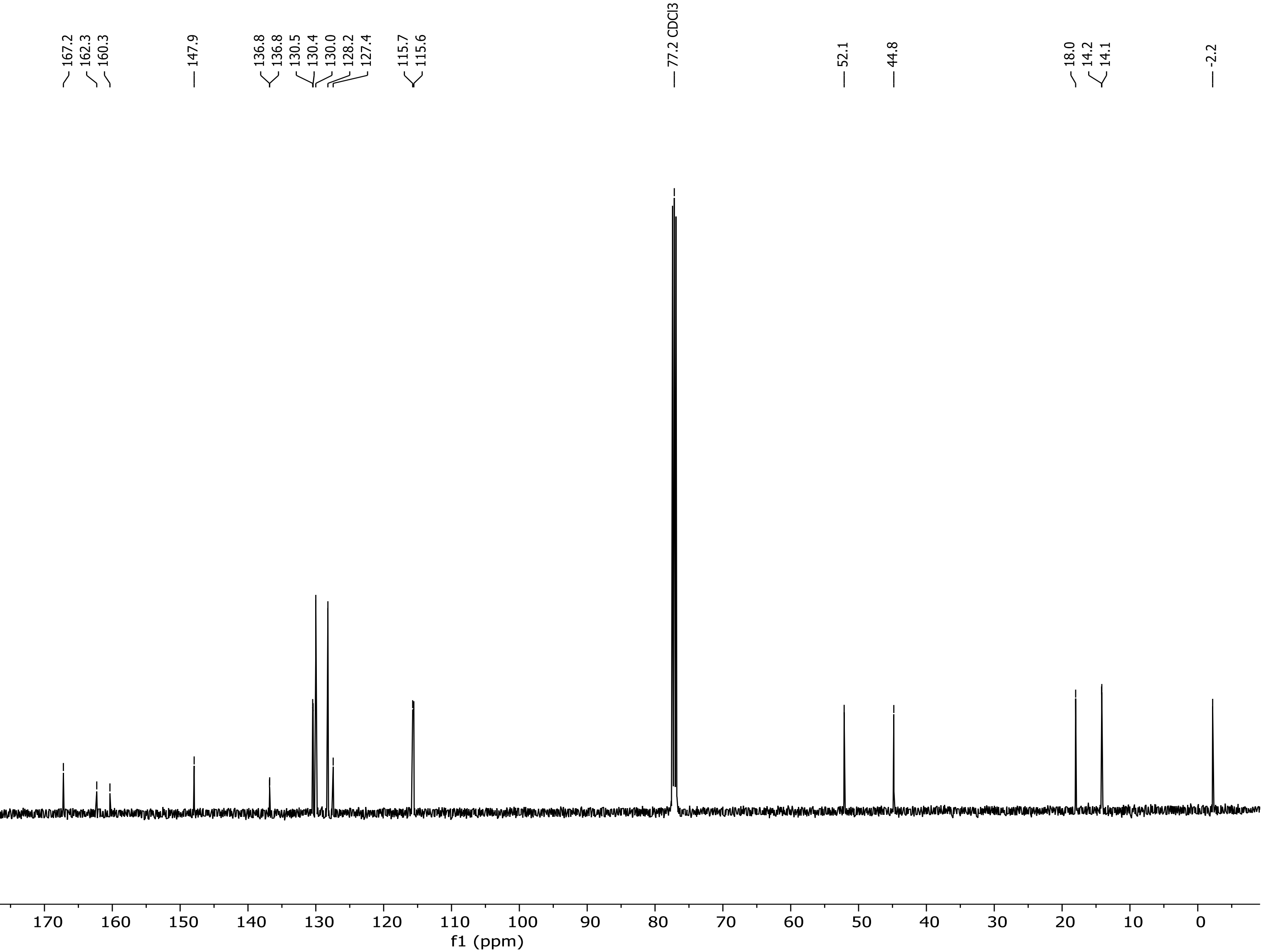

Figure S110. ^{29}Si NMR ($^1H/^{29}Si$ HMQC, 99 MHz, $CDCl_3$, optimized for J = 7 Hz) of **3ea.**

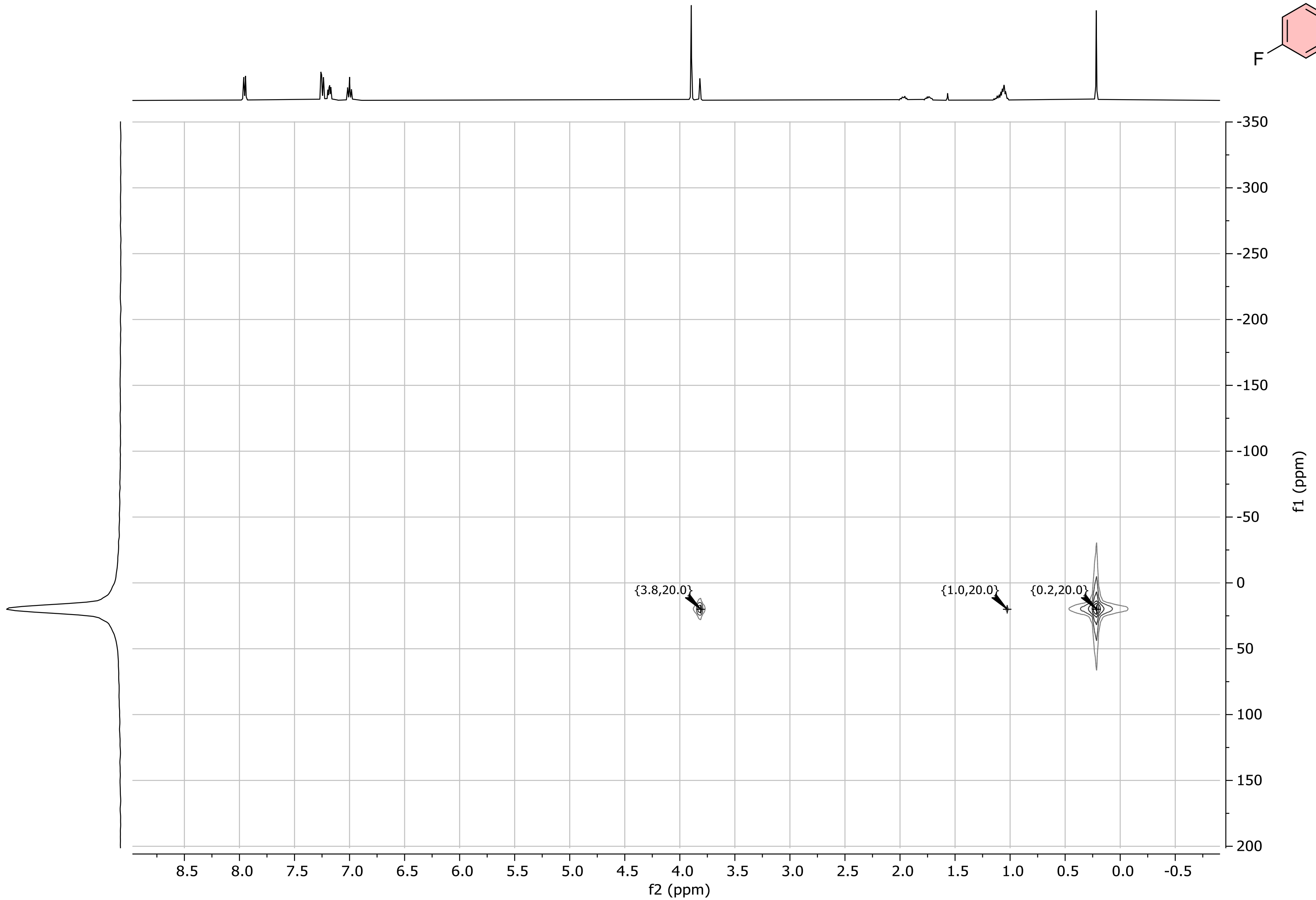

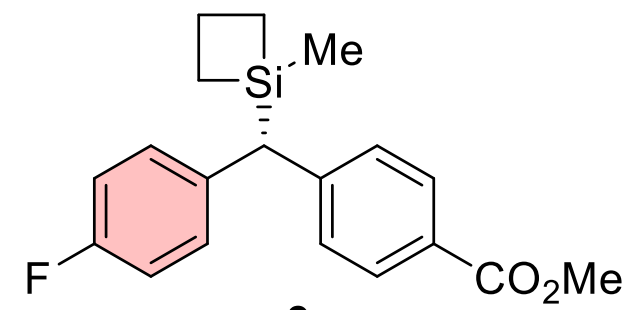

3ea
$C_{19}H_{21}FO_2Si$
M_W = 328.46

Figure S111. **^{19}F NMR** (470 MHz, $CDCl_3$, 298 K) of **3ea**.

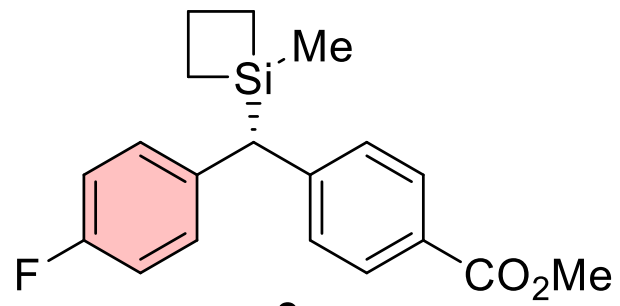

3ea

$C_{19}H_{21}FO_2Si$

M_W = 328.46

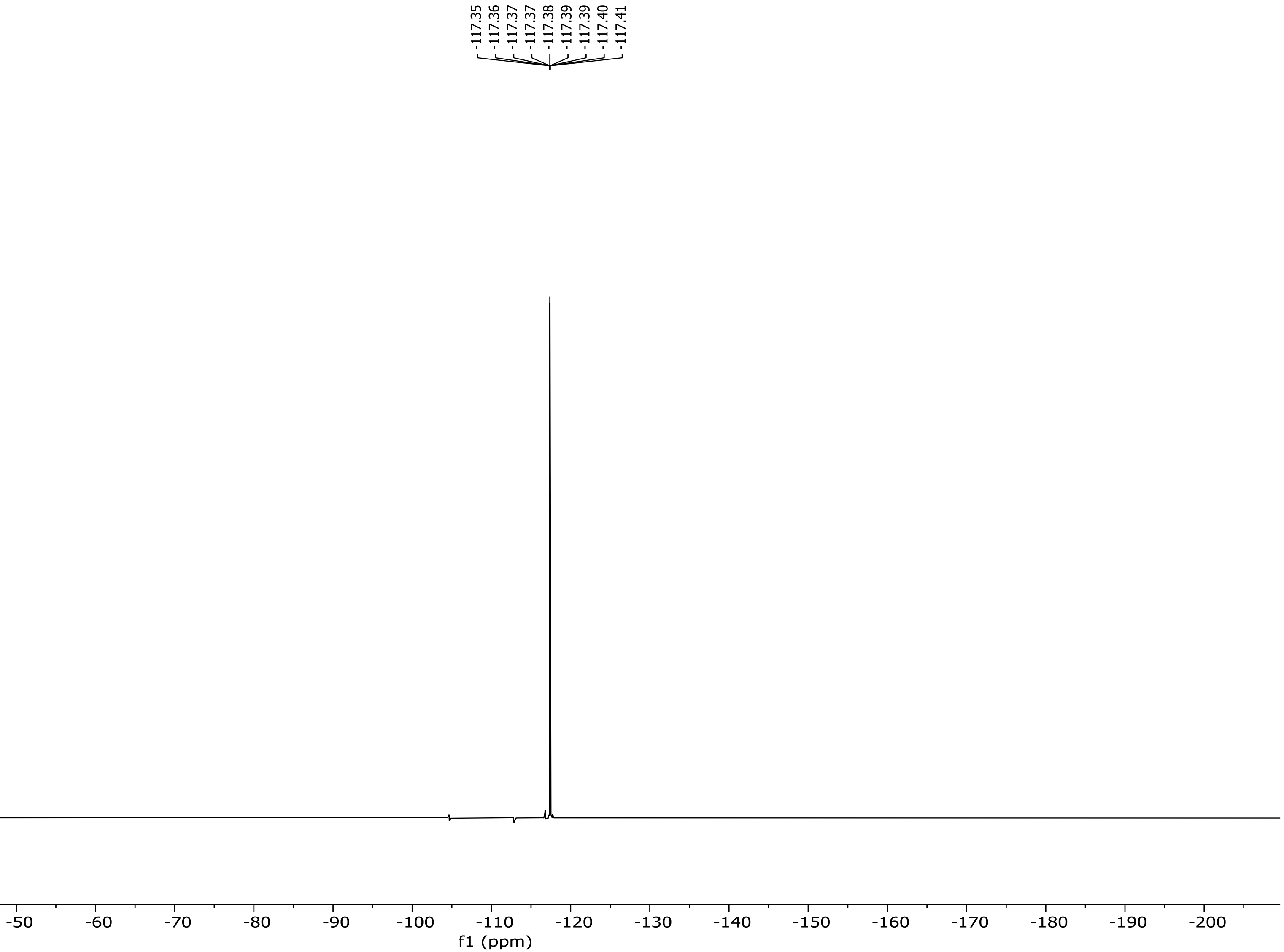

Figure S112. **¹H NMR** (500 MHz, $CDCl_3$, 298 K) of **3fa**.

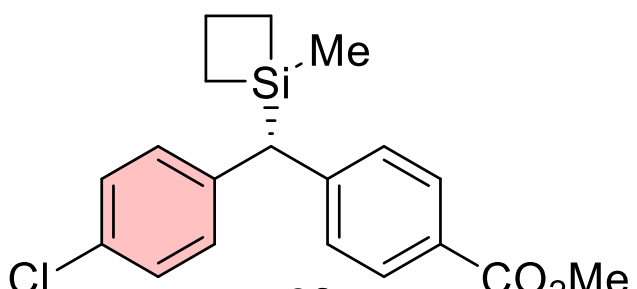

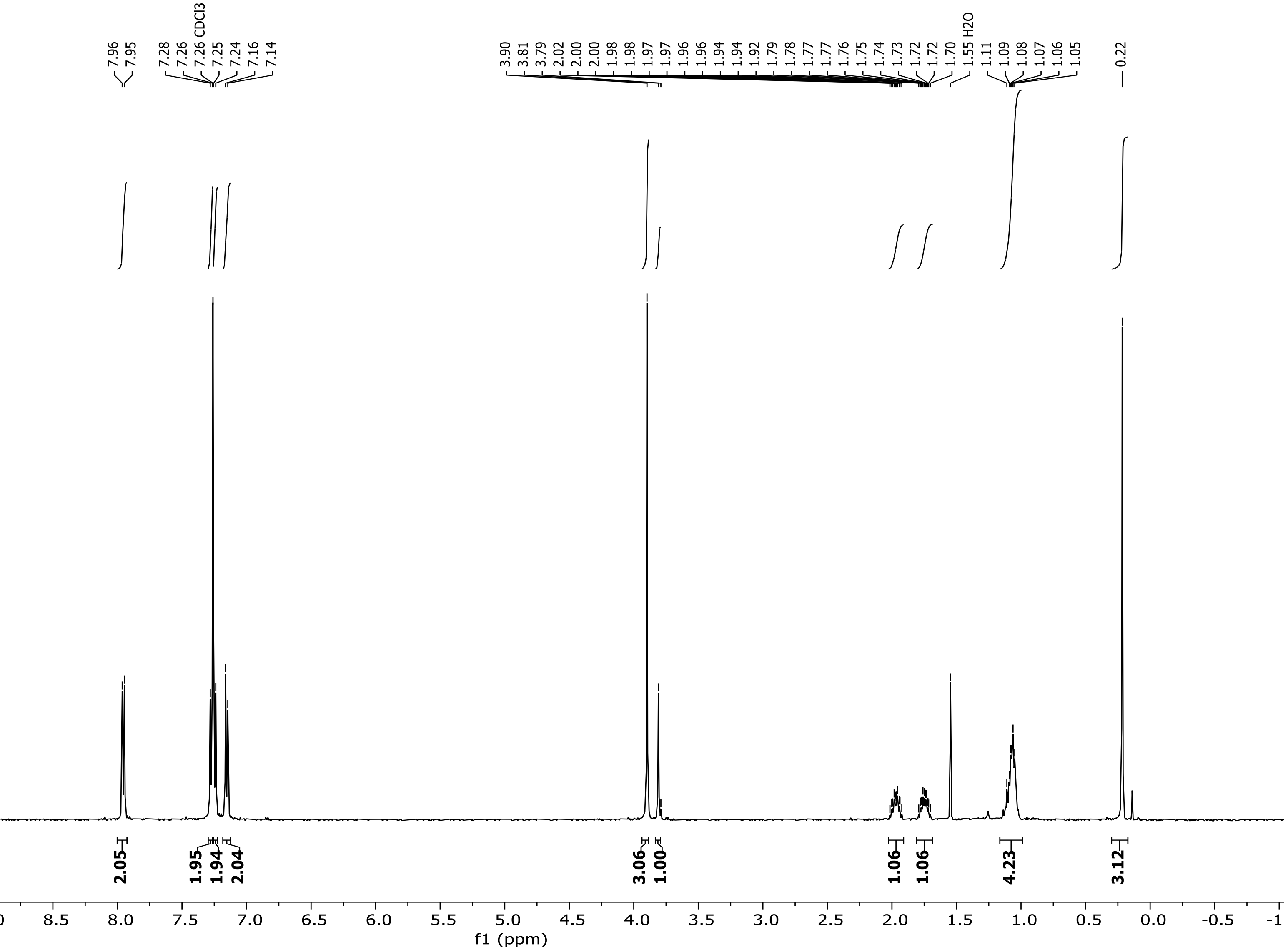

Figure S113. ^{13}**C NMR** (125 MHz, $CDCl_3$, 298 K) of **3fa**.

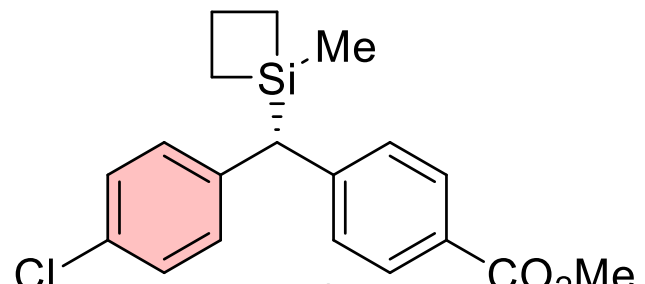

3fa
$C_{19}H_{21}ClO_2Si$
M_W = 344.91

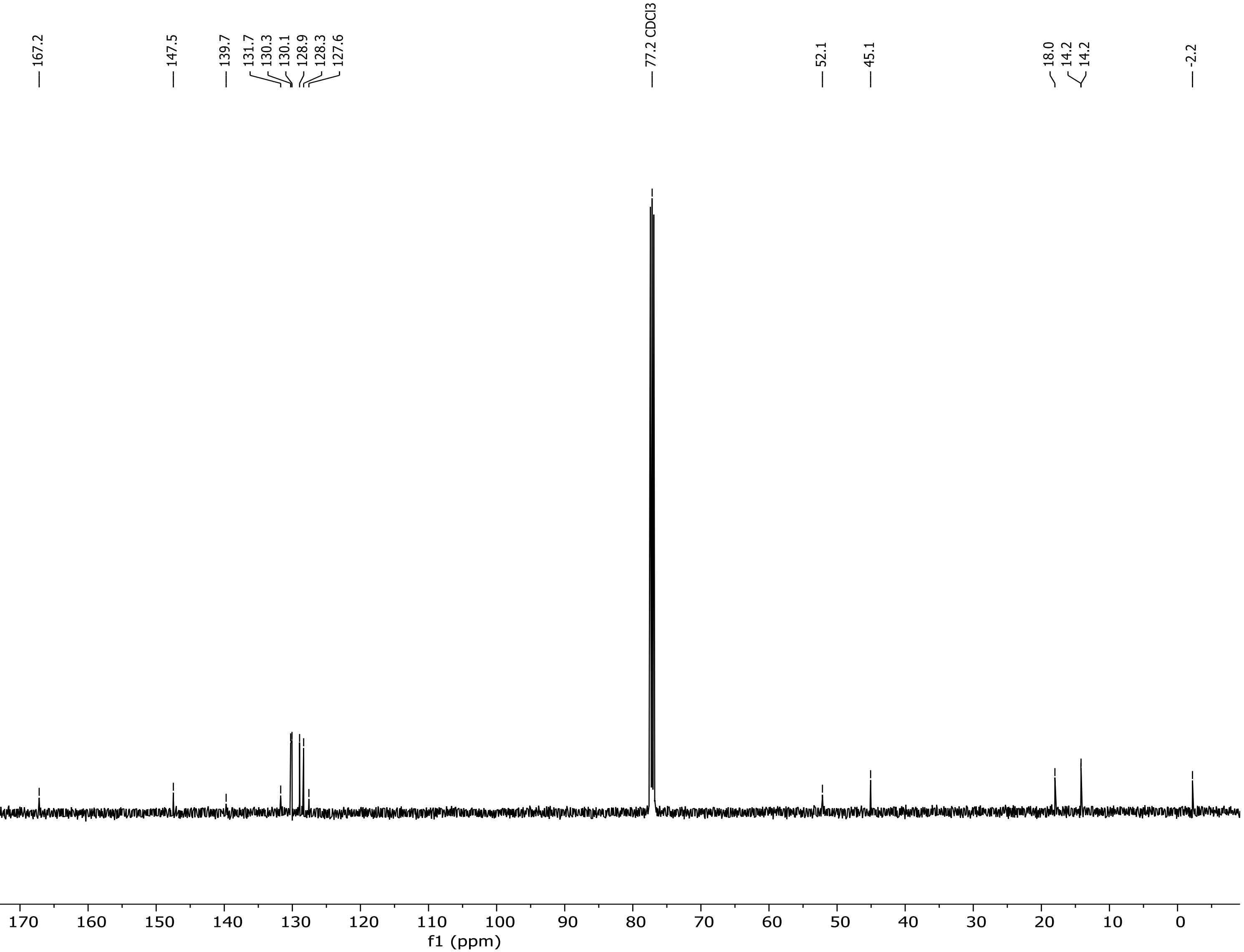

Figure S114. ^{29}Si NMR (^{1}H/^{29}Si HMQC, 99 MHz, $CDCl_3$, optimized for *J* = 7 Hz) of **3fa.**

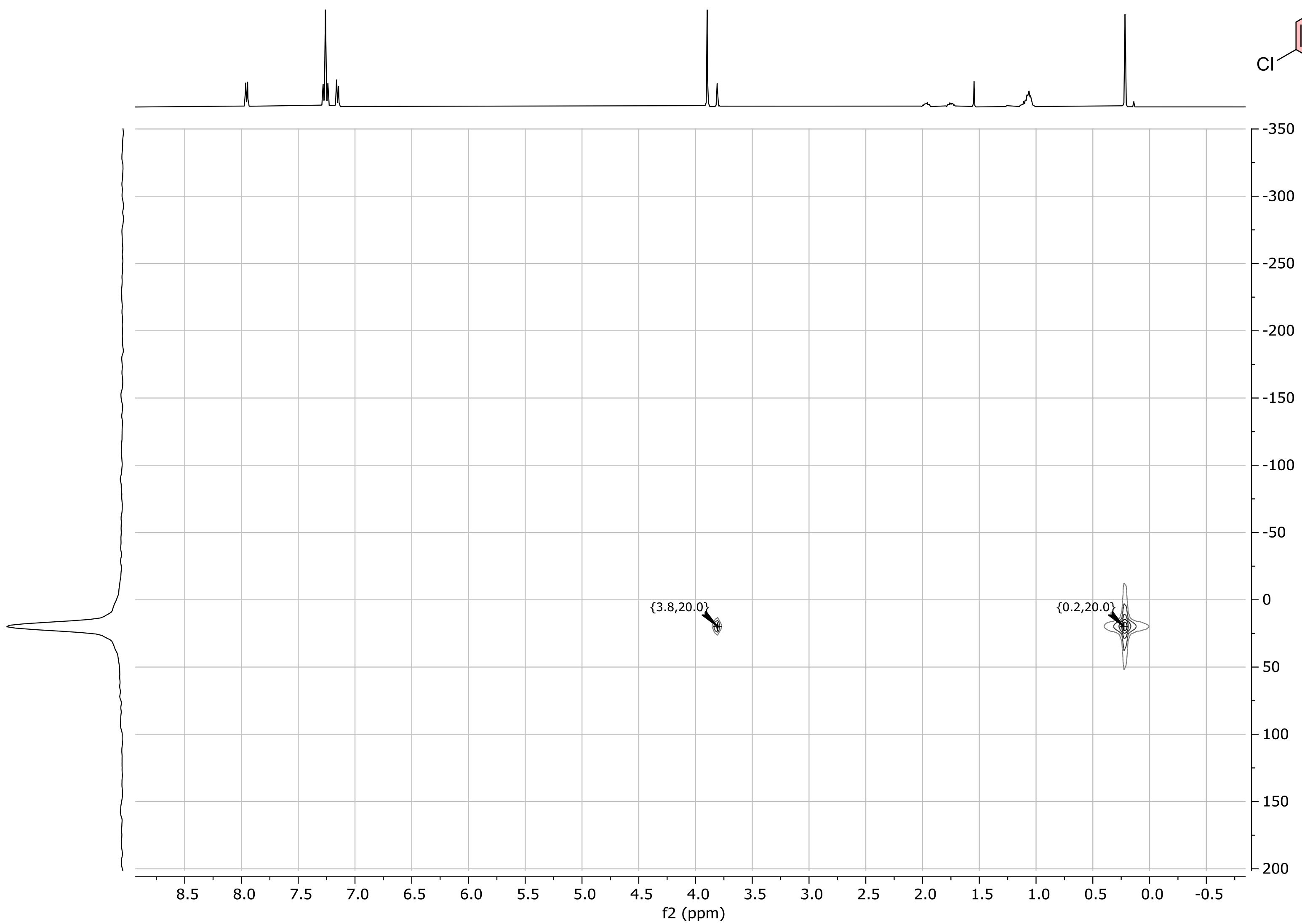

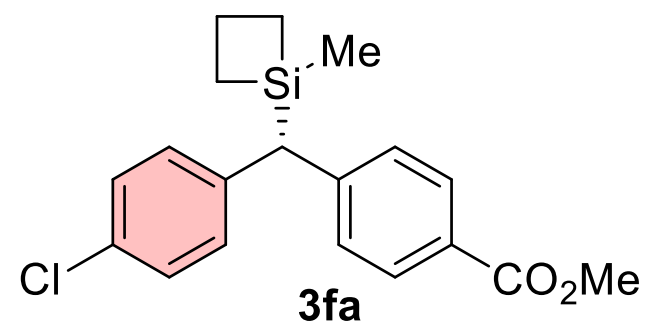

3fa
$C_{19}H_{21}ClO_2Si$
M_W = 344.91

Figure S115. **^{1}H NMR** (500 MHz, $CDCl_3$, 298 K) of **3ga**.

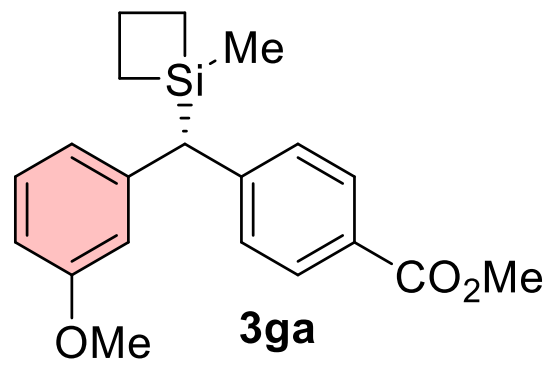

3ga

$C_{20}H_{24}O_3Si$

M_W = 340.49

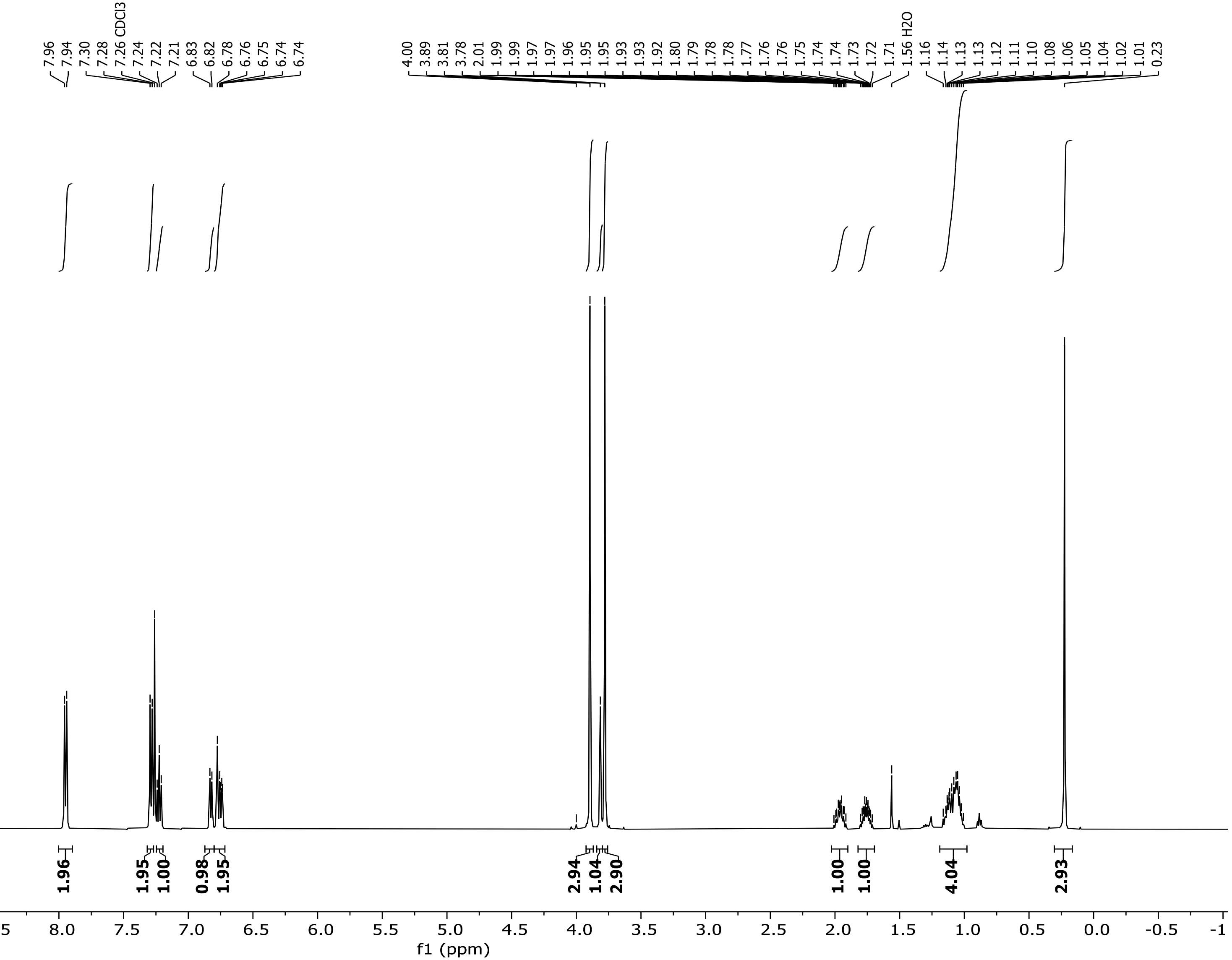

Figure S116. ^{13}**C NMR** (125 MHz, $CDCl_3$, 298 K) of **3ga**.

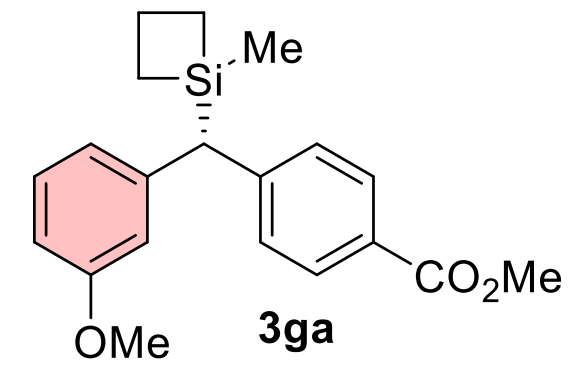

3ga

$C_{20}H_{24}O_3Si$

M_W = 340.49

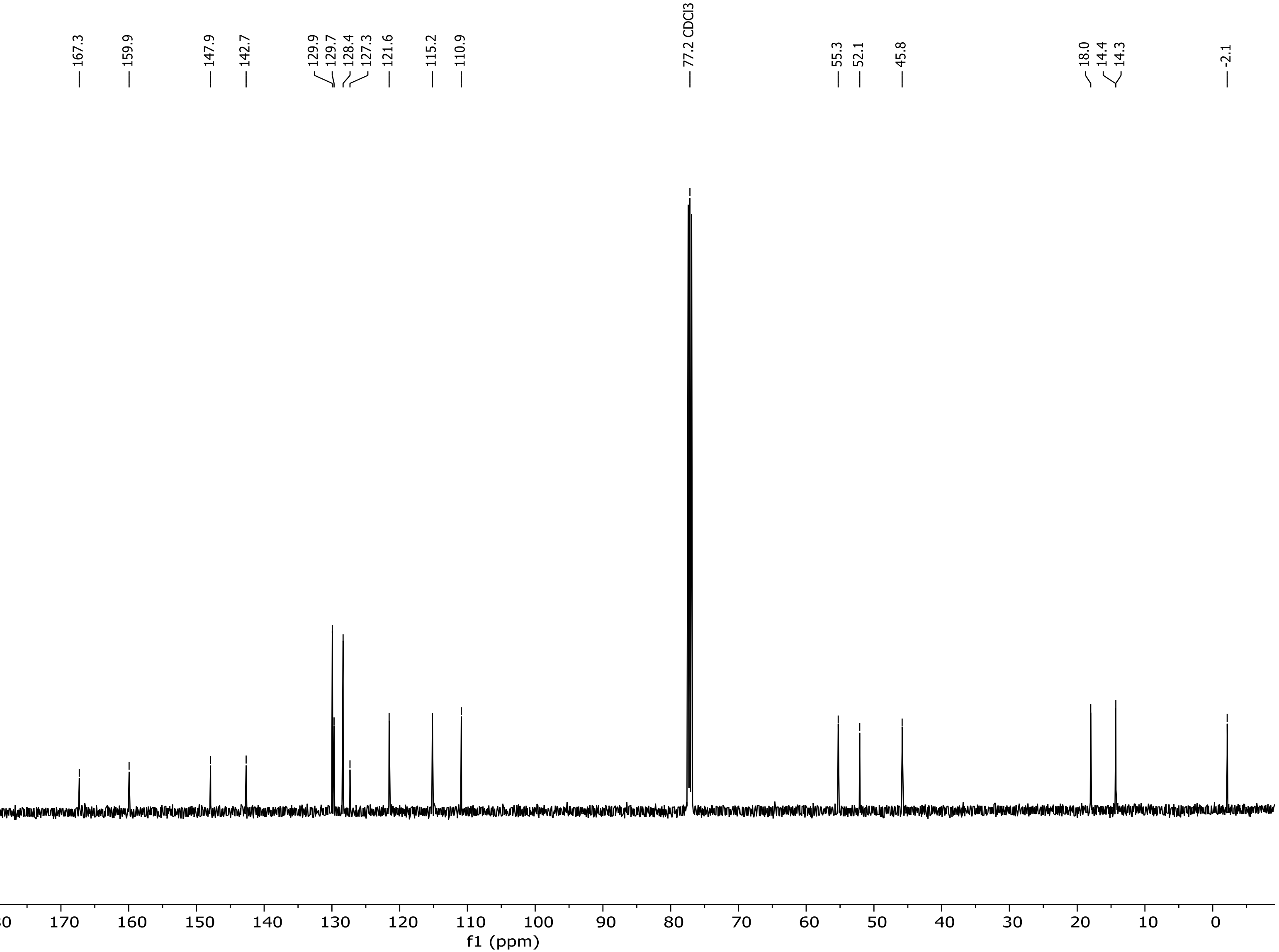

Figure S117. ^{29}Si NMR ($^{1}H/^{29}Si$ HMQC, 99 MHz, $CDCl_3$, optimized for J = 7 Hz) of **3aa.**

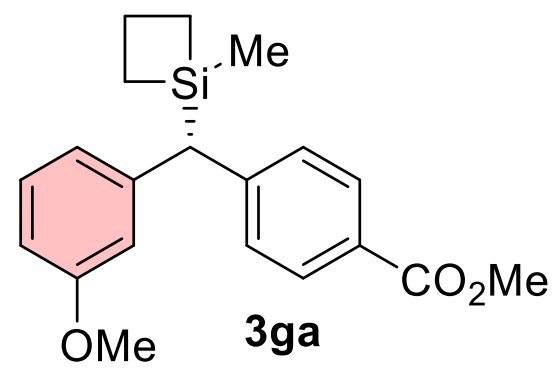

3ga
$C_{20}H_{24}O_3Si$
M_W = 340.49

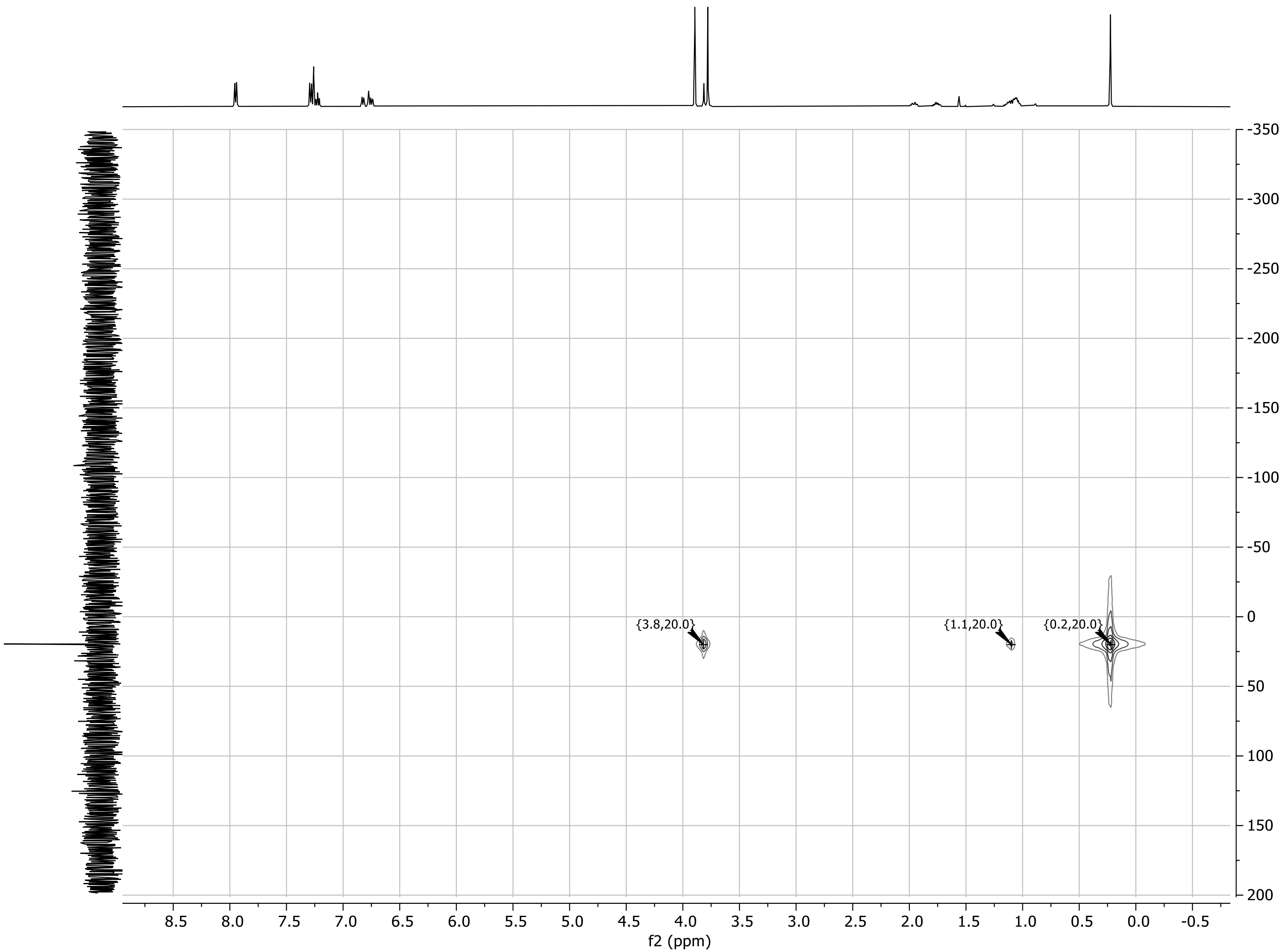

Figure S118. ¹H NMR (500 MHz, $CDCl_3$, 298 K) of **3ab**.

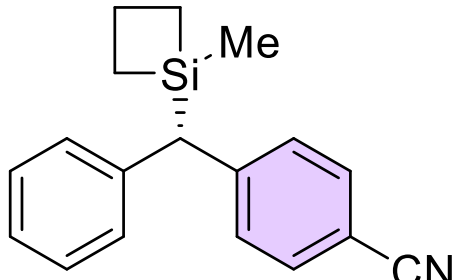

3ab
$C_{18}H_{19}NSi$
M_W = 277.44

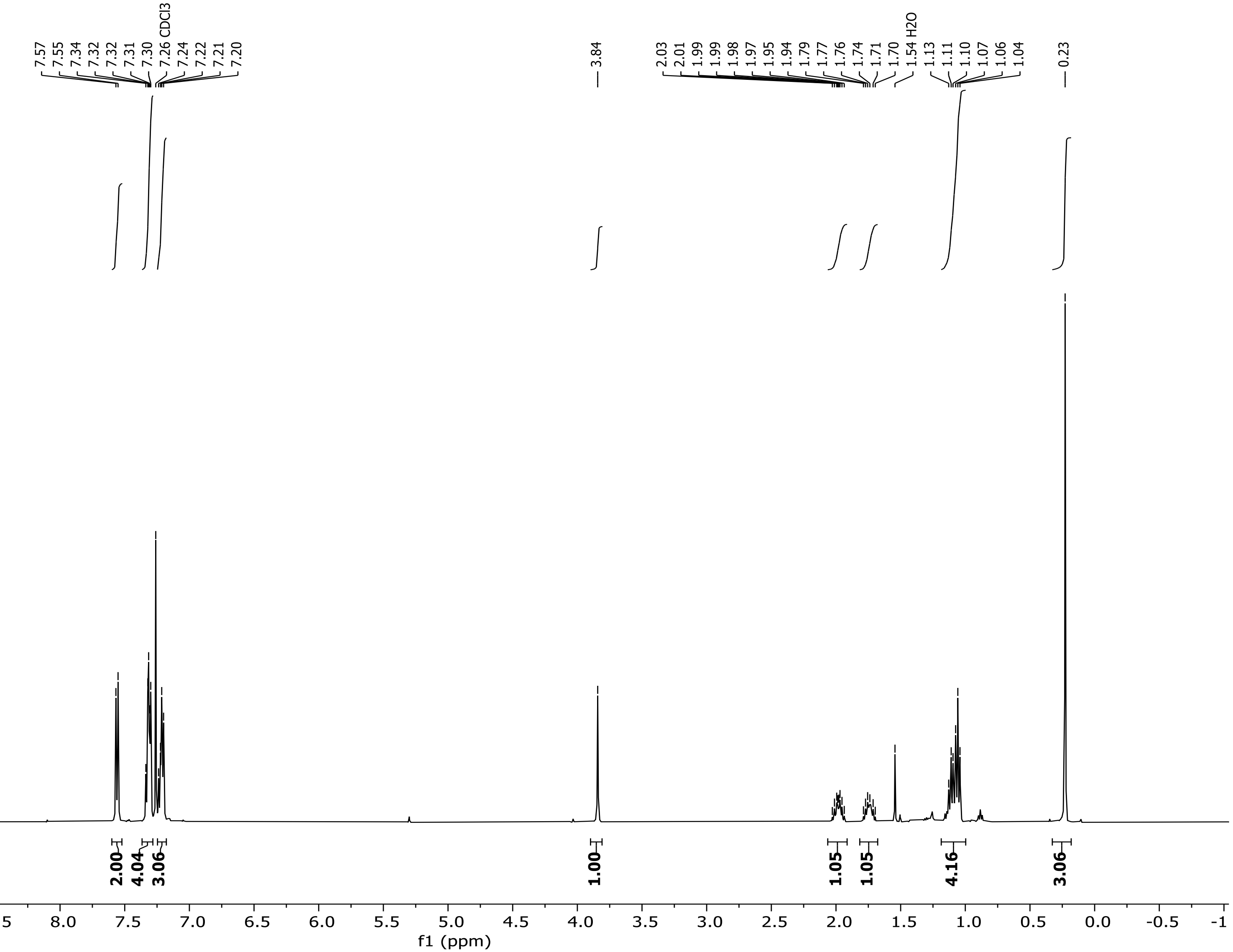

Figure S119. ^{13}**C NMR** (125 MHz, $CDCl_3$, 298 K) of **3ab**.

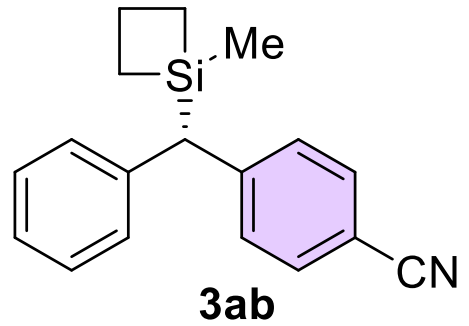

3ab
$C_{18}H_{19}NSi$
M_W = 277.44

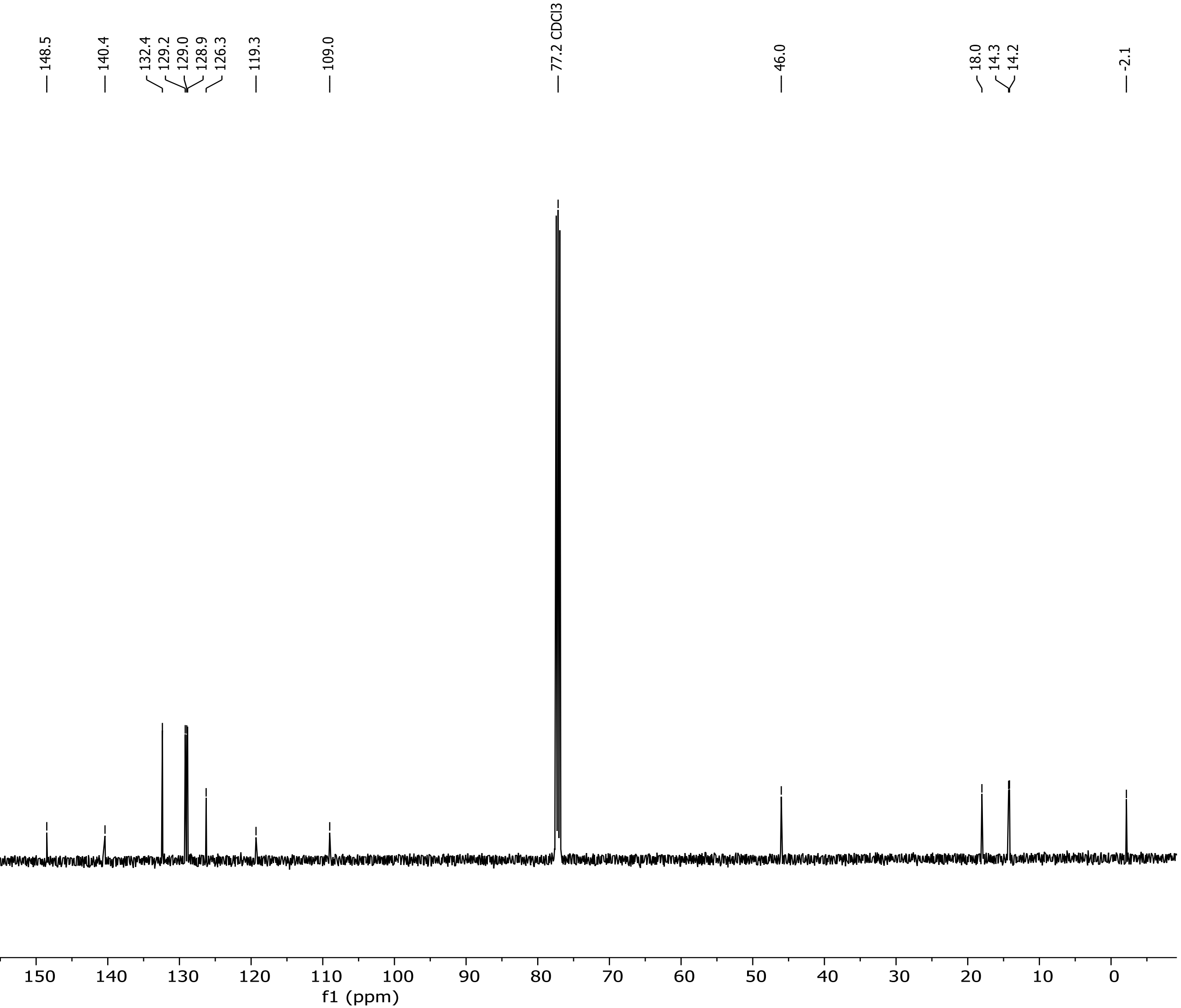

Figure S120. ^{29}Si NMR ($^{1}H/^{29}Si$ HMQC, 99 MHz, $CDCl_3$, optimized for J = 7 Hz) of **3ab.**

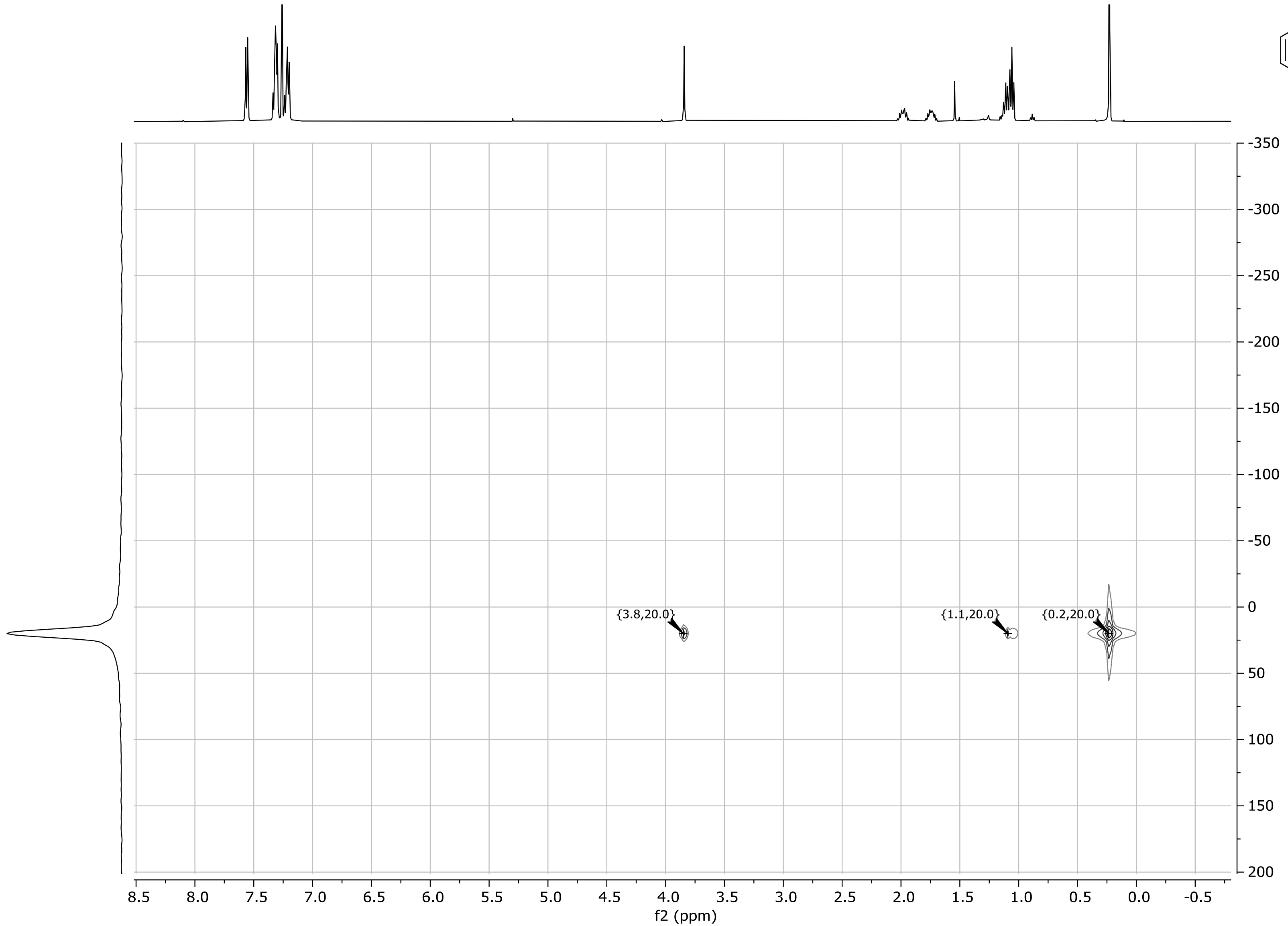

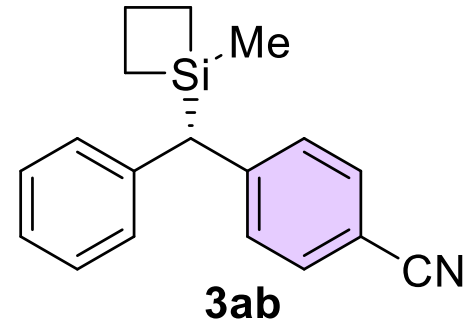

3ab
$C_{18}H_{19}NSi$
M_W = 277.44

Figure S121. **^{1}H NMR** (500 MHz, $CDCl_3$, 298 K) of **3ac**.

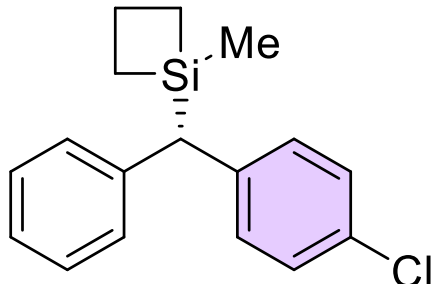

3ac

$C_{17}H_{19}ClSi$

M_W = 286.87

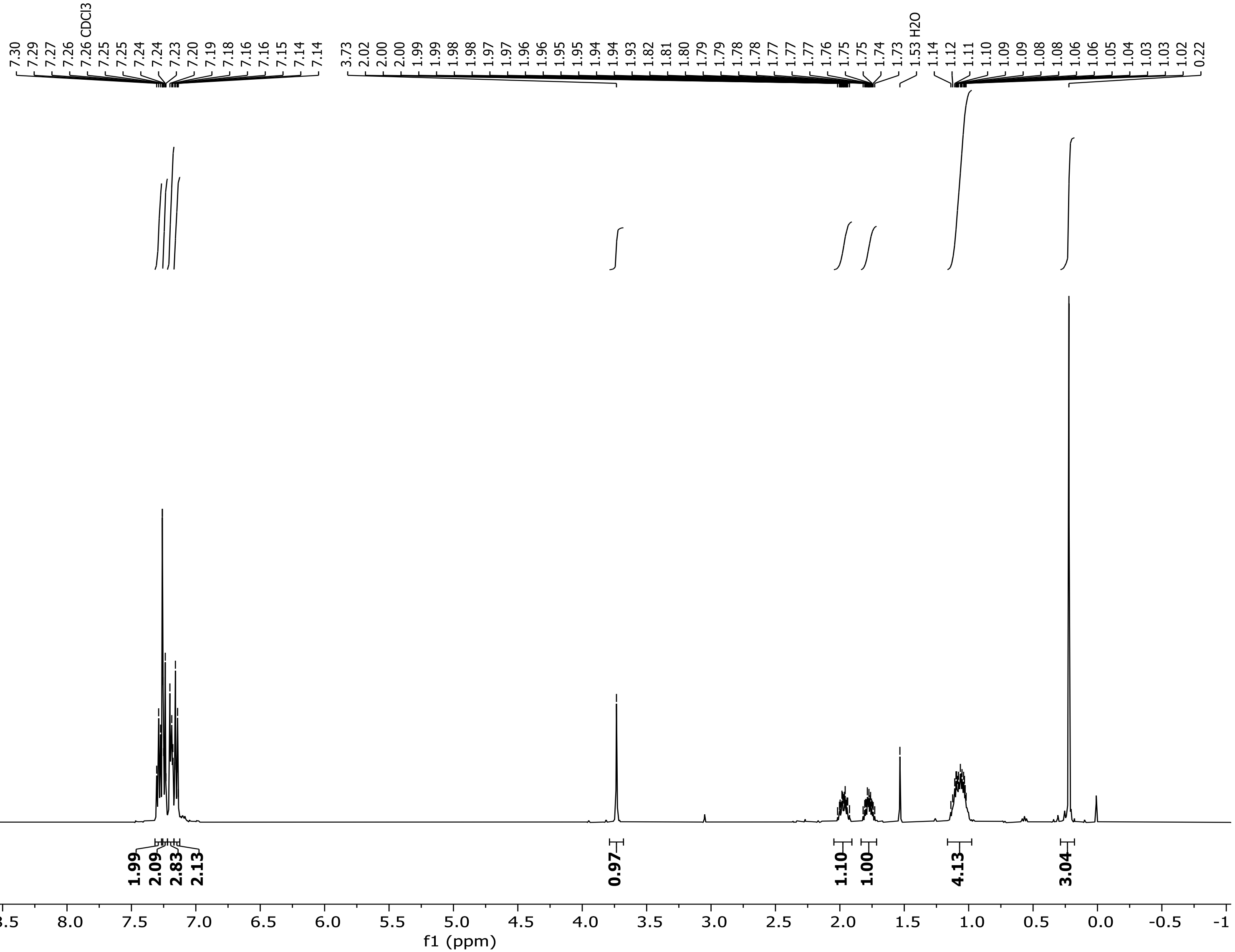

Figure S122. ^{13}**C NMR** (125 MHz, $CDCl_3$, 298 K) of **3ac**.

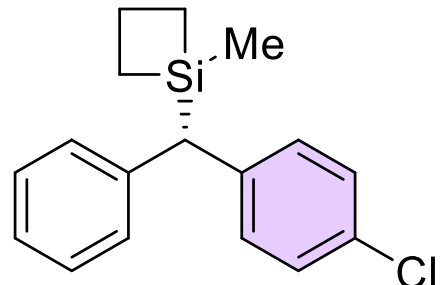

3ac
$C_{17}H_{19}ClSi$
M_W = 286.87

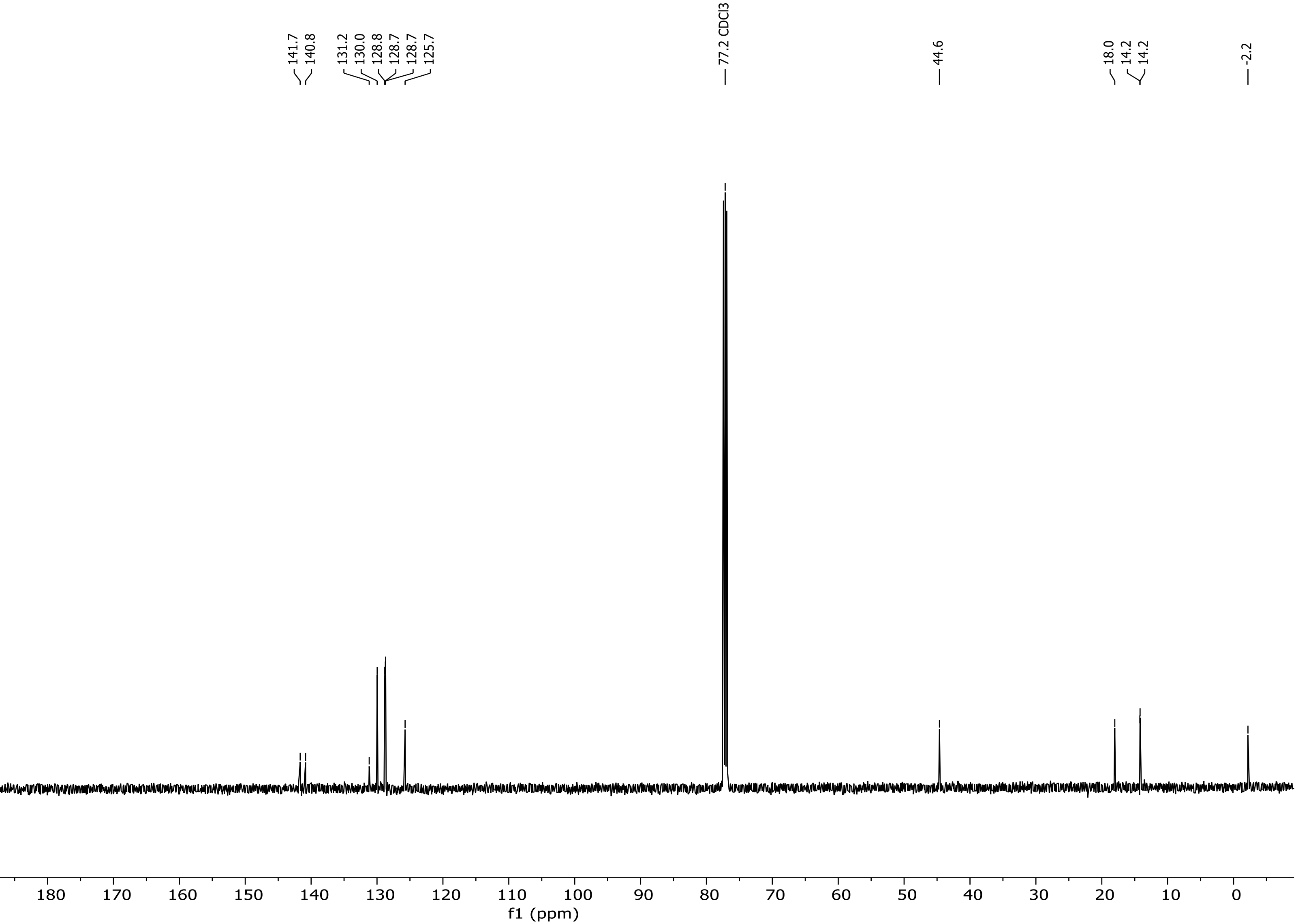

Figure S123. ^{29}**Si NMR** (^{1}H/^{29}Si HMQC, 99 MHz, $CDCl_3$, optimized for J = 7 Hz) of **3ac.**

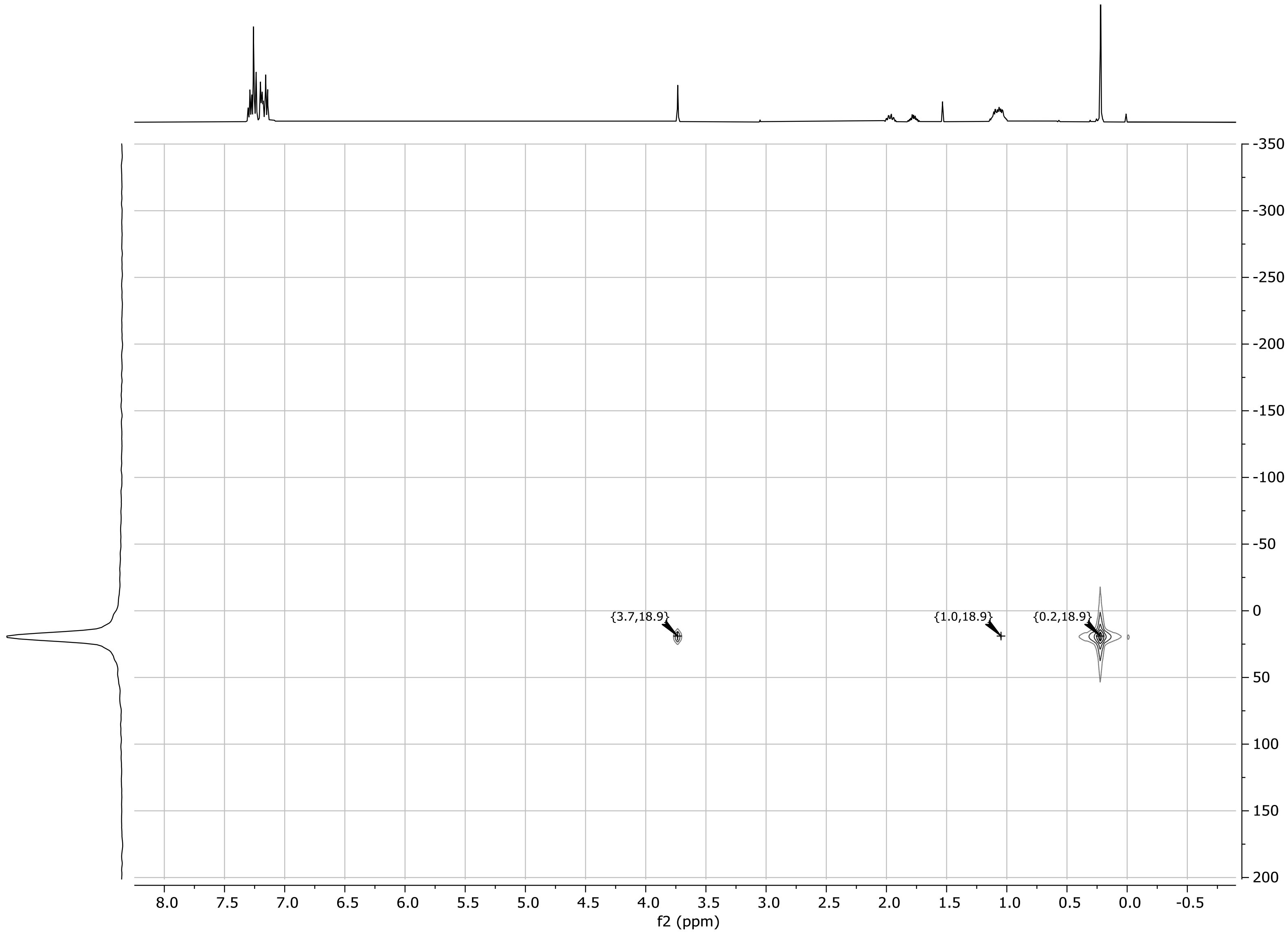

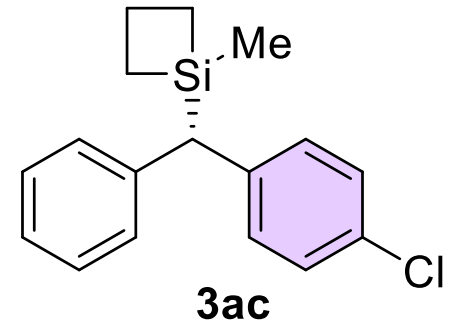

3ac

$C_{17}H_{19}ClSi$

M_W = 286.87

Figure S124. ¹H NMR (500 MHz, $CDCl_3$, 298 K) of **3ae**.

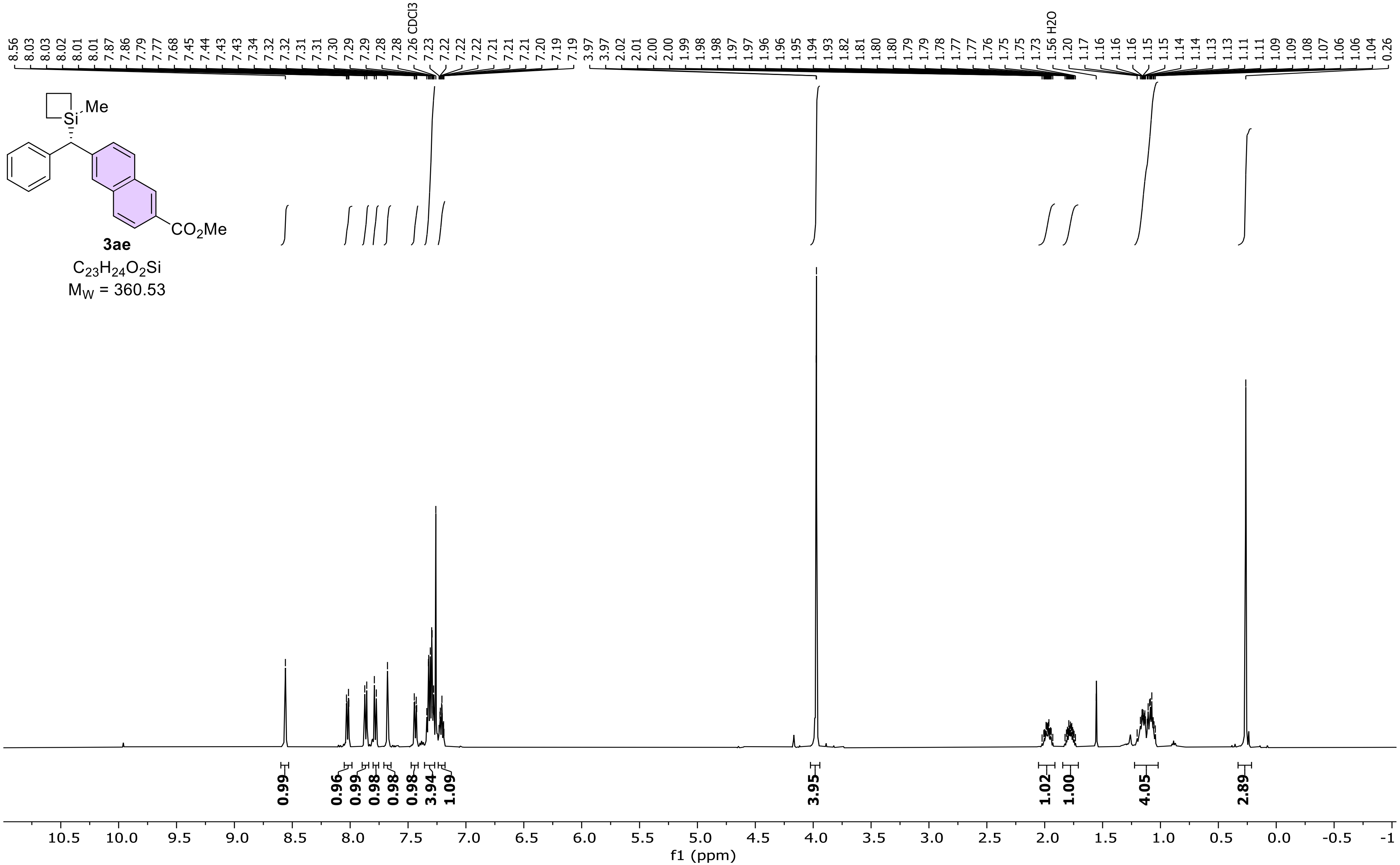

Figure S125. ^{13}C NMR (125 MHz, $CDCl_3$, 298 K) of **3ae**.

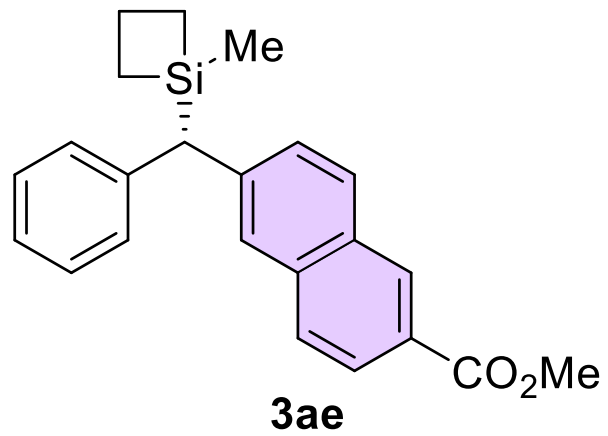

3ae
$C_{23}H_{24}O_2Si$
M_W = 360.53

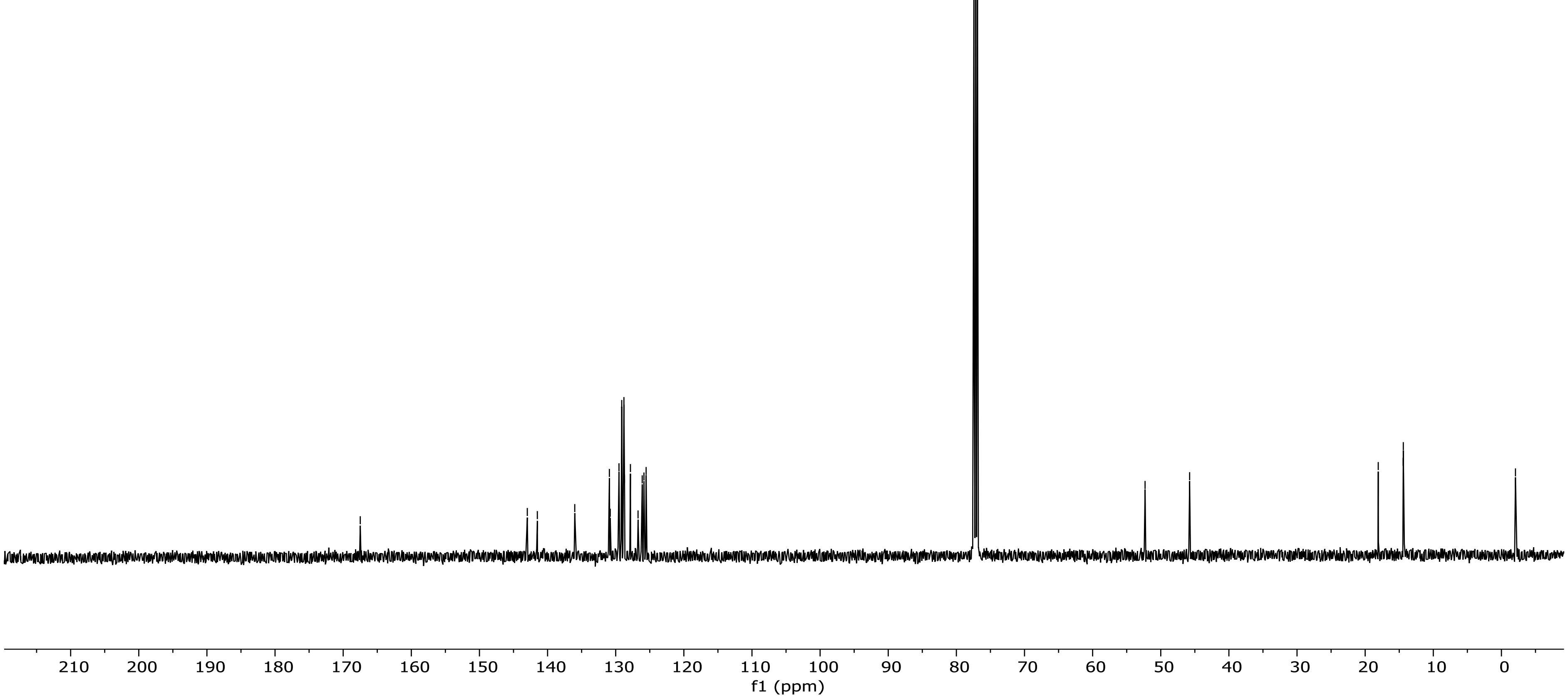

Figure S126. ^{29}Si NMR ($^{1}H/^{29}Si$ HMQC, 99 MHz, $CDCl_3$, optimized for J = 7 Hz) of **3ae.**

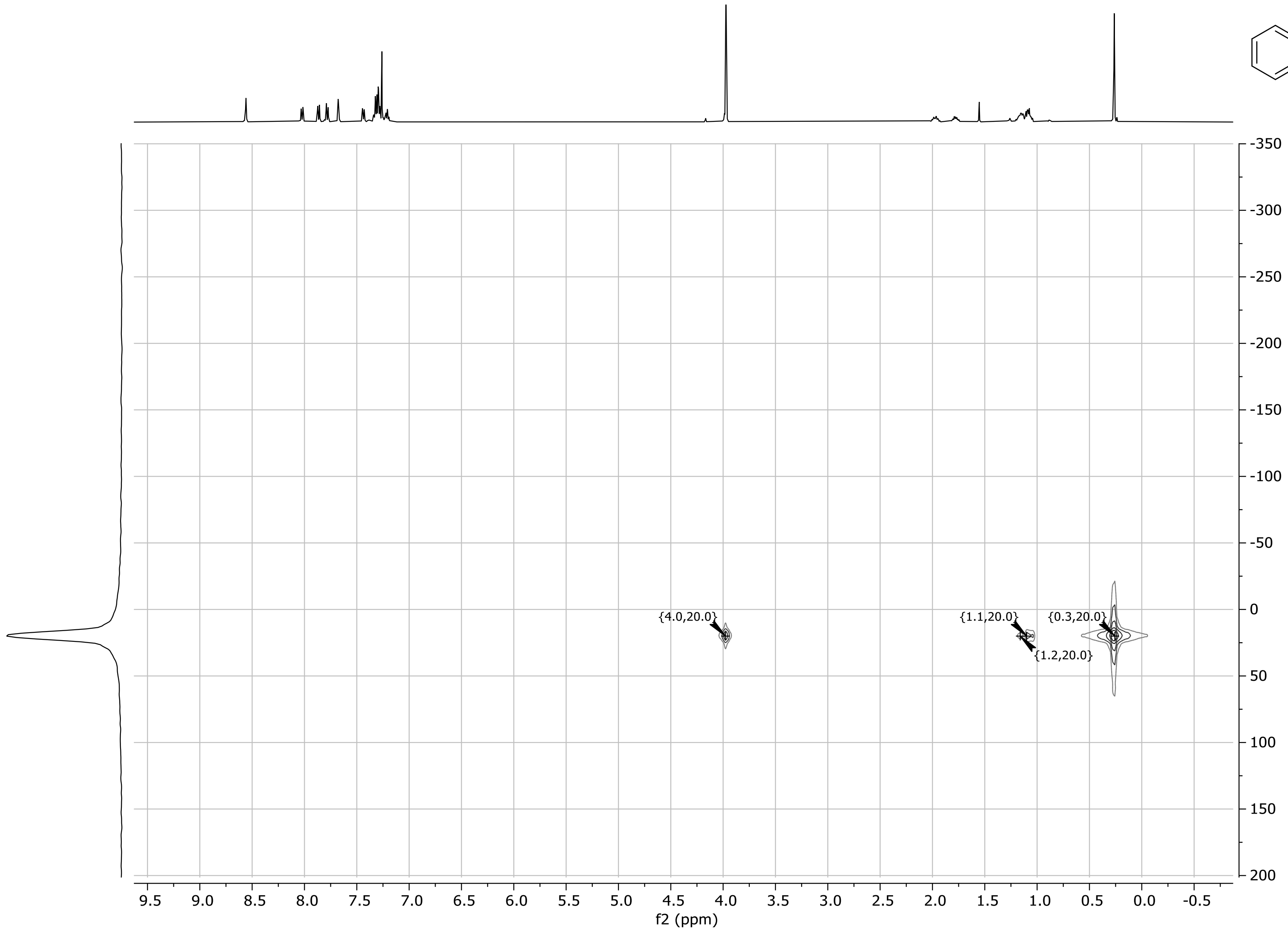

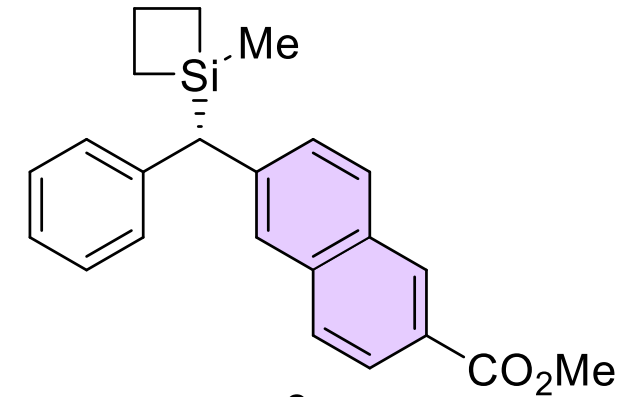

3ae
$C_{23}H_{24}O_2Si$
M_W = 360.53

Figure S127. **^{1}H NMR** (500 MHz, $CDCl_3$, 298 K) of **7aa**.

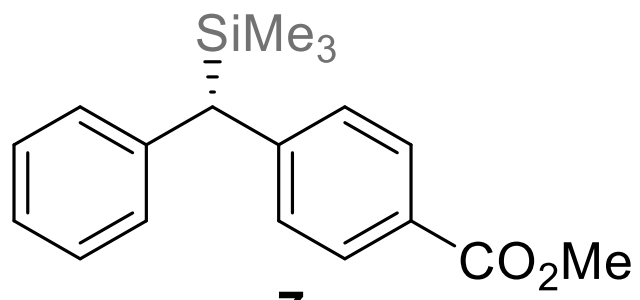

7aa
$C_{18}H_{22}O_2Si$
M_W = 298.46

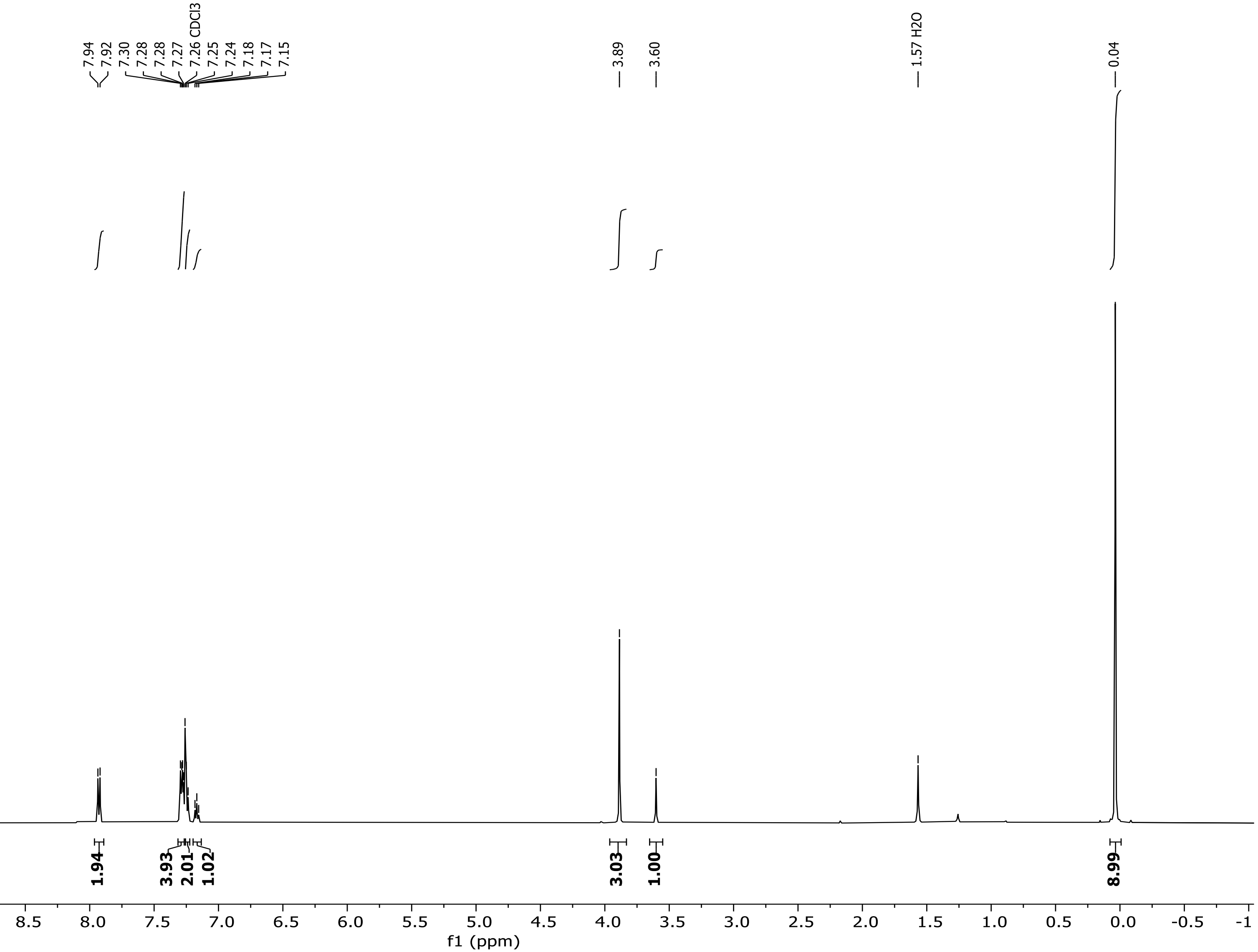

Figure S128. **^{13}C NMR** (100 MHz, $CDCl_3$, 298 K) of **7aa**.

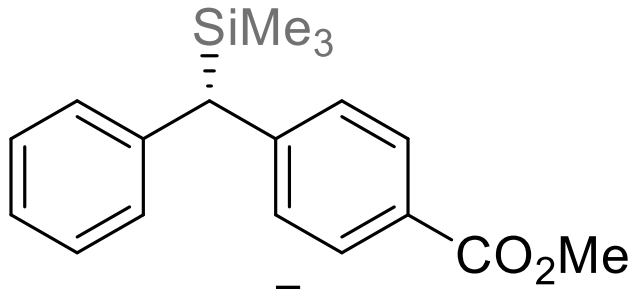

7aa

$C_{18}H_{22}O_2Si$

M_W = 298.46

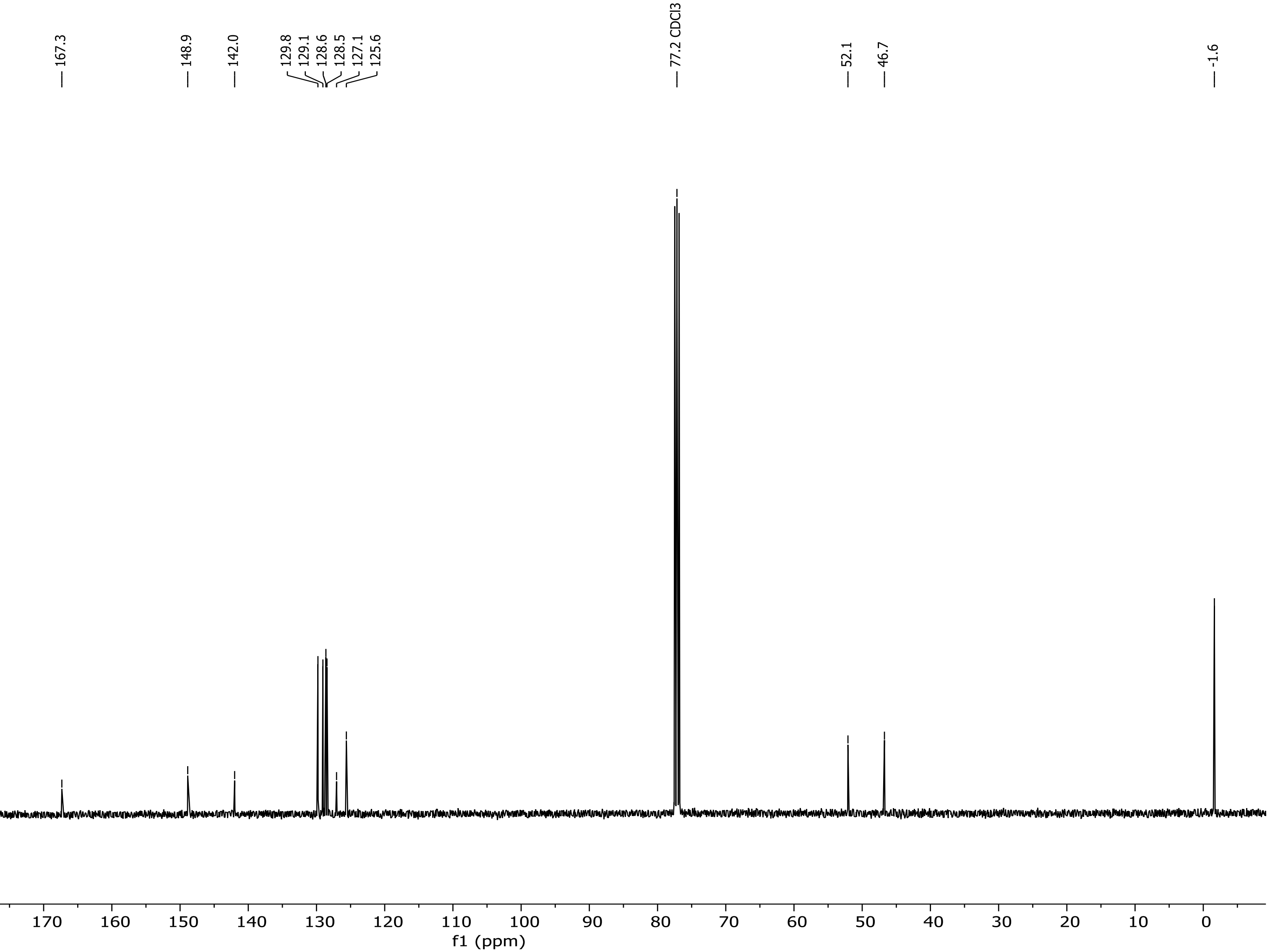

Figure S129. ^{29}Si NMR ($^{1}H/^{29}Si$ HMQC, 99 MHz, $CDCl_3$, optimized for J = 7 Hz) of **7aa.**

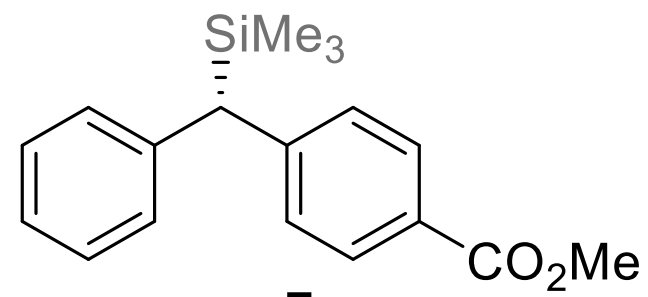

7aa
$C_{18}H_{22}O_2Si$
M_W = 298.46

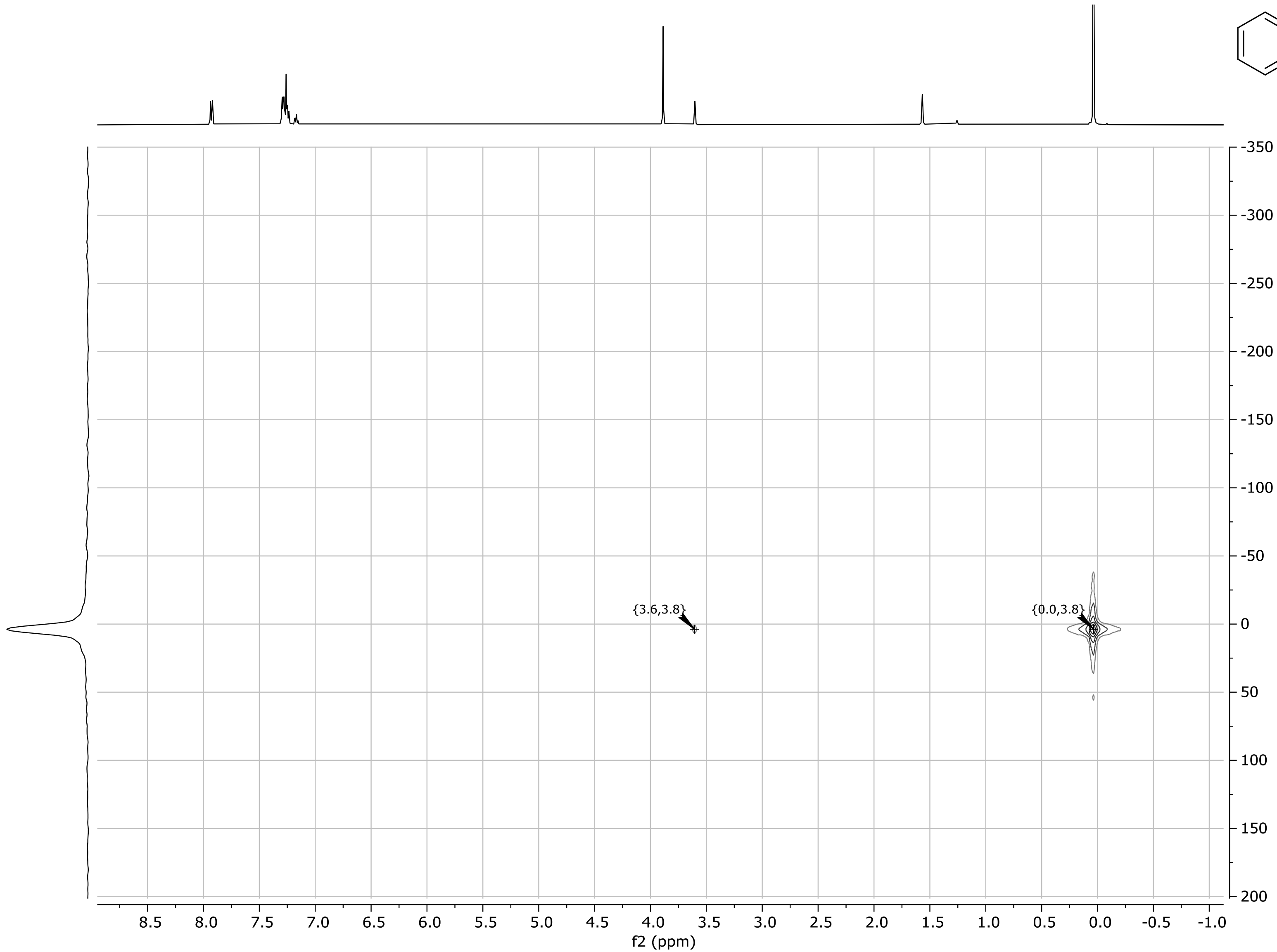

Figure S130. ^{1}**H NMR** (500 MHz, $CDCl_3$, 298 K) of **5aa**.

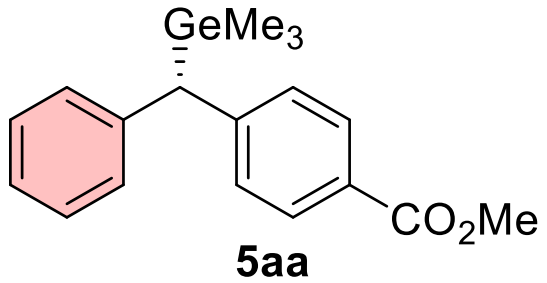

5aa

$C_{18}H_{22}GeO_2$

M_W = 343.00

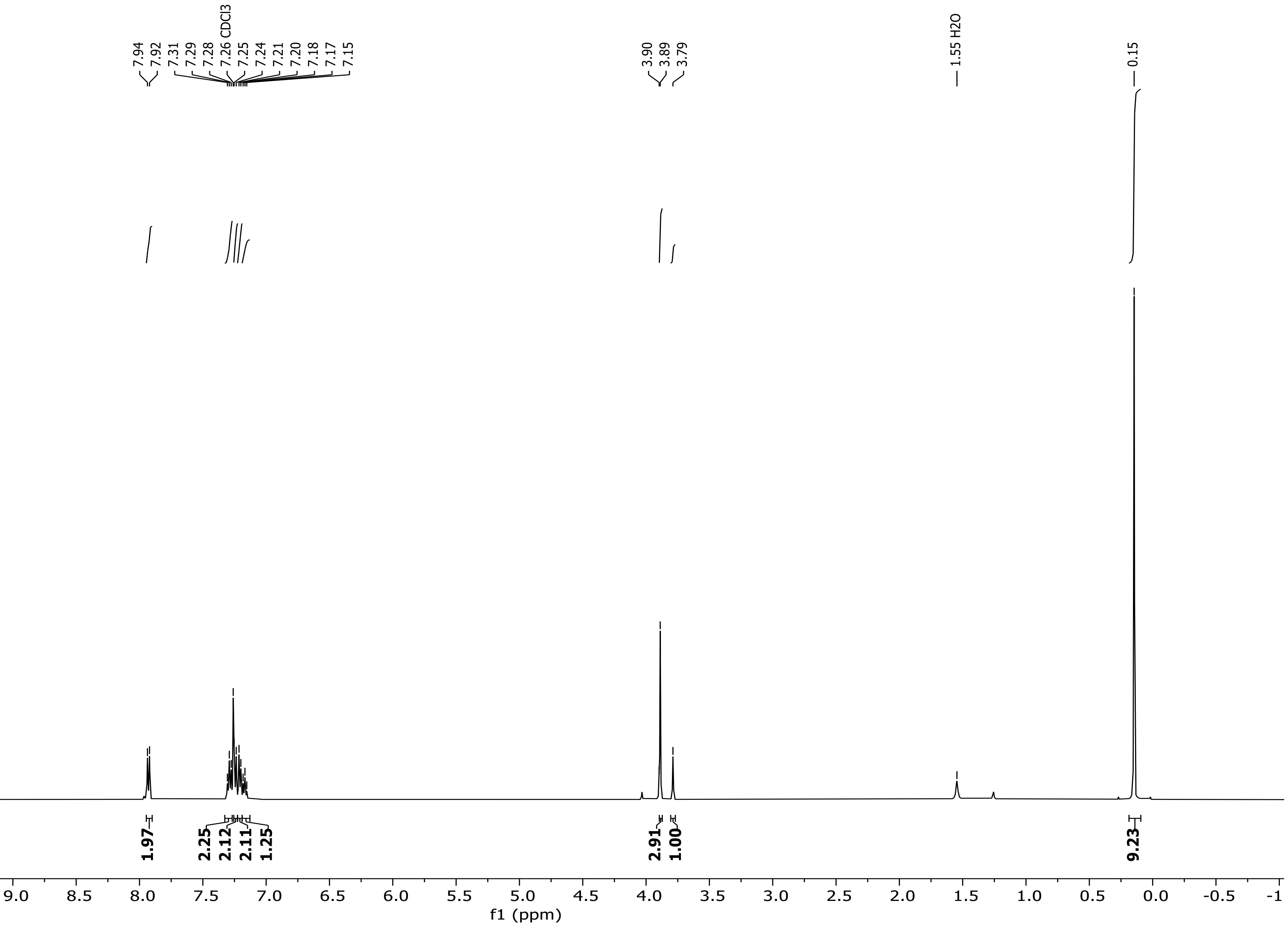

Figure S131. **^{13}C NMR** (100 MHz, $CDCl_3$, 298 K) of **5aa**.

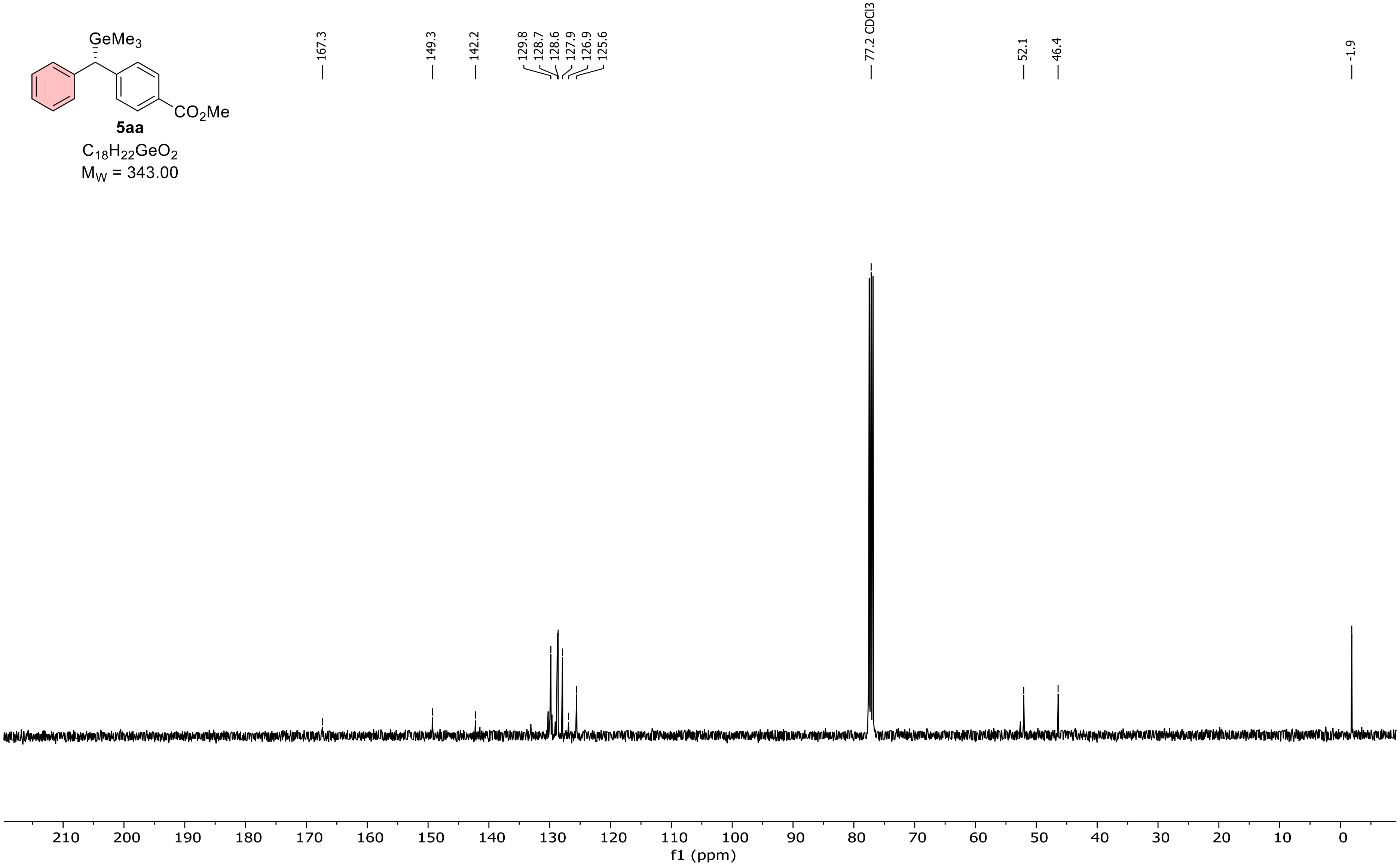

Figure S132. **^{1}H NMR** (500 MHz, $CDCl_3$, 298 K) of **5ba**.

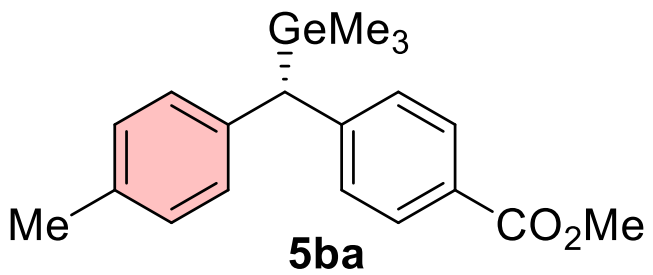

5ba

$C_{19}H_{24}GeO_2$

M_W = 357.03

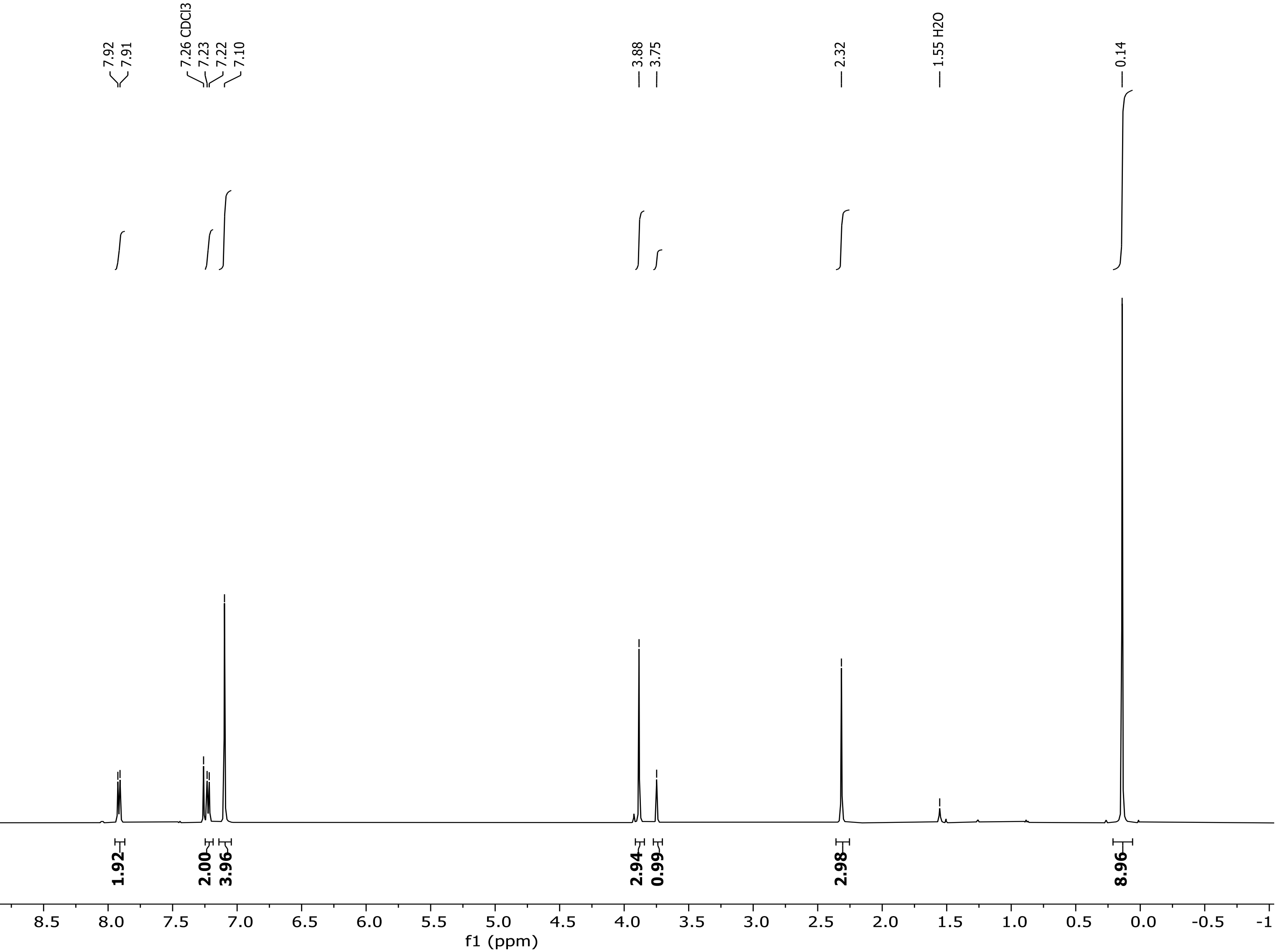

Figure S133. ^{13}**C NMR** (100 MHz, $CDCl_3$, 298 K) of **5ba**.

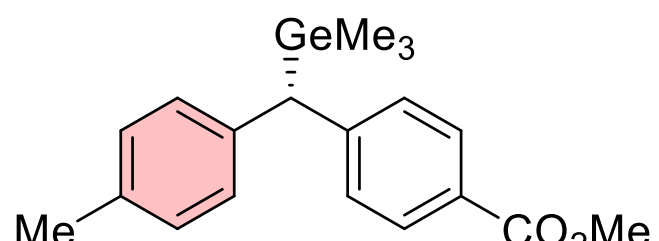

5ba
$C_{19}H_{24}GeO_2$
M_W = 357.03

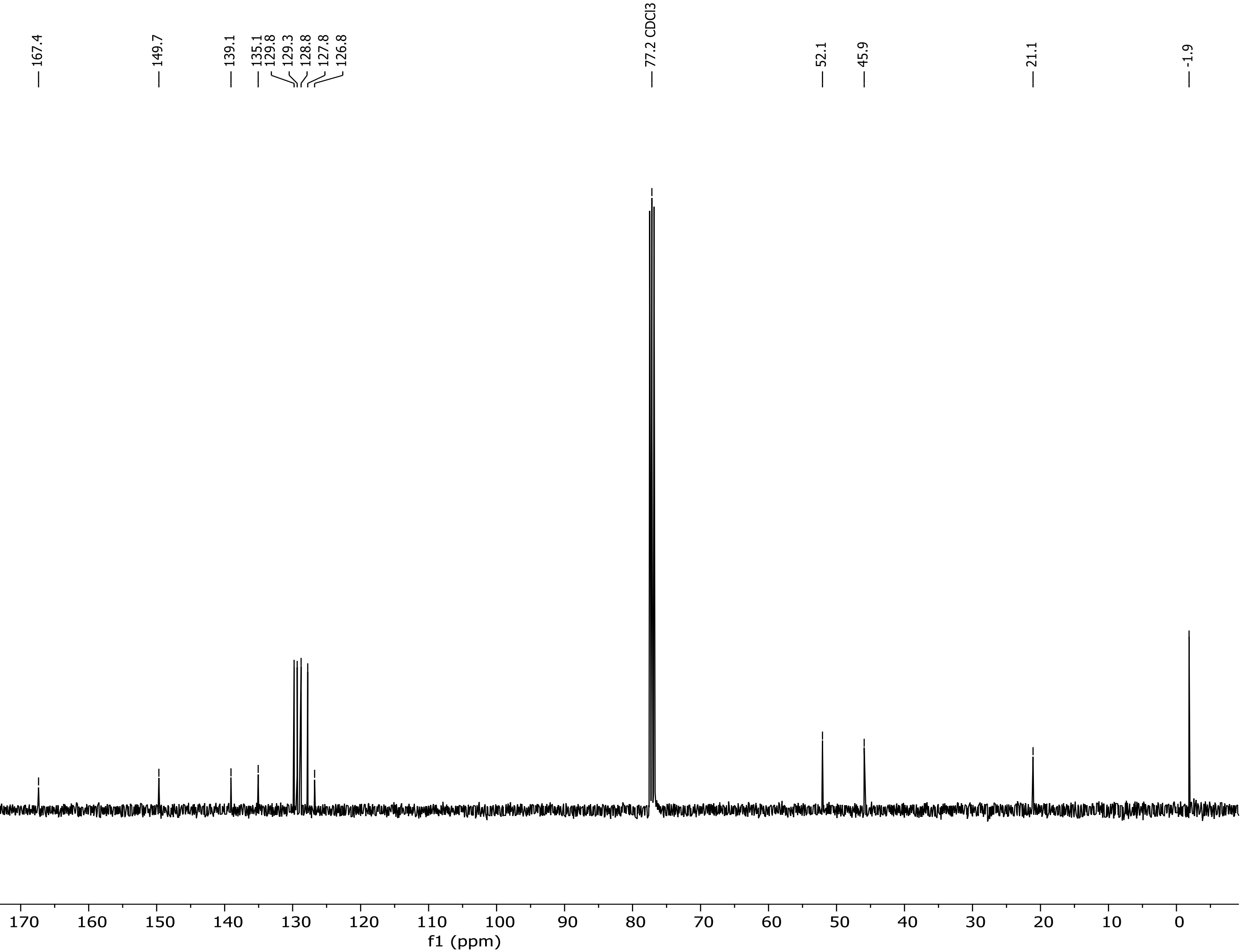

Figure S134. **¹H NMR** (500 MHz, $CDCl_3$, 298 K) of **5ca**.

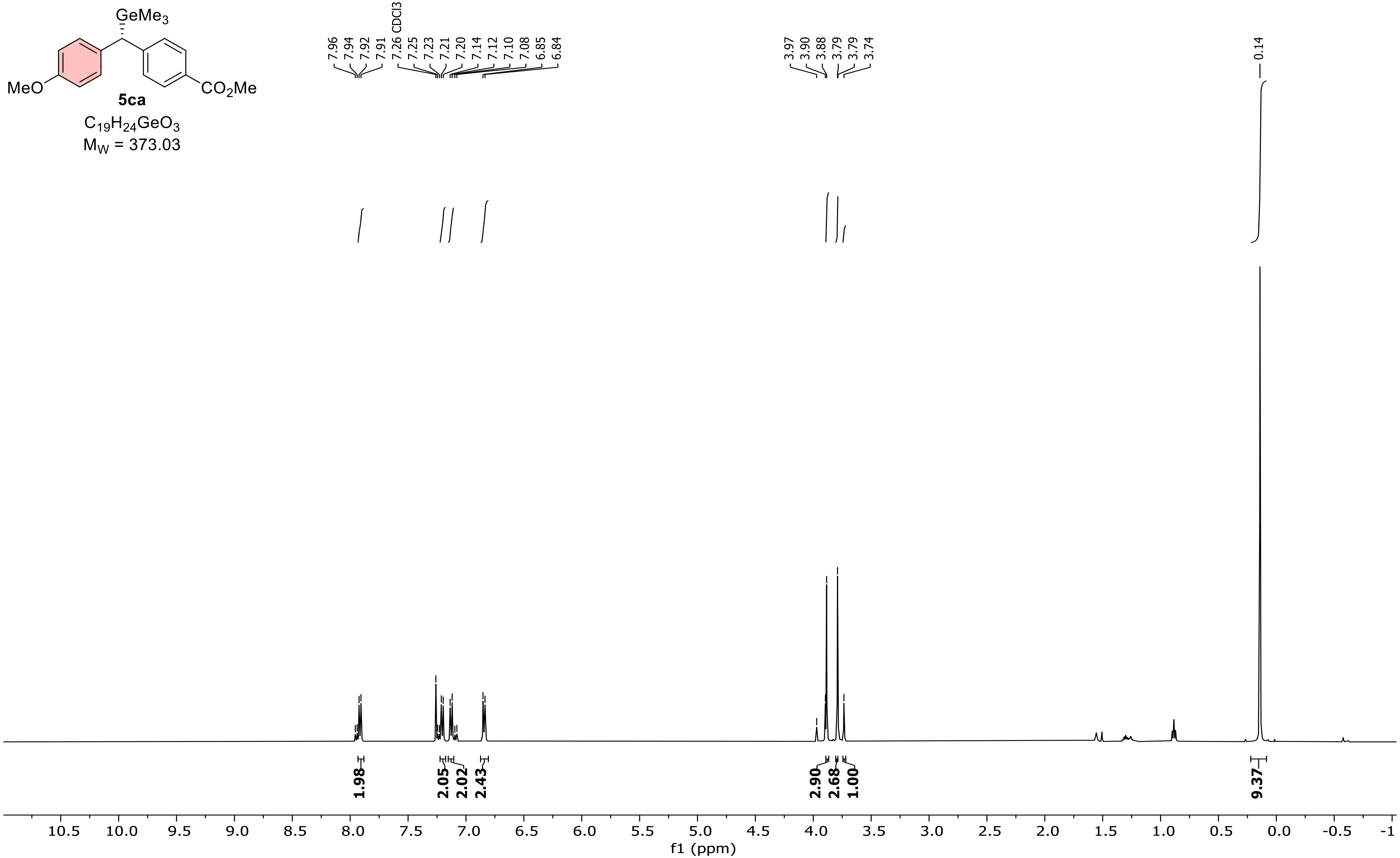

Figure S135. ^{13}**C NMR** (100 MHz, $CDCl_3$, 298 K) of **5ca**.

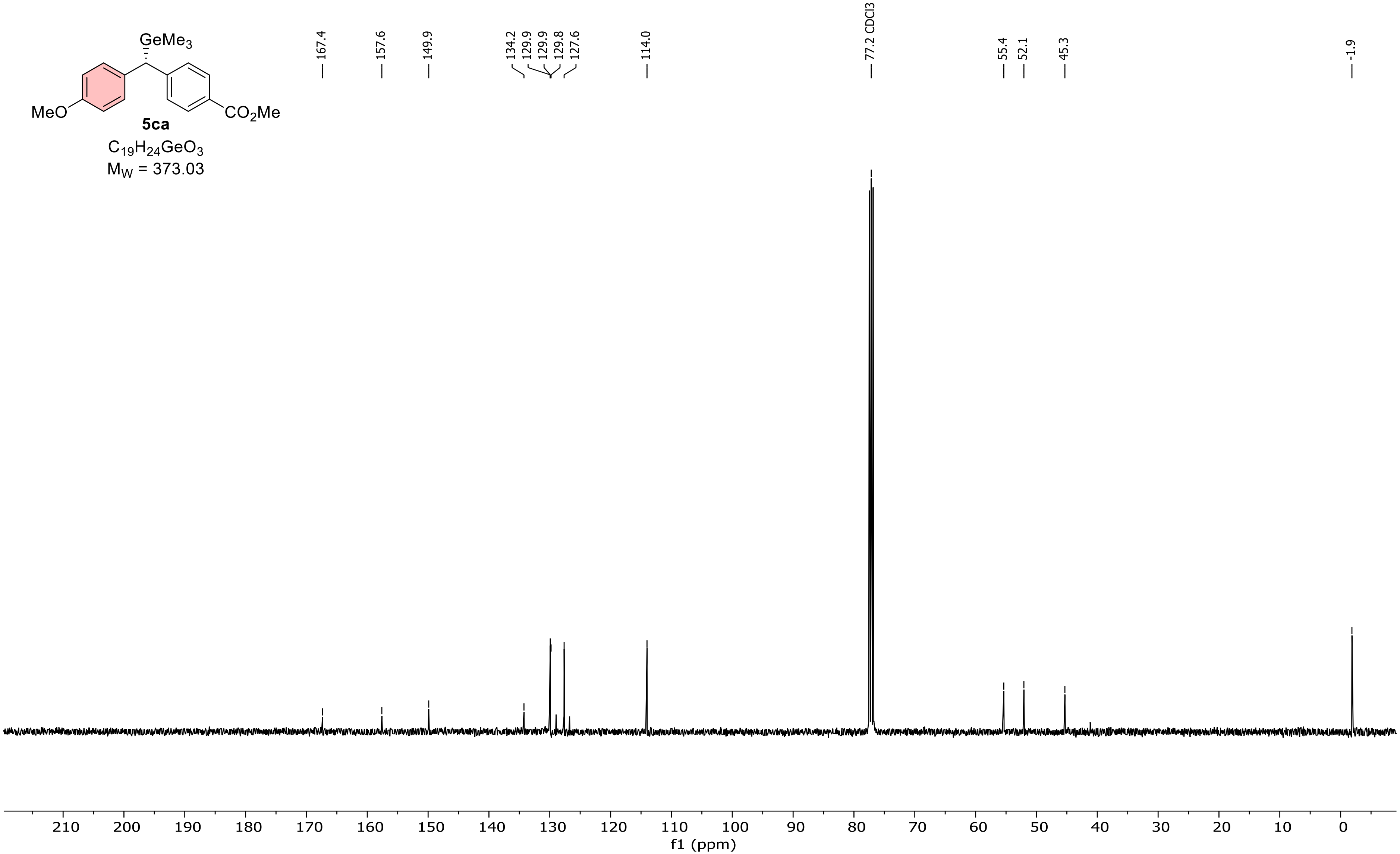

Figure S136. **¹H NMR** (500 MHz, $CDCl_3$, 298 K) of **5da**.

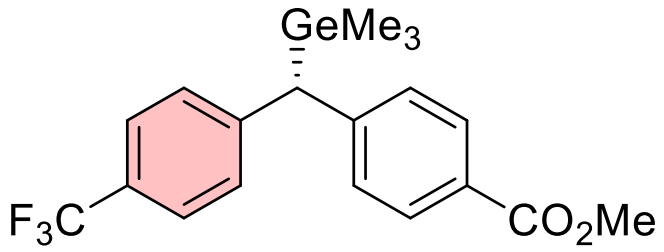

5da
$C_{19}H_{21}F_3GeO_2$
M_W = 411.00

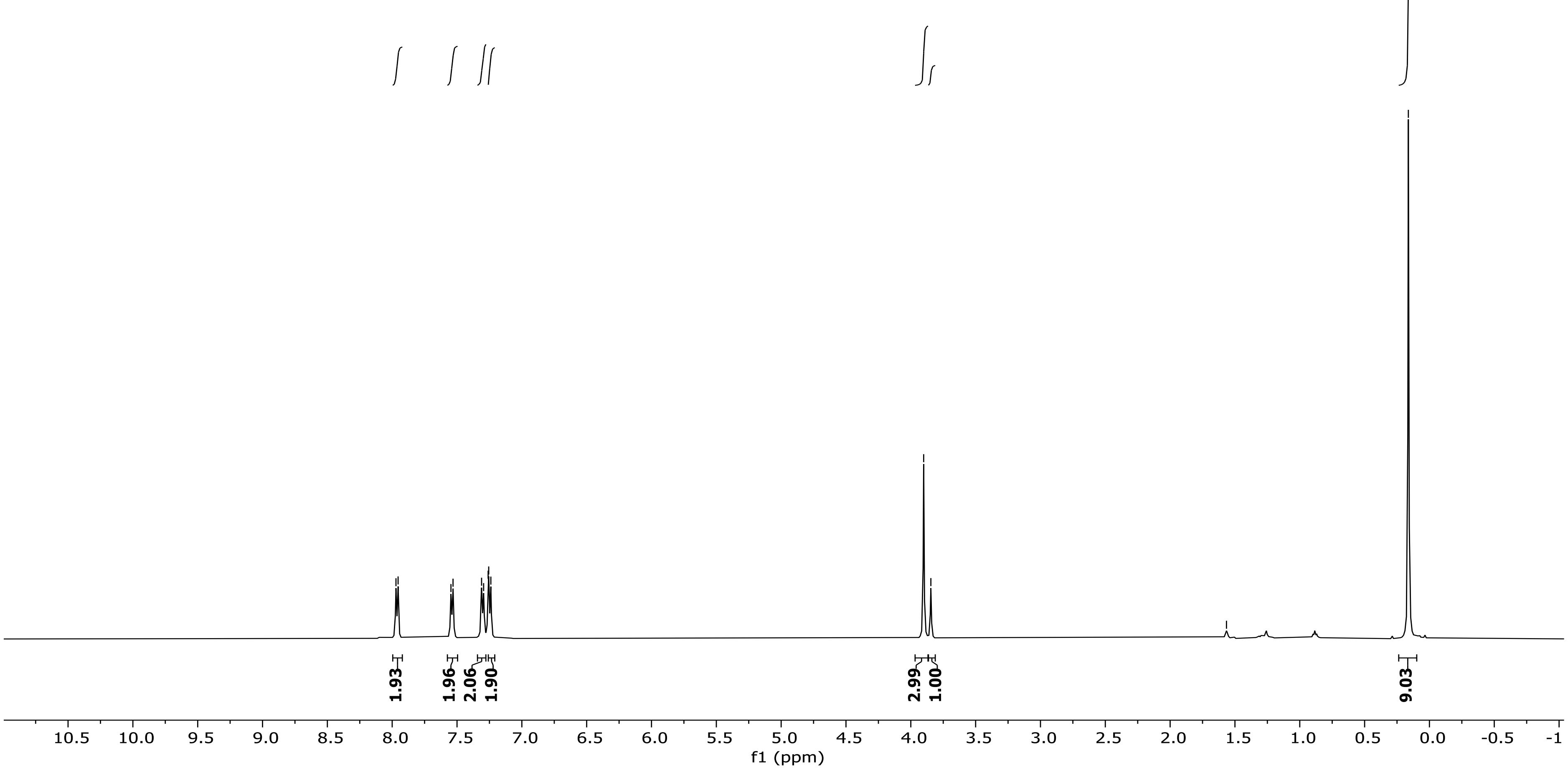

Figure S137. **^{13}C NMR** (125 MHz, $CDCl_3$, 298 K) of **5da**.

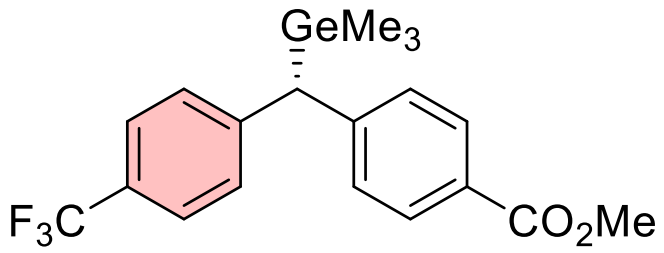

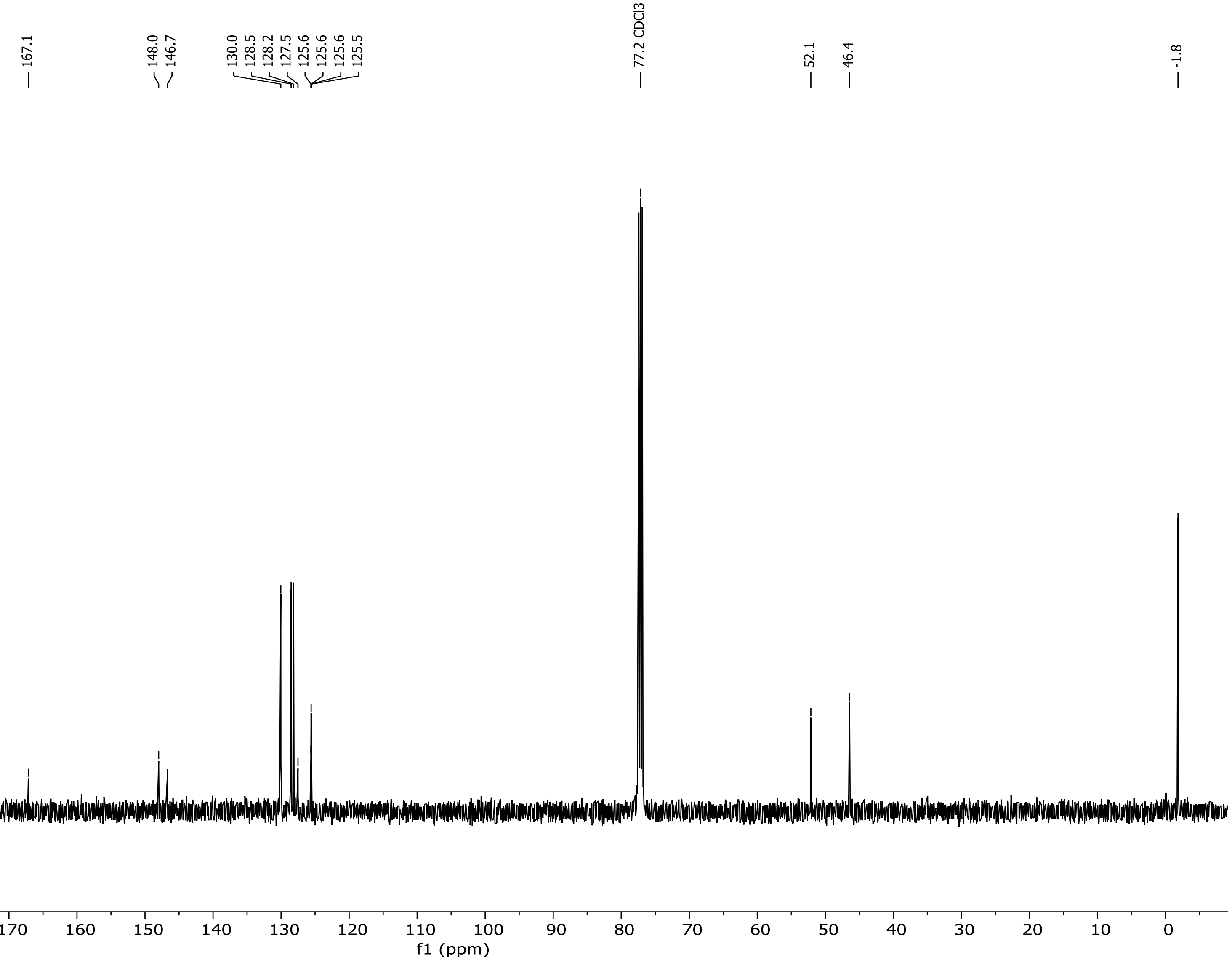

Figure S138. ^{19}**F NMR** (470 MHz, $CDCl_3$, 298 K) of **5da**.

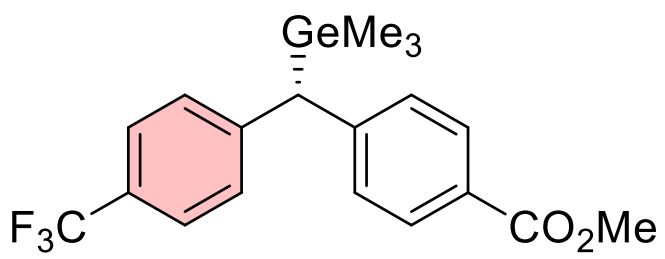

5da

$C_{19}H_{21}F_3GeO_2$

M_W = 411.00

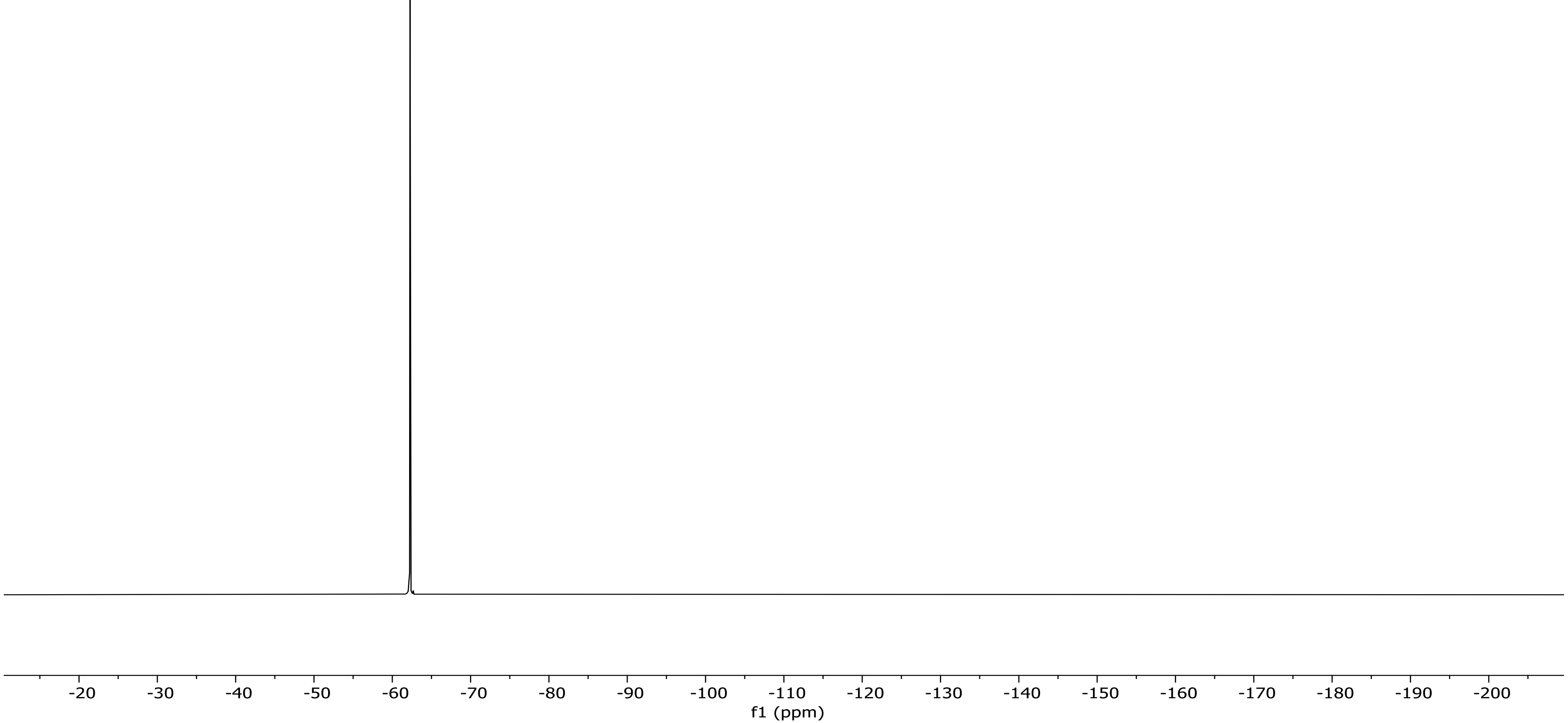

Figure S139. **^{1}H NMR** (500 MHz, $CDCl_3$, 298 K) of **5ab**.

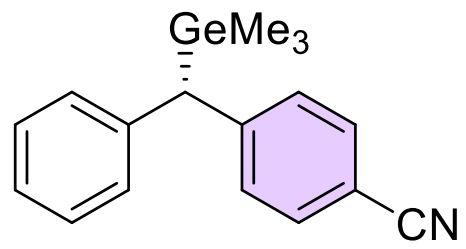

5ab
$C_{17}H_{19}GeN$
M_W = 309.98

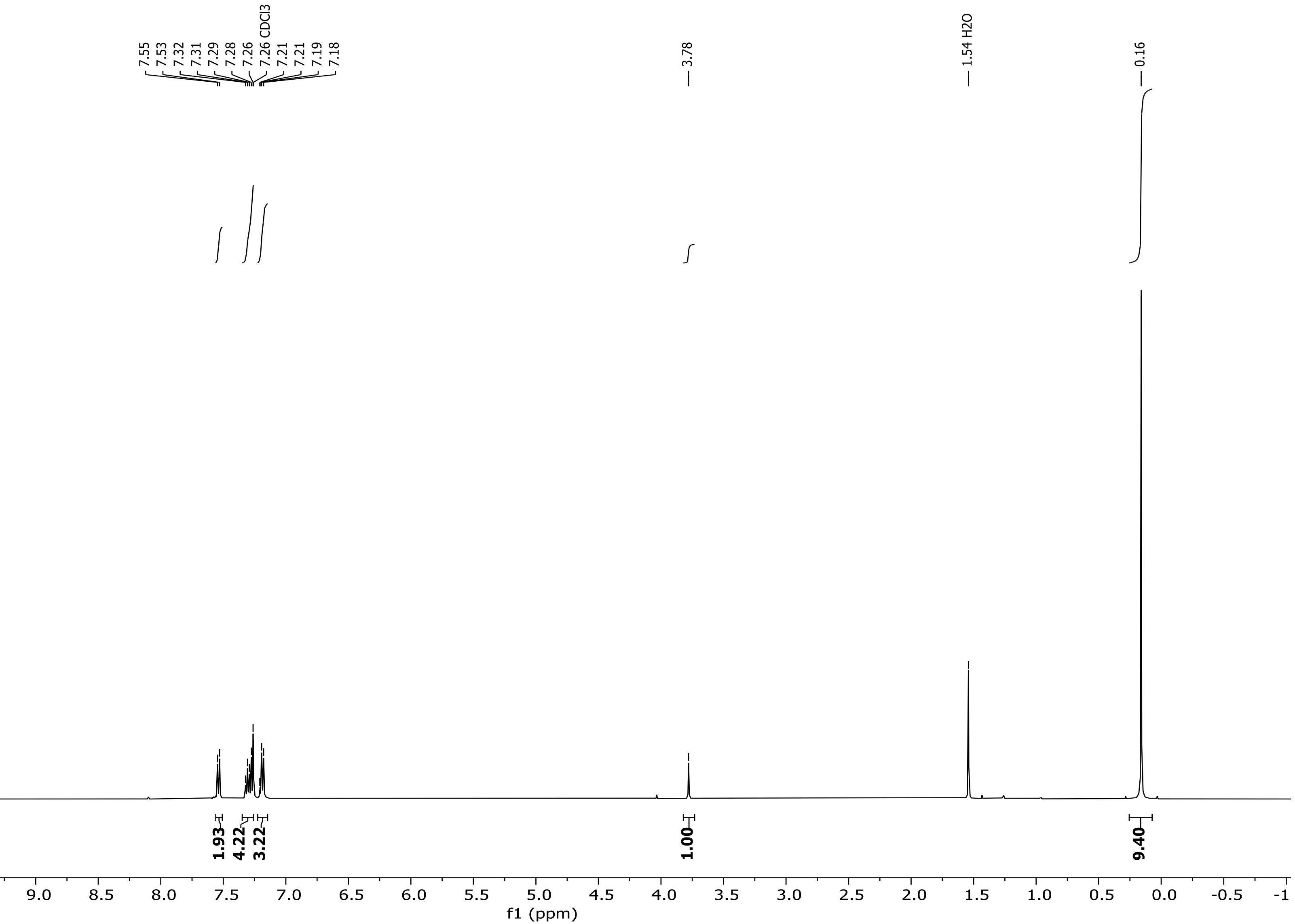

Figure S140. ^{13}C NMR (125 MHz, $CDCl_3$, 298 K) of **5ab**.

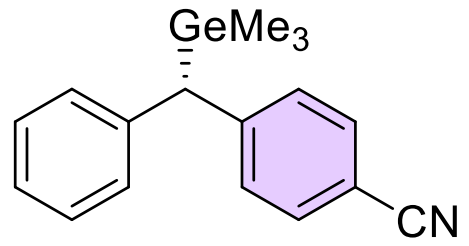

5ab
$C_{17}H_{19}GeN$
M_W = 309.98

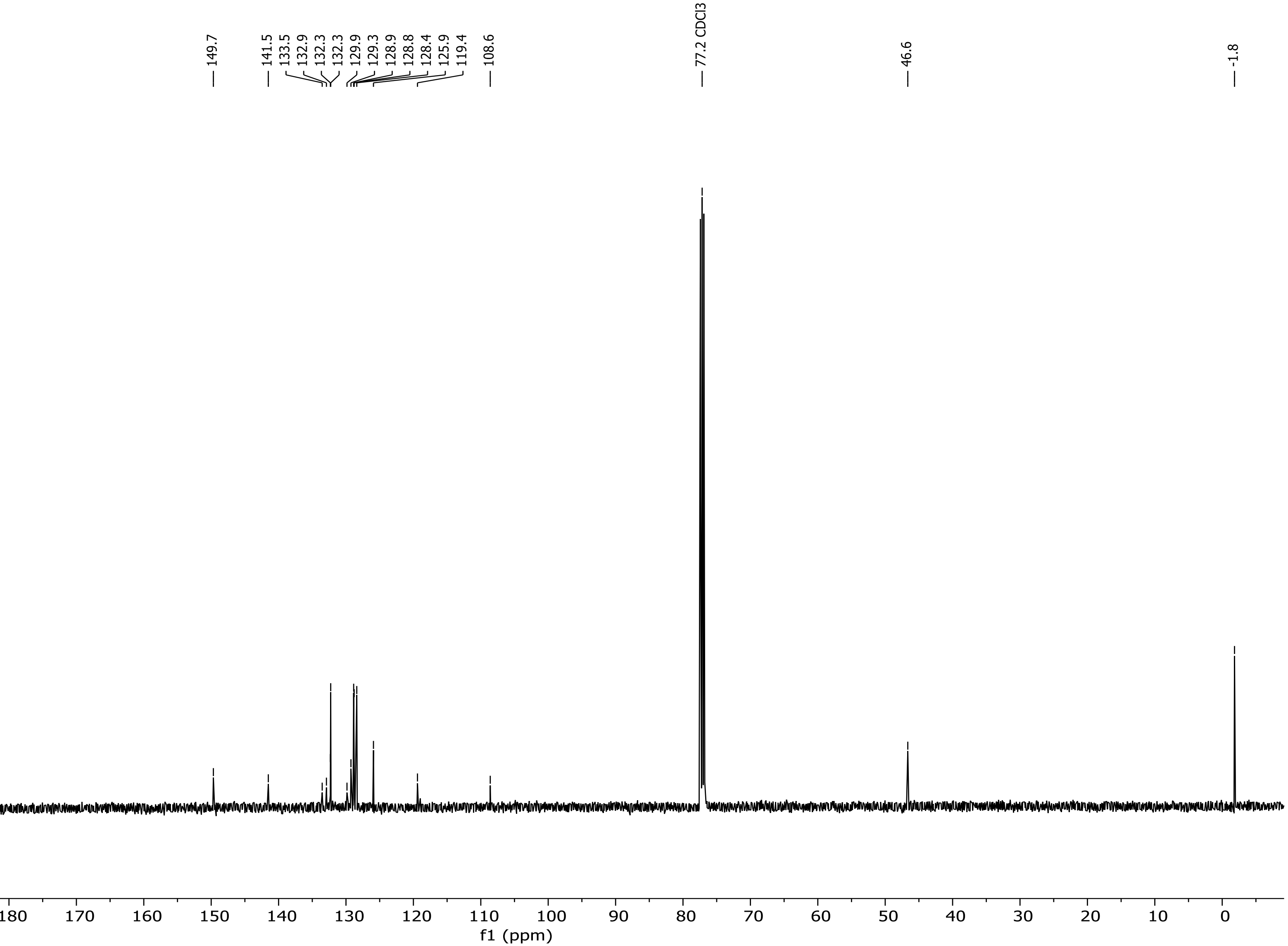

Figure S141. **^{1}H NMR** (500 MHz, $CDCl_3$, 298 K) of **5ah**.

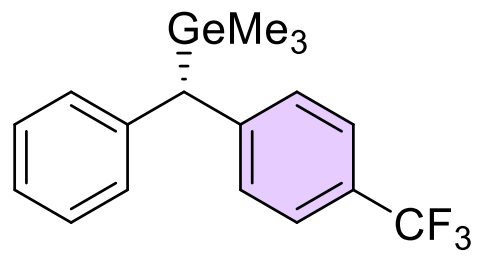

5ah
$C_{17}H_{19}F_3Ge$
M_W = 352.96

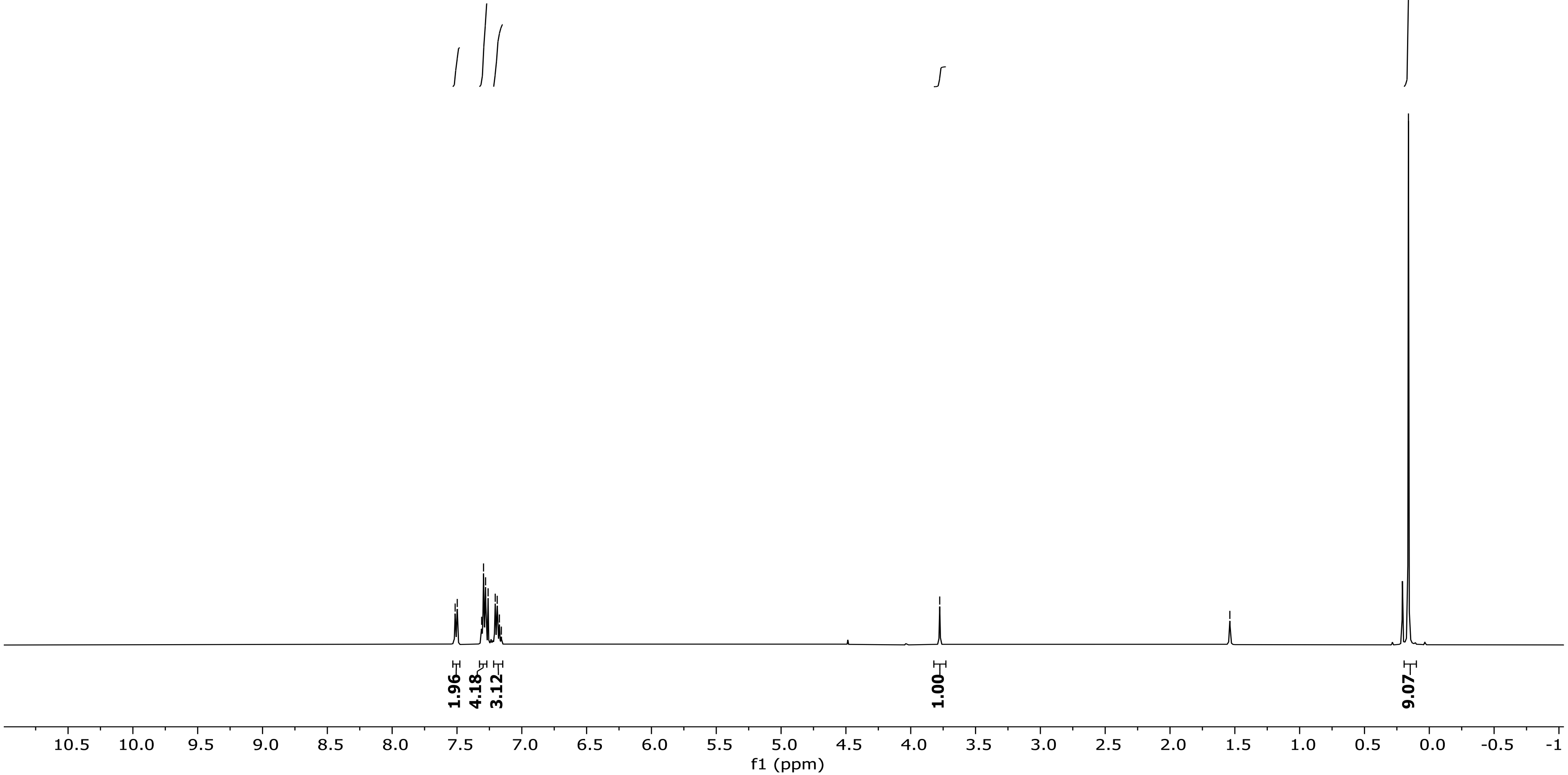

Figure S142. ^{13}**C NMR** (125 MHz, $CDCl_3$, 298 K) of **5ah**.

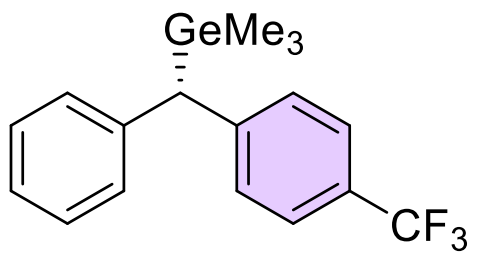

5ah
$C_{17}H_{19}F_3Ge$
M_W = 352.96

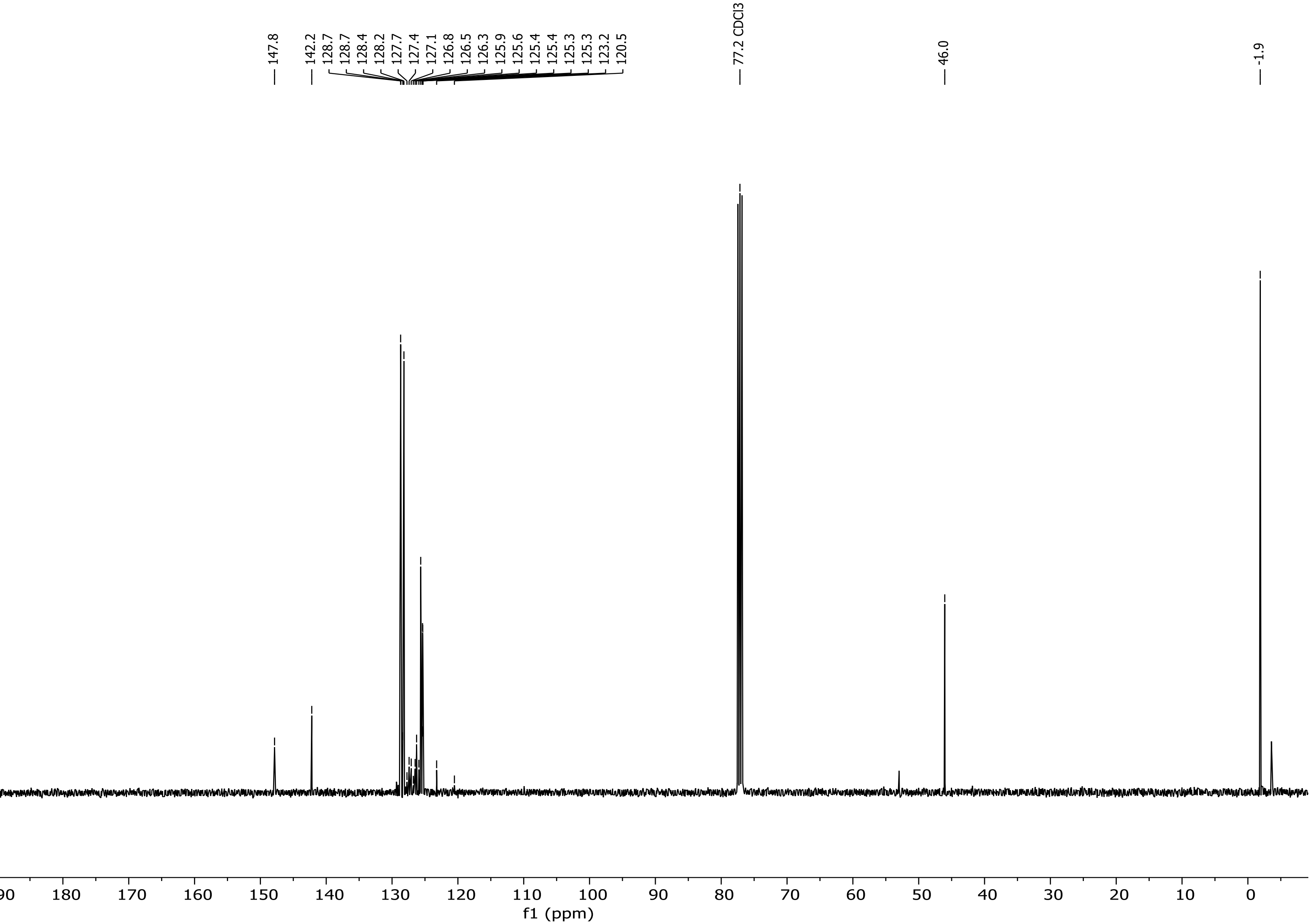

Figure S143. ^{19}**F NMR** (470 MHz, $CDCl_3$, 298 K) of **5ah**.

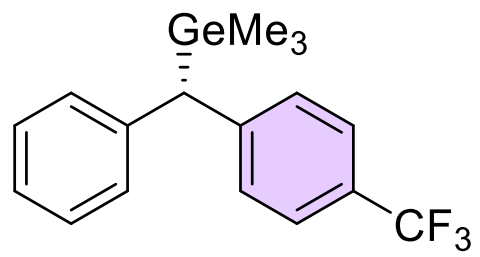

5ah
$C_{17}H_{19}F_3Ge$
M_W = 352.96

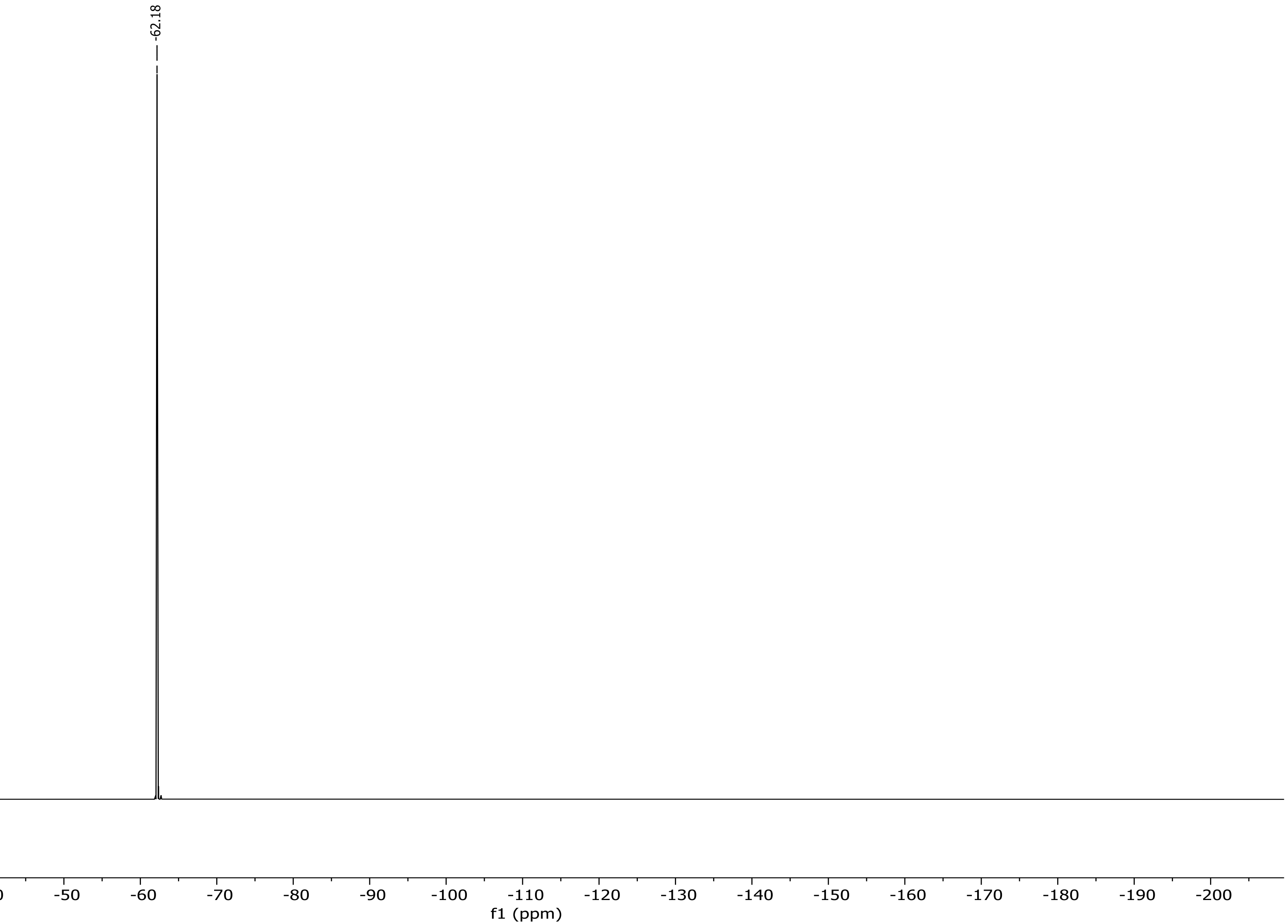

Figure S144. **¹H NMR** (500 MHz, $CDCl_3$, 298 K) of **5ai**.

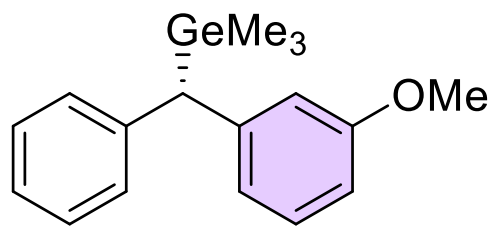

5ai
$C_{17}H_{22}GeO$
M_W = 314.99

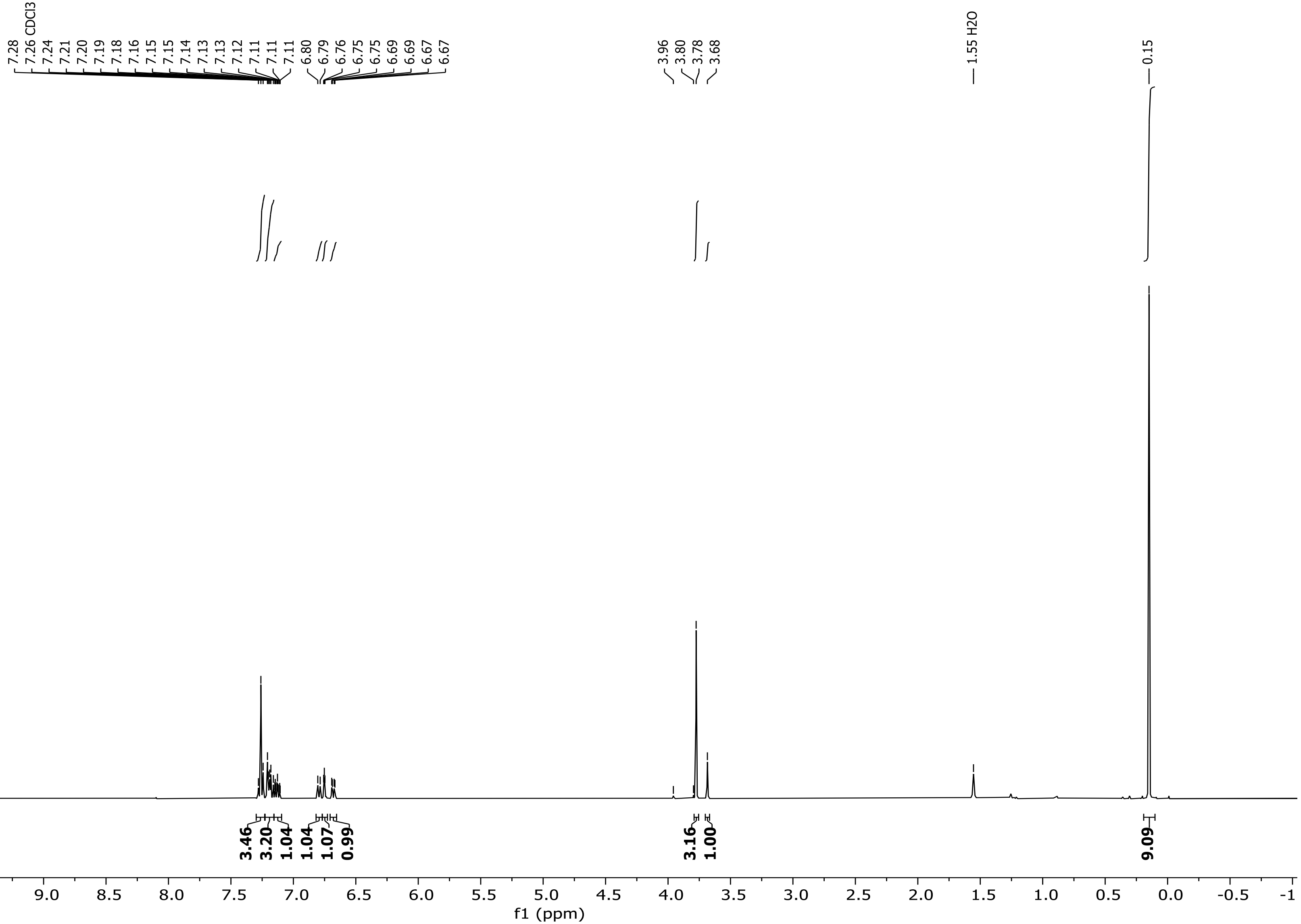

Figure S145. ^{13}**C NMR** (125 MHz, $CDCl_3$, 298 K) of **5ai**.

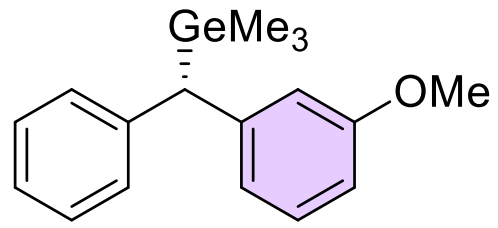

5ai
$C_{17}H_{22}GeO$
M_W = 314.99

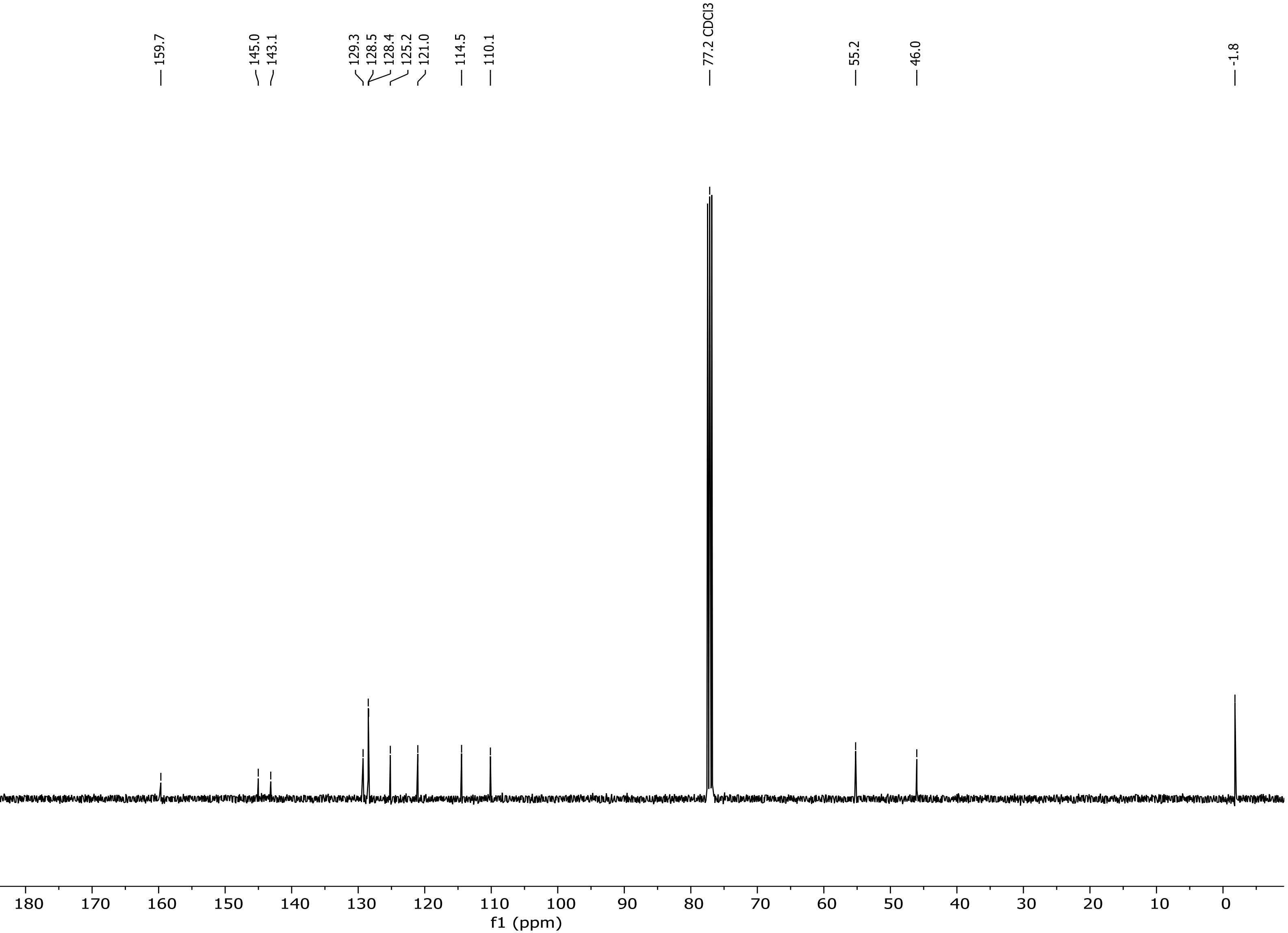

Figure S146. ¹H NMR (500 MHz, $CDCl_3$, 298 K) of **9aa**.

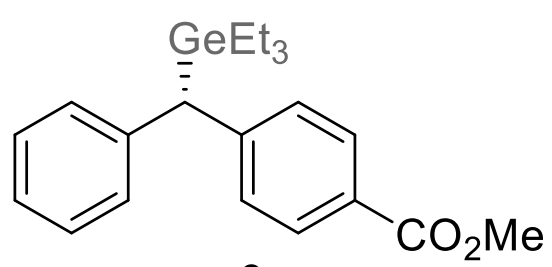

9aa

$C_{21}H_{28}GeO_2$

M_W = 385.08

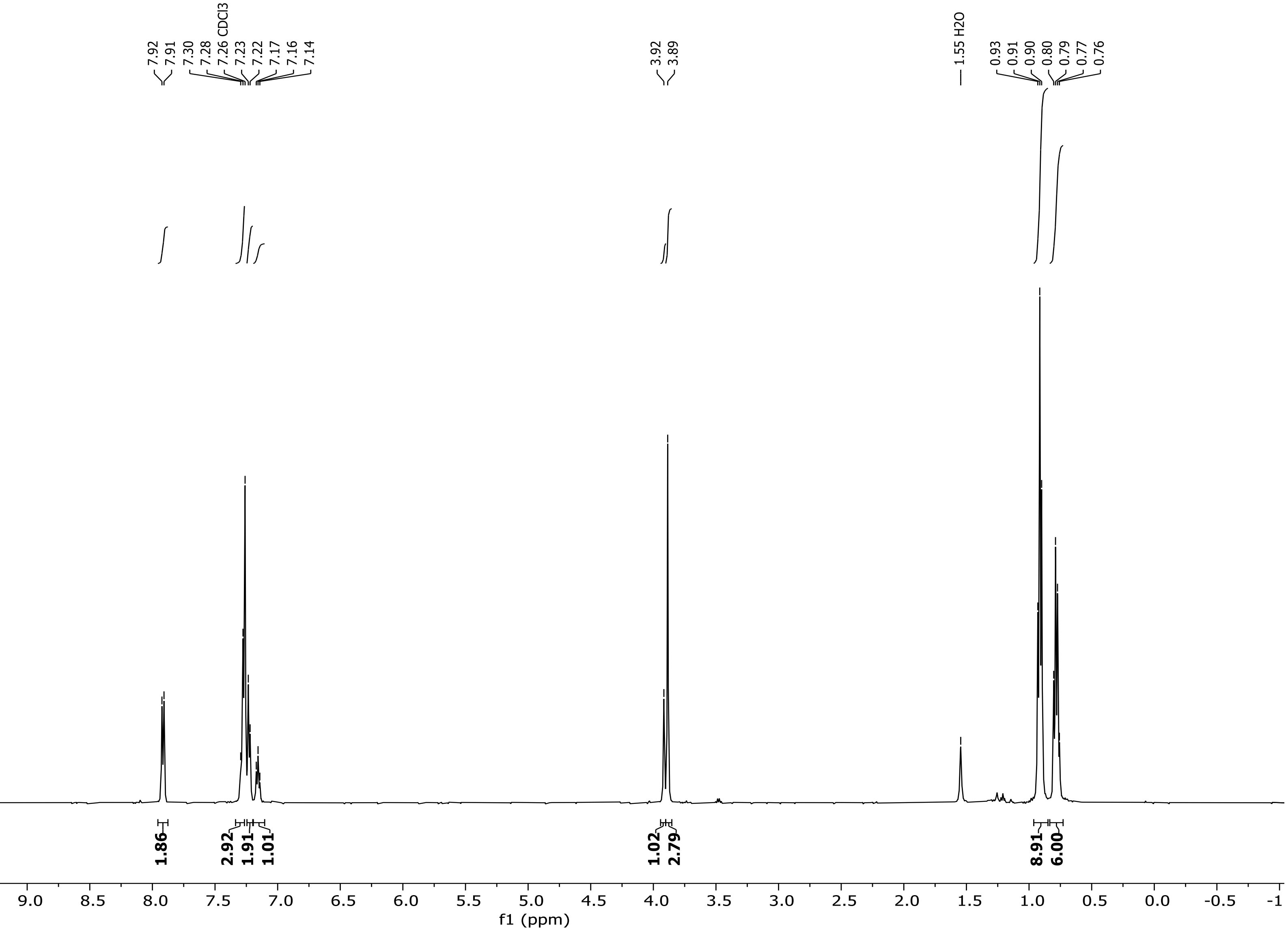

Figure S147. ^{13}**C NMR** (125 MHz, $CDCl_3$, 298 K) of **9aa**.

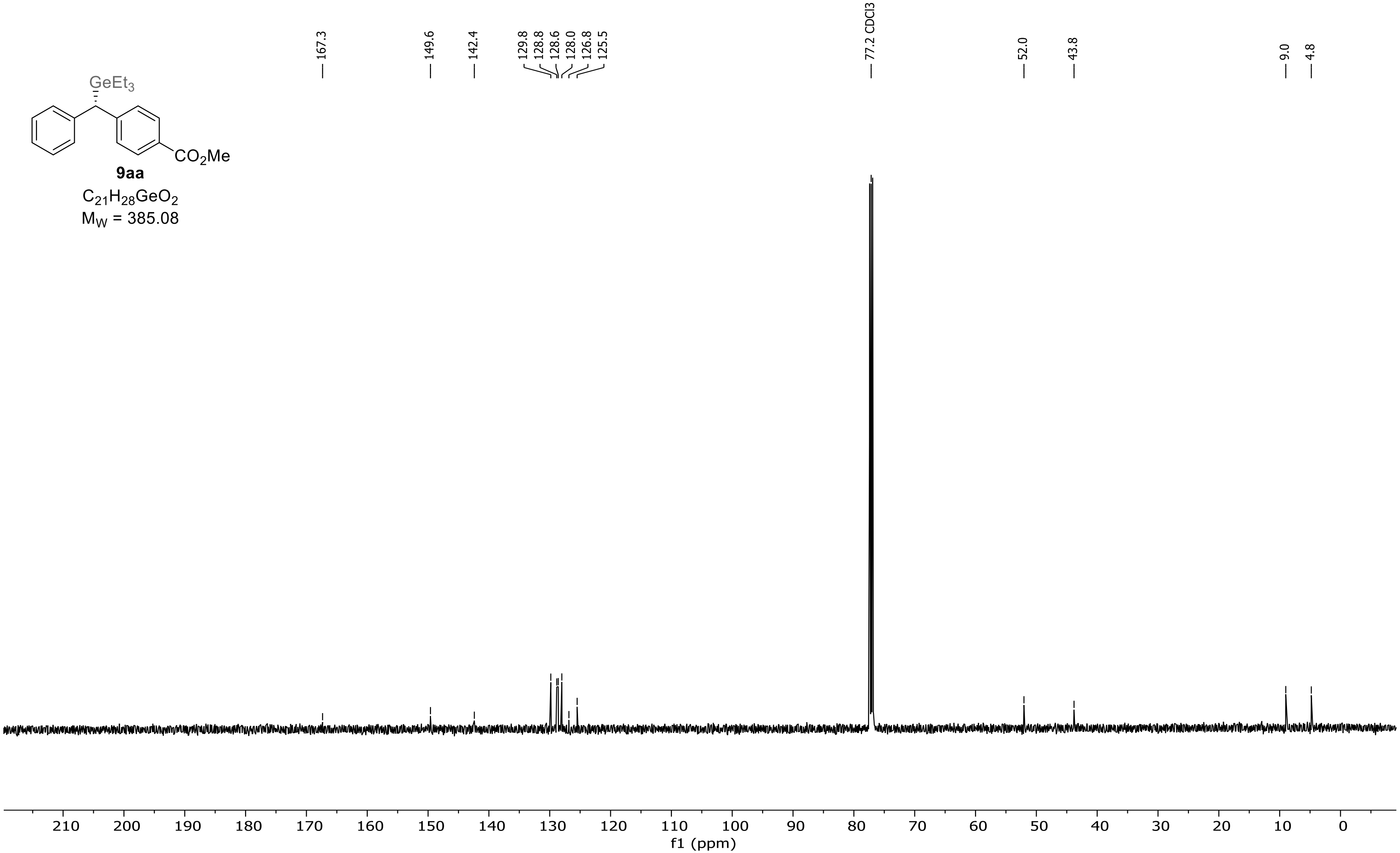

11. References

S1 T. Li, X. Cheng, J. Lu, H. Wang, Q. Fang and Z. Lu, *Chin. J. Chem.*, 2022, **40**, 1033–1038.

S2 E. G. A. Acuña-Bolomey and M. Oestreich, *Synthesis*, 2025, **57**, 3273–3282.

S3 X. Cheng, H. Lu and Z. Lu, *Nat. Commun.*, 2019, **10**, 3549.

S4 X. Cheng, T. Li, Y. Liu and Z. Lu, *ACS Catal.*, 2021, **11**, 11059–11065.

S5 Y.-Z. Wang, B. Sun, J.-F. Guo, X.-Y. Zhu, Y.-C. Gu, Y.-P. Han, C. Ma and T.-S. Mei, *Nat. Commun.*, 2025, **16**, 1108.

S6 J. L. Hofstra, A. H. Cherney, C. M. Ordner and S. E. Reisman, *J. Am. Chem. Soc.*, 2018, **140**, 139–142.
